# Supplementary material for: Enhanced and unified anatomical labeling for a common mouse brain atlas
Source: Nat Commun. 2019 Nov 7;10:5067. doi: 10.1038/s41467-019-13057-w (PMC6838086; doi:10.1038/s41467-019-13057-w)
Supplement: Supplementary file 1 — Supplementary Information [file 41467_2019_13057_MOESM1_ESM.pdf]

Supplementary Information

Enhanced and Unified Anatomical Labeling for a Common Mouse Brain Atlas

Chon et al.,

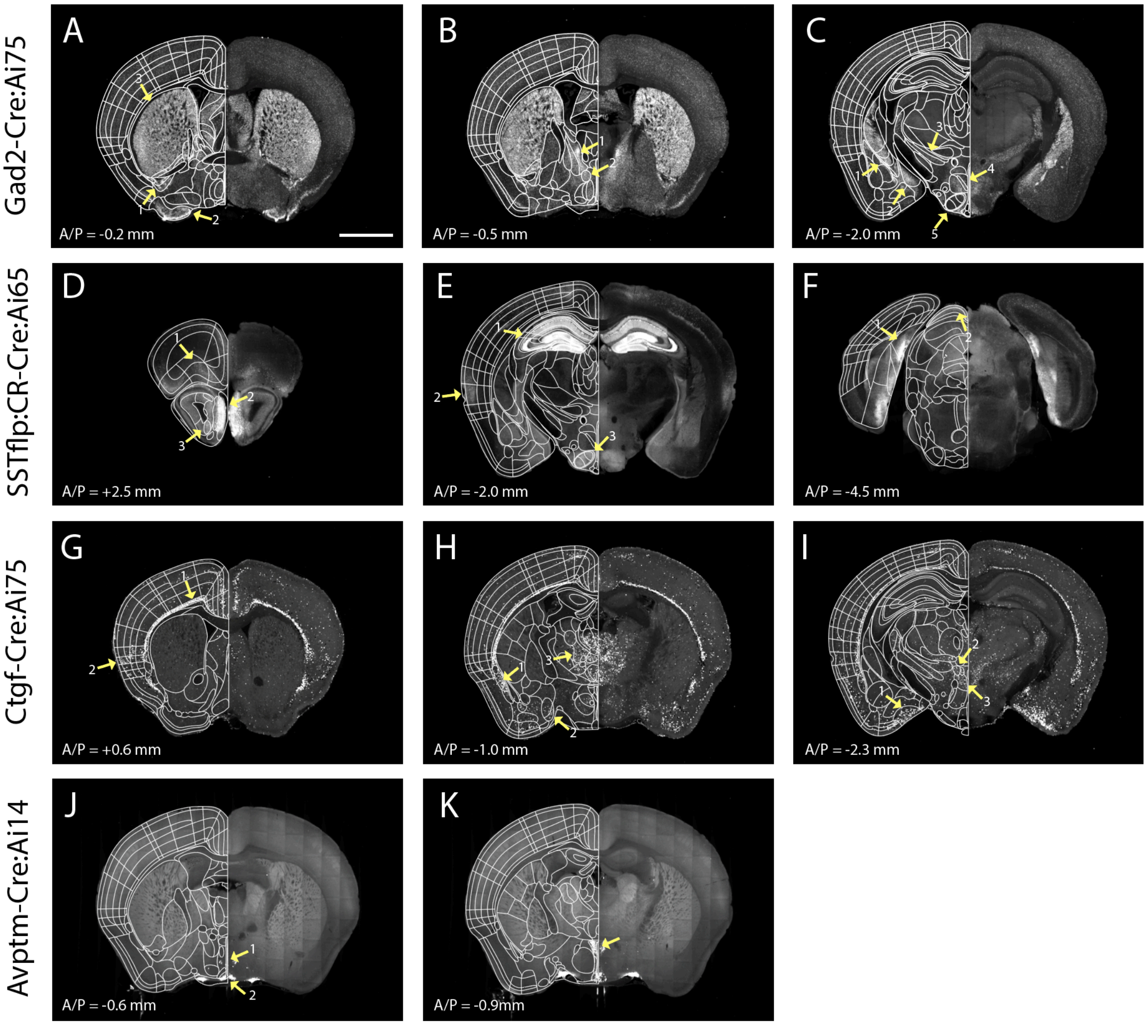

Supplementary Figure 1. **Additional marker brains used for alignment of anatomical borders in our FP based labels**

(A-C) Gad2-Cre:Ai75 brain to delineate (A) the interstitial nucleus of the posterior limb of the anterior commissure (arrow 1), the olfactory tubercle (arrow 2) and the caudate putamen (arrow 3), Scale bar = 2mm (B) the bed nuclei of the stria terminalis medial division posteromedial part (arrow 1), the striohypothalamic nucleus (arrow 2), and (C) the central amygdaloid nuclei (arrow 1), the medial amygdala posterodorsal division (arrow 2), the zona incerta (arrow 3) and other hypothalamic structures such as the dorsomedial hypothalamic nucleus, ventral part (arrow 4) and the ventromedial hypothalamic nucleus (arrow 5). (D-F) SSTflp:CR-Cre:Ai65 transgenic mouse line to delineate the following structures: (D) the orbital cortex area (arrow 1), layers of the olfactory bulb (arrow 2), the anterior olfactory area ventral part (arrow 3), (E) the CA2 of hippocampus (arrow 1), the ectorhinal cortex (arrow 2), the ventromedial hypothalamic nucleus (arrow 3), (F) the postsubiculum (arrow 1), and the superficial gray layer of the superior colliculus (arrow 2). (G-I) Ctgf-Cre:Ai75 transgenic mouse line to delineate the following structures: (G) layer 6b of the isocortex (arrow 1), the granular insular cortex (arrow 2), (H) the dorsal endopiriform nucleus (arrow 1), the medial amygdalar nucleus

anterodorsal (arrow 2), the thalamic structures such as the anteromedial thalamic nucleus (arrow 3), (I) the posteromedial cortical amygdala (arrow 1), the subparafascicular thalamic nucleus (arrow 2), and the posterior hypothalamic nucleus dorsal part (arrow 3). (J-K) Avptm-Cre:Ai14 transgenic mouse line to delineate the following structures: (J) the medial hypothalamic area (arrow 1), the suprachiasmatic nucleus ventromedial part (arrow 2), and (K) the paraventricular hypothalamic nuclei (arrow).

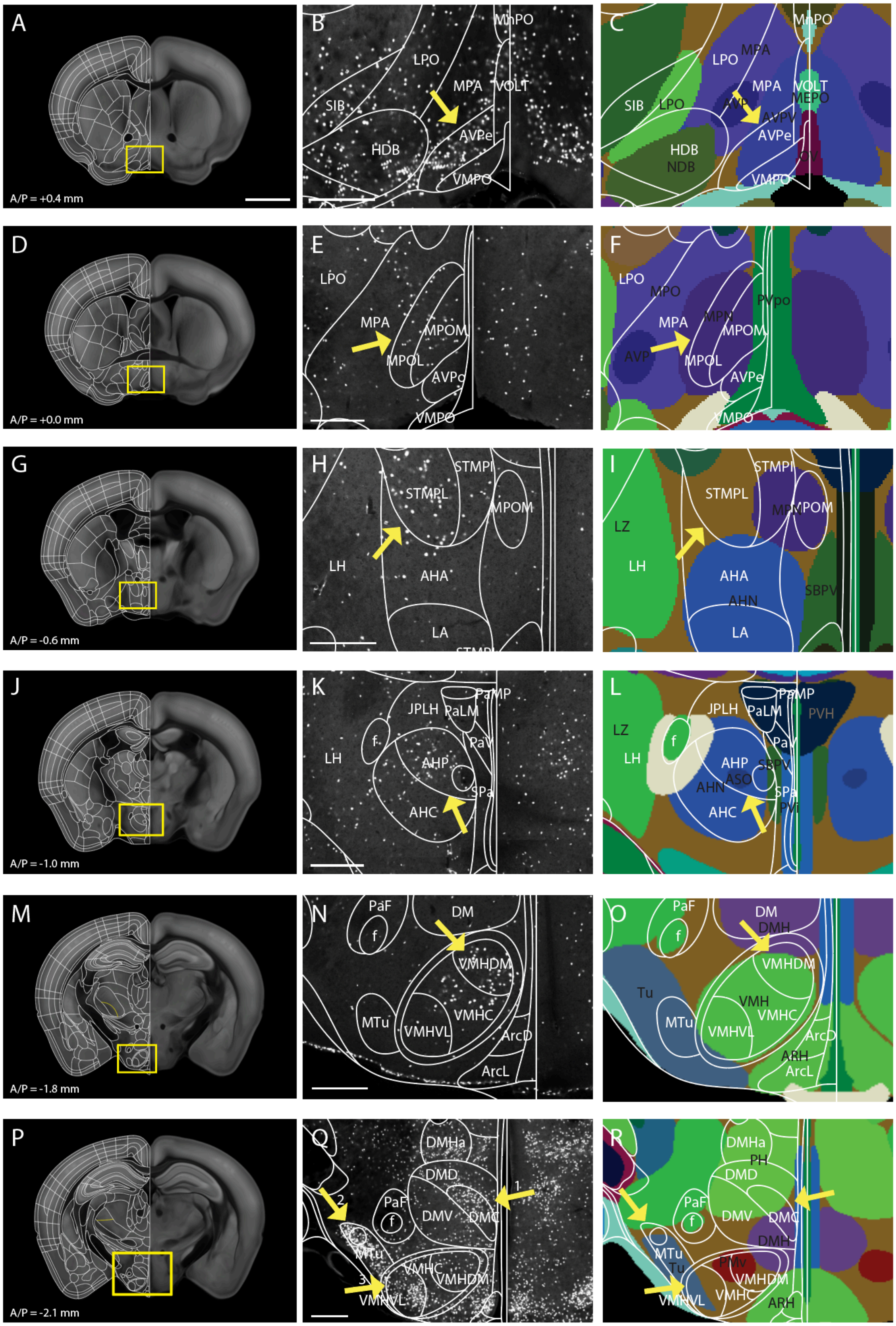

A
A/P = +0.4 mm
B
MnPO
LPO
MPA
VOLT
SIB
AVPe
HDB
VMPO
C
MnPO
MPA
LPO
MPA
VOLT
MEPO
SIB
LPO
AVP
AVPV
AVPe
HDB
NDB
VMPO
D
A/P = +0.0 mm
E
LPO
MPA
MPOM
MPOL
AVPe
VMPO
F
LPO
MPO
PVpo
MPA
MPN
MPOM
AVP
MPOL
AVPe
VMPO
G
A/P = -0.6 mm
H
STMPI
STMPL
MPOM
LH
AHA
LA
I
STMPI
STMPL
MPOM
LZ
LH
AHA
AHN
SBPV
LA
J
A/P = -1.0 mm
K
PaMP
JPLH
PaLM
f
Pa
LH
AHP
SPa
AHC
L
PaMP
JPLH
PaLM
PVH
LZ
f
Pa
LH
AHP
SBPV
AHN
ASO
SPa
AHC
PVi
M
A/P = -1.8 mm
N
PaF
DM
f
VMHDM
MTu
VMHC
VMHVL
ArcD
ArcL
O
PaF
DM
f
DMH
VMHDM
Tu
VMH
MTu
VMHC
VMHVL
ArcD
ARH
ArcL
P
A/P = -2.1 mm
Q
DMHa
DMD
2
1
PaF
f
DMV
DMC
MTu
3
VMHC
VMHDM
VMHVL
R
DMHa
PH
DMD
PaF
f
DMV
DMC
DMH
MTu
Tu
PMv
VMHDM
VMHC
VMHVL
ARH

Supplementary Figure 2. Comparison between the CCFv3 and our FP based labels in the hypothalamus

First column: Our highly segmented FP based labels on the Allen CCF. Scale bar = 2mm. Second column: our labels (white lines) with marker brain background. Scale bar = 300μm. Third column: comparison between our labels and the CCFv3 labels (colored background). Anatomical names in black and white are from the CCFv3 and our labels, respectively. (A-C) SST-Cre:H2B-GFP (B) used to delineate the anteroventral periventricular nucleus (AVPe) indicated by yellow arrow. High in cell density, AVPe was delineated more ventrally compared CCFv3 label (C). (D-F) SST-Cre:H2B-GFP (E) used to delineate the medial preoptic nucleus, lateral and medial parts (MPOL and MPOM, respectively). Our labels have a higher degree of segmentation, whereas it is defined as a single structure in the CCFv3 labels (F). (G-I) Ctgf:Ai75 (H) used to delineate the anterior hypothalamic area, anterior part (AHA). Lower in cell density compared to adjacent substructures, AHA was delineated from the bed nucleus of the stria terminalis, medial division, posterolateral part (STMPL), determining the boundary between pallidum and hypothalamus (I). (J-L) Ctgf:Ai75 (K) used to delineate the anterior hypothalamic area, posterior part (AHP). With higher cell density in the AHP compared to the central part (AHC), the boundary between the substructures was determined, compared to a single structure delineation of AHN in the CCFv3 label (L). (M-O) Ctgf:Ai75 (N) used to delineate sub-structures of the ventromedial hypothalamic nucleus (VMH). With denser population of cells in the dorsomedial part (VMHDM) compared to central and ventrolateral parts (VMHC and VMHVL, respectively), boundaries between the substructures were determined, compared to single structure delineation of VMH in the CCFv3 label (O). (P-R) SST-Cre:H2B-GFP (Q) used to delineate the dorsomedial hypothalamic nucleus, compact part (DMC, arrow 1), medial tuberal nucleus (MTu, arrow 2), and the ventromedial hypothalamic nucleus, ventrolateral part (VMHVL, arrow 3). With higher cell density in the DMC and the VMHVL compared to adjacent substructures, boundaries between the substructures were determined, compared to a single structure delineation of the DMH and the VMH, respectively, in CCFv3 label (R).

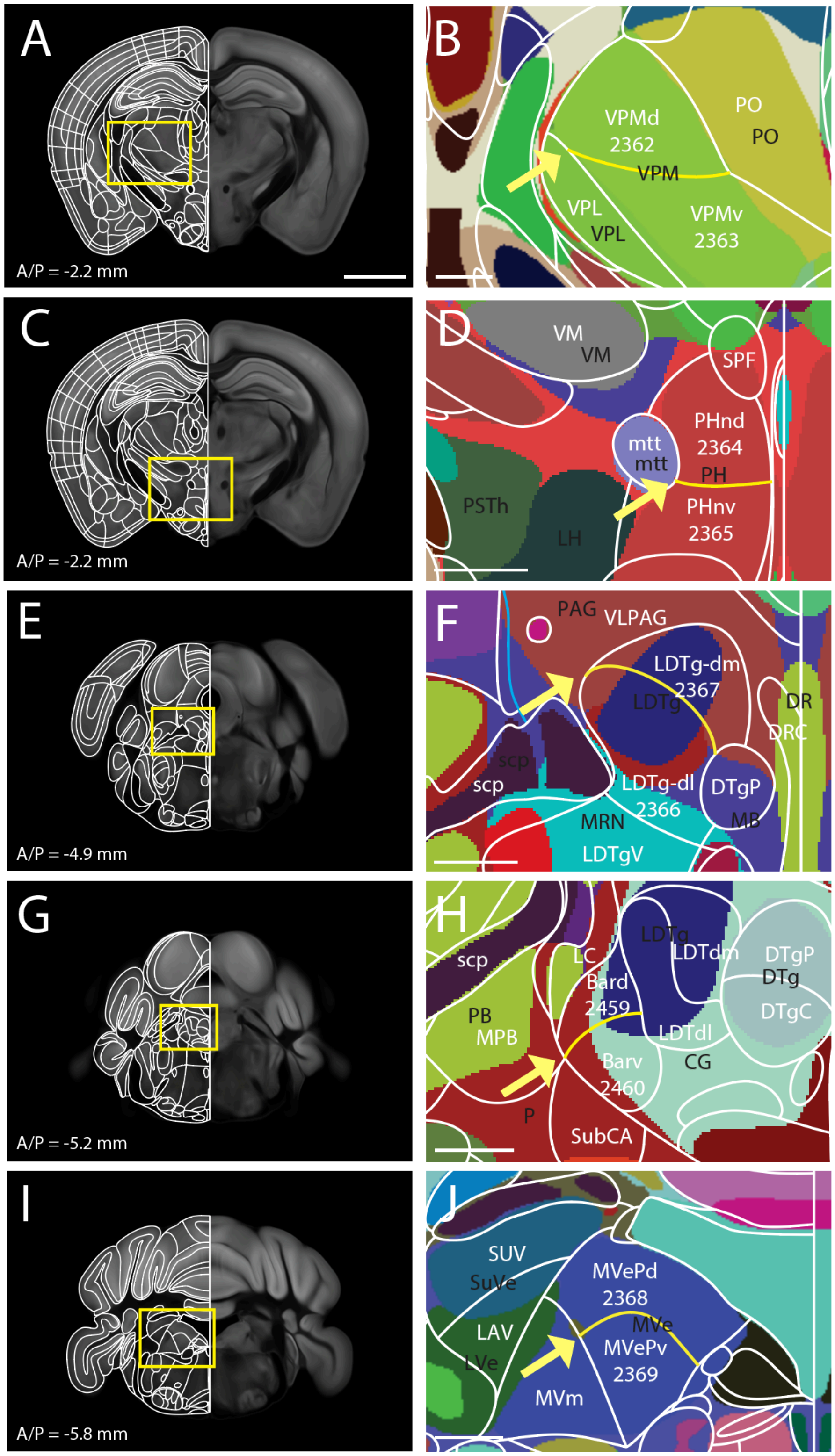

Supplementary Figure 3. Added delineation comparison with CCFv3 labels
First column: Our highly segmented FP based labels on the Allen CCF. Scale bar = 2mm.
Second column: comparison between our labels (white lines) and the CCFv3 labels

(colored background). Scale bar = 300μm. Anatomical names in black and white are from the CCFv3 and our labels, respectively. All structures were further segmented with marker brain neuronal populations as seen in Figure 3. (A-B) Ventral posteromedial nucleus of the thalamus (VPM) further segmented to dorsal and ventral parts (VPMd and VPMv, respectively) (C-D) Posterior hypothalamic nucleus (PH) further segmented to dorsal and ventral parts (PHnd and PHnv, respectively). (E-F) Laterodorsal tegmental nucleus, dorsal part (LDTg) further segmented to lateral and medial divisions (LDTg-dl and LDTg-dm, respectively). (G-H) Barrington nucleus (Bar) further segmented into dorsal and ventral parts (Bard and Barv, respectively). (I-J) Medial vestibular nucleus, parvicellular part (MVp) further segmented into dorsal and ventral parts (MVpd and MVpv, respectively).

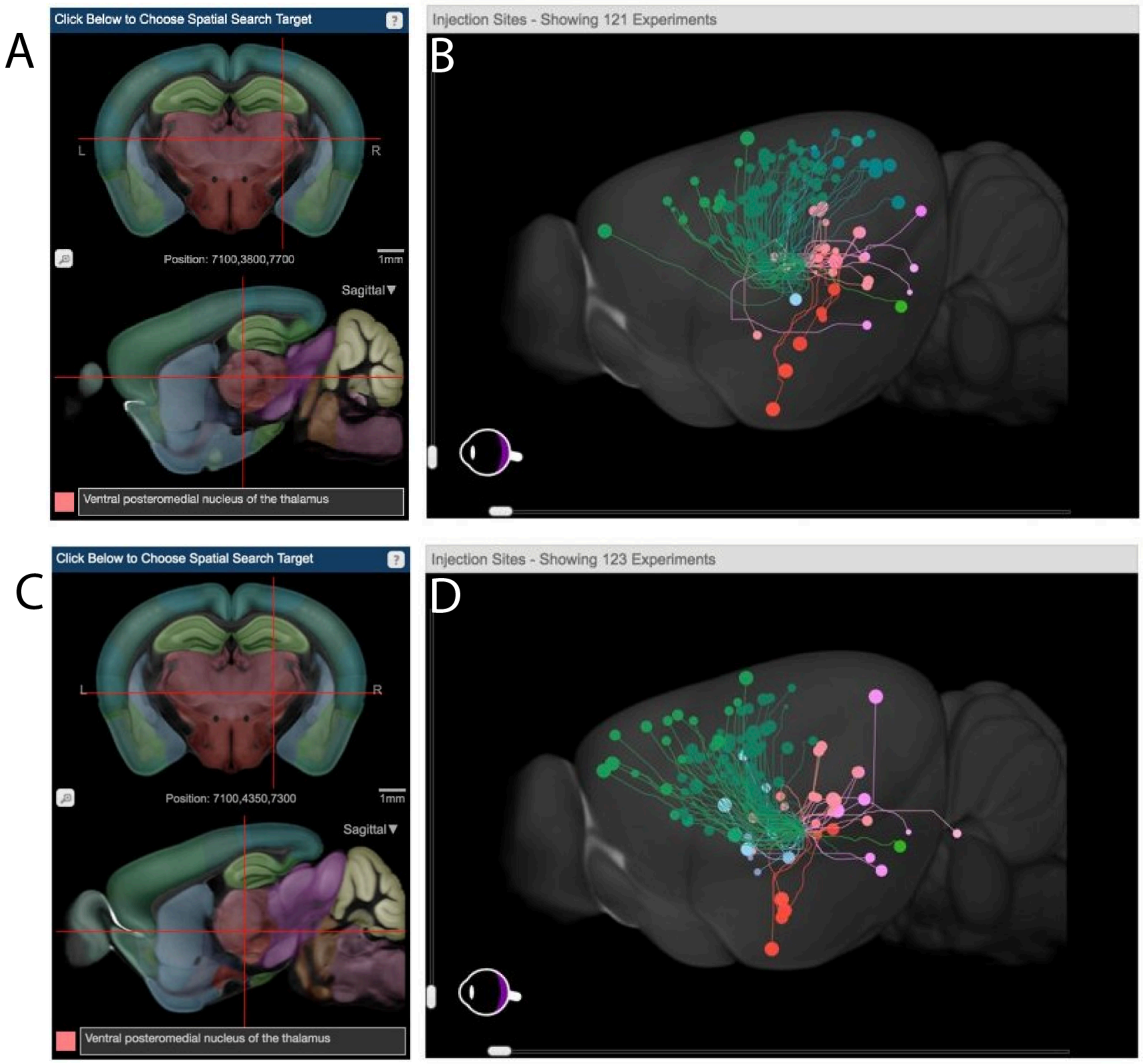

Supplementary Figure 4. Different input to subregions of the ventral posteromedial nucleus of thalamus (VPM)
(A-D) Allen connectivity input pattern to dorsal (A-B) and ventral (C-D) region of the VPM. (A) Data query position for the VPMd, (B) Areas projecting to the VPMd. (C) Data query position for the VPMv, (D) Areas projecting to the VPMd. Note that the VPMd and the VPMv receive input preferentially from posterior and anterior cortical areas, respectively.

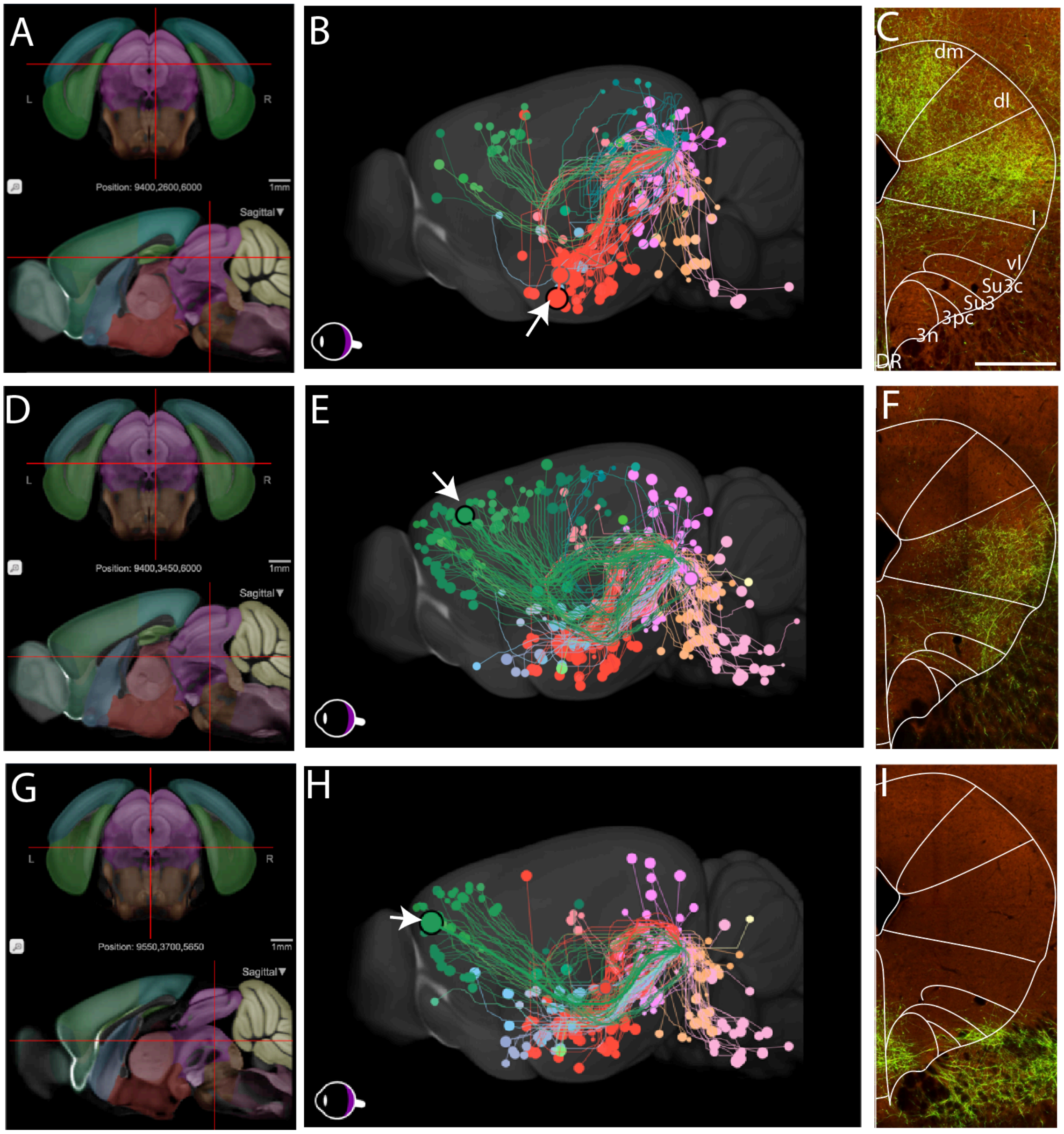

Supplementary Figure 5. Different input to subregions of the periaqueductal grey (PAG)
First column: Spatial query in Allen connectivity database for the dorsal (A), the lateral (D), and the ventral (G) PAG area. Second column: areas projecting to the dorsal (B), the lateral (E), and the ventral (H) PAG area. Third column: Examples of long-range projection from areas highlighted with arrows in the second column, innervating different subregions of the PAG. Scale bar = 200μm.

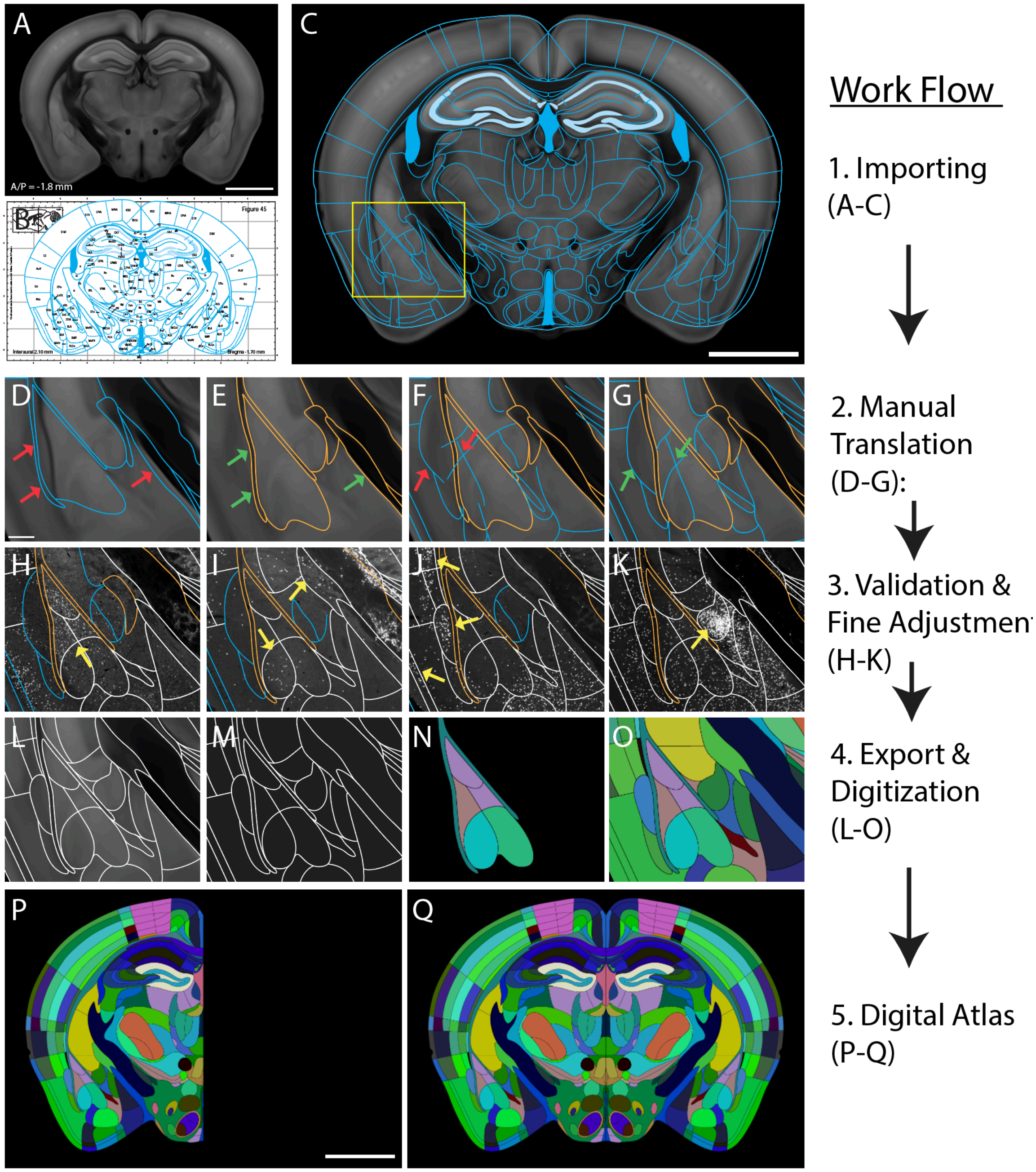

Supplementary Figure 6. Atlasing work flow
(1) Importing: Raw FP labels (B) were imported into the Allen CCF (A) via linear translation (C), Scale bar = 2mm. (2) Manual Translation: Landmark boundaries (D, blue) moved manually (E, orange) based on autofluorescent background. Scale bar = 300µm. Adjacent structures (F, blue) were adjusted (G, blue) based on set landmark structures. (3) Validation and Fine Adjustment: Individual structures adjusted based on several marker brains, including Cux2-Cre:Ai75 (H), PV-Cre:H2B-GFP (I), Ctgf-Cre:Ai75 (J), and SST-Cre:H2B-GFP (K). Orange boundaries denote landmark boundaries, blue boundaries denote adjusted FP labels from step 2, and white labels denote manually adjusted boundaries based on specific neuron populations. (4) Export

and Digitization: Finalized boundaries on Adobe Illustrator (L) exported as tiff (M). Unique numerical IDs were assigned to each structure using FIJI (N) at regional (O) and hemisphere (P) levels. (5) Digital Atlas: Digitized hemisphere (P) was duplicated for whole brain labels (Q) throughout 123 A/P planes. Scale bar = 2mm.

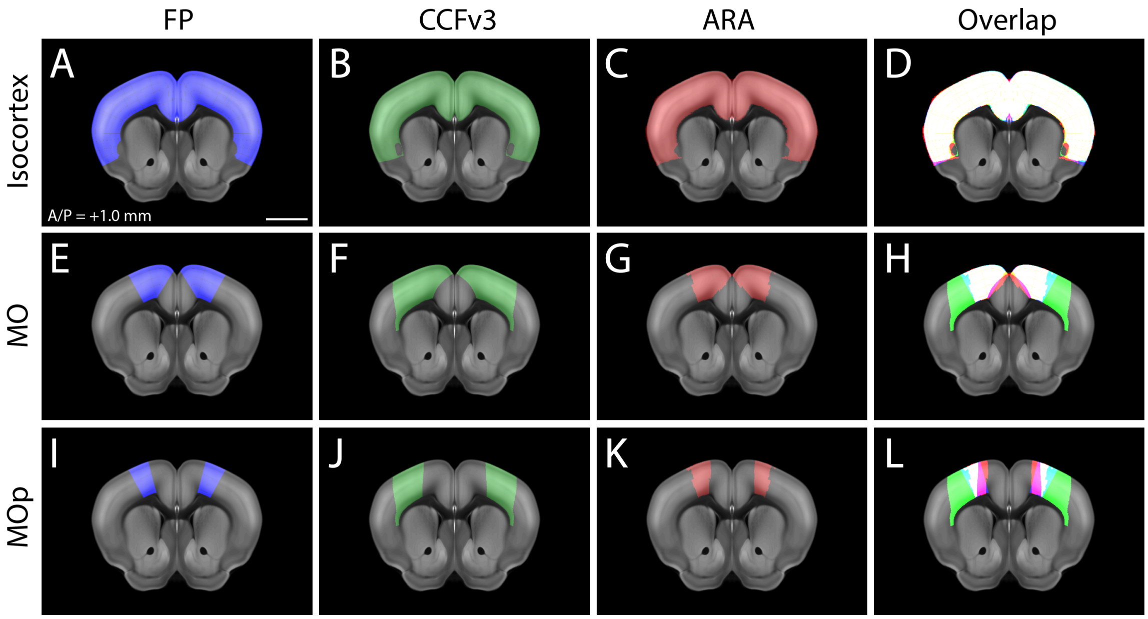

Supplementary Figure 7. Overlap of brain regions across the three different atlases First column: Our segmented FP based labels on the Allen CCF. Second column: CCFv3 labels. Third column: the ARA labels. Fourth column: Overlap between the all three labels. (A-C) Isocortex defined by the three atlases in one framework. Overlapping region of the isocortex in all three labels is highlighted in white (D). (E-H) The motor area (MO) and (I-L) the primary motor area (MOp). Note the discrepancies between the atlases at the MOp level. Scale bar in (A) = 2mm.
