# Supplementary material for: Enhanced and unified anatomical labeling for a common mouse brain atlas
Source: Nat Commun. 2019 Nov 7;10:5067. doi: 10.1038/s41467-019-13057-w (PMC6838086; doi:10.1038/s41467-019-13057-w)
Supplement: Supplementary file 2 — Description of Additional Supplementary Files [file 41467_2019_13057_MOESM2_ESM.doc]

**Description of Additional Supplementary Files**

**Supplementary Data 1: Transgenic mouse list**List of all transgenic mouse brains used for fine label alignments and further segmentations. Column1: Cell type specific Cre drivers, Column2: Cre dependent reporter lines, Column3: Categories for cell type markers, Column4: Data source where high-resolution image data was acquired, Column5: Downloading location if data was from a publically available website, Column6: Specific structures that were highlighted from marker brains. See Supplementary Data 2 for abbreviations.

**Supplementary Data 2: Information on anatomical label identification**
List of the FP based labels’ full names (Column1), abbreviation (Column2), structural ID (Column3), structure order (Column4), parent ID (Column5) for the ontology, Parent abbreviation (Column6), matched CCFv3 full name (Column7), and CCFv3 abbreviation (Column8). Structure order helps to sort areas in ontological hierarchy. Parent ID and abbreviation specify immediately higher order structure. Lack of clearly matched areas between our FP based labels and the CCFv3 labels were left blank. Segmented structures were highlighted as yellow.

**Supplementary Data 3: Overlap between corresponding labels from different atlases**
Dice Similarity Coefficient (DSC) of matched anatomical regions between any given two atlases. DSC = 1 for the perfect overlap, DSC = 0 for no overlap. Yongsoo Kim, Ph.D. Assistant Professor Department of Neural and Behavioral Sciences College of Medicine Penn State University Email: yuk17@psu.edu http://kimlab.io The first tab: Comparison between our FP based labels and the CCFv3, The second tab: Comparison between our FP based labels and the ARA. Brain regions were arranged based on the Allen ontology.

**Supplementary Data 4: A series of digitized labels in every 100 µm z spacing**
The file name contains the information about Bregma anterior posterior (AP) coordinate of each image. For example, “78_AP-3.4” means 78th coronal plane located at AP:-3.4mm. The file contains digitized labels with distinct numerical values (structural ID) that can be found in Supplementary Data 2. We recommend to use FIJI (or ImageJ) to open images. When mouse pointer is placed on labels, FIJI can display label value. Web visualization of these labels with anatomical annotation can be found in <http://kimlab.io/brain-map/atlas/>

**Supplementary Data 5: A series of matched CCF background coronal images to Supplementary File 1**
Z numbers in the file name are matched with label numbers in Supplementary Data 4. For example, “AllenCCF_Z078” is the CCF background for the label 78_AP-3.4. Pixel resolution = 10 µm.

**Supplementary Data 6: A series of vector drawing files in every 100 µm z spacing**
Each Illustrator file contains vector drawing over a corresponding CCF background image. Anatomical label names can be found in <http://kimlab.io/brain-map/atlas/>

**Supplementary Data 7: Image registration parameter files for Elastix**
Par_affine.txt and Par_bspline.txt are parameter files for our linear and non-linear image registration using Elastix, respectively.
