# Supplementary figures and images for: Enhanced and unified anatomical labeling for a common mouse brain atlas (part 2 of 3)
Source: Nat Commun. 2019 Nov 7;10:5067. doi: 10.1038/s41467-019-13057-w (PMC6838086; doi:10.1038/s41467-019-13057-w)

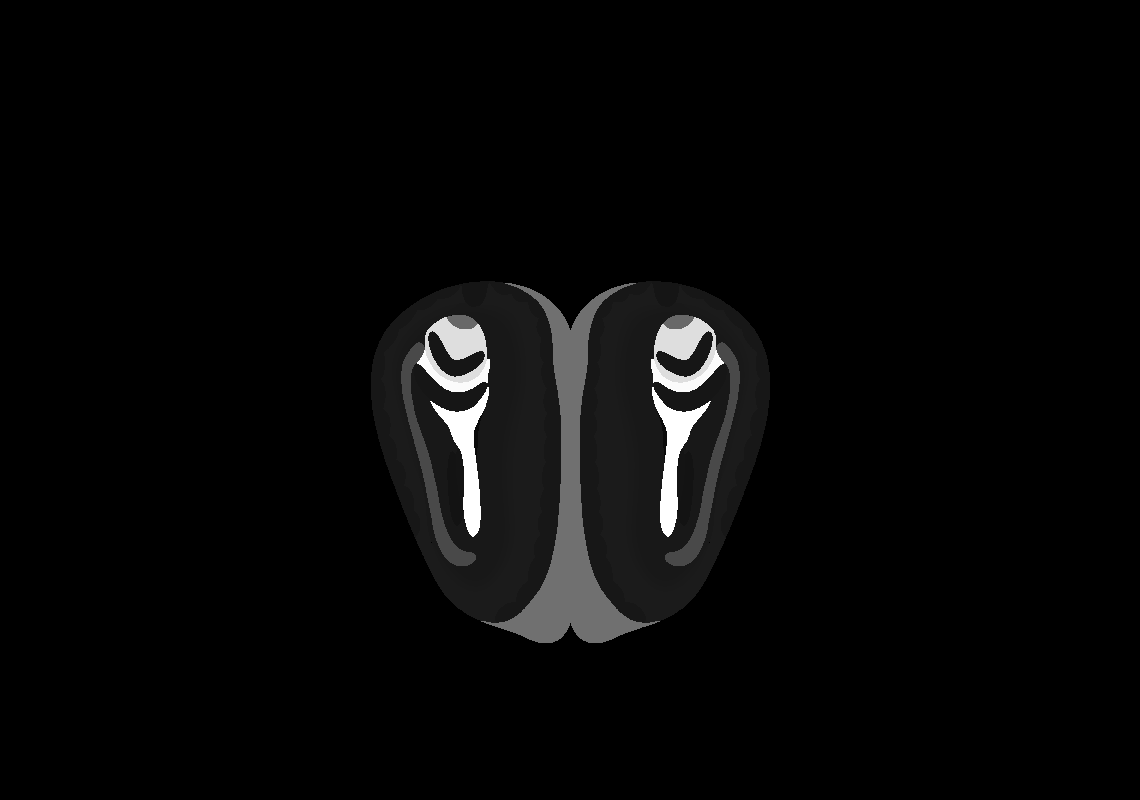

Supplement: Supplementary file 6 — Supplementary Data 4 [file 41467_2019_13057_MOESM6_ESM.zip › Suppl_File1_Labels/10_AP+3.4.tif]

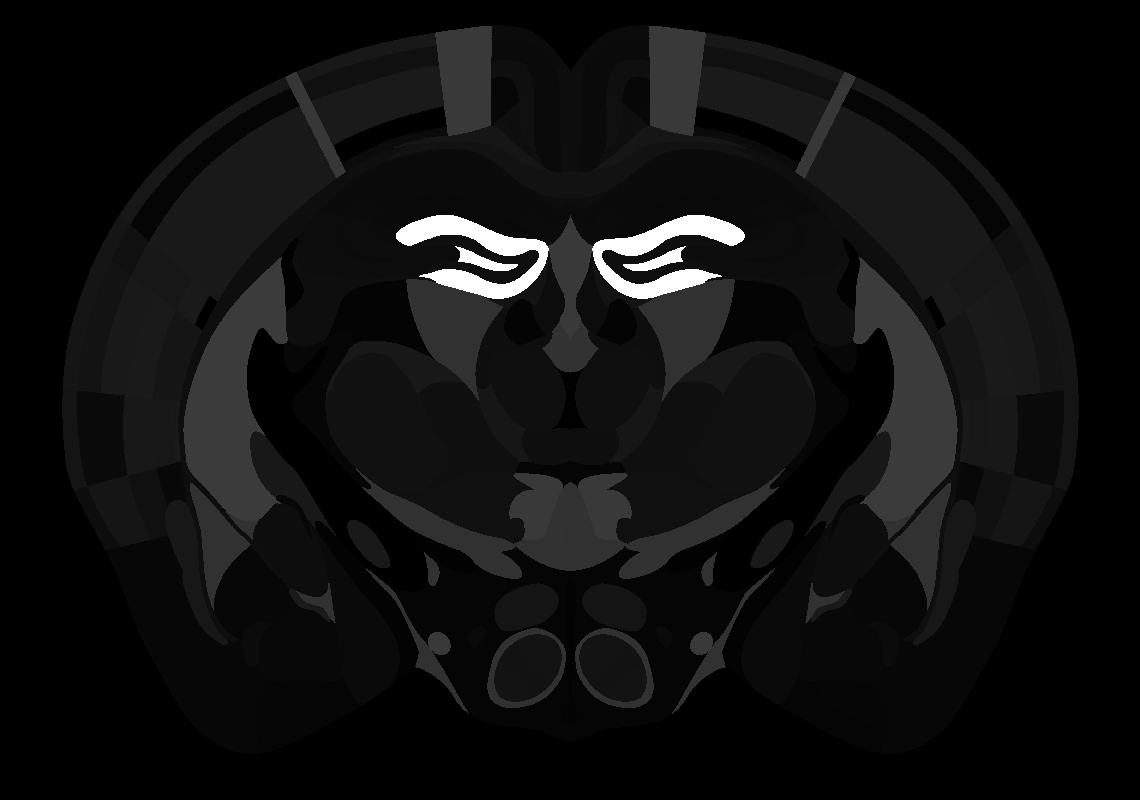

Supplement: Supplementary file 6 — Supplementary Data 4 [file 41467_2019_13057_MOESM6_ESM.zip › Suppl_File1_Labels/61_AP-1.7.tif]

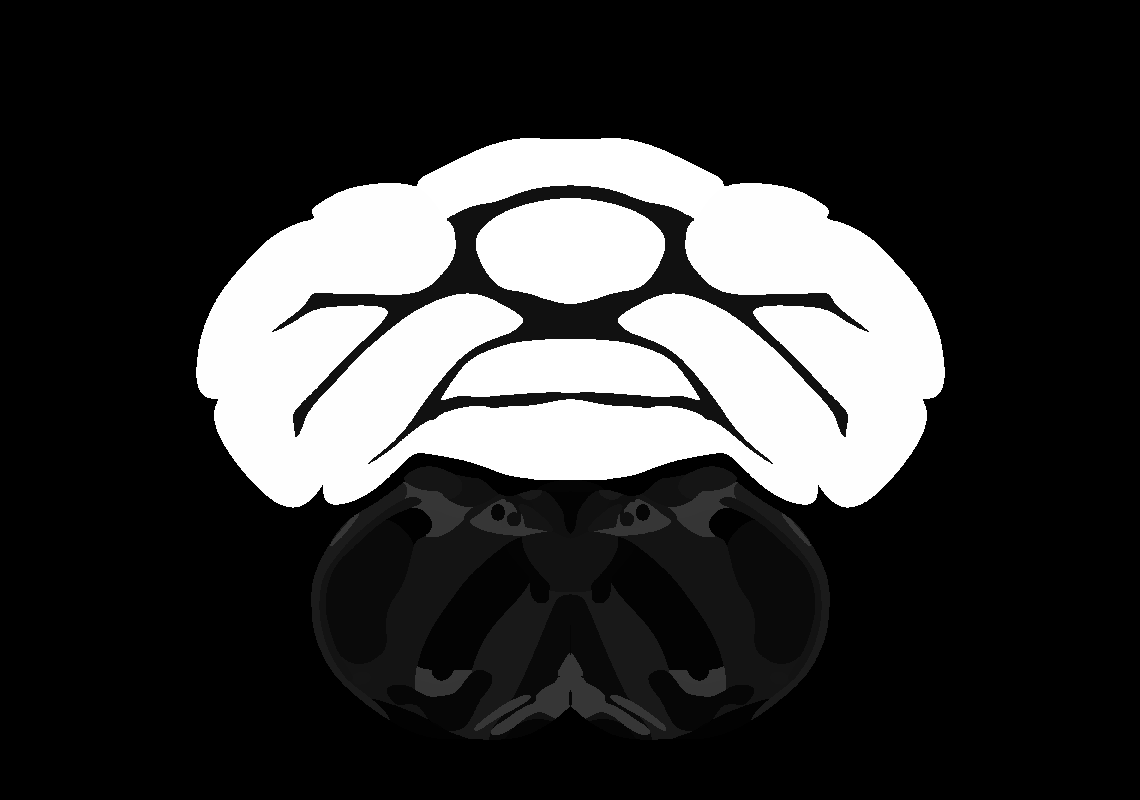

Supplement: Supplementary file 6 — Supplementary Data 4 [file 41467_2019_13057_MOESM6_ESM.zip › Suppl_File1_Labels/116_AP-7.2.tif]

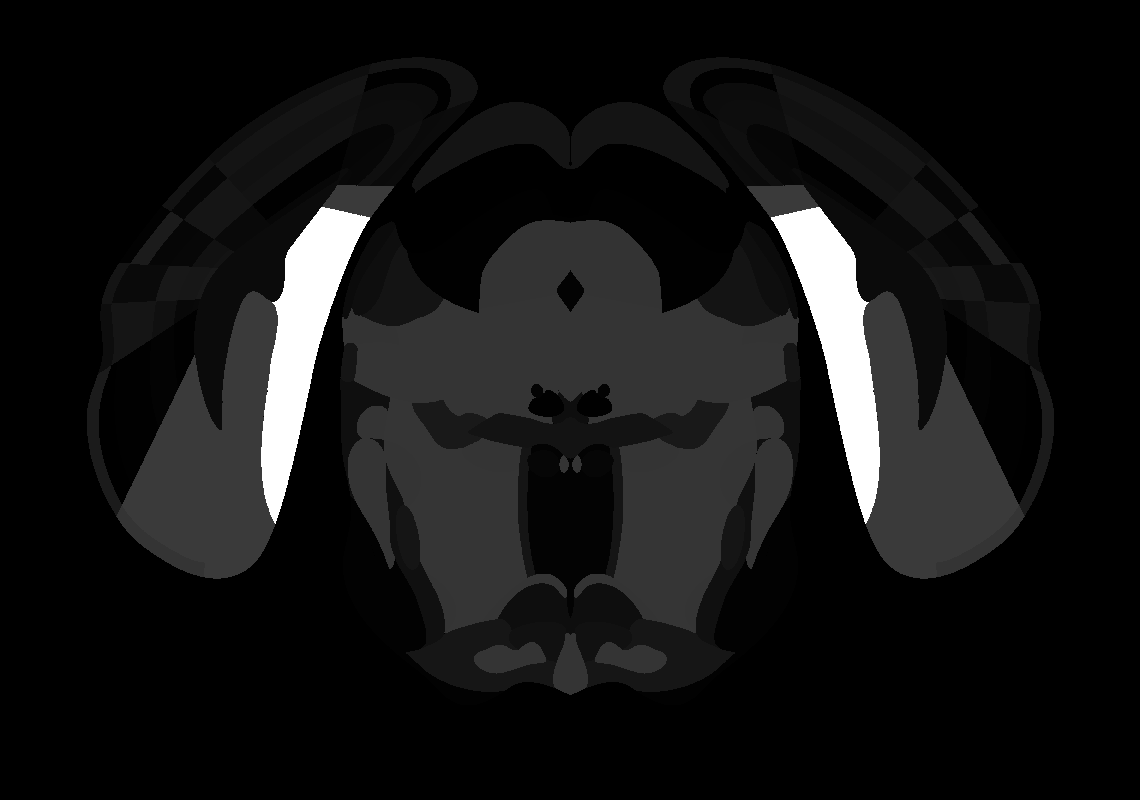

Supplement: Supplementary file 6 — Supplementary Data 4 [file 41467_2019_13057_MOESM6_ESM.zip › Suppl_File1_Labels/88_AP-4.4.tif]

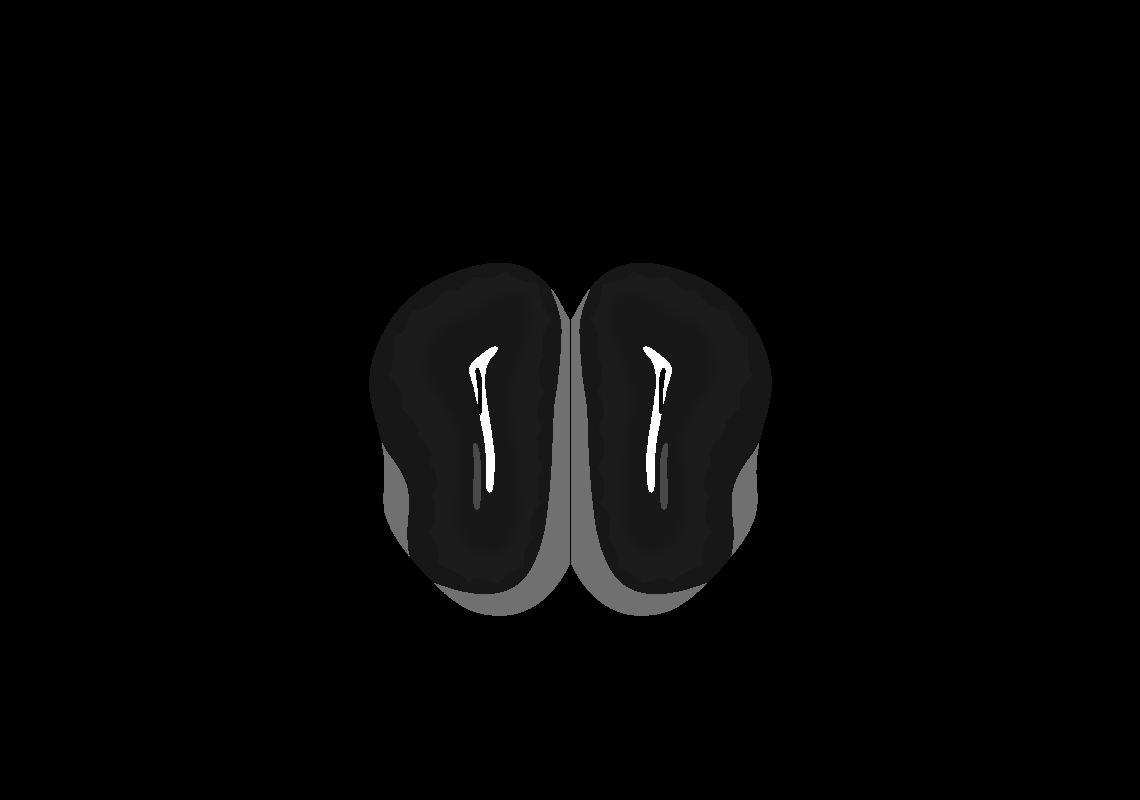

Supplement: Supplementary file 6 — Supplementary Data 4 [file 41467_2019_13057_MOESM6_ESM.zip › Suppl_File1_Labels/1_AP+4.3.tif]

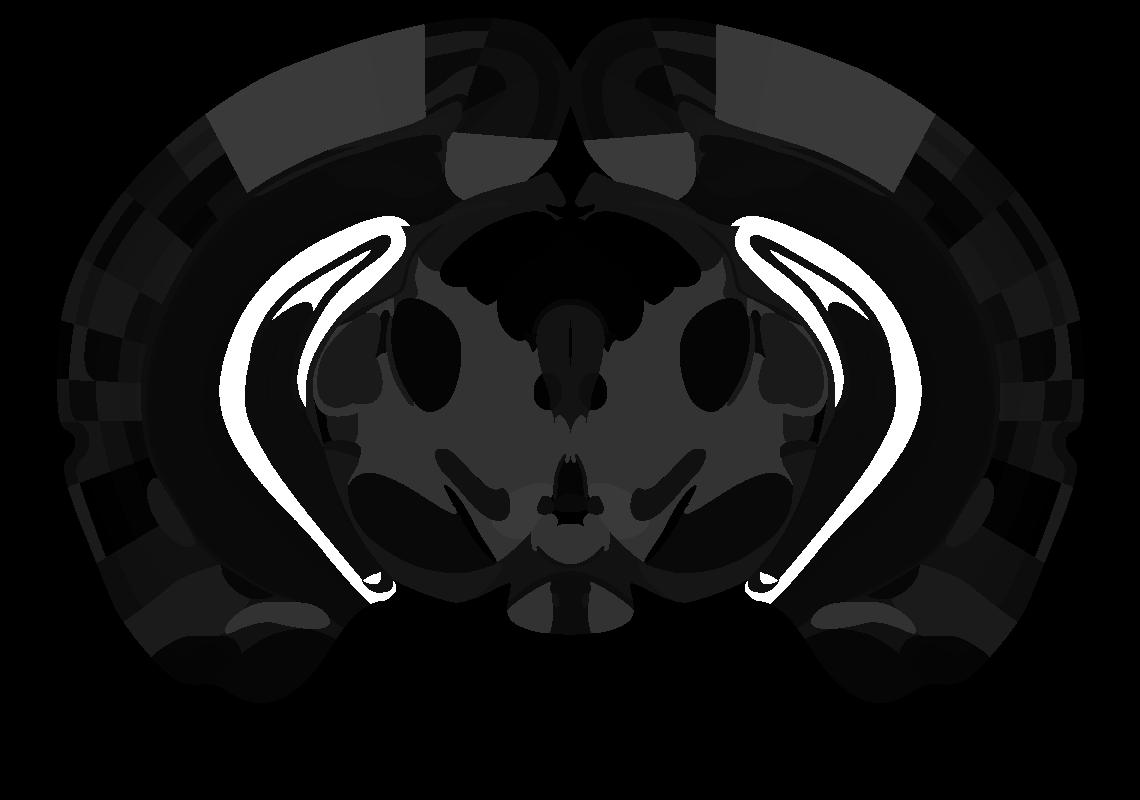

Supplement: Supplementary file 6 — Supplementary Data 4 [file 41467_2019_13057_MOESM6_ESM.zip › Suppl_File1_Labels/75_AP-3.1.tif]

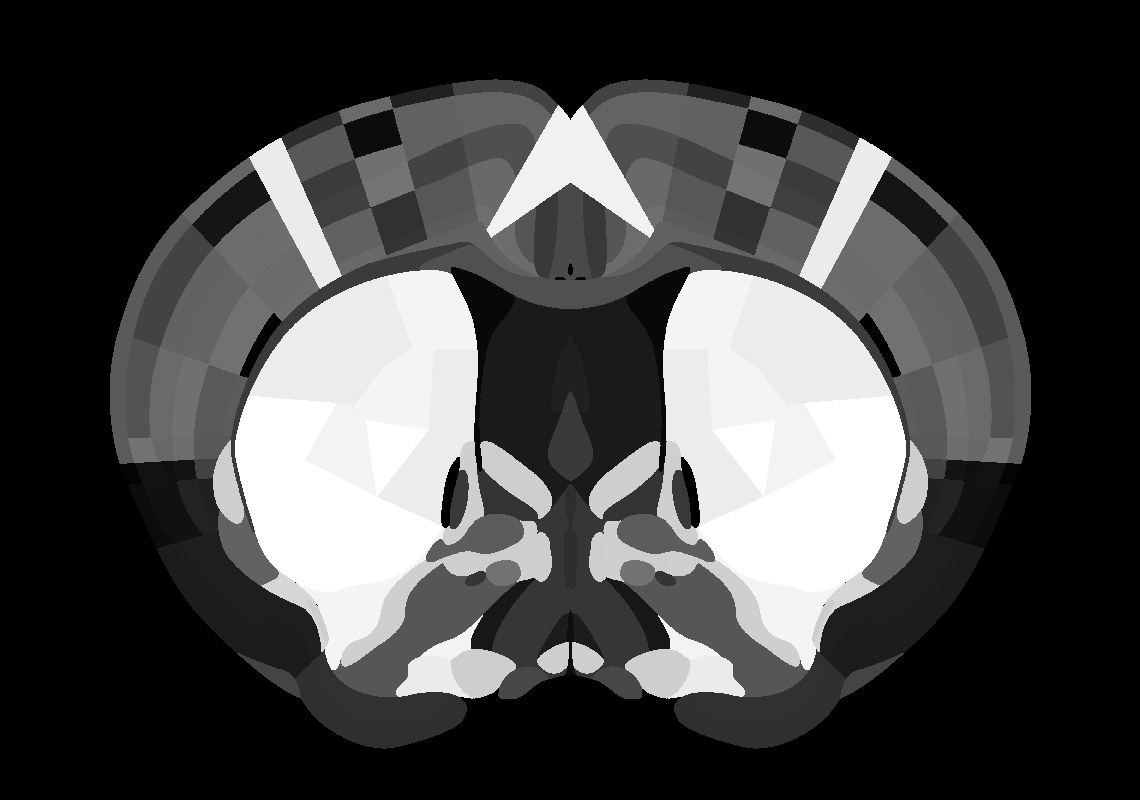

Supplement: Supplementary file 6 — Supplementary Data 4 [file 41467_2019_13057_MOESM6_ESM.zip › Suppl_File1_Labels/42_AP+0.2.tif]

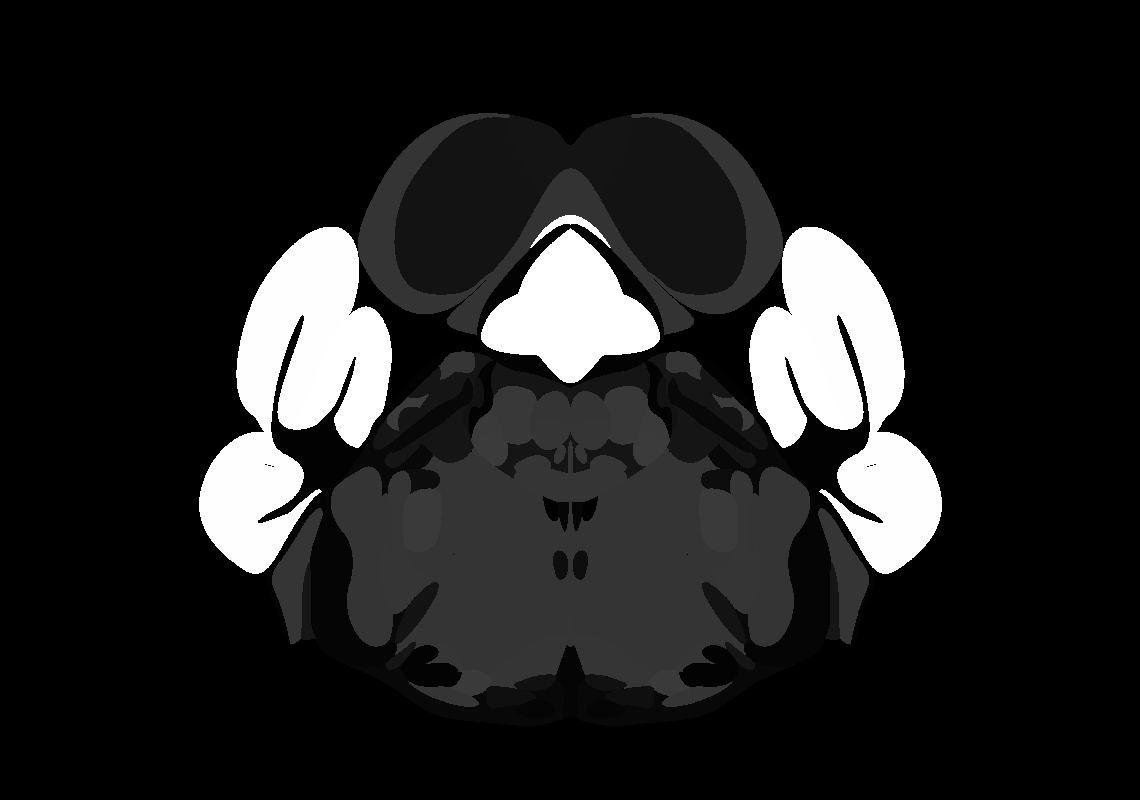

Supplement: Supplementary file 6 — Supplementary Data 4 [file 41467_2019_13057_MOESM6_ESM.zip › Suppl_File1_Labels/96_AP-5.2.tif]

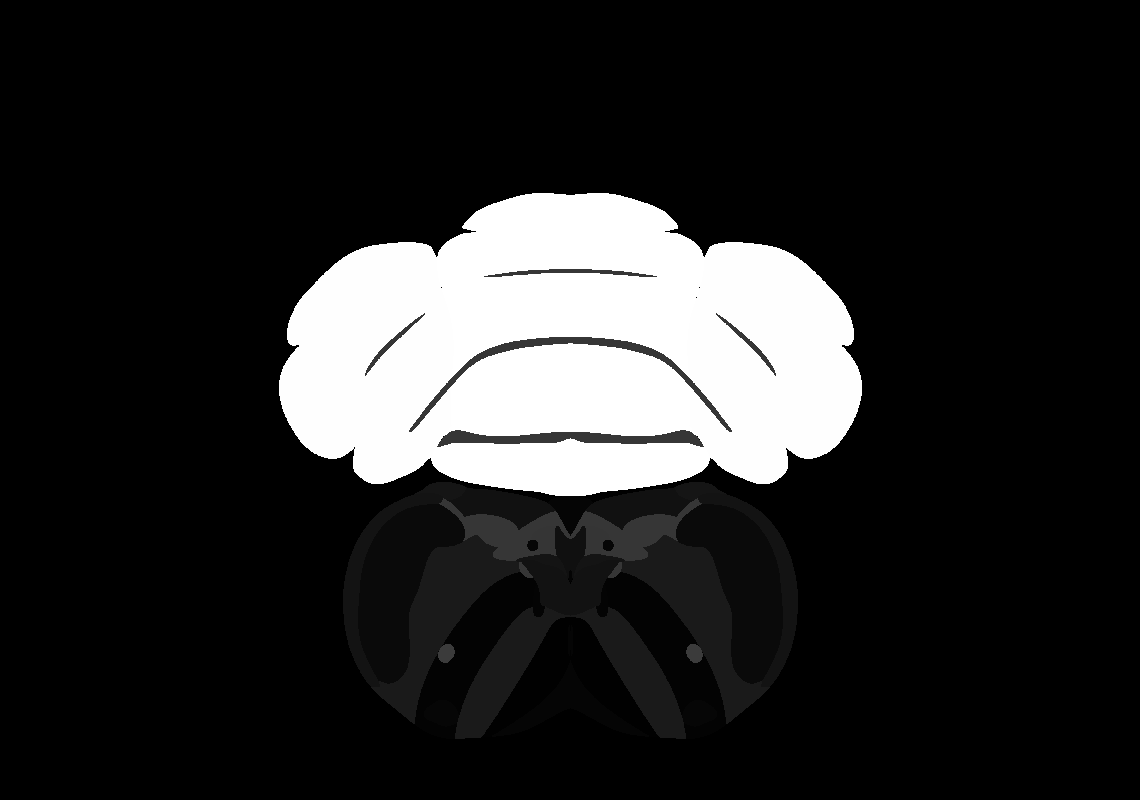

Supplement: Supplementary file 6 — Supplementary Data 4 [file 41467_2019_13057_MOESM6_ESM.zip › Suppl_File1_Labels/121_AP-7.7.tif]

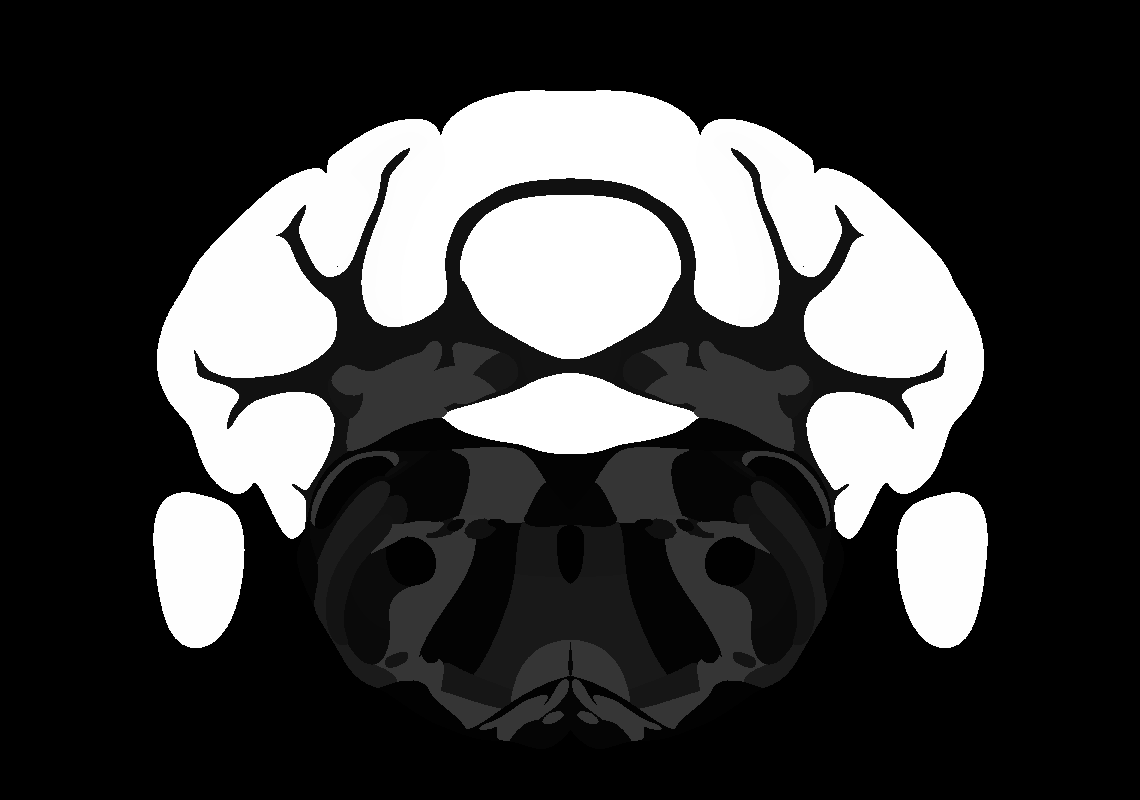

Supplement: Supplementary file 6 — Supplementary Data 4 [file 41467_2019_13057_MOESM6_ESM.zip › Suppl_File1_Labels/108_AP-6.4.tif]

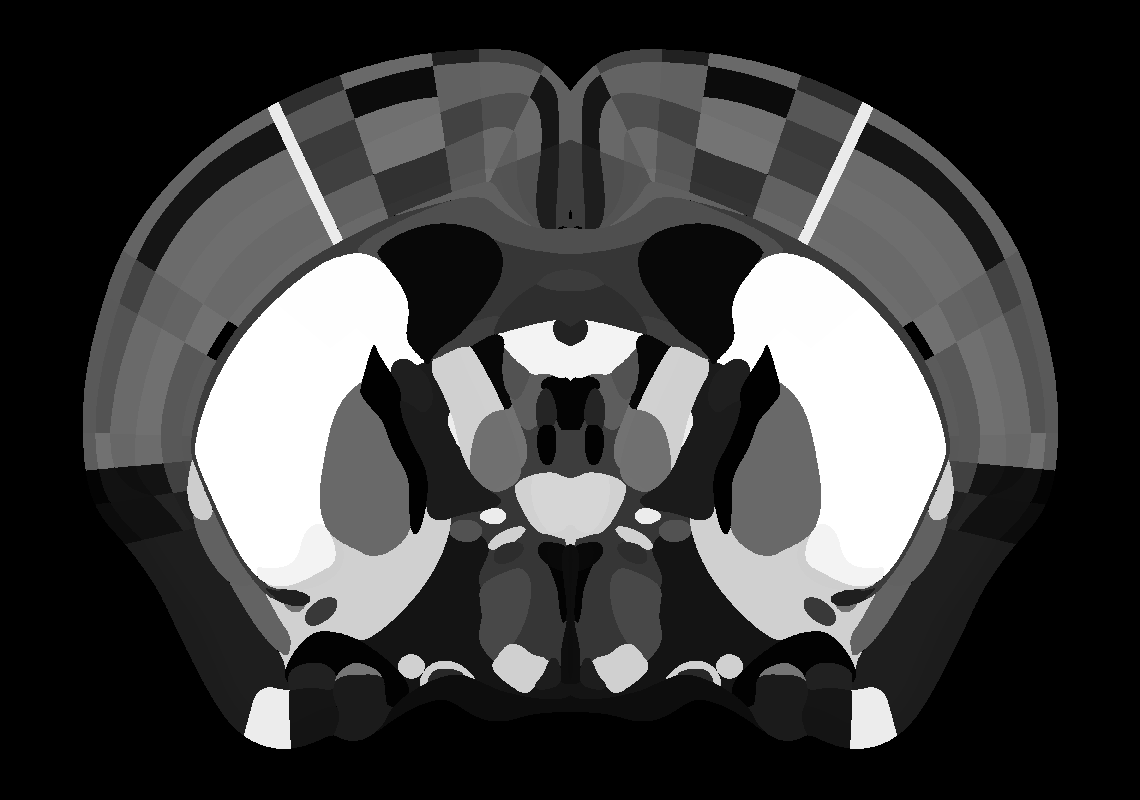

Supplement: Supplementary file 6 — Supplementary Data 4 [file 41467_2019_13057_MOESM6_ESM.zip › Suppl_File1_Labels/51_AP-0.7.tif]

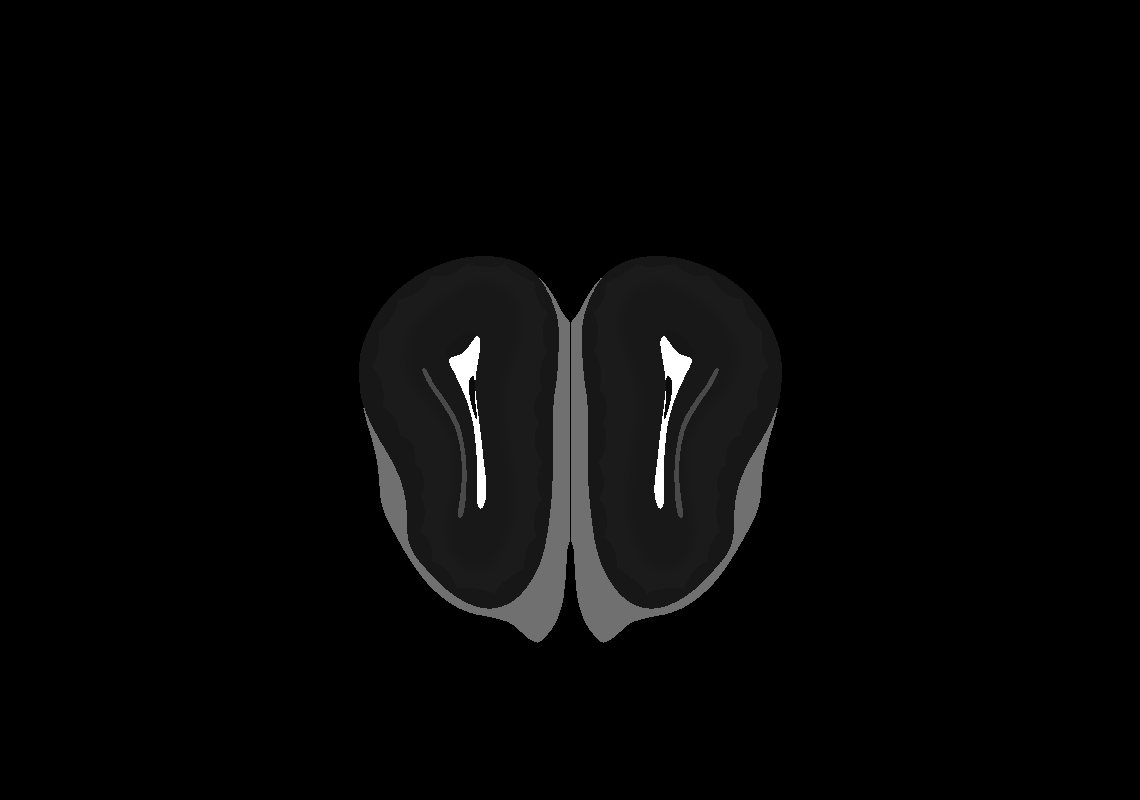

Supplement: Supplementary file 6 — Supplementary Data 4 [file 41467_2019_13057_MOESM6_ESM.zip › Suppl_File1_Labels/5_AP+3.9.tif]

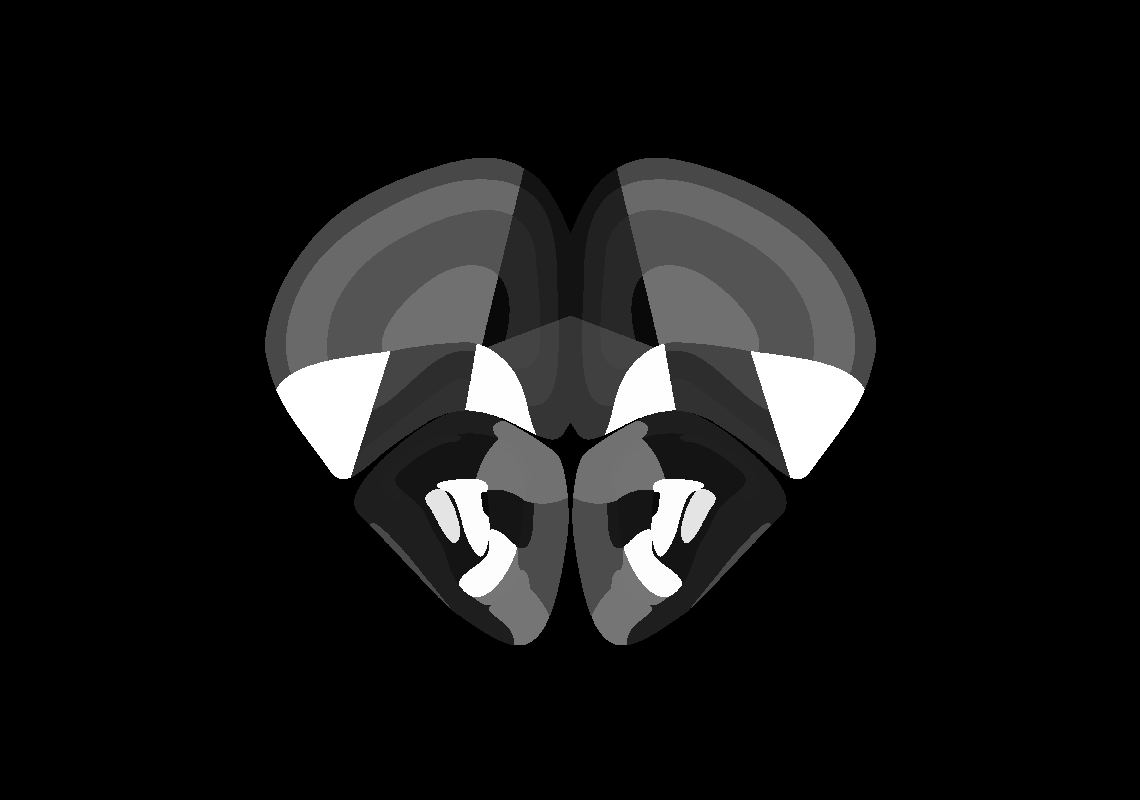

Supplement: Supplementary file 6 — Supplementary Data 4 [file 41467_2019_13057_MOESM6_ESM.zip › Suppl_File1_Labels/20_AP+2.4.tif]

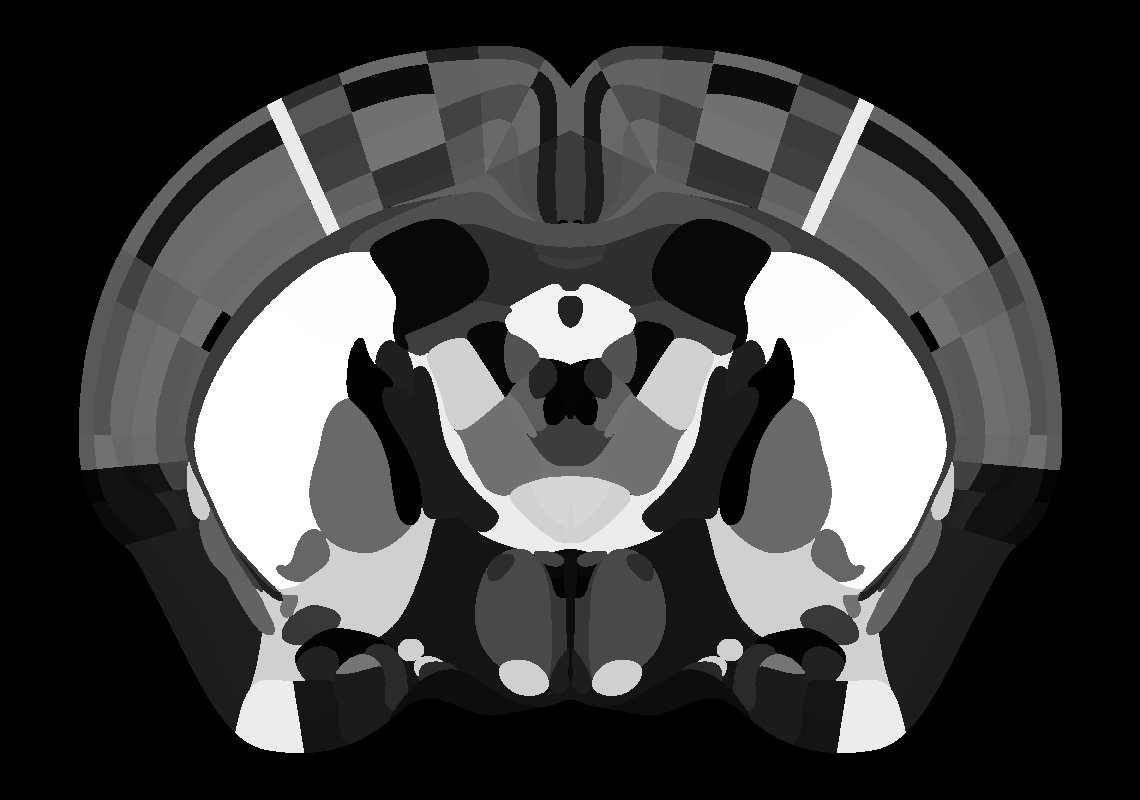

Supplement: Supplementary file 6 — Supplementary Data 4 [file 41467_2019_13057_MOESM6_ESM.zip › Suppl_File1_Labels/52_AP-0.8.tif]

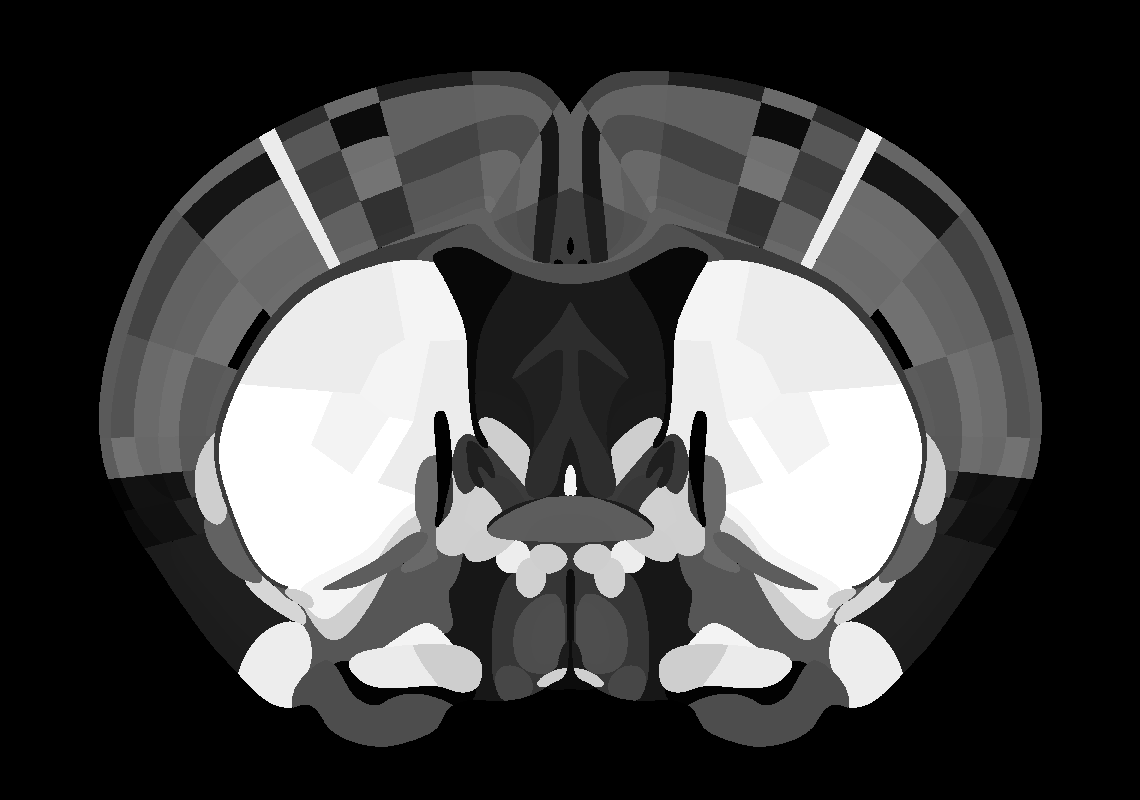

Supplement: Supplementary file 6 — Supplementary Data 4 [file 41467_2019_13057_MOESM6_ESM.zip › Suppl_File1_Labels/45_AP-0.1.tif]

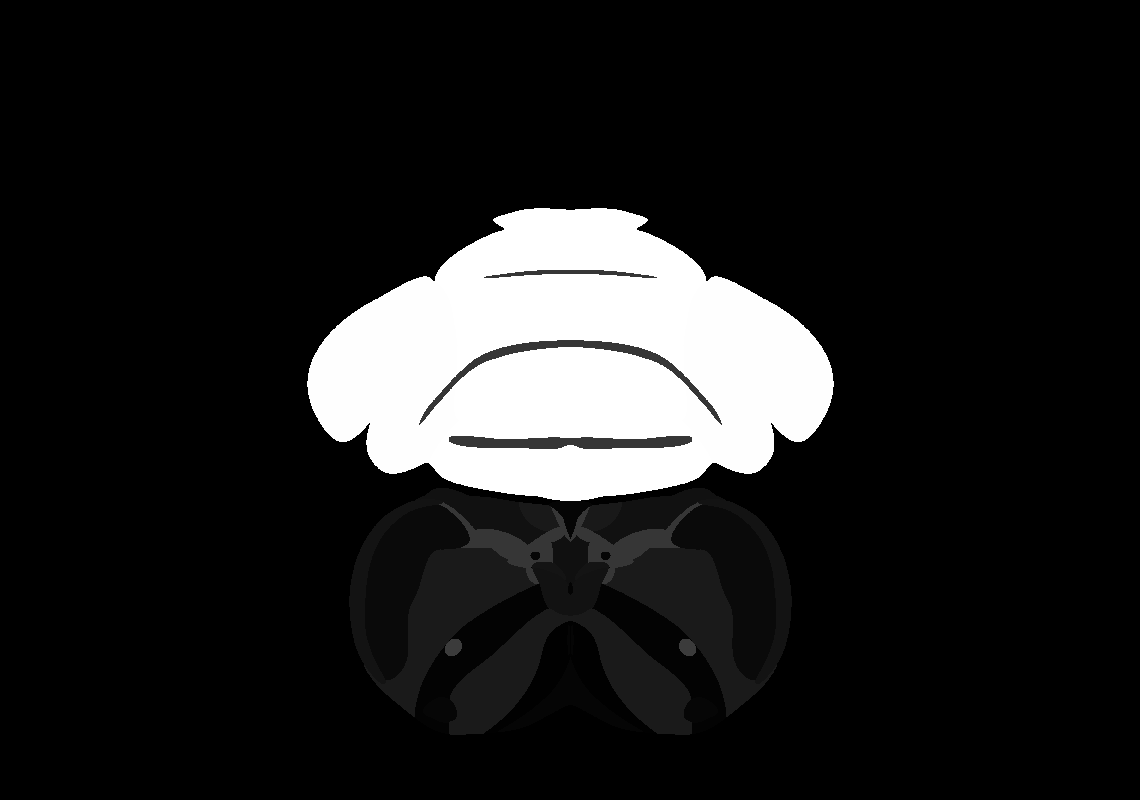

Supplement: Supplementary file 6 — Supplementary Data 4 [file 41467_2019_13057_MOESM6_ESM.zip › Suppl_File1_Labels/122_AP-7.8.tif]

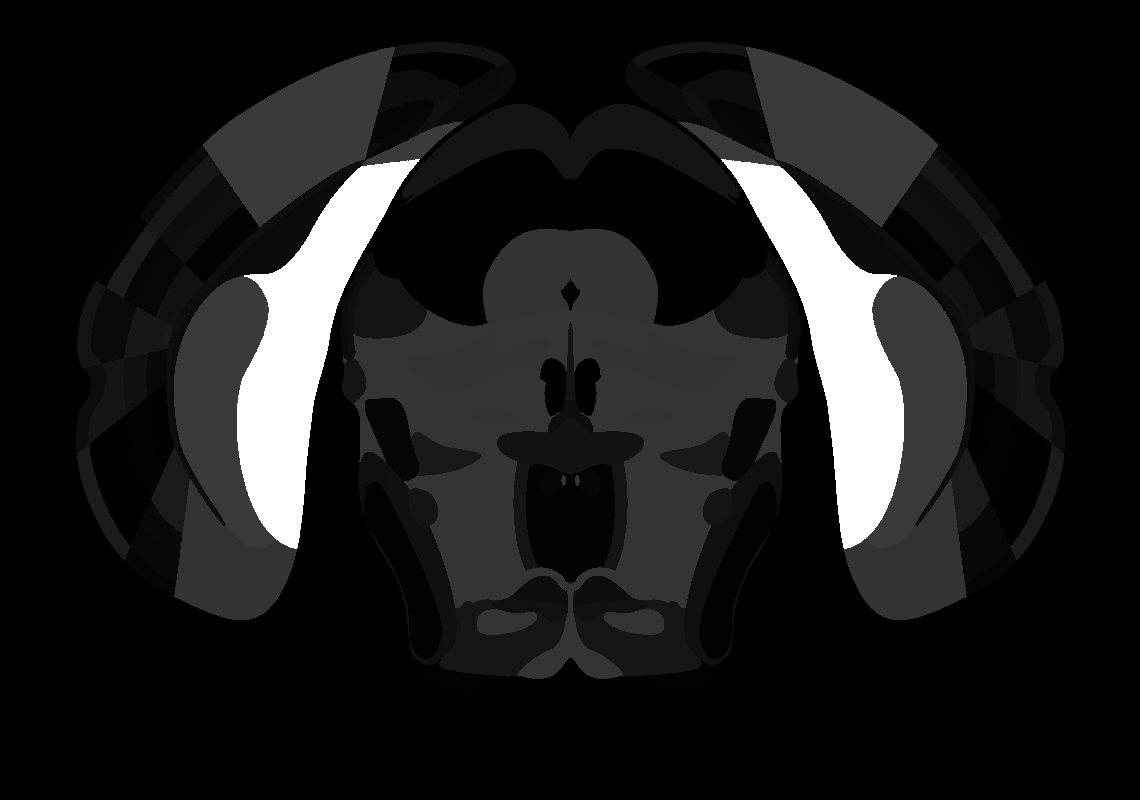

Supplement: Supplementary file 6 — Supplementary Data 4 [file 41467_2019_13057_MOESM6_ESM.zip › Suppl_File1_Labels/85_AP-4.1.tif]

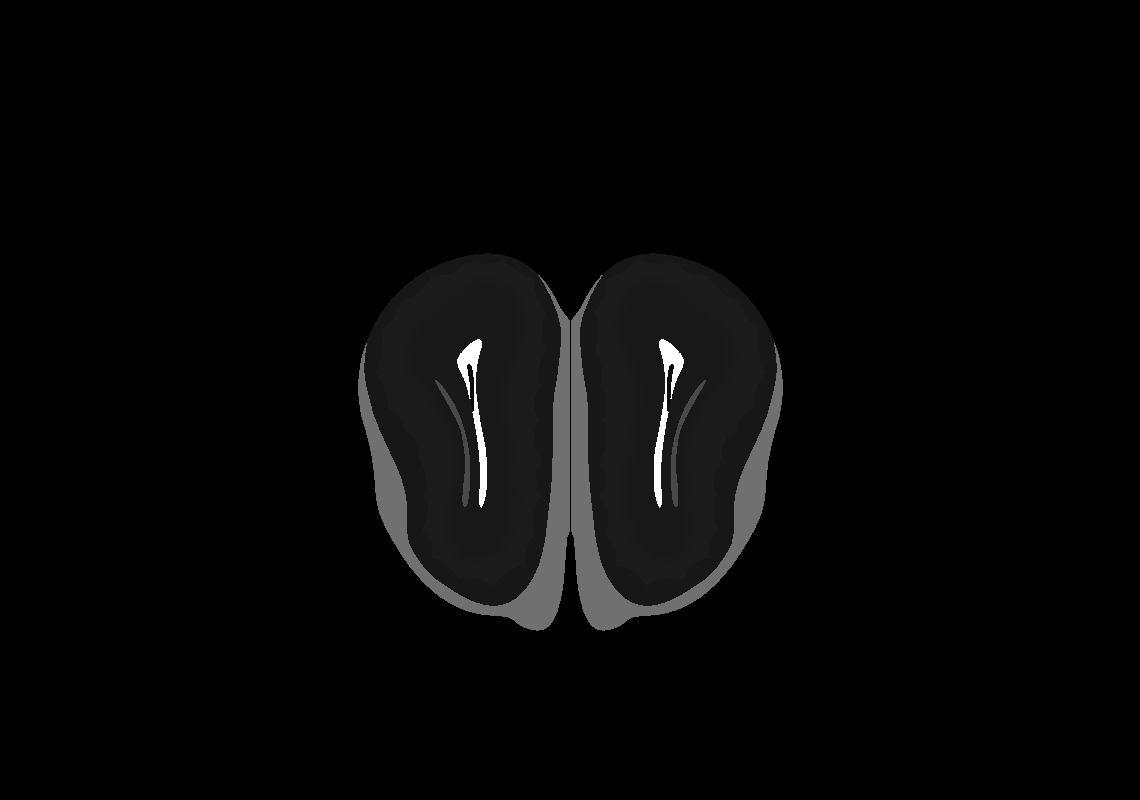

Supplement: Supplementary file 6 — Supplementary Data 4 [file 41467_2019_13057_MOESM6_ESM.zip › Suppl_File1_Labels/4_AP+4.0.tif]

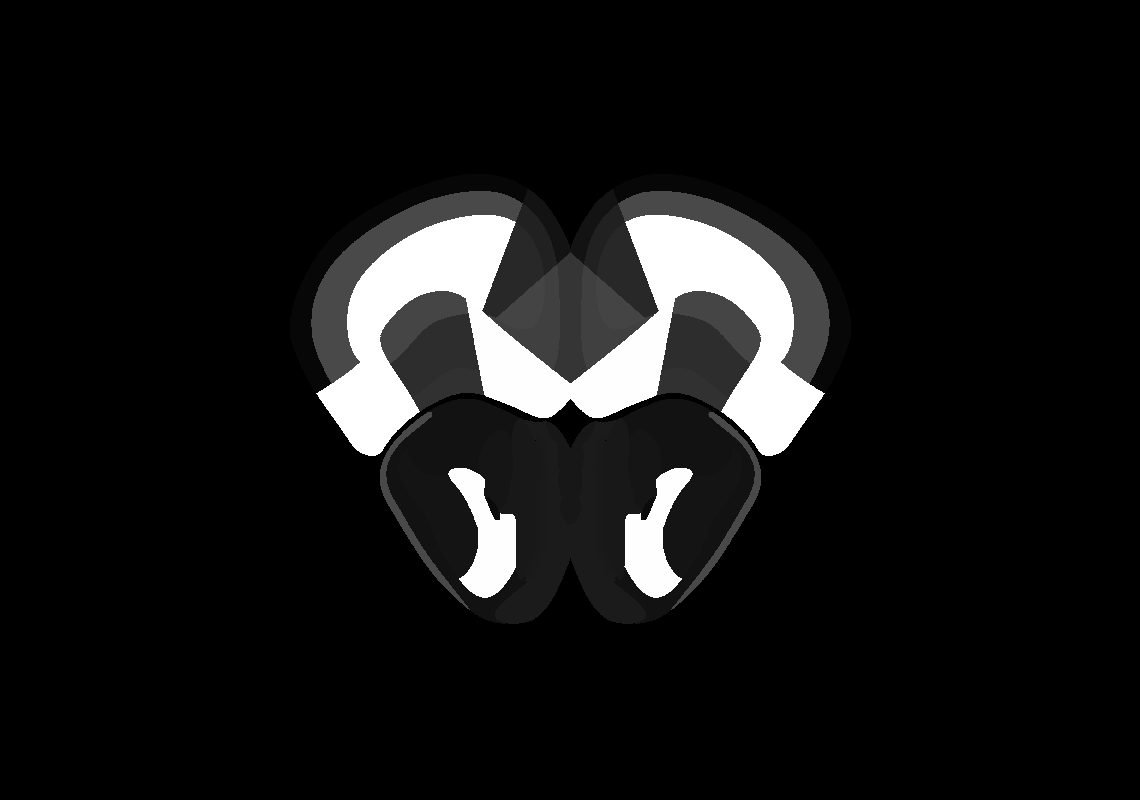

Supplement: Supplementary file 6 — Supplementary Data 4 [file 41467_2019_13057_MOESM6_ESM.zip › Suppl_File1_Labels/17_AP+2.7.tif]

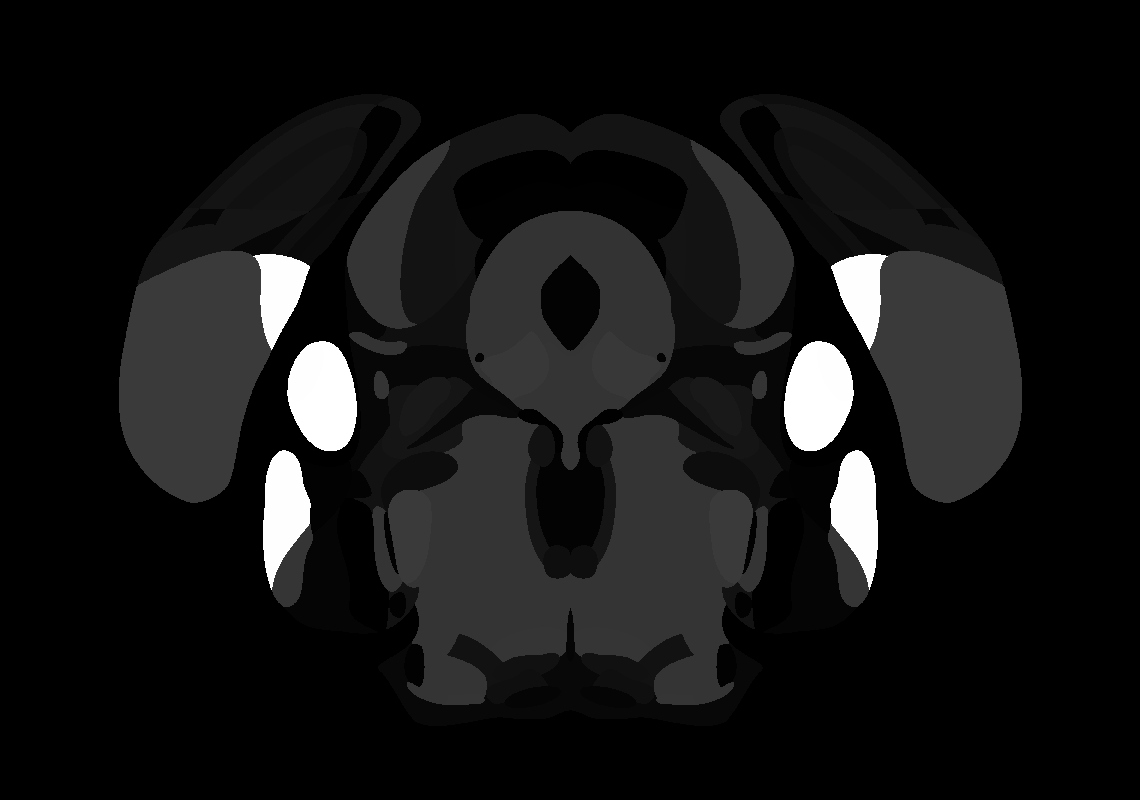

Supplement: Supplementary file 6 — Supplementary Data 4 [file 41467_2019_13057_MOESM6_ESM.zip › Suppl_File1_Labels/92_AP-4.8.tif]

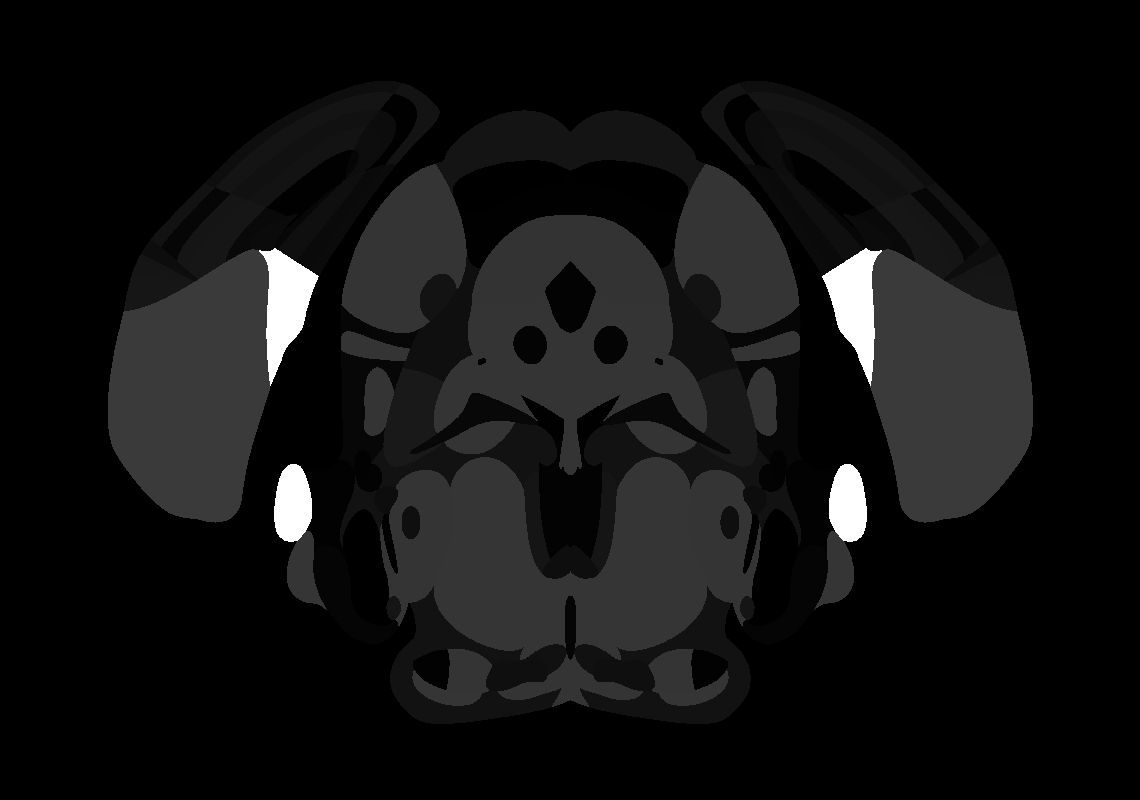

Supplement: Supplementary file 6 — Supplementary Data 4 [file 41467_2019_13057_MOESM6_ESM.zip › Suppl_File1_Labels/91_AP-4.7.tif]

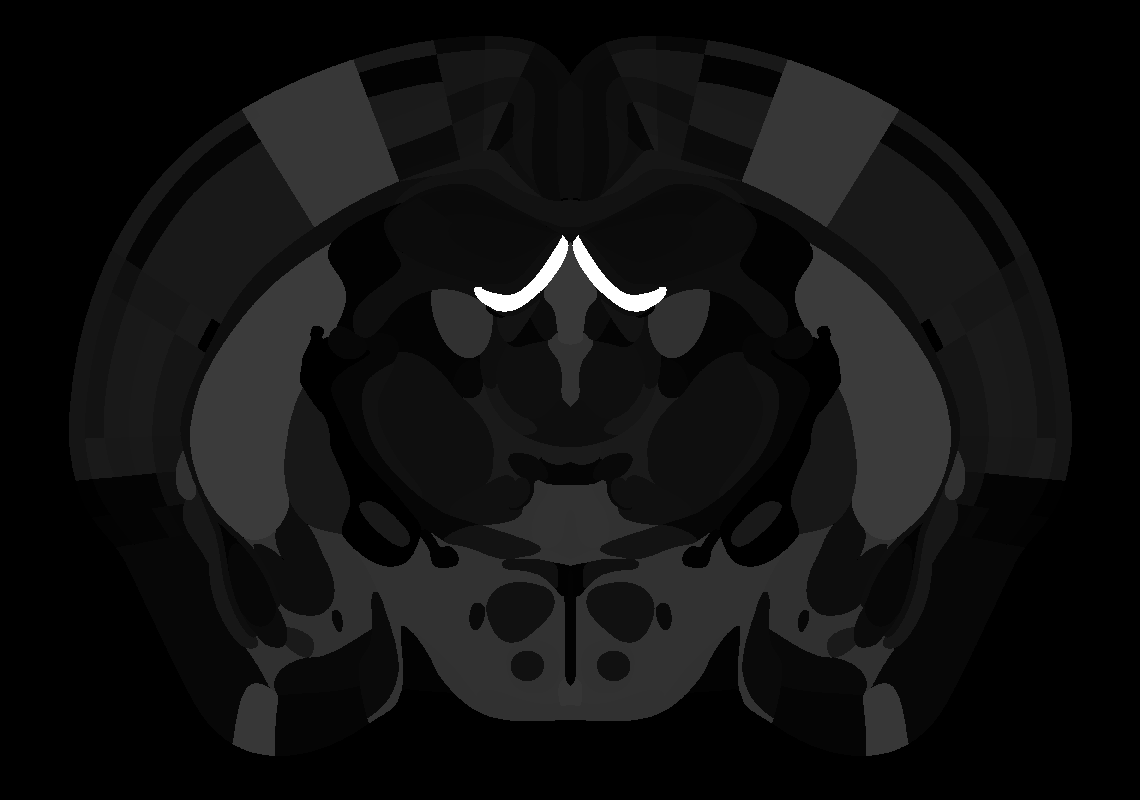

Supplement: Supplementary file 6 — Supplementary Data 4 [file 41467_2019_13057_MOESM6_ESM.zip › Suppl_File1_Labels/56_AP-1.2.tif]

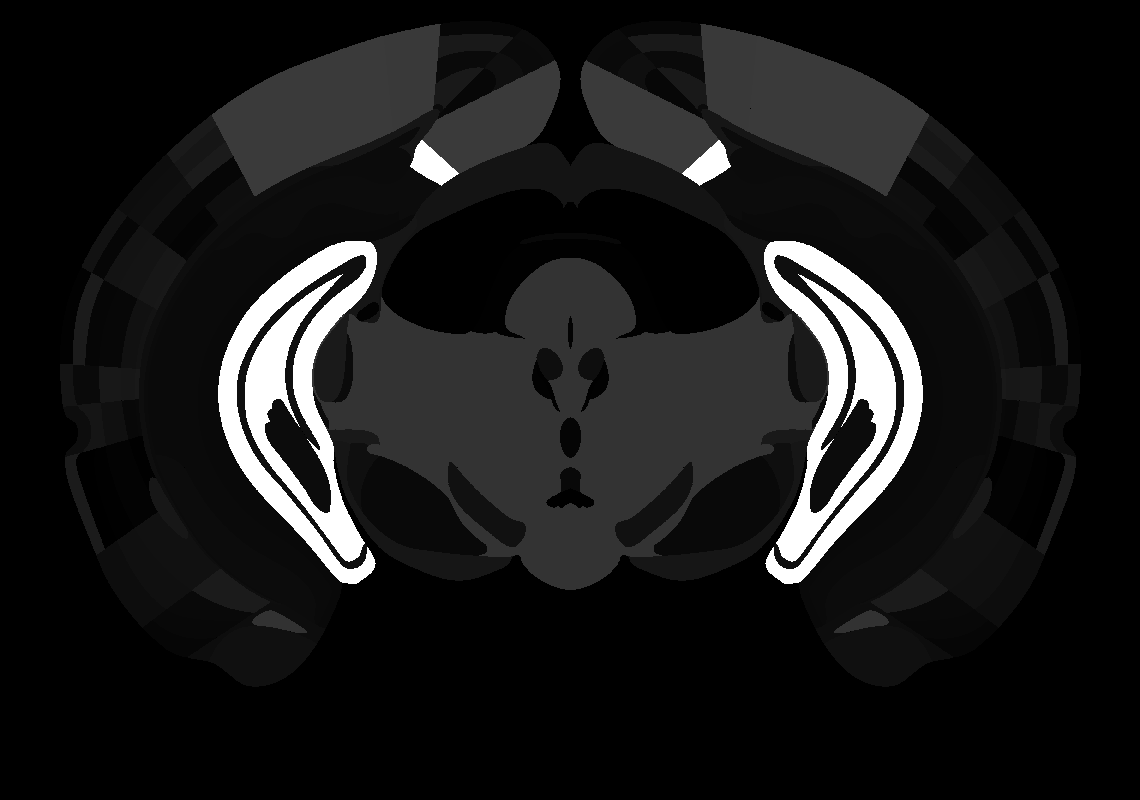

Supplement: Supplementary file 6 — Supplementary Data 4 [file 41467_2019_13057_MOESM6_ESM.zip › Suppl_File1_Labels/78_AP-3.4.tif]

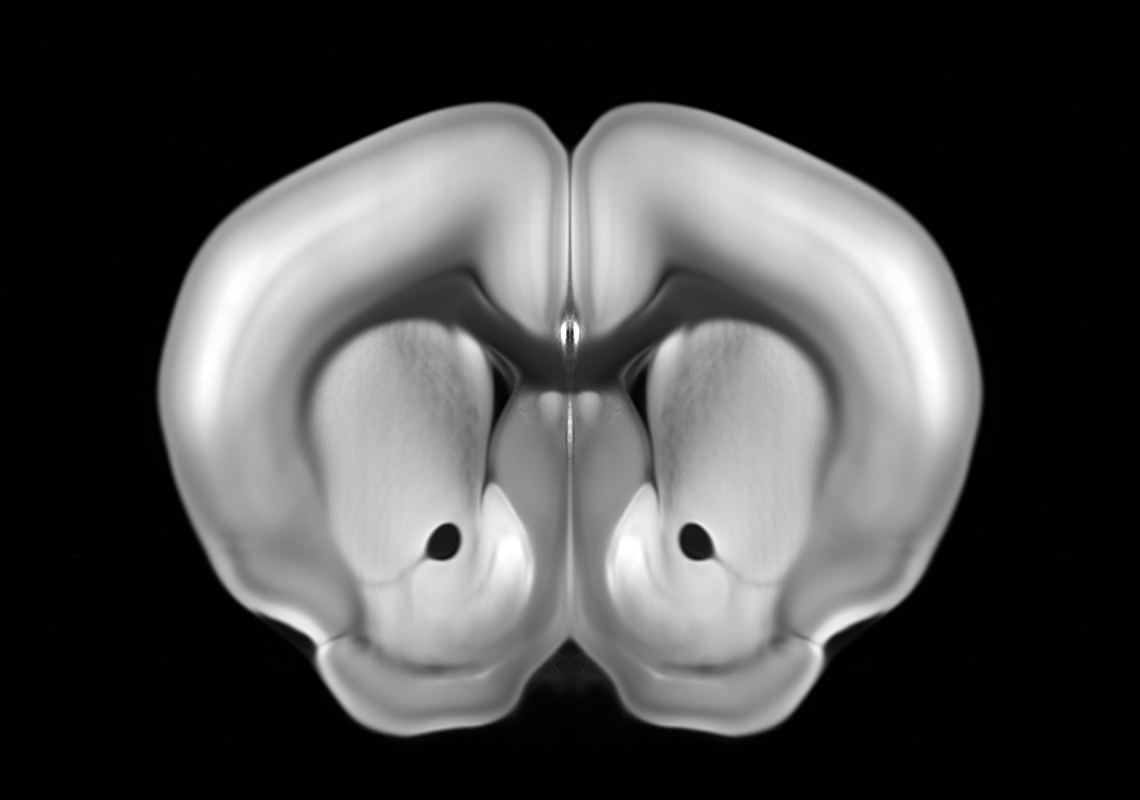

Supplement: Supplementary file 7 — Supplementary Data 5 [file 41467_2019_13057_MOESM7_ESM.zip › Suppl_File2_CCFbackground/AllenCCF_Z034.tif]

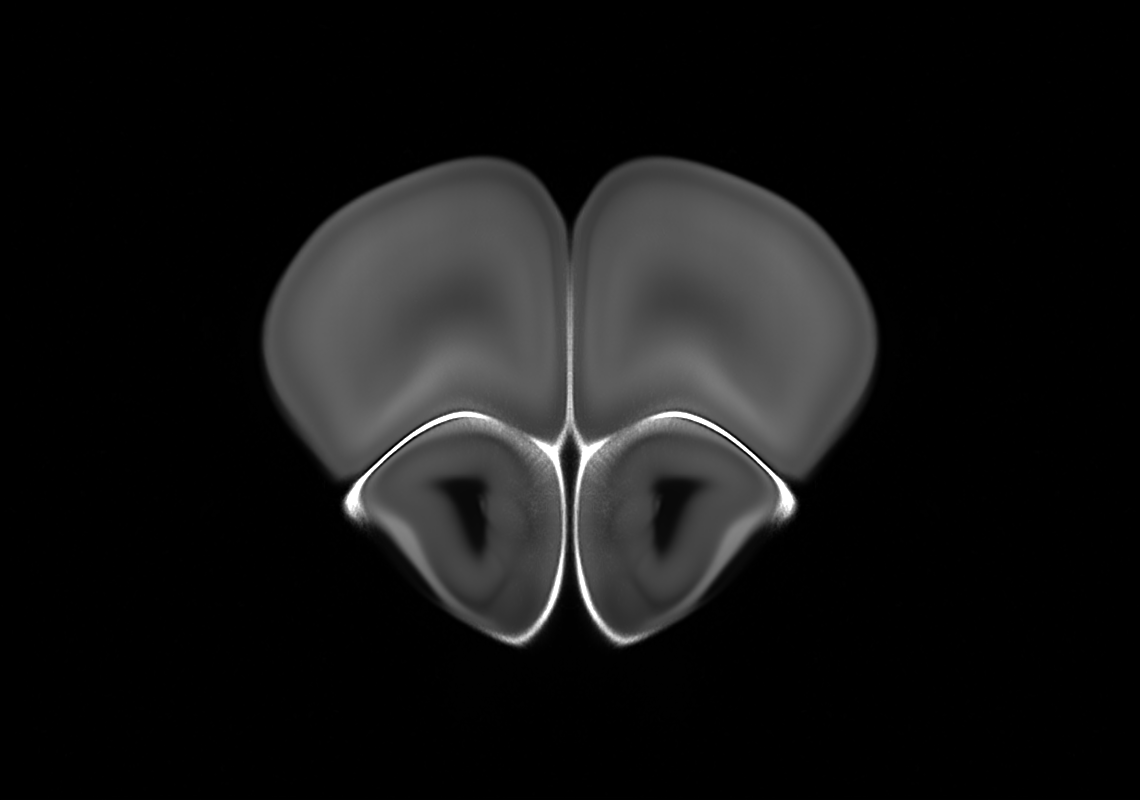

Supplement: Supplementary file 7 — Supplementary Data 5 [file 41467_2019_13057_MOESM7_ESM.zip › Suppl_File2_CCFbackground/AllenCCF_Z020.tif]

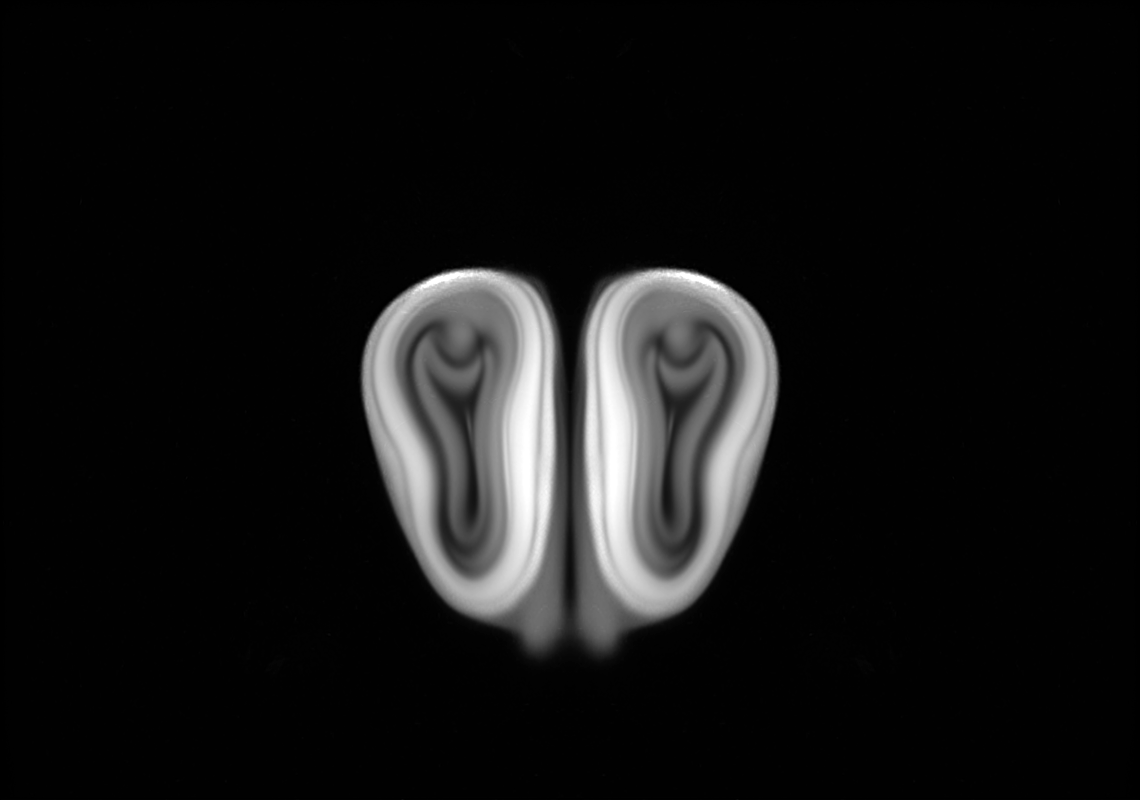

Supplement: Supplementary file 7 — Supplementary Data 5 [file 41467_2019_13057_MOESM7_ESM.zip › Suppl_File2_CCFbackground/AllenCCF_Z008.tif]

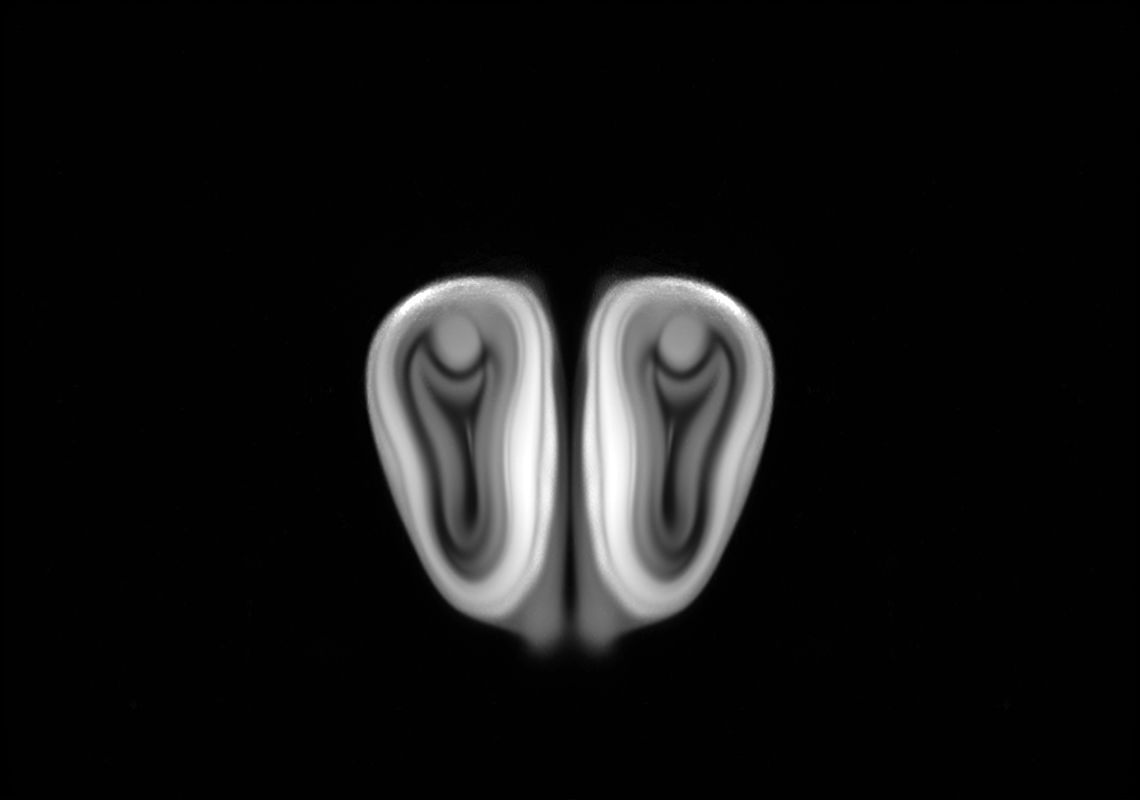

Supplement: Supplementary file 7 — Supplementary Data 5 [file 41467_2019_13057_MOESM7_ESM.zip › Suppl_File2_CCFbackground/AllenCCF_Z009.tif]

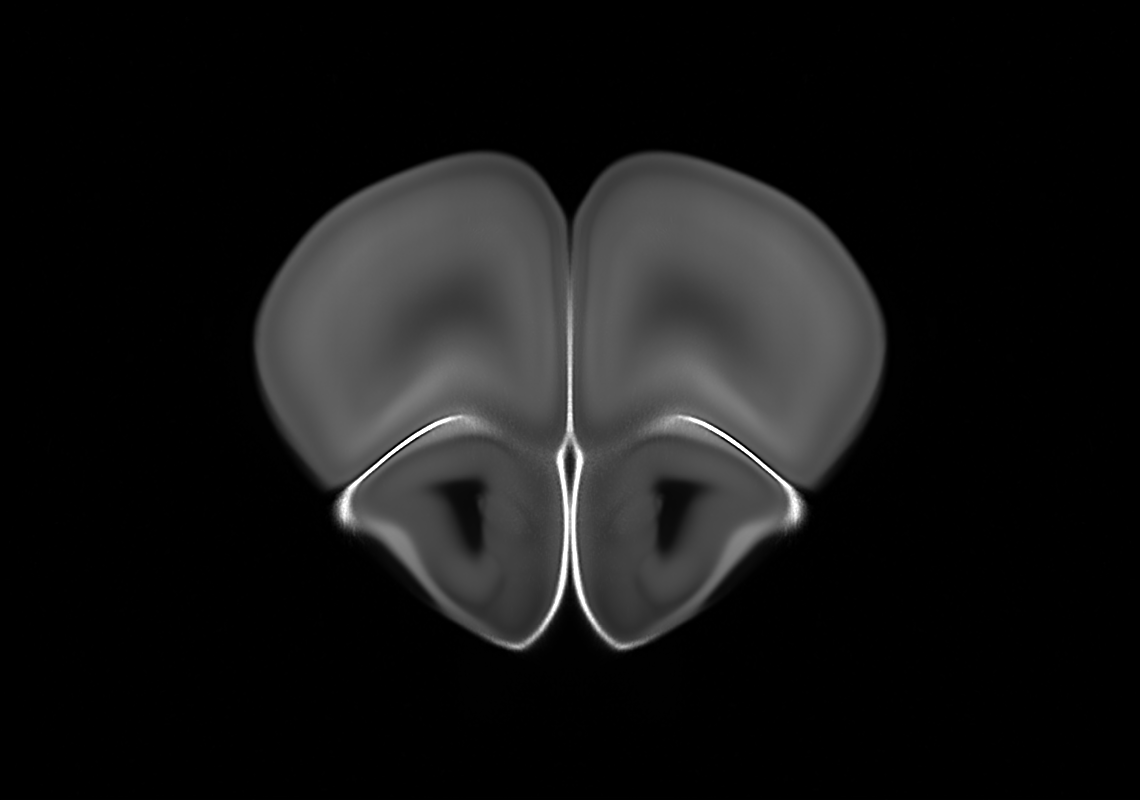

Supplement: Supplementary file 7 — Supplementary Data 5 [file 41467_2019_13057_MOESM7_ESM.zip › Suppl_File2_CCFbackground/AllenCCF_Z021.tif]

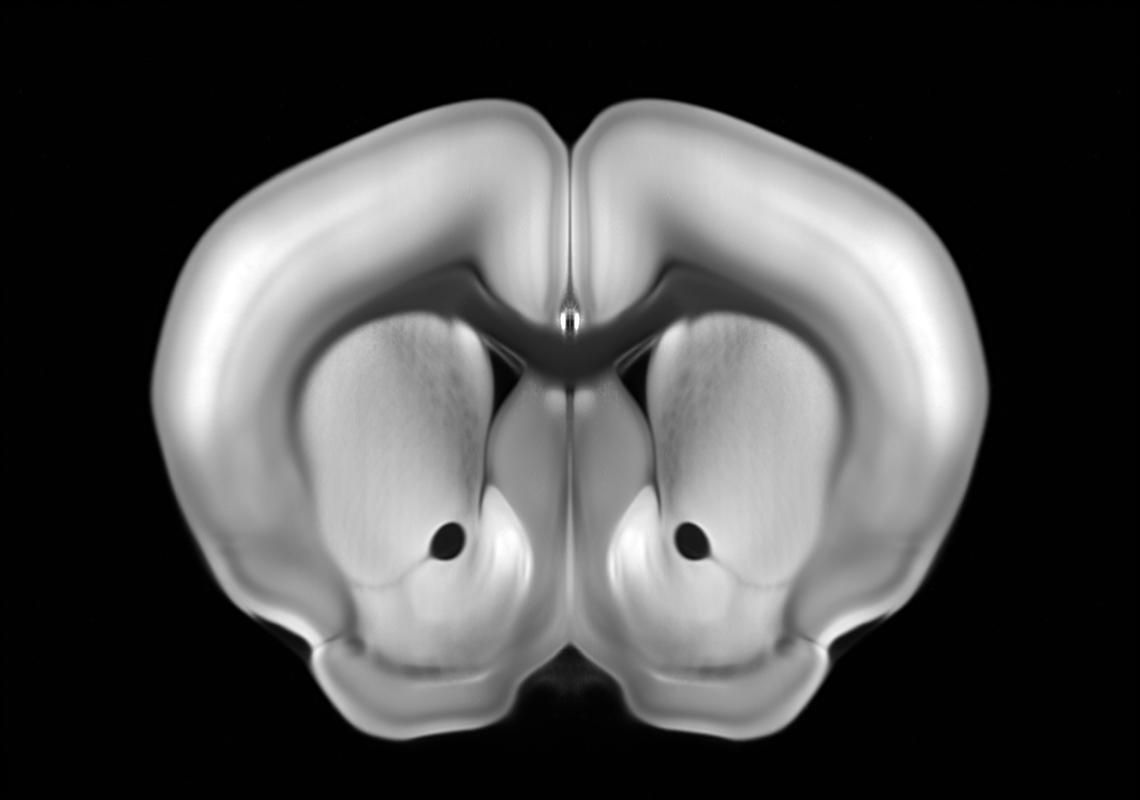

Supplement: Supplementary file 7 — Supplementary Data 5 [file 41467_2019_13057_MOESM7_ESM.zip › Suppl_File2_CCFbackground/AllenCCF_Z035.tif]

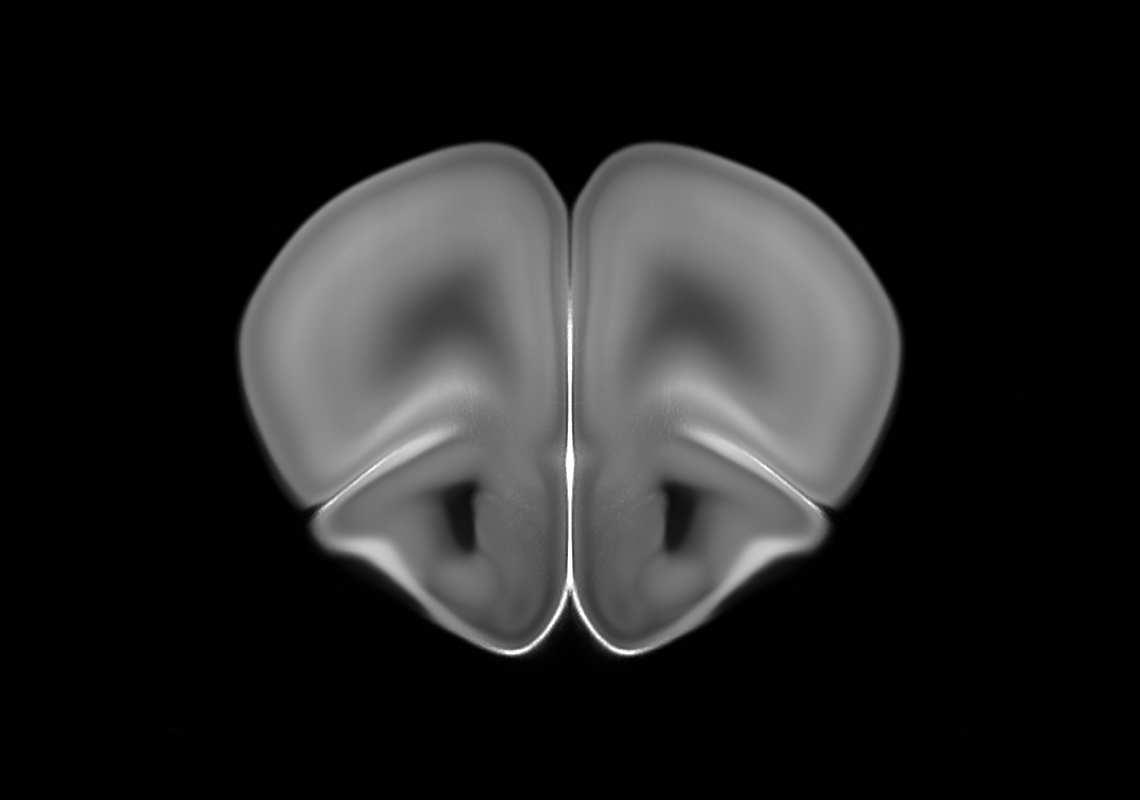

Supplement: Supplementary file 7 — Supplementary Data 5 [file 41467_2019_13057_MOESM7_ESM.zip › Suppl_File2_CCFbackground/AllenCCF_Z023.tif]

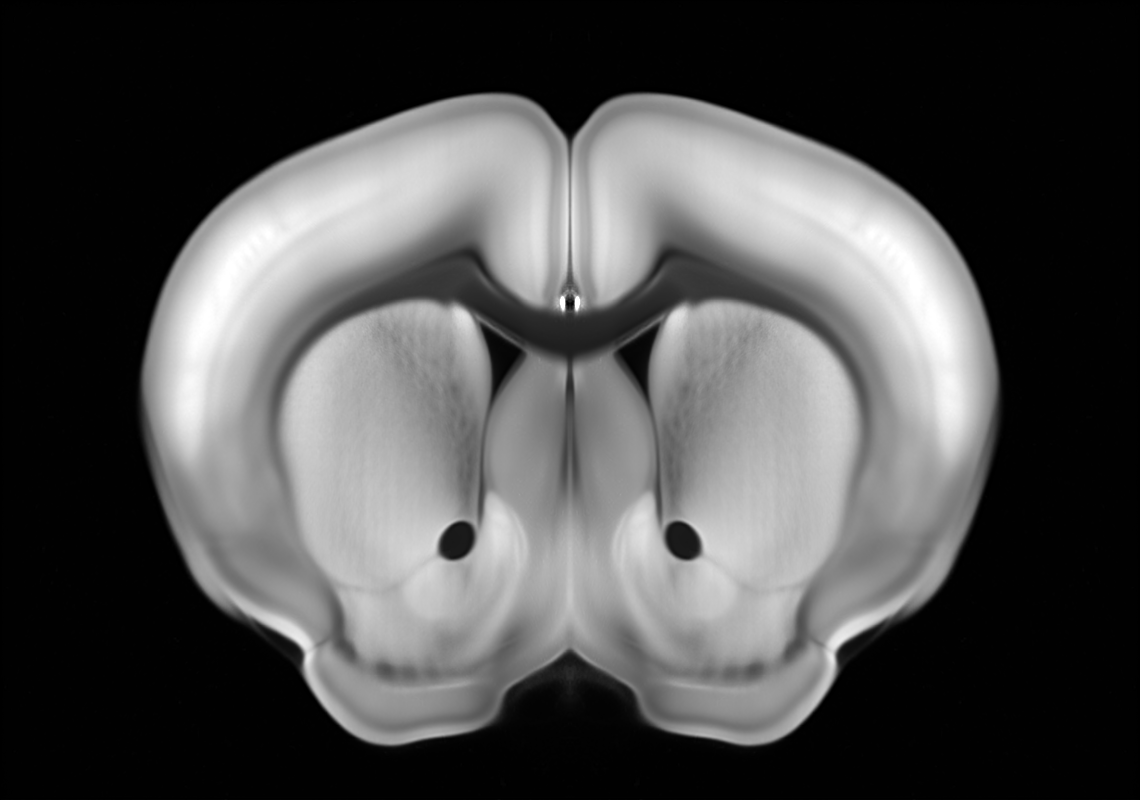

Supplement: Supplementary file 7 — Supplementary Data 5 [file 41467_2019_13057_MOESM7_ESM.zip › Suppl_File2_CCFbackground/AllenCCF_Z037.tif]

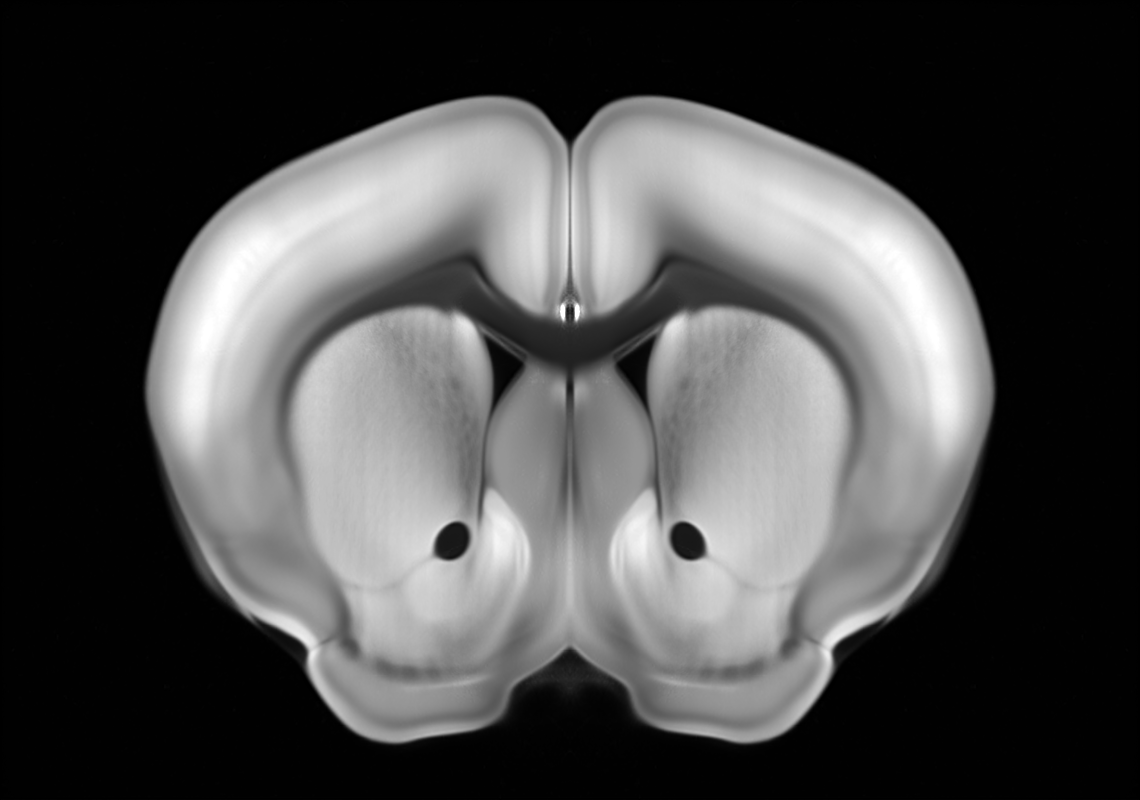

Supplement: Supplementary file 7 — Supplementary Data 5 [file 41467_2019_13057_MOESM7_ESM.zip › Suppl_File2_CCFbackground/AllenCCF_Z036.tif]

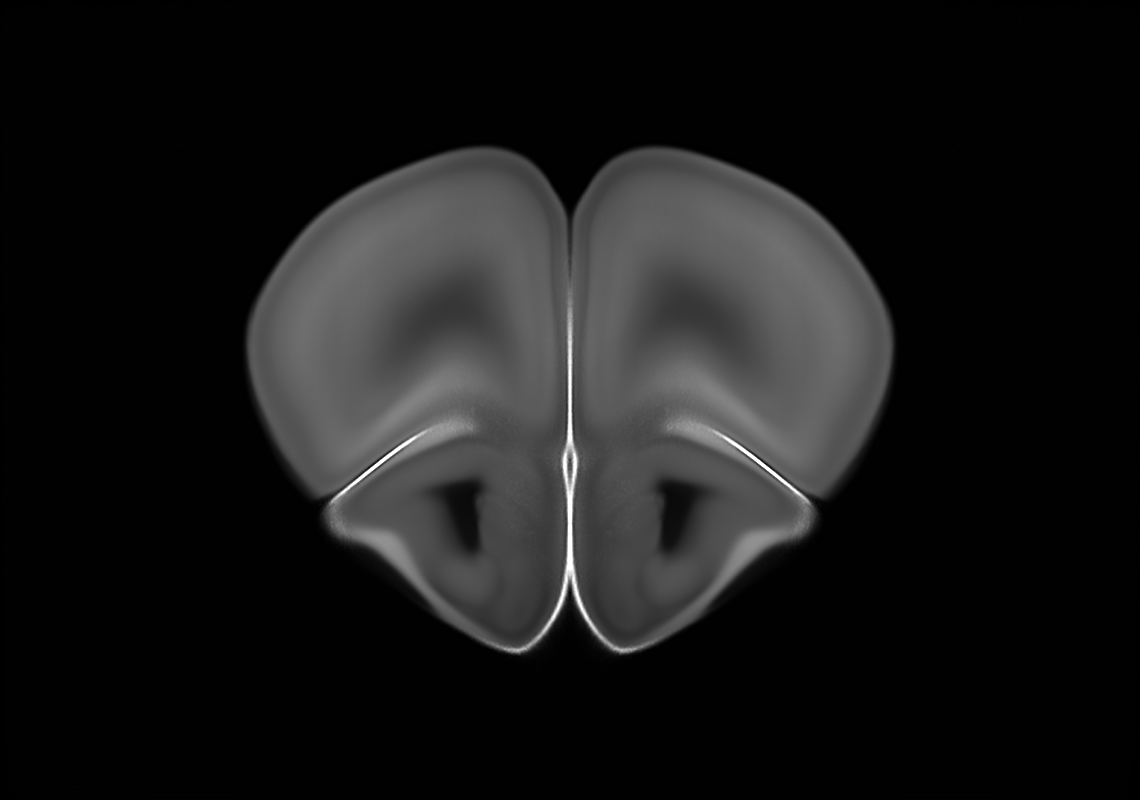

Supplement: Supplementary file 7 — Supplementary Data 5 [file 41467_2019_13057_MOESM7_ESM.zip › Suppl_File2_CCFbackground/AllenCCF_Z022.tif]

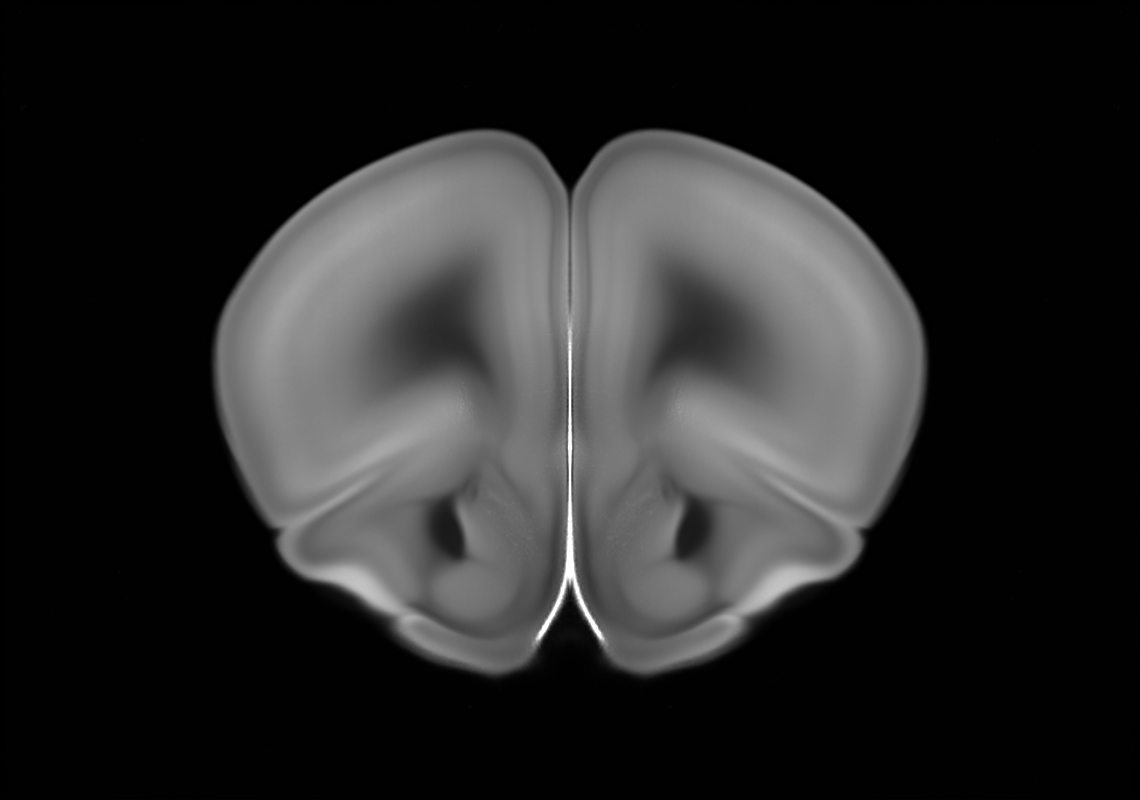

Supplement: Supplementary file 7 — Supplementary Data 5 [file 41467_2019_13057_MOESM7_ESM.zip › Suppl_File2_CCFbackground/AllenCCF_Z026.tif]

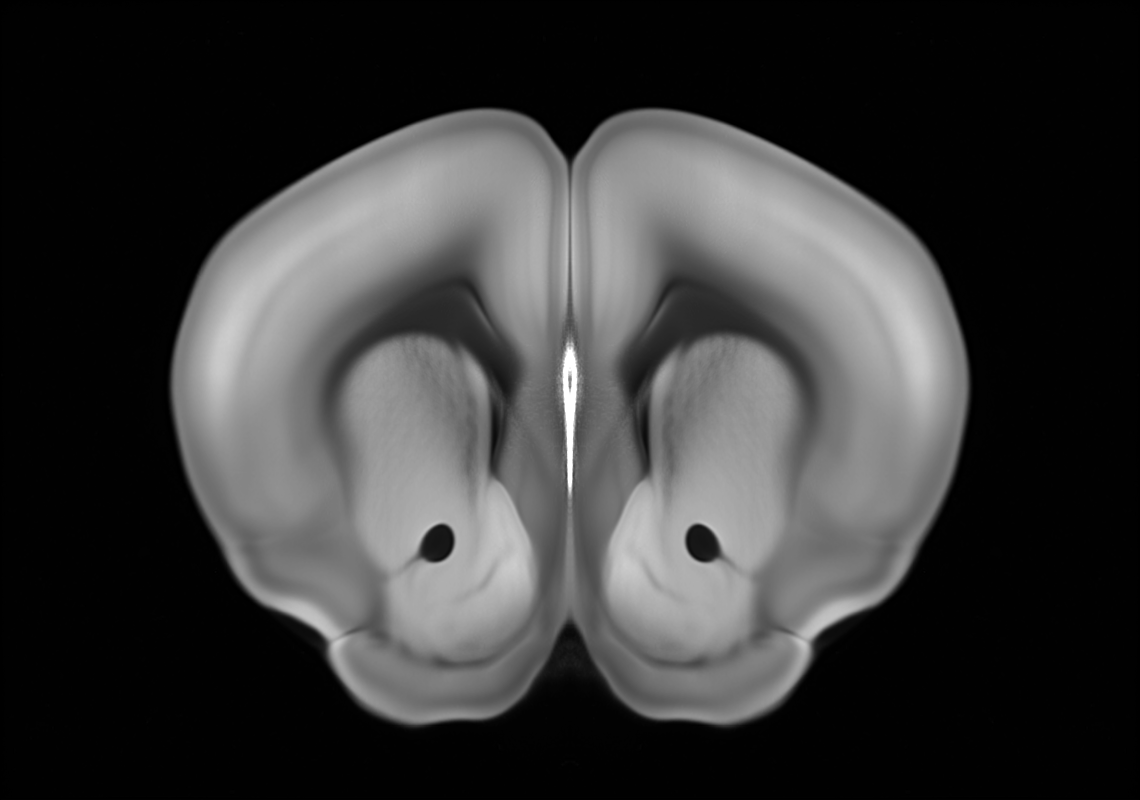

Supplement: Supplementary file 7 — Supplementary Data 5 [file 41467_2019_13057_MOESM7_ESM.zip › Suppl_File2_CCFbackground/AllenCCF_Z032.tif]

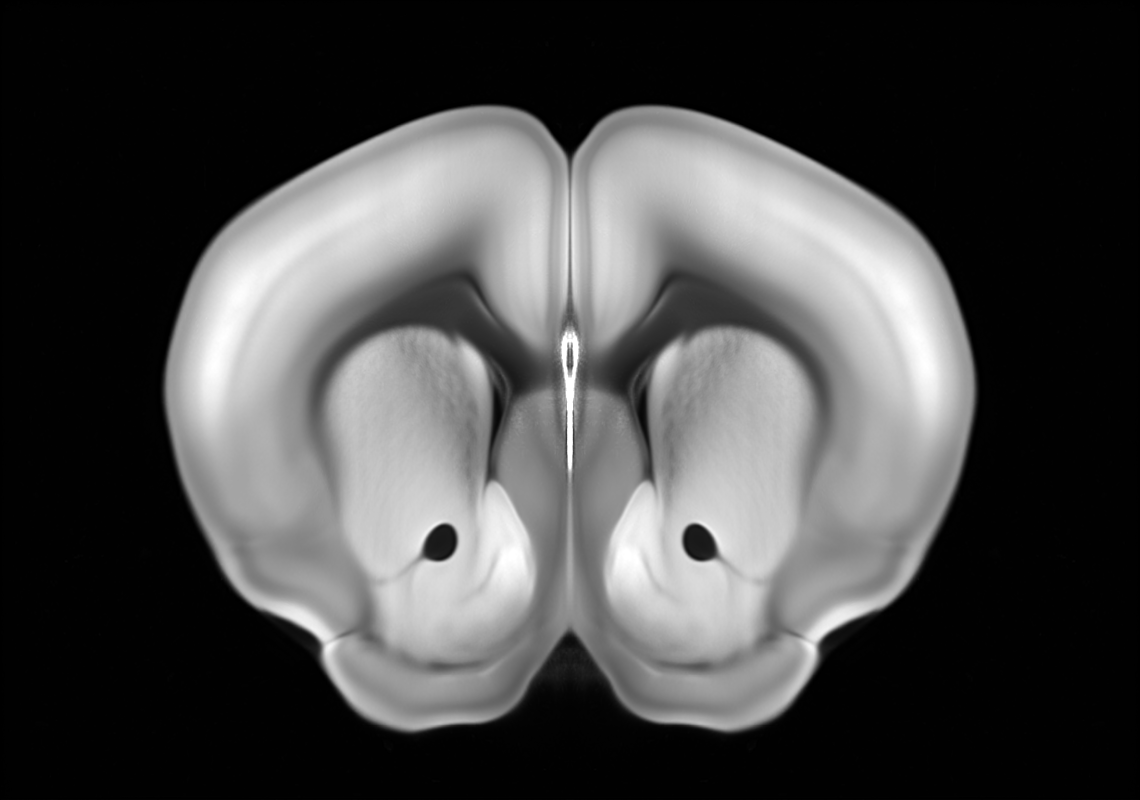

Supplement: Supplementary file 7 — Supplementary Data 5 [file 41467_2019_13057_MOESM7_ESM.zip › Suppl_File2_CCFbackground/AllenCCF_Z033.tif]

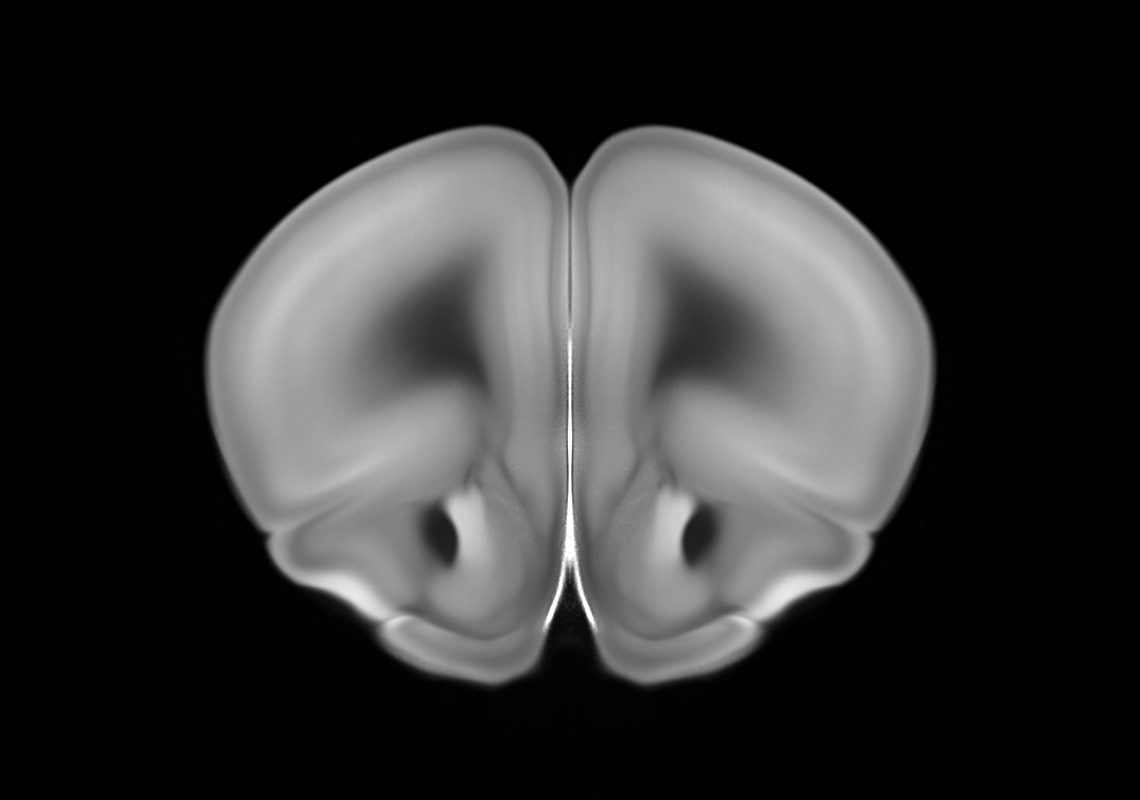

Supplement: Supplementary file 7 — Supplementary Data 5 [file 41467_2019_13057_MOESM7_ESM.zip › Suppl_File2_CCFbackground/AllenCCF_Z027.tif]

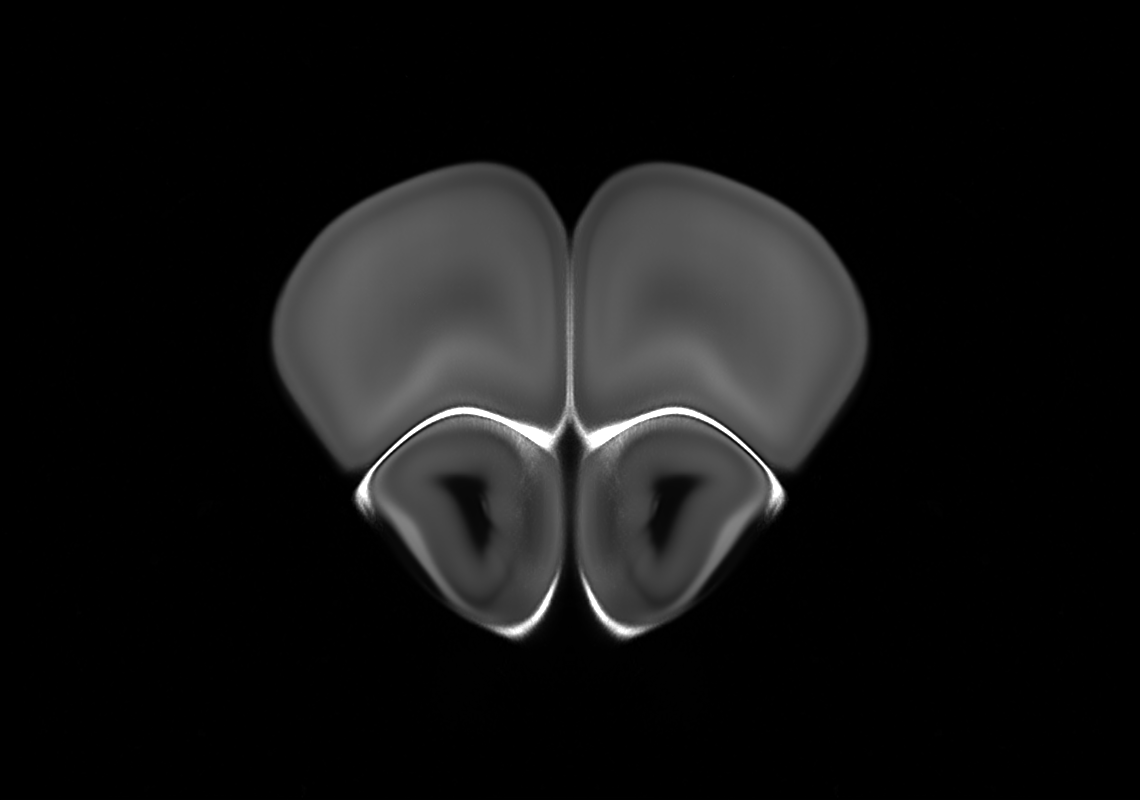

Supplement: Supplementary file 7 — Supplementary Data 5 [file 41467_2019_13057_MOESM7_ESM.zip › Suppl_File2_CCFbackground/AllenCCF_Z019.tif]

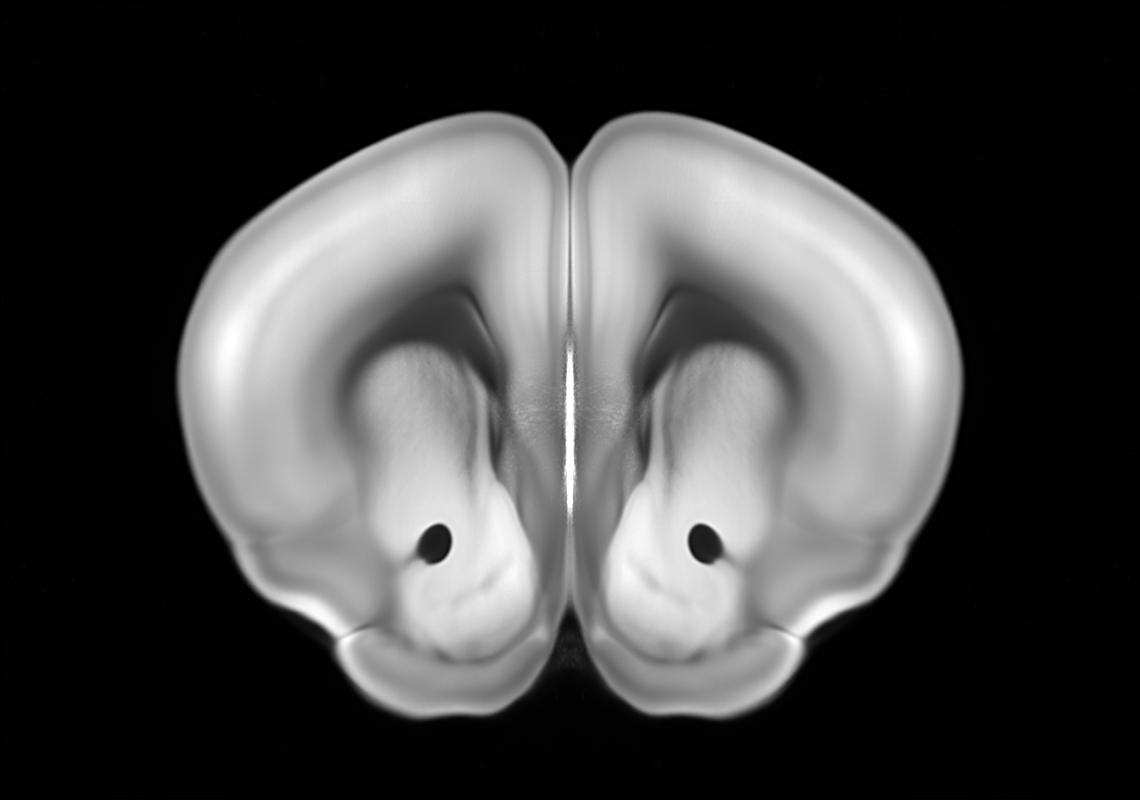

Supplement: Supplementary file 7 — Supplementary Data 5 [file 41467_2019_13057_MOESM7_ESM.zip › Suppl_File2_CCFbackground/AllenCCF_Z031.tif]

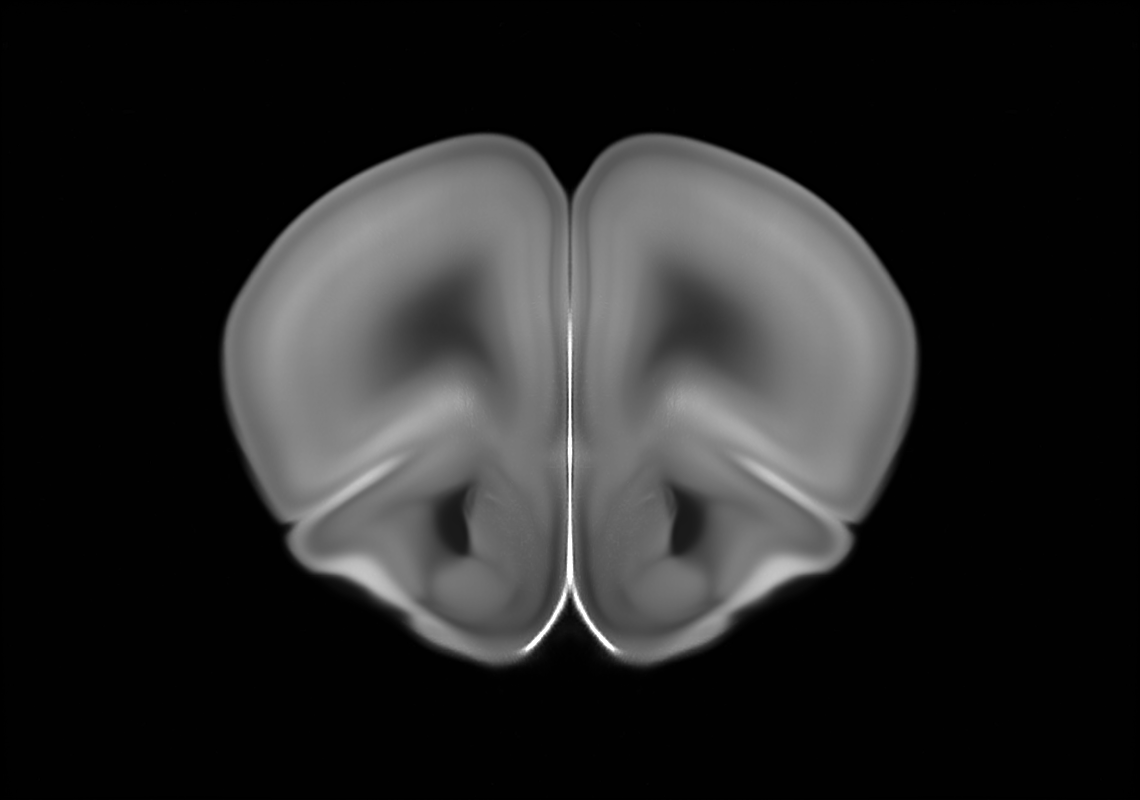

Supplement: Supplementary file 7 — Supplementary Data 5 [file 41467_2019_13057_MOESM7_ESM.zip › Suppl_File2_CCFbackground/AllenCCF_Z025.tif]

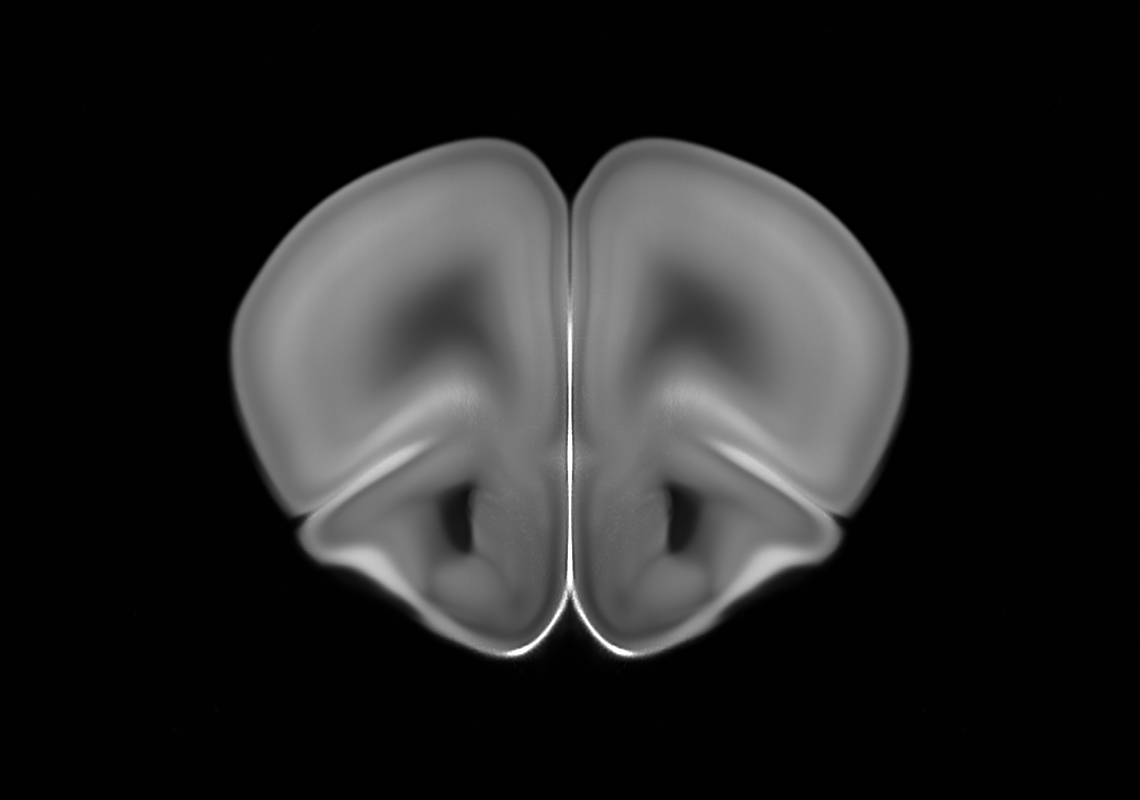

Supplement: Supplementary file 7 — Supplementary Data 5 [file 41467_2019_13057_MOESM7_ESM.zip › Suppl_File2_CCFbackground/AllenCCF_Z024.tif]

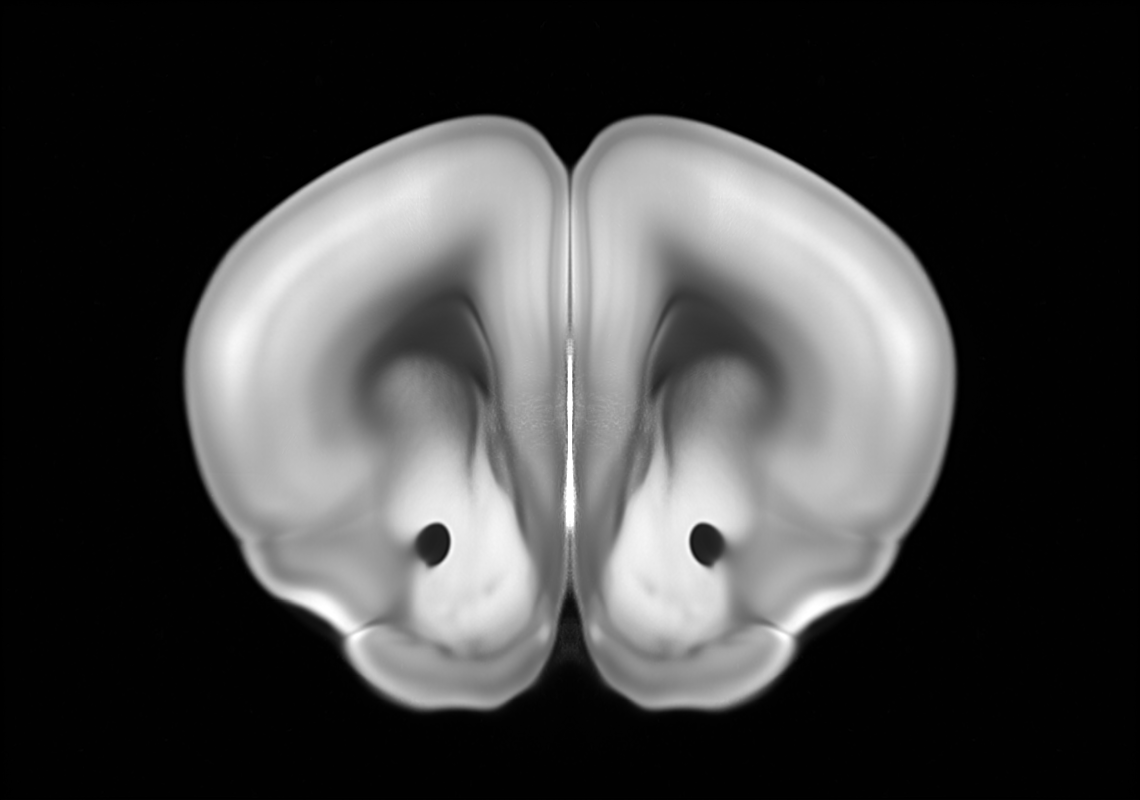

Supplement: Supplementary file 7 — Supplementary Data 5 [file 41467_2019_13057_MOESM7_ESM.zip › Suppl_File2_CCFbackground/AllenCCF_Z030.tif]

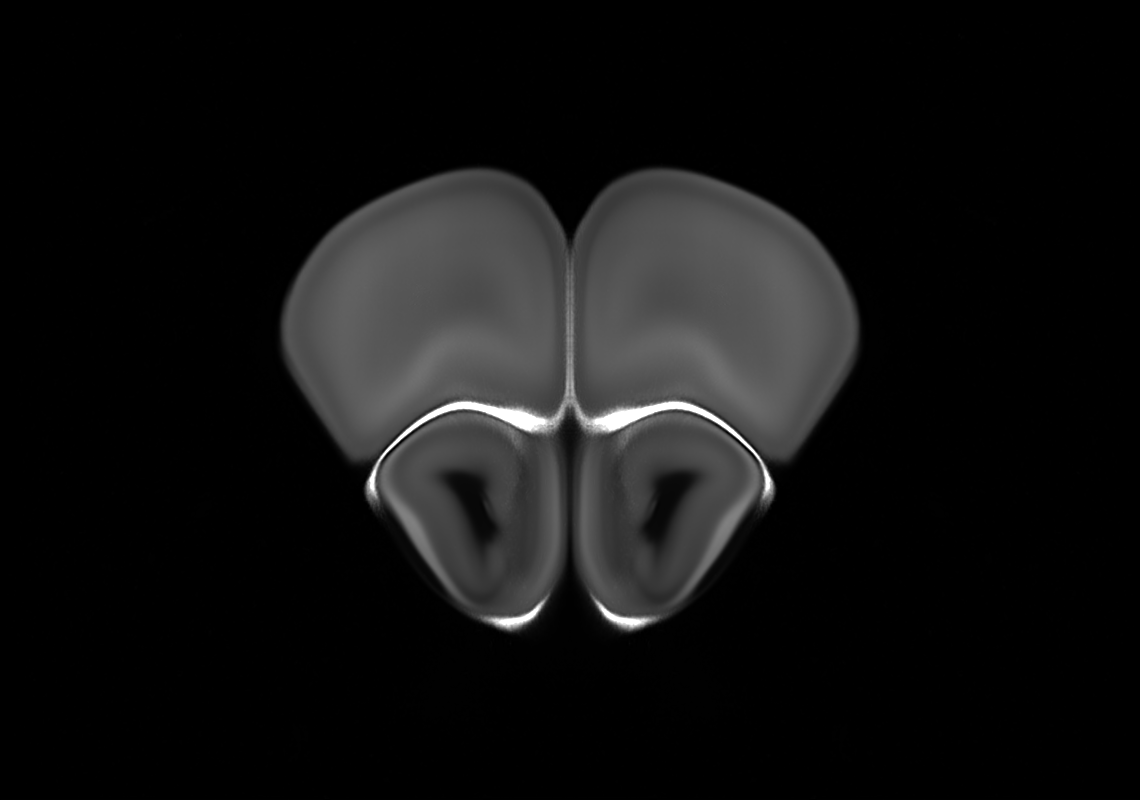

Supplement: Supplementary file 7 — Supplementary Data 5 [file 41467_2019_13057_MOESM7_ESM.zip › Suppl_File2_CCFbackground/AllenCCF_Z018.tif]

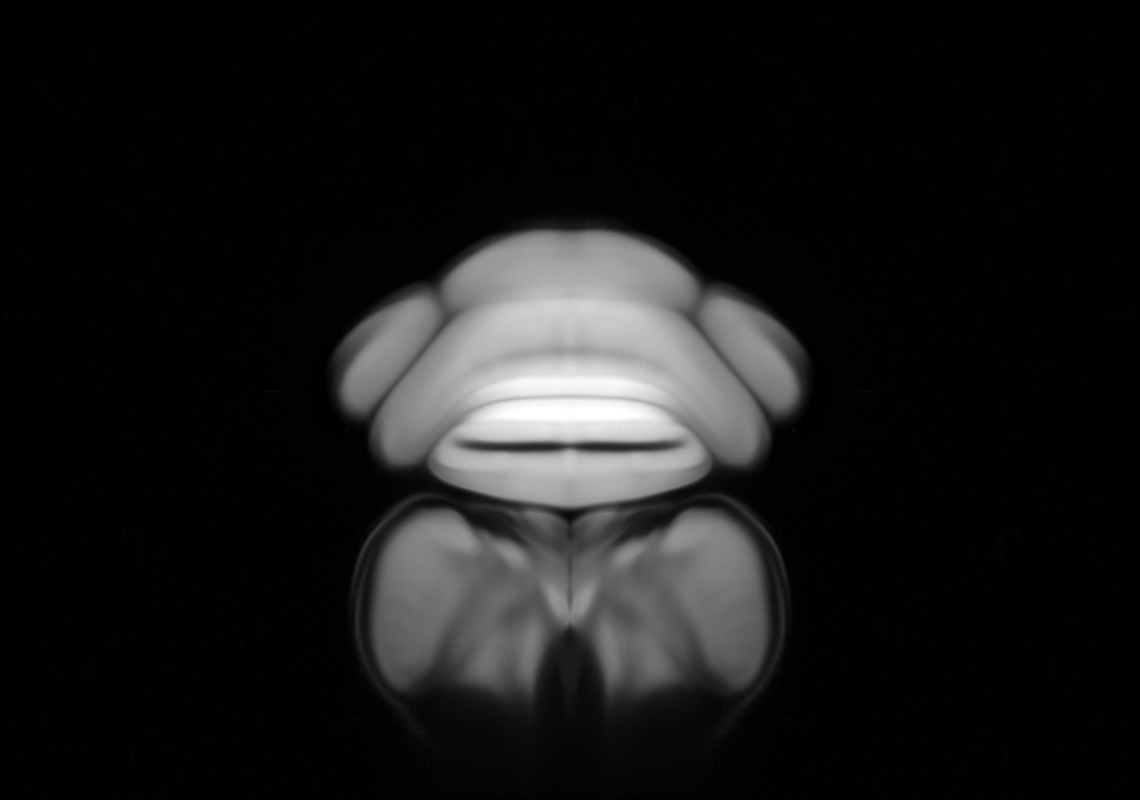

Supplement: Supplementary file 7 — Supplementary Data 5 [file 41467_2019_13057_MOESM7_ESM.zip › Suppl_File2_CCFbackground/AllenCCF_Z123.tif]

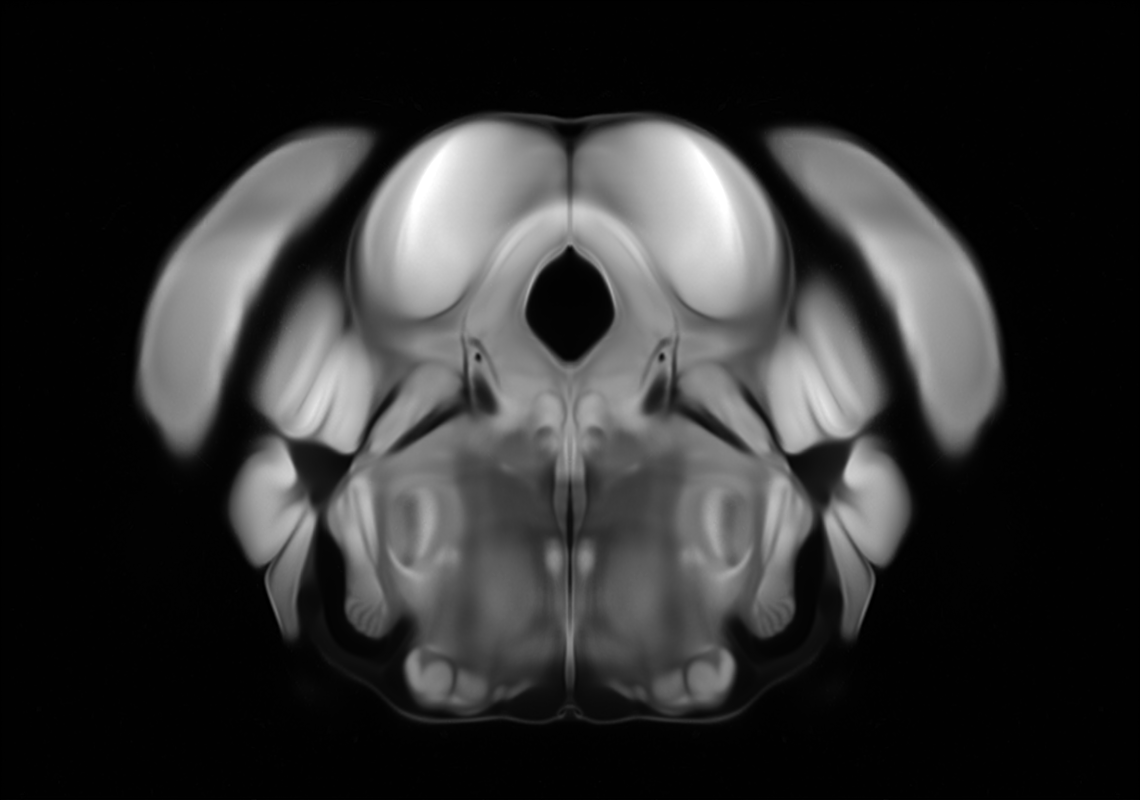

Supplement: Supplementary file 7 — Supplementary Data 5 [file 41467_2019_13057_MOESM7_ESM.zip › Suppl_File2_CCFbackground/AllenCCF_Z094.tif]

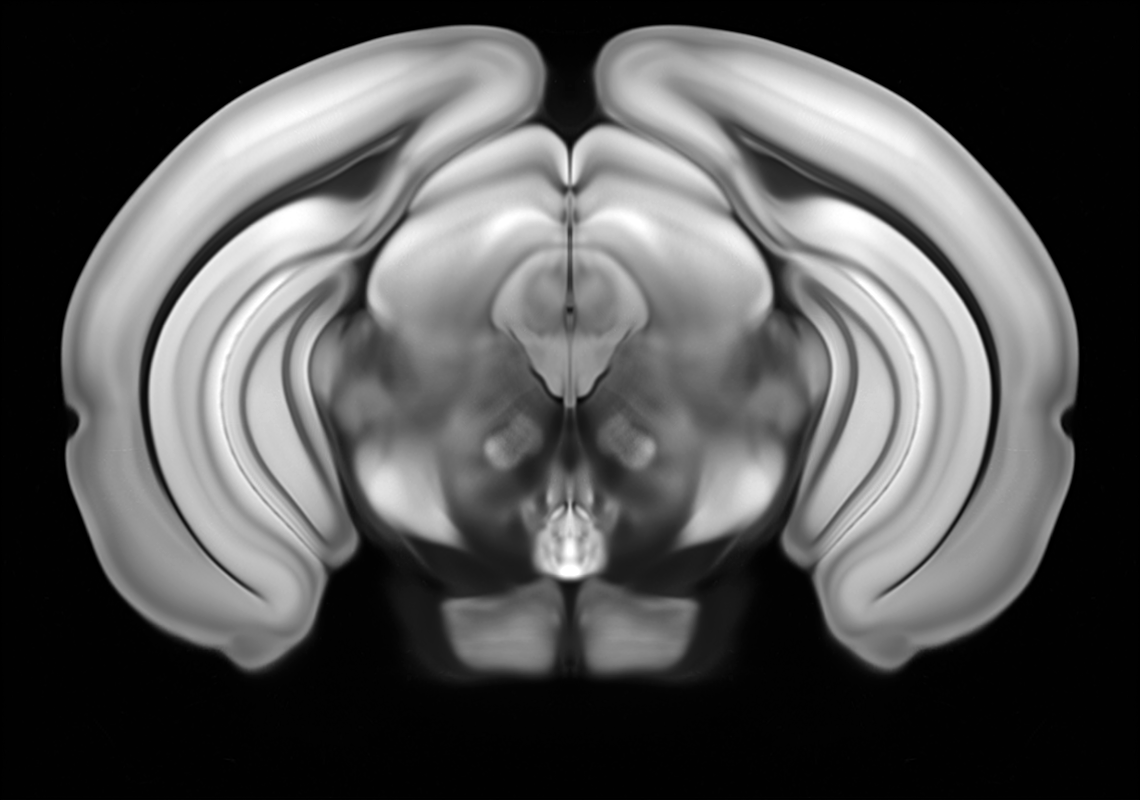

Supplement: Supplementary file 7 — Supplementary Data 5 [file 41467_2019_13057_MOESM7_ESM.zip › Suppl_File2_CCFbackground/AllenCCF_Z080.tif]

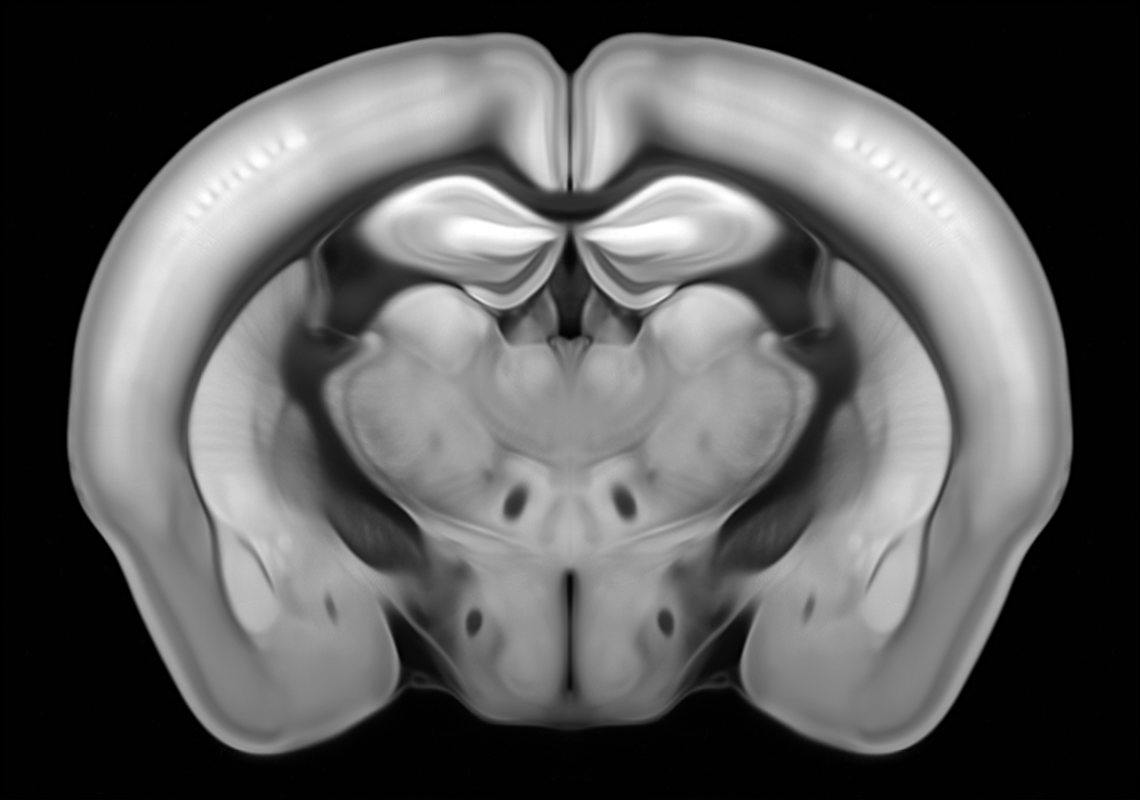

Supplement: Supplementary file 7 — Supplementary Data 5 [file 41467_2019_13057_MOESM7_ESM.zip › Suppl_File2_CCFbackground/AllenCCF_Z057.tif]

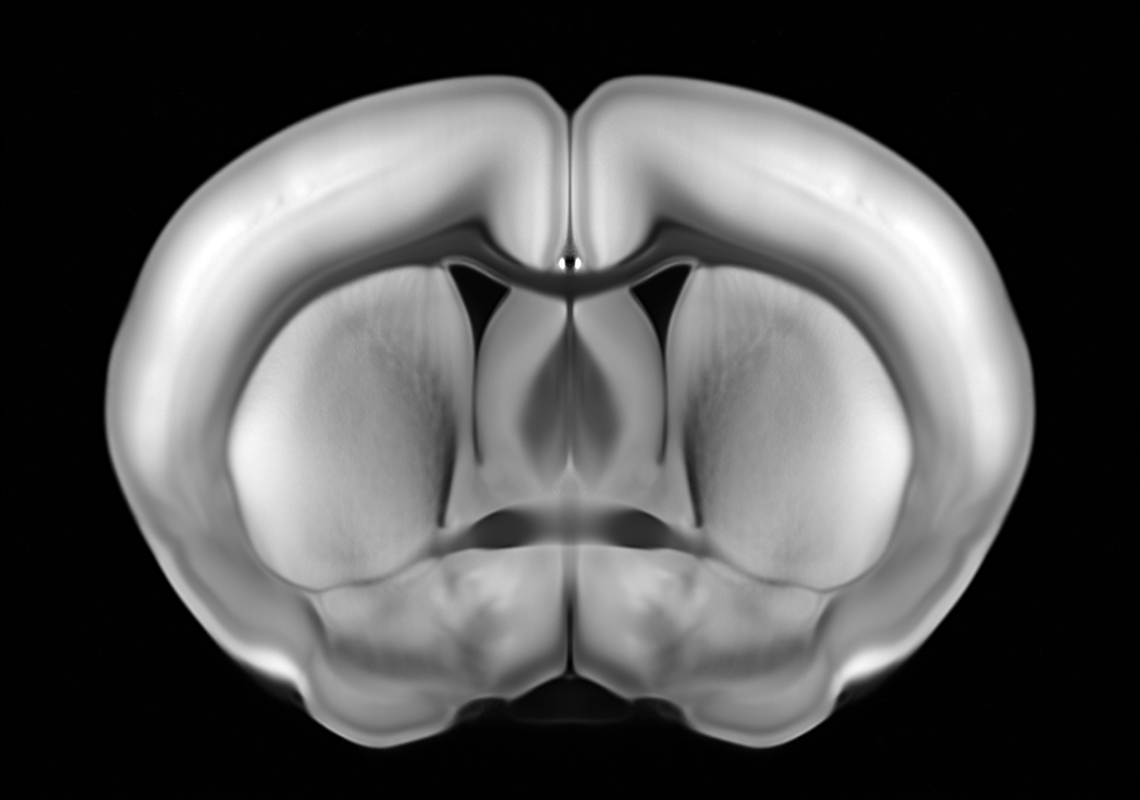

Supplement: Supplementary file 7 — Supplementary Data 5 [file 41467_2019_13057_MOESM7_ESM.zip › Suppl_File2_CCFbackground/AllenCCF_Z043.tif]

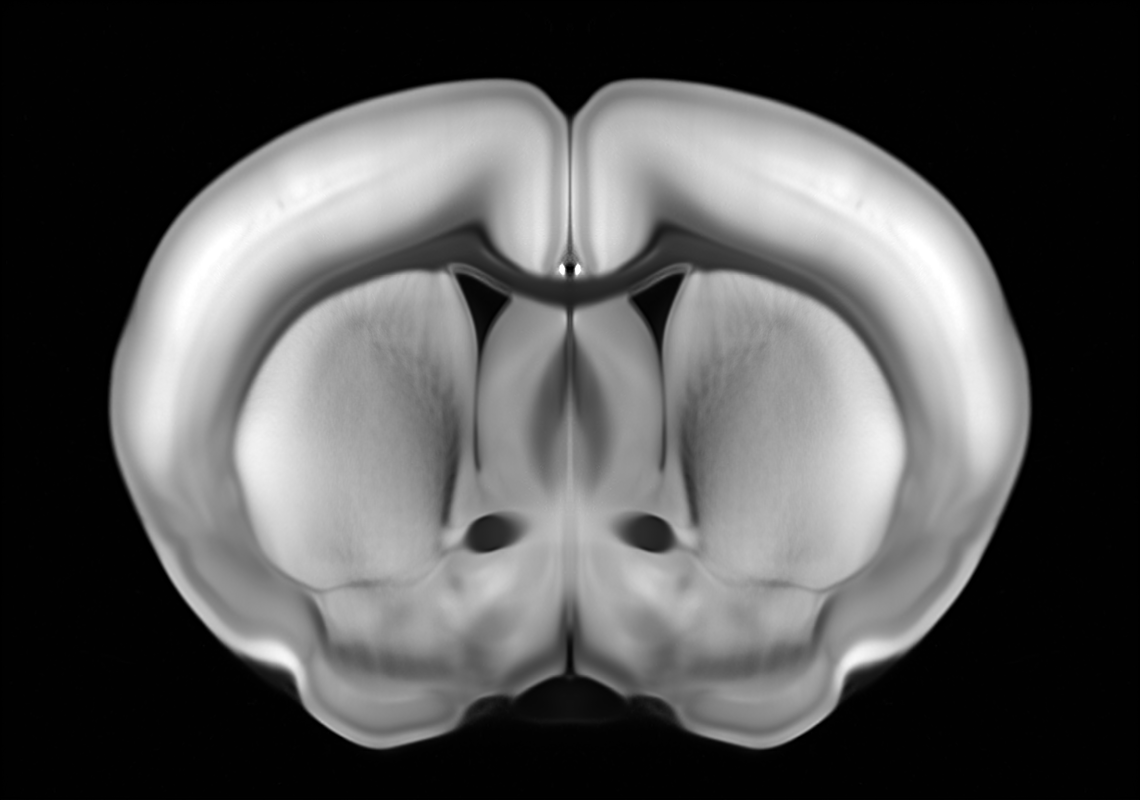

Supplement: Supplementary file 7 — Supplementary Data 5 [file 41467_2019_13057_MOESM7_ESM.zip › Suppl_File2_CCFbackground/AllenCCF_Z042.tif]

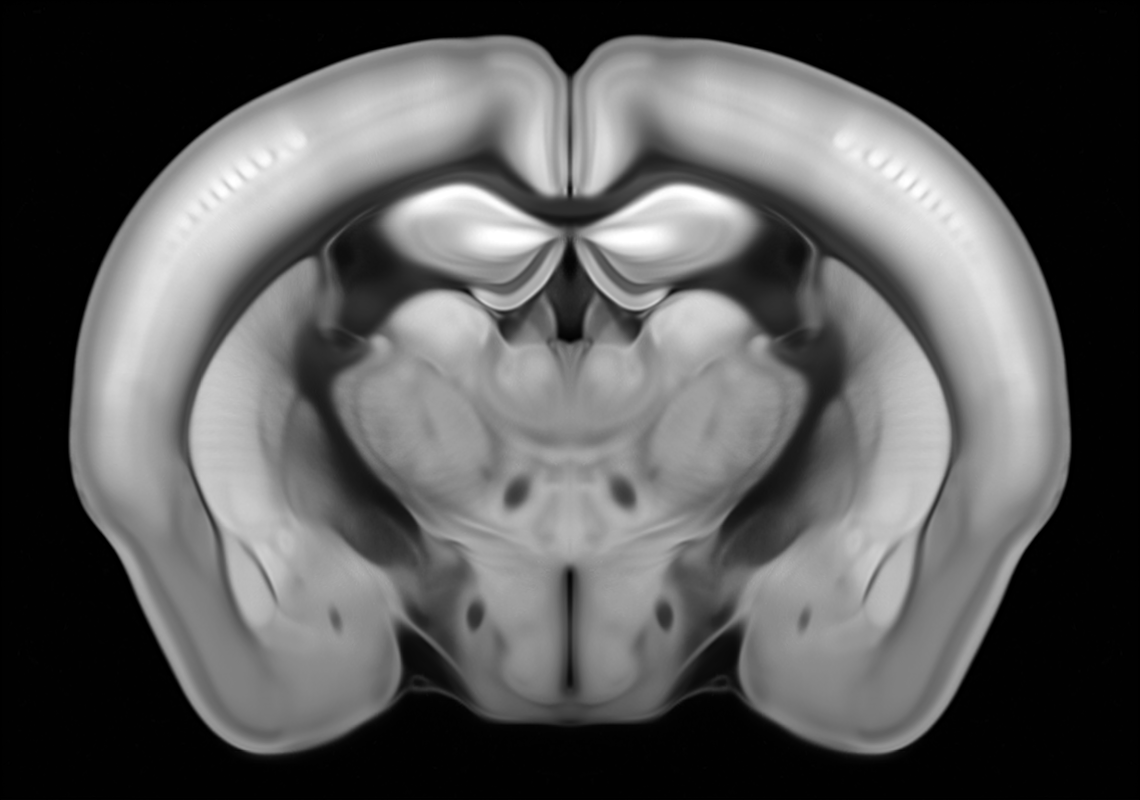

Supplement: Supplementary file 7 — Supplementary Data 5 [file 41467_2019_13057_MOESM7_ESM.zip › Suppl_File2_CCFbackground/AllenCCF_Z056.tif]

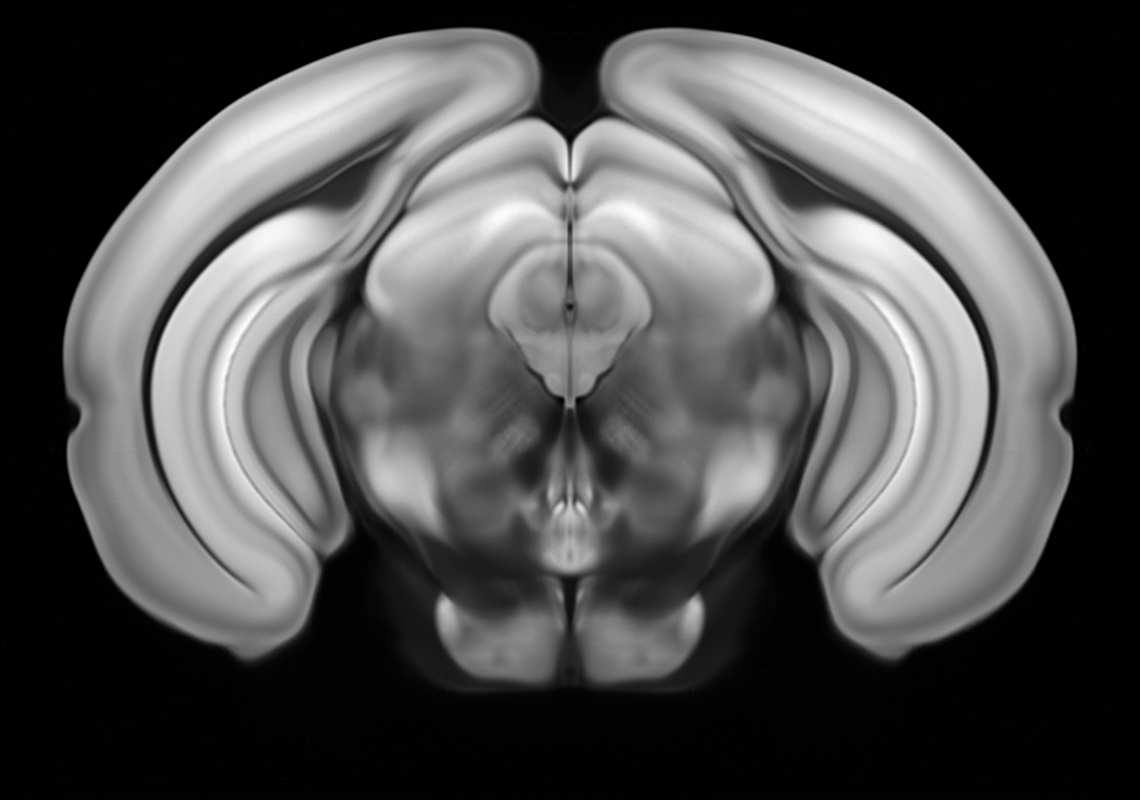

Supplement: Supplementary file 7 — Supplementary Data 5 [file 41467_2019_13057_MOESM7_ESM.zip › Suppl_File2_CCFbackground/AllenCCF_Z081.tif]

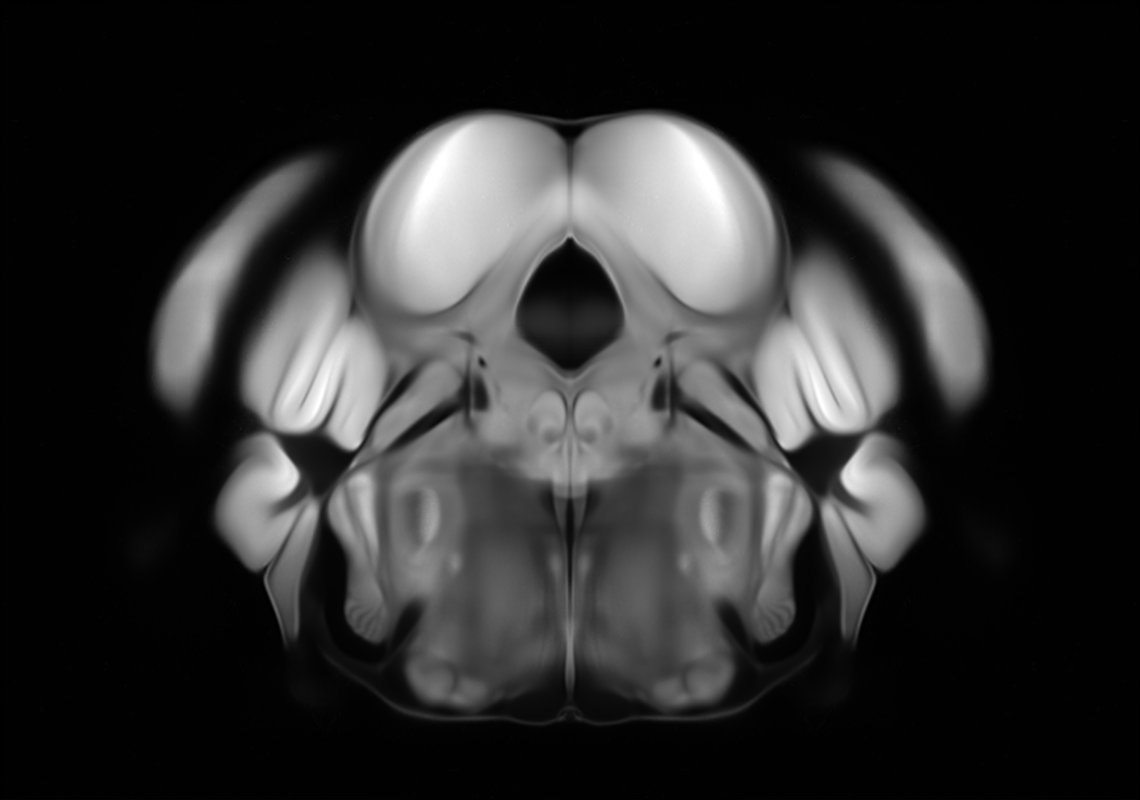

Supplement: Supplementary file 7 — Supplementary Data 5 [file 41467_2019_13057_MOESM7_ESM.zip › Suppl_File2_CCFbackground/AllenCCF_Z095.tif]

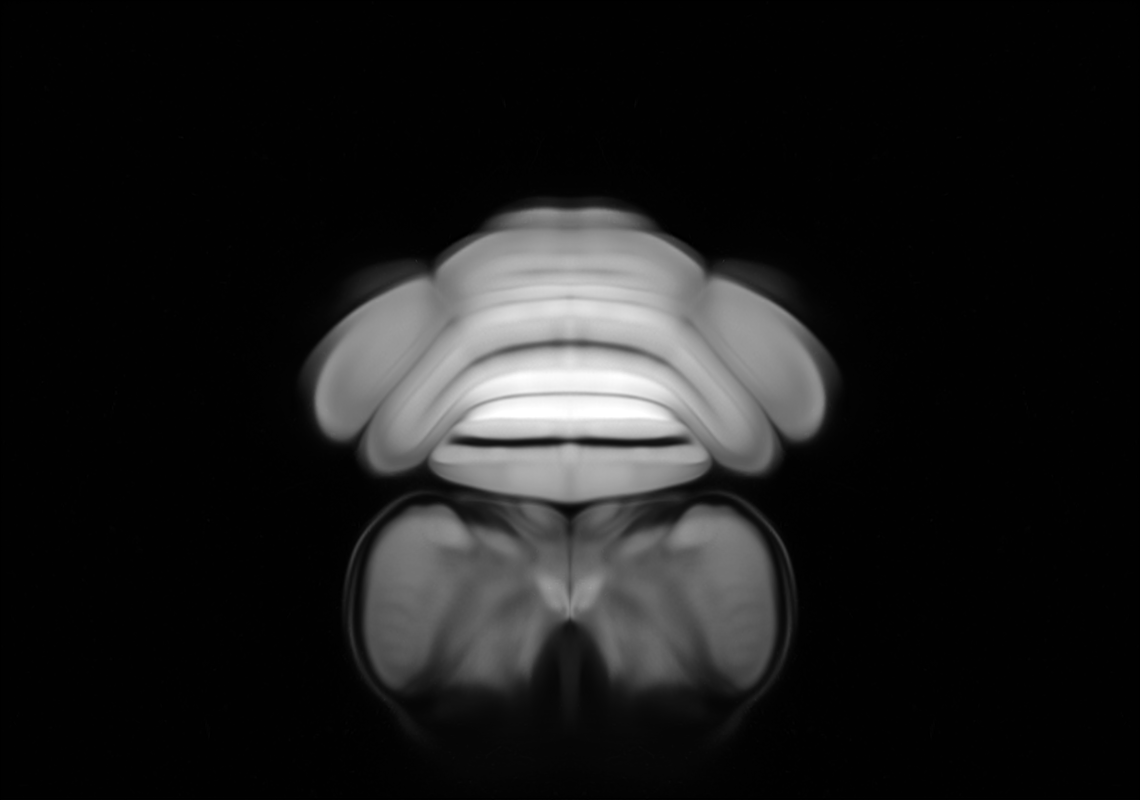

Supplement: Supplementary file 7 — Supplementary Data 5 [file 41467_2019_13057_MOESM7_ESM.zip › Suppl_File2_CCFbackground/AllenCCF_Z122.tif]

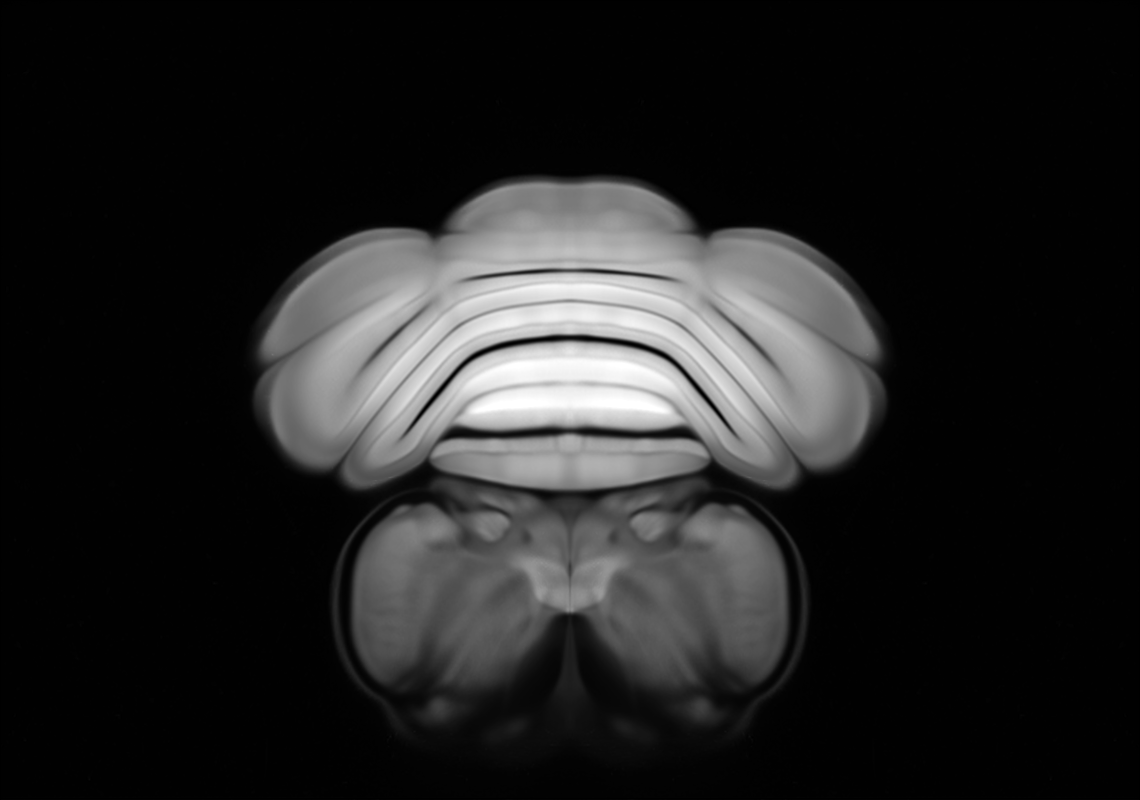

Supplement: Supplementary file 7 — Supplementary Data 5 [file 41467_2019_13057_MOESM7_ESM.zip › Suppl_File2_CCFbackground/AllenCCF_Z120.tif]

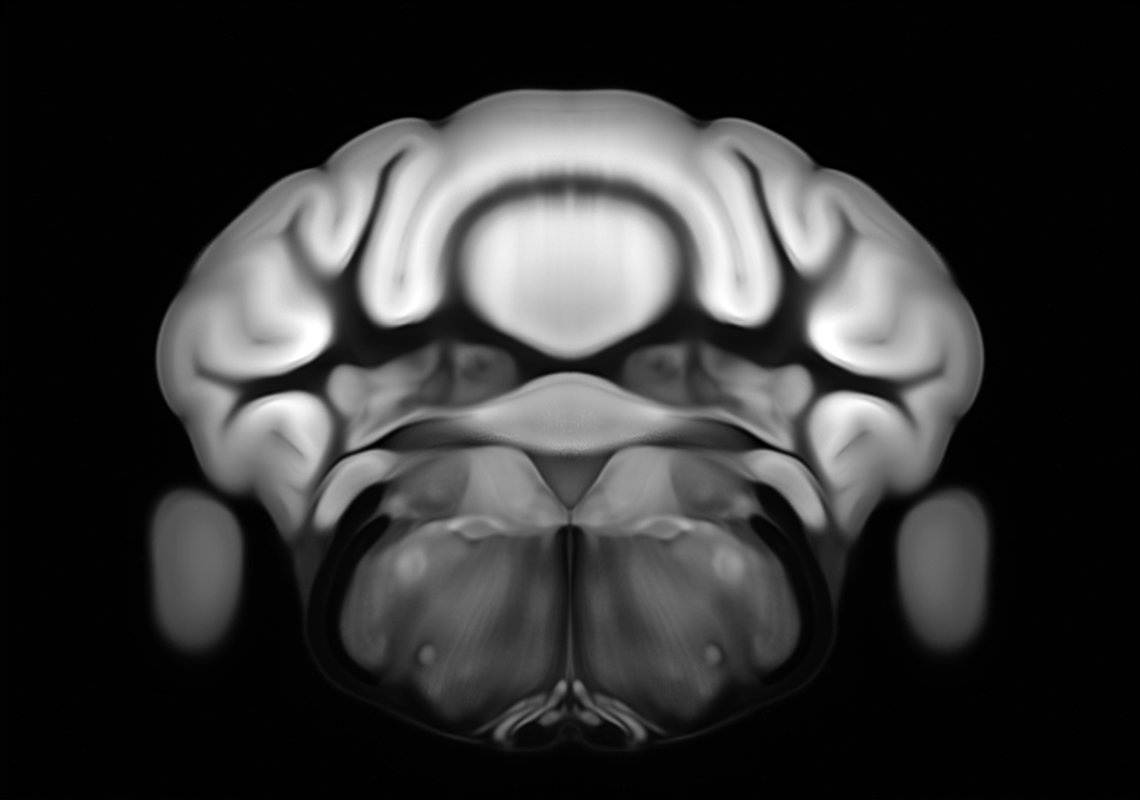

Supplement: Supplementary file 7 — Supplementary Data 5 [file 41467_2019_13057_MOESM7_ESM.zip › Suppl_File2_CCFbackground/AllenCCF_Z108.tif]

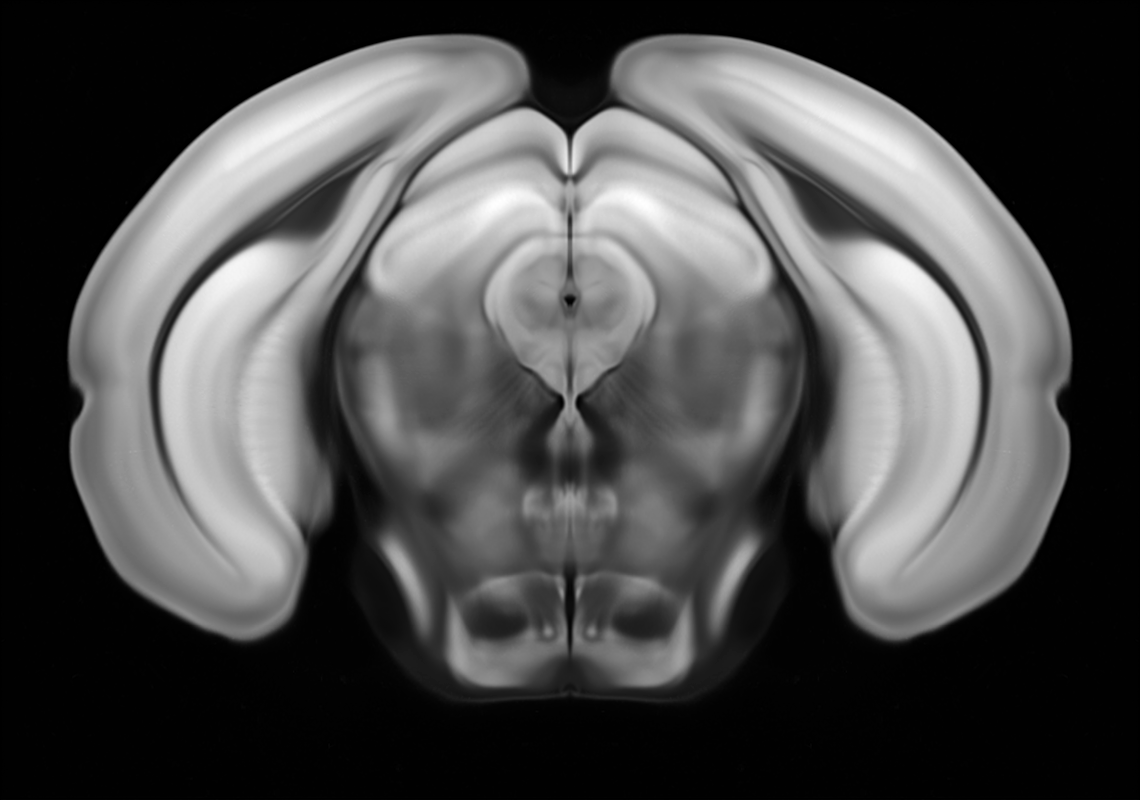

Supplement: Supplementary file 7 — Supplementary Data 5 [file 41467_2019_13057_MOESM7_ESM.zip › Suppl_File2_CCFbackground/AllenCCF_Z083.tif]

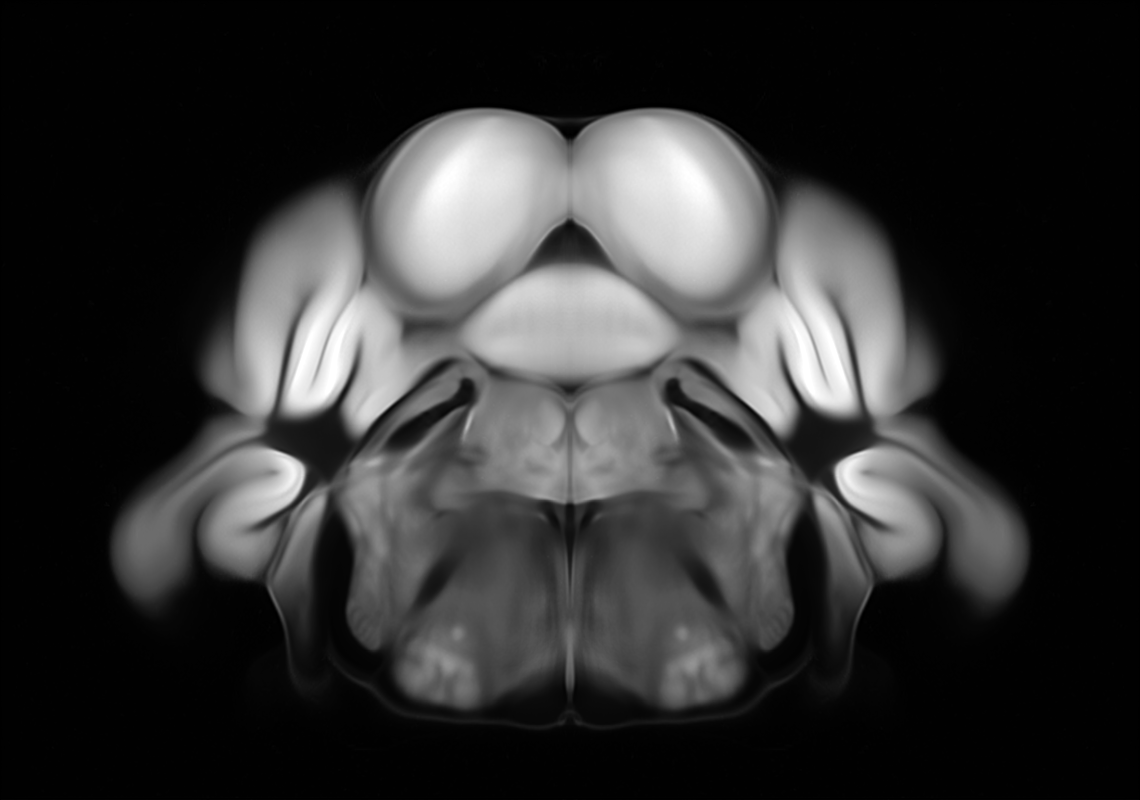

Supplement: Supplementary file 7 — Supplementary Data 5 [file 41467_2019_13057_MOESM7_ESM.zip › Suppl_File2_CCFbackground/AllenCCF_Z097.tif]

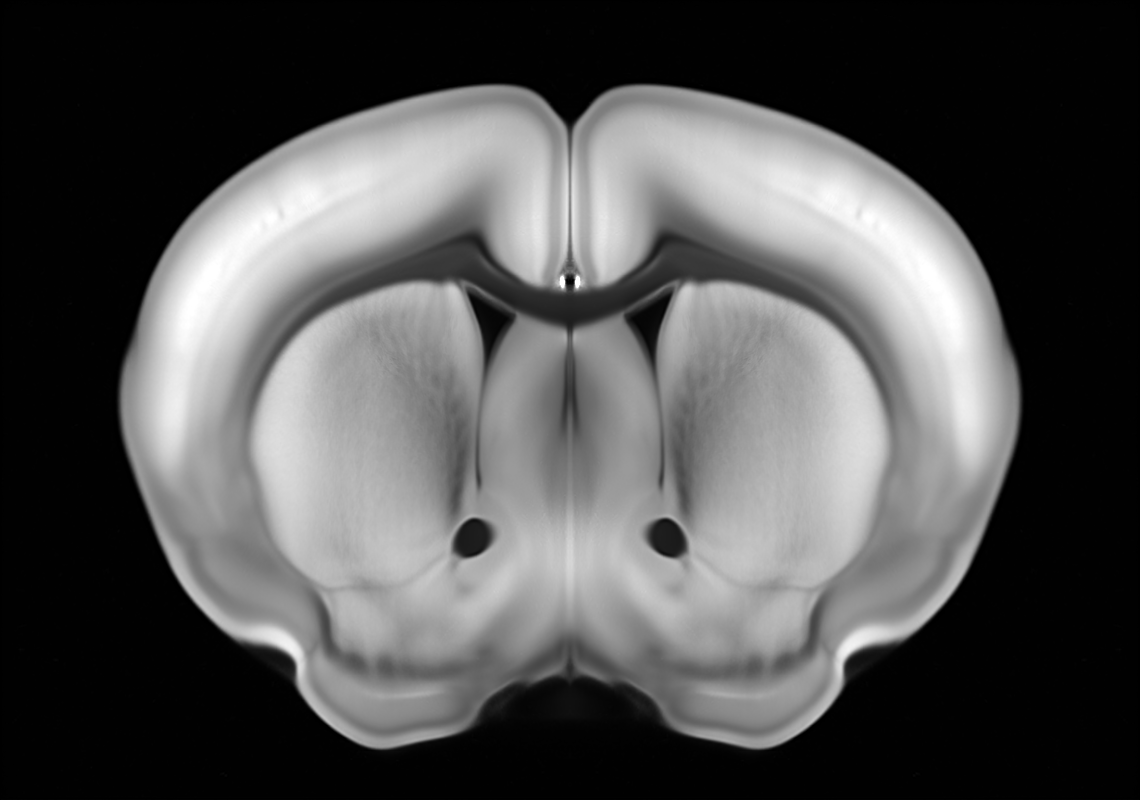

Supplement: Supplementary file 7 — Supplementary Data 5 [file 41467_2019_13057_MOESM7_ESM.zip › Suppl_File2_CCFbackground/AllenCCF_Z040.tif]

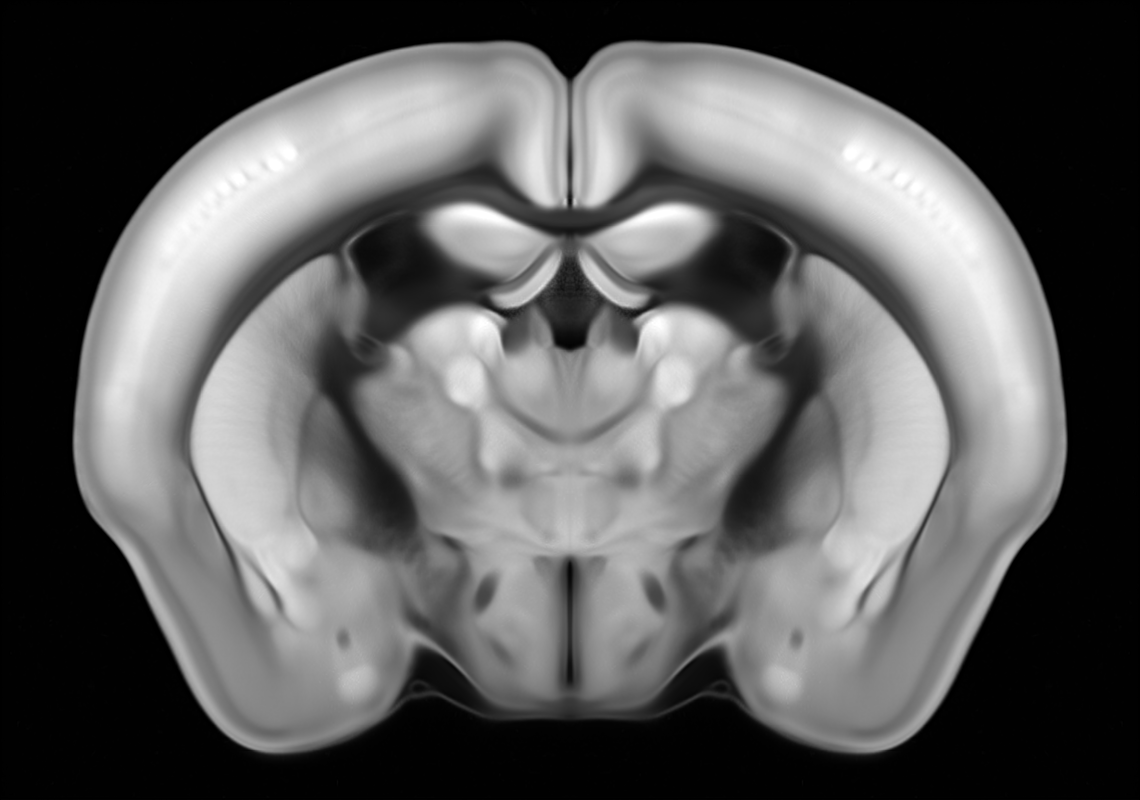

Supplement: Supplementary file 7 — Supplementary Data 5 [file 41467_2019_13057_MOESM7_ESM.zip › Suppl_File2_CCFbackground/AllenCCF_Z054.tif]

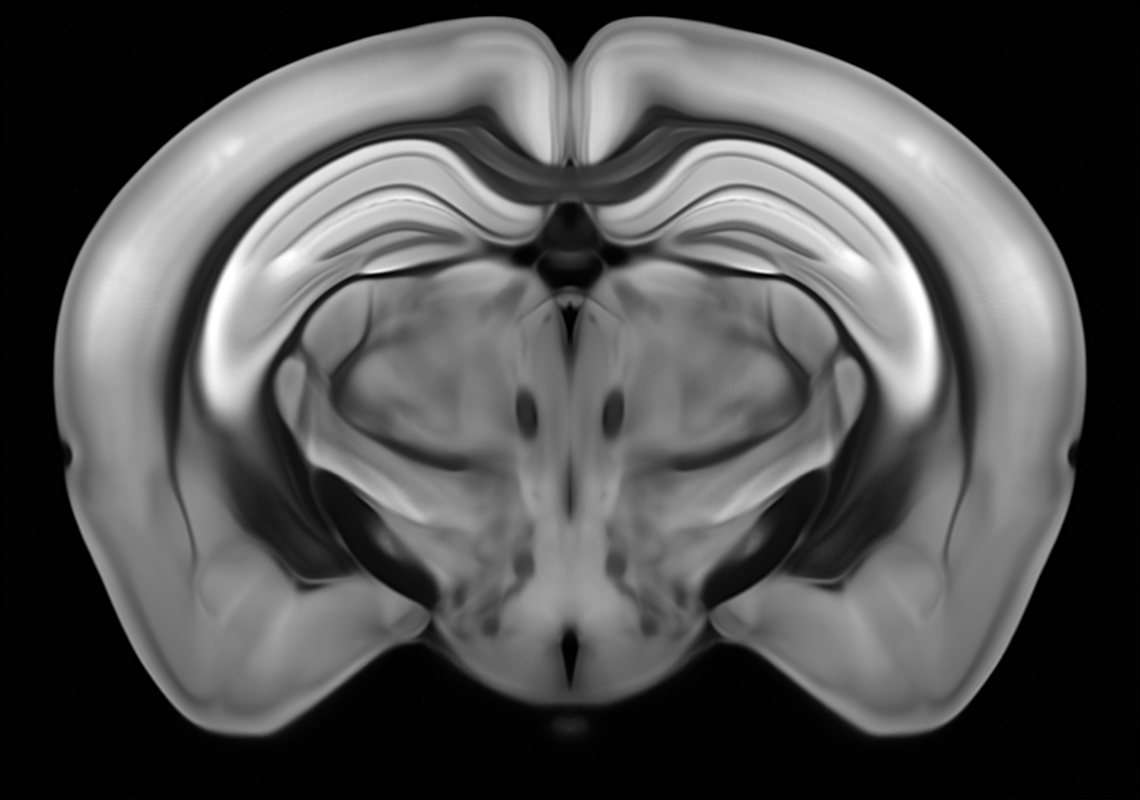

Supplement: Supplementary file 7 — Supplementary Data 5 [file 41467_2019_13057_MOESM7_ESM.zip › Suppl_File2_CCFbackground/AllenCCF_Z068.tif]

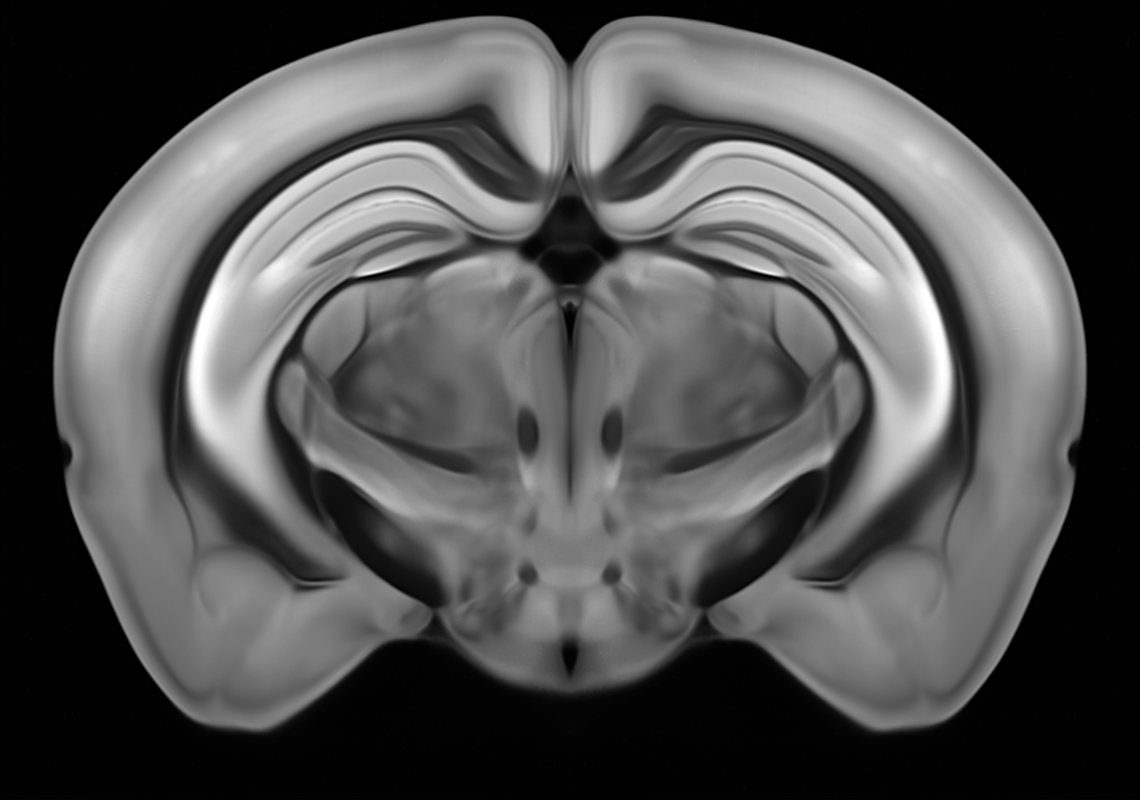

Supplement: Supplementary file 7 — Supplementary Data 5 [file 41467_2019_13057_MOESM7_ESM.zip › Suppl_File2_CCFbackground/AllenCCF_Z069.tif]

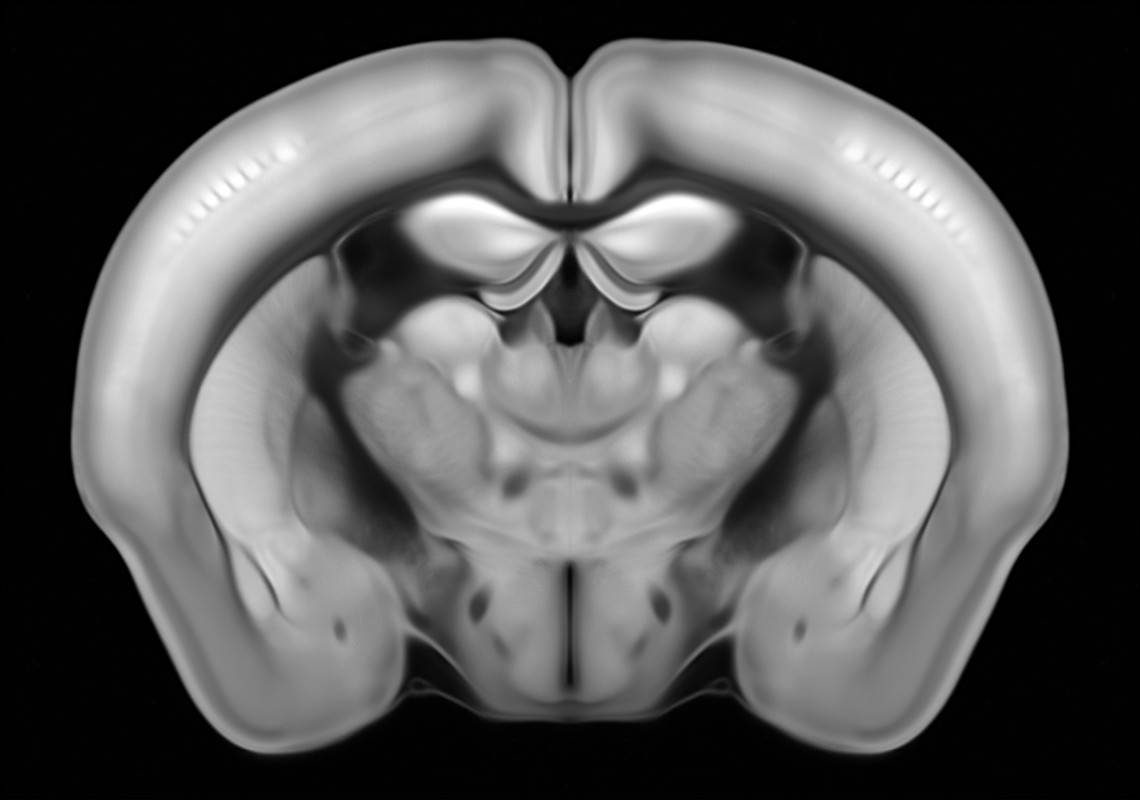

Supplement: Supplementary file 7 — Supplementary Data 5 [file 41467_2019_13057_MOESM7_ESM.zip › Suppl_File2_CCFbackground/AllenCCF_Z055.tif]

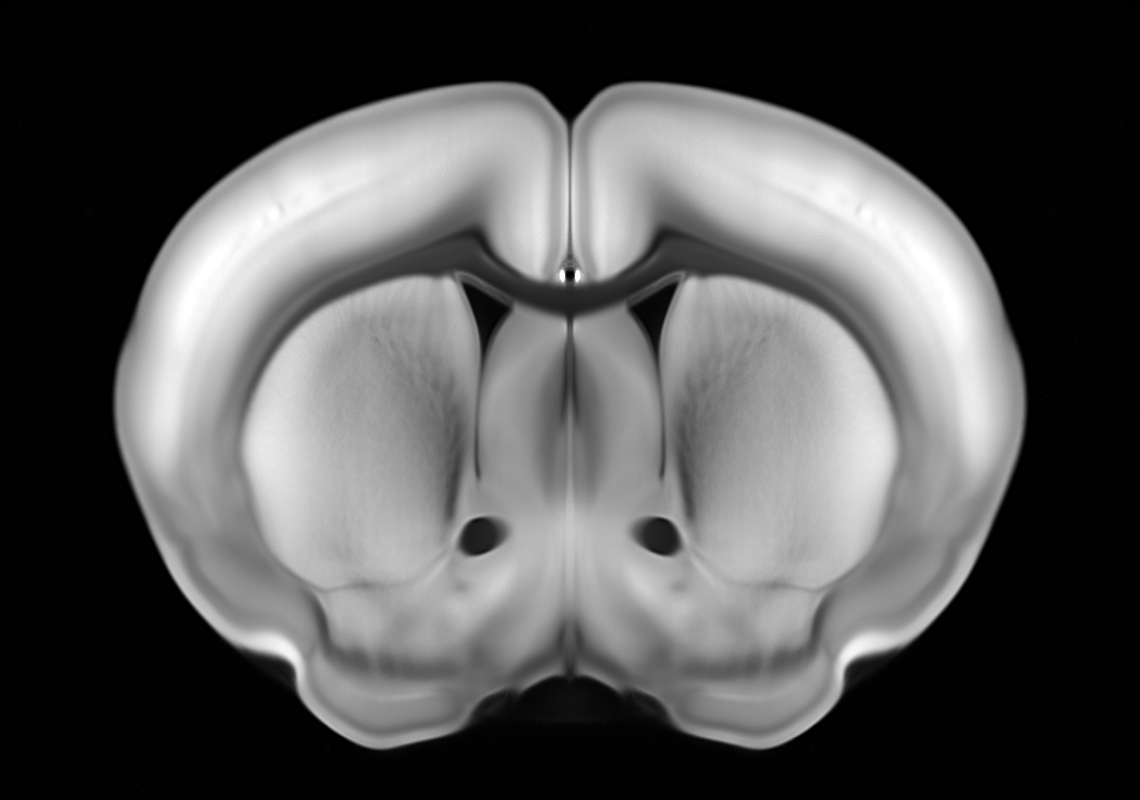

Supplement: Supplementary file 7 — Supplementary Data 5 [file 41467_2019_13057_MOESM7_ESM.zip › Suppl_File2_CCFbackground/AllenCCF_Z041.tif]

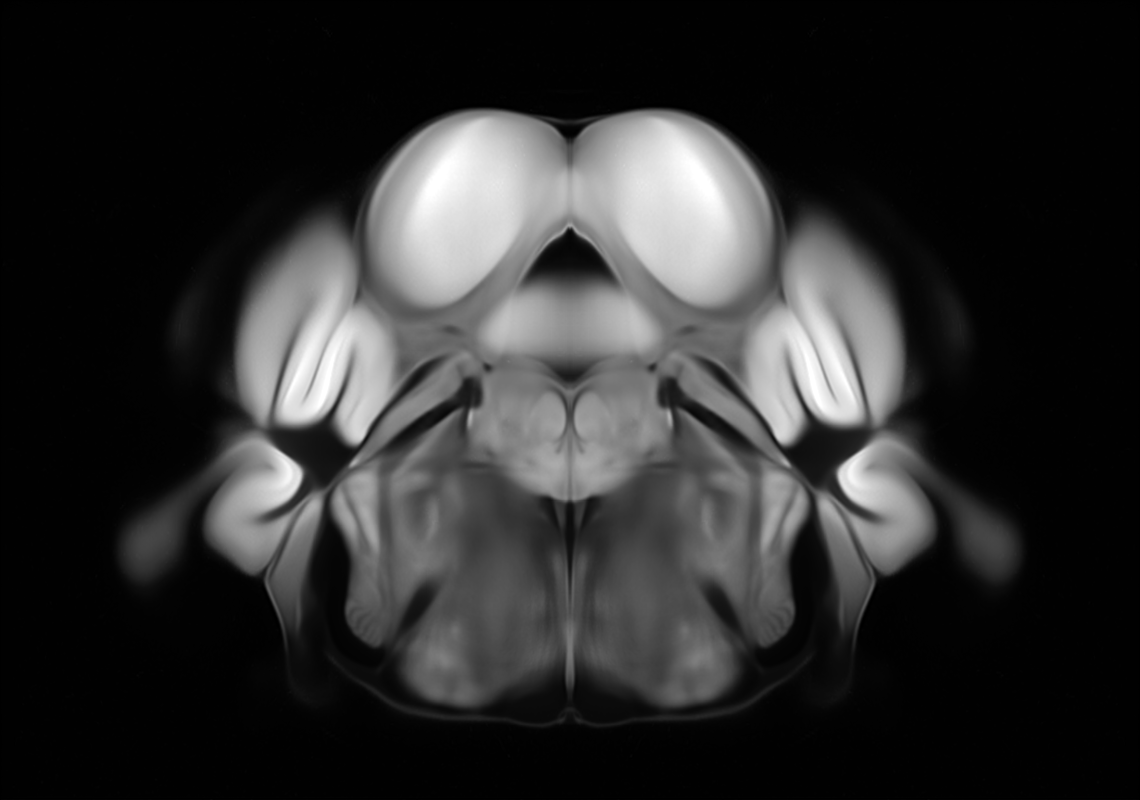

Supplement: Supplementary file 7 — Supplementary Data 5 [file 41467_2019_13057_MOESM7_ESM.zip › Suppl_File2_CCFbackground/AllenCCF_Z096.tif]

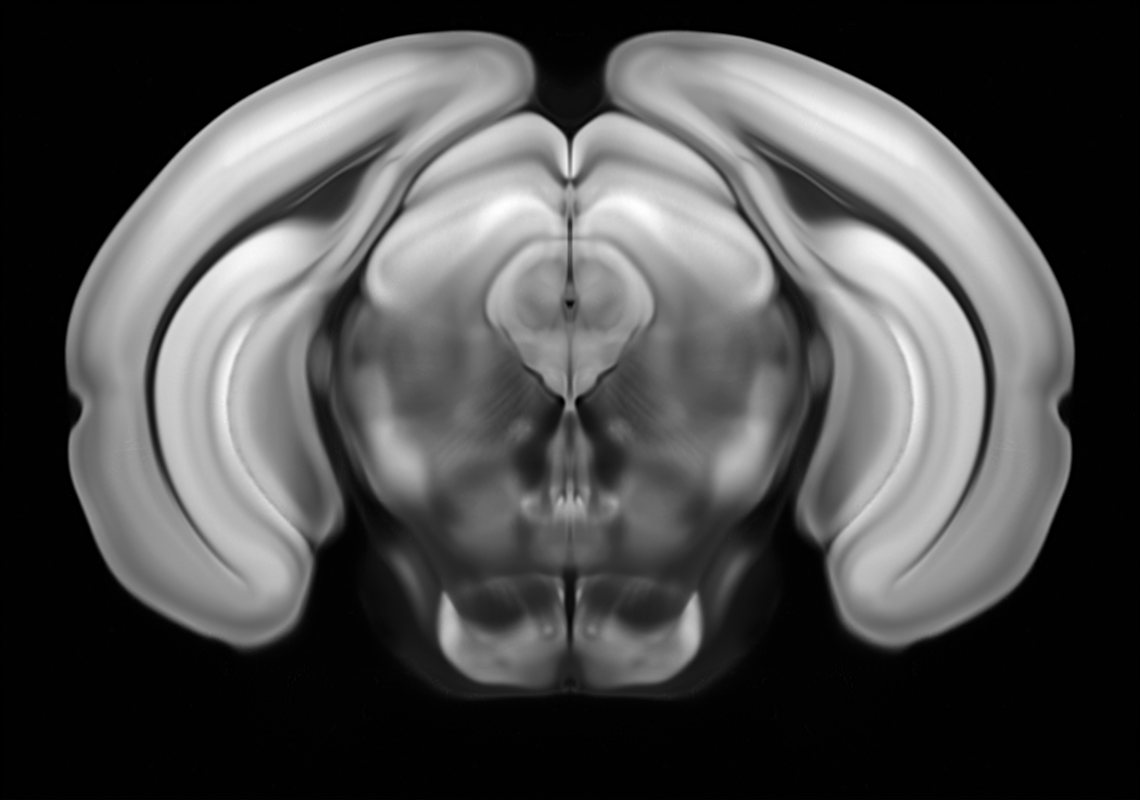

Supplement: Supplementary file 7 — Supplementary Data 5 [file 41467_2019_13057_MOESM7_ESM.zip › Suppl_File2_CCFbackground/AllenCCF_Z082.tif]

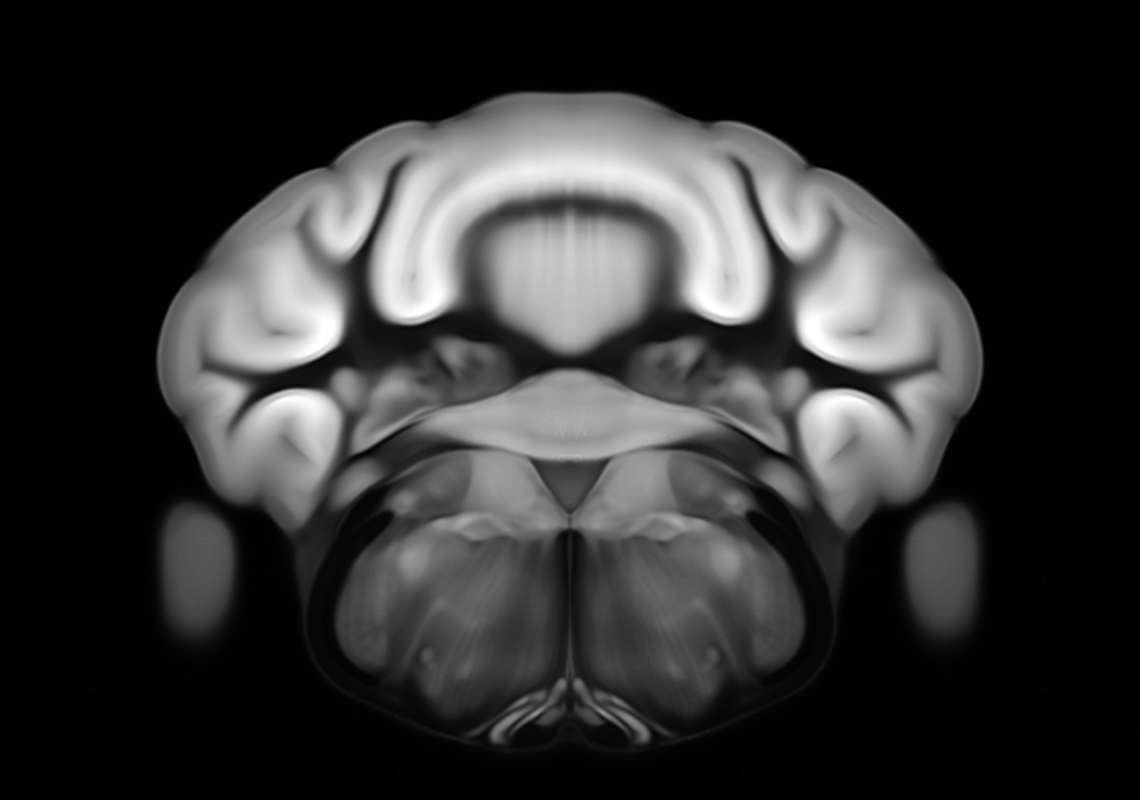

Supplement: Supplementary file 7 — Supplementary Data 5 [file 41467_2019_13057_MOESM7_ESM.zip › Suppl_File2_CCFbackground/AllenCCF_Z109.tif]

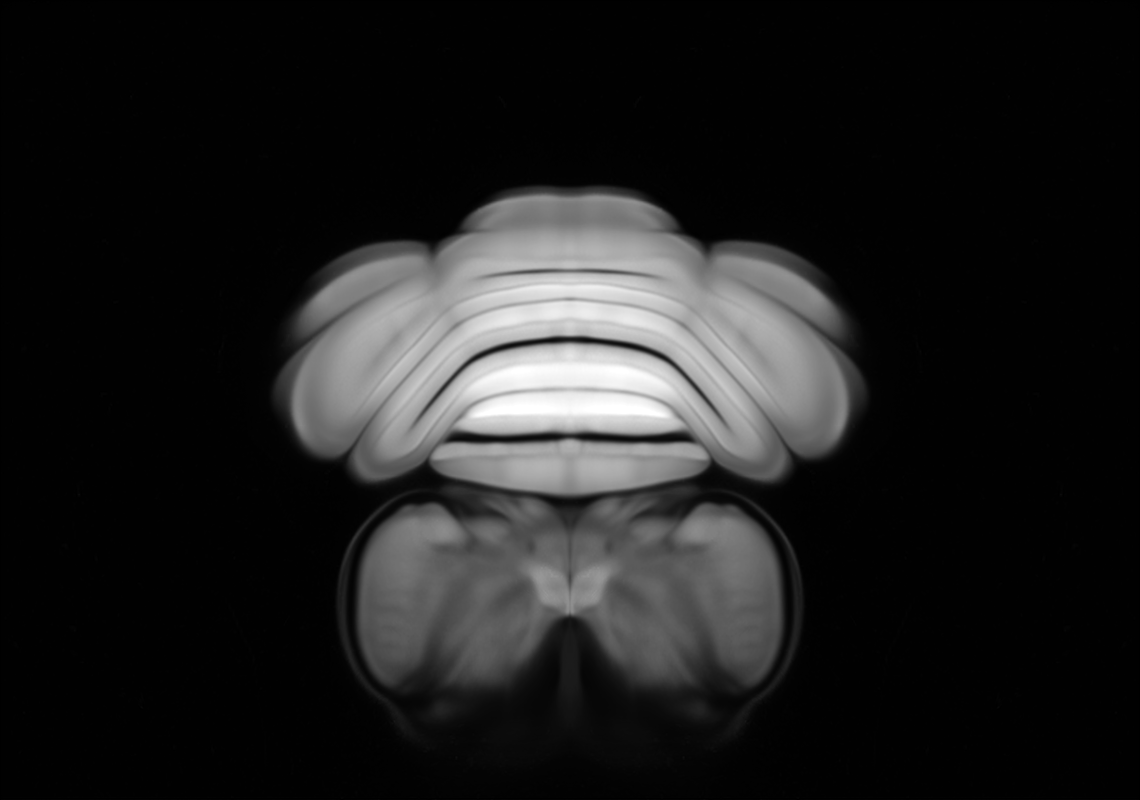

Supplement: Supplementary file 7 — Supplementary Data 5 [file 41467_2019_13057_MOESM7_ESM.zip › Suppl_File2_CCFbackground/AllenCCF_Z121.tif]

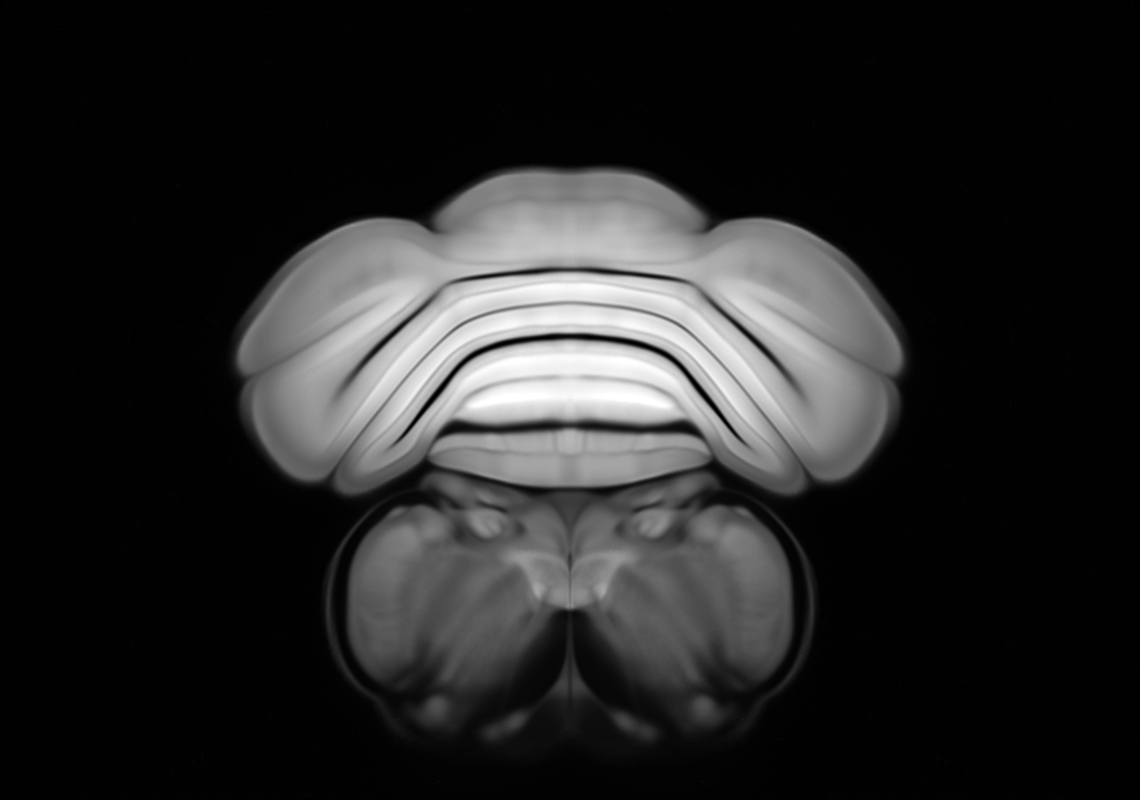

Supplement: Supplementary file 7 — Supplementary Data 5 [file 41467_2019_13057_MOESM7_ESM.zip › Suppl_File2_CCFbackground/AllenCCF_Z119.tif]

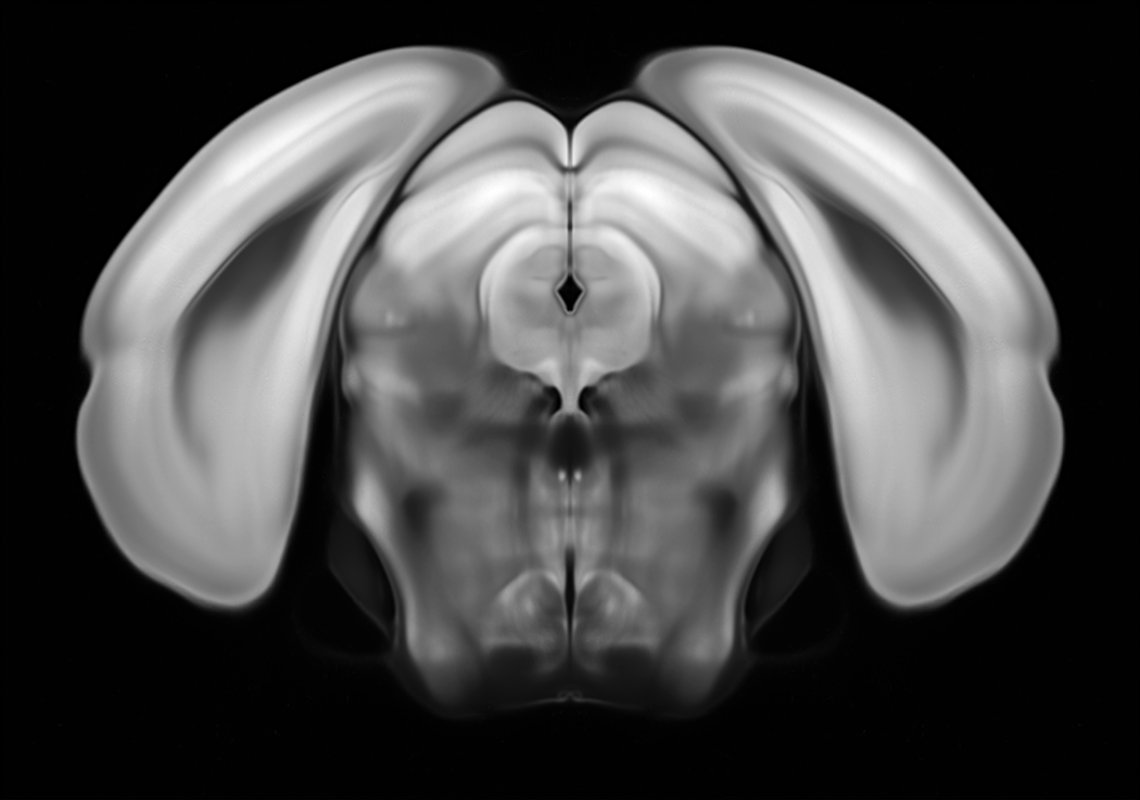

Supplement: Supplementary file 7 — Supplementary Data 5 [file 41467_2019_13057_MOESM7_ESM.zip › Suppl_File2_CCFbackground/AllenCCF_Z086.tif]

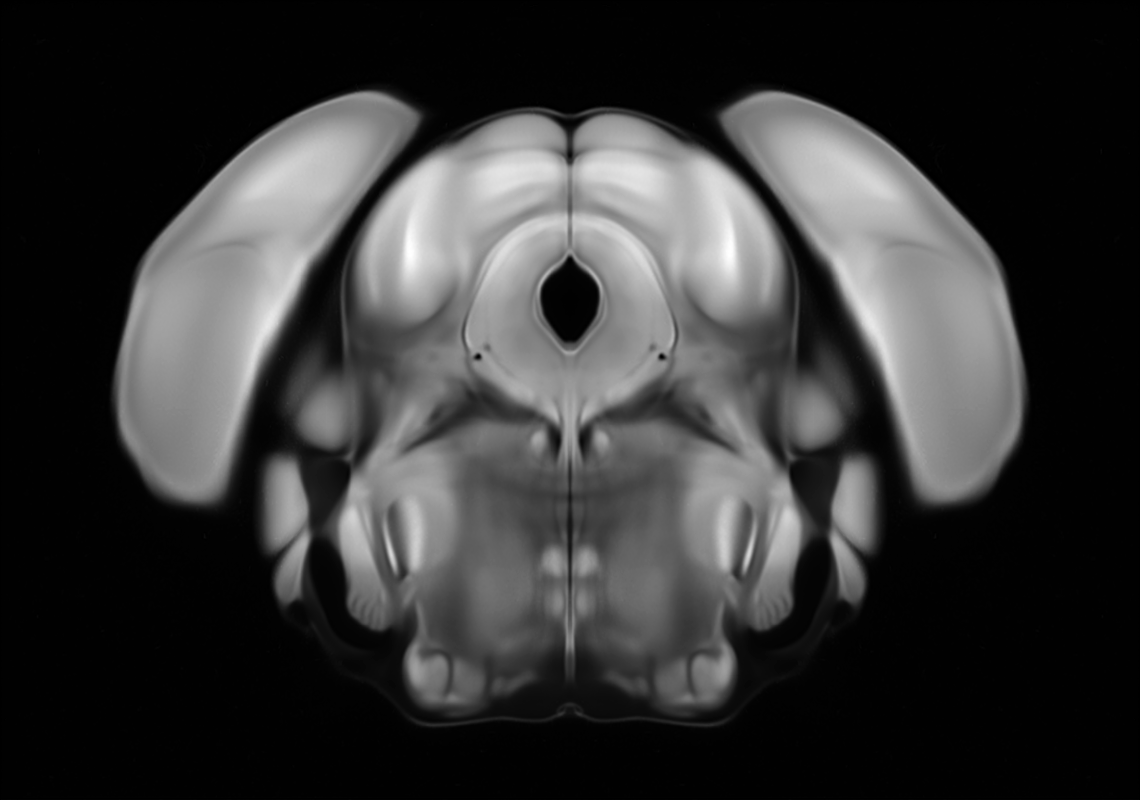

Supplement: Supplementary file 7 — Supplementary Data 5 [file 41467_2019_13057_MOESM7_ESM.zip › Suppl_File2_CCFbackground/AllenCCF_Z092.tif]

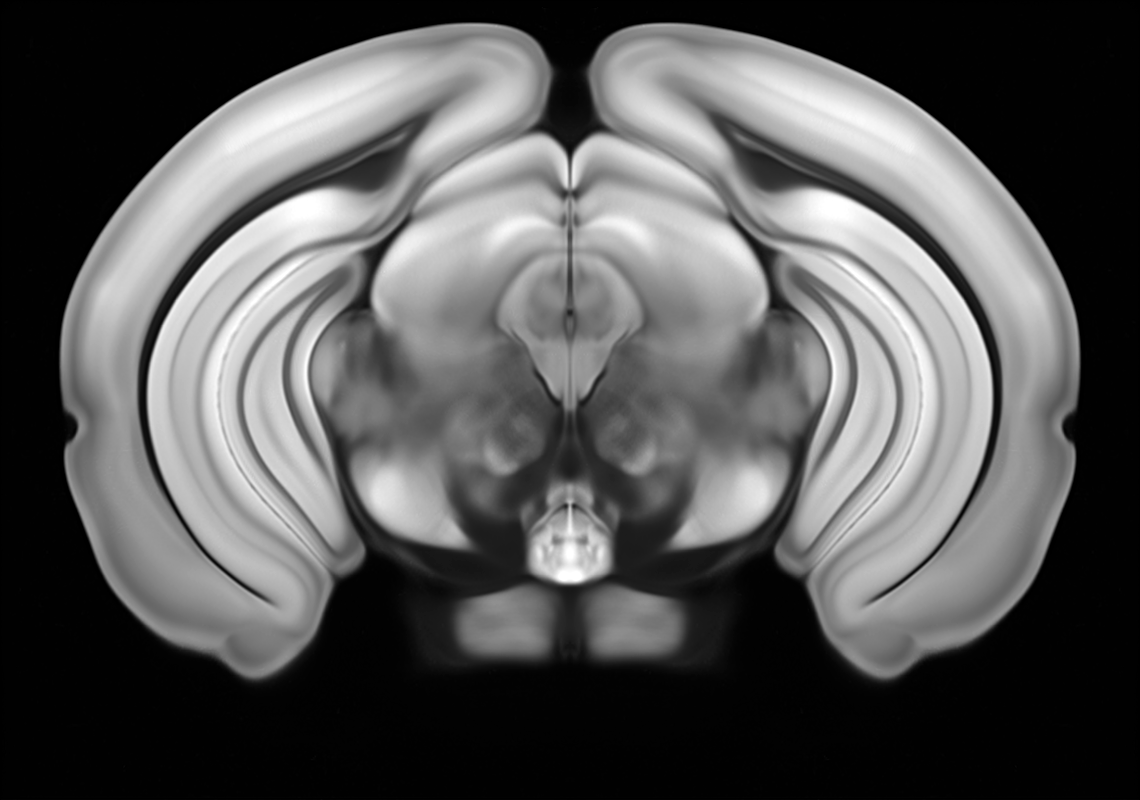

Supplement: Supplementary file 7 — Supplementary Data 5 [file 41467_2019_13057_MOESM7_ESM.zip › Suppl_File2_CCFbackground/AllenCCF_Z079.tif]

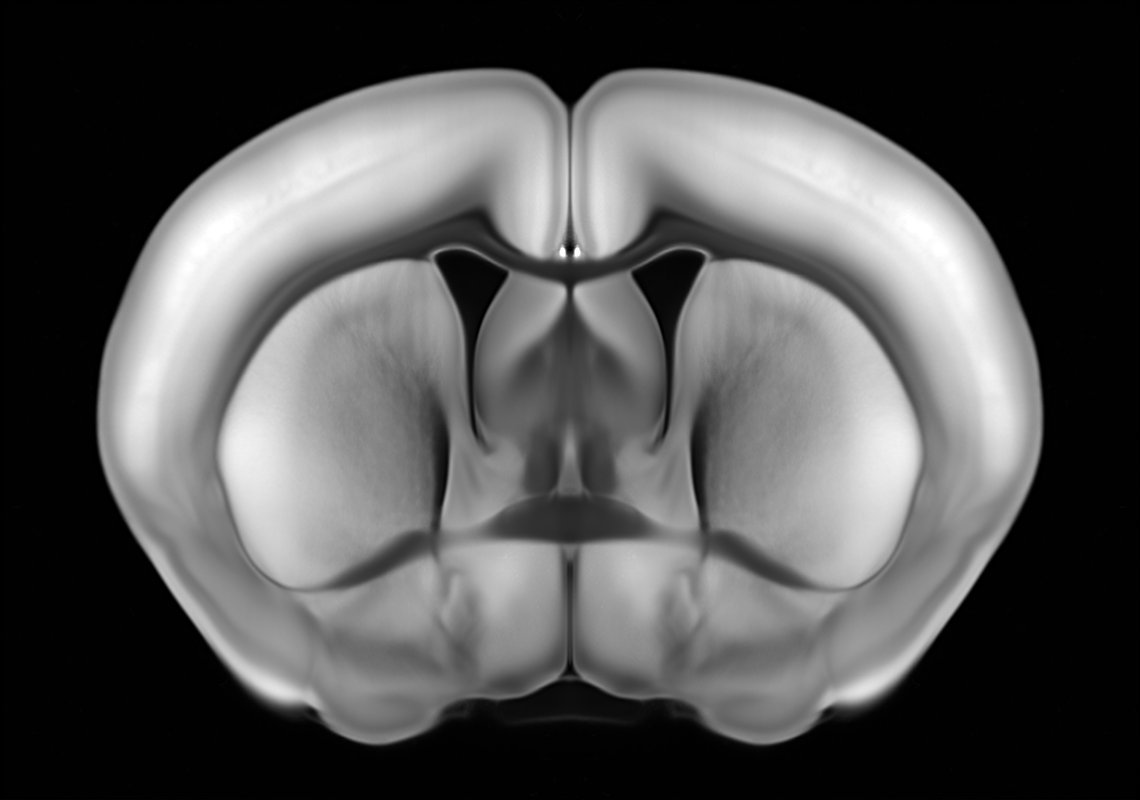

Supplement: Supplementary file 7 — Supplementary Data 5 [file 41467_2019_13057_MOESM7_ESM.zip › Suppl_File2_CCFbackground/AllenCCF_Z045.tif]

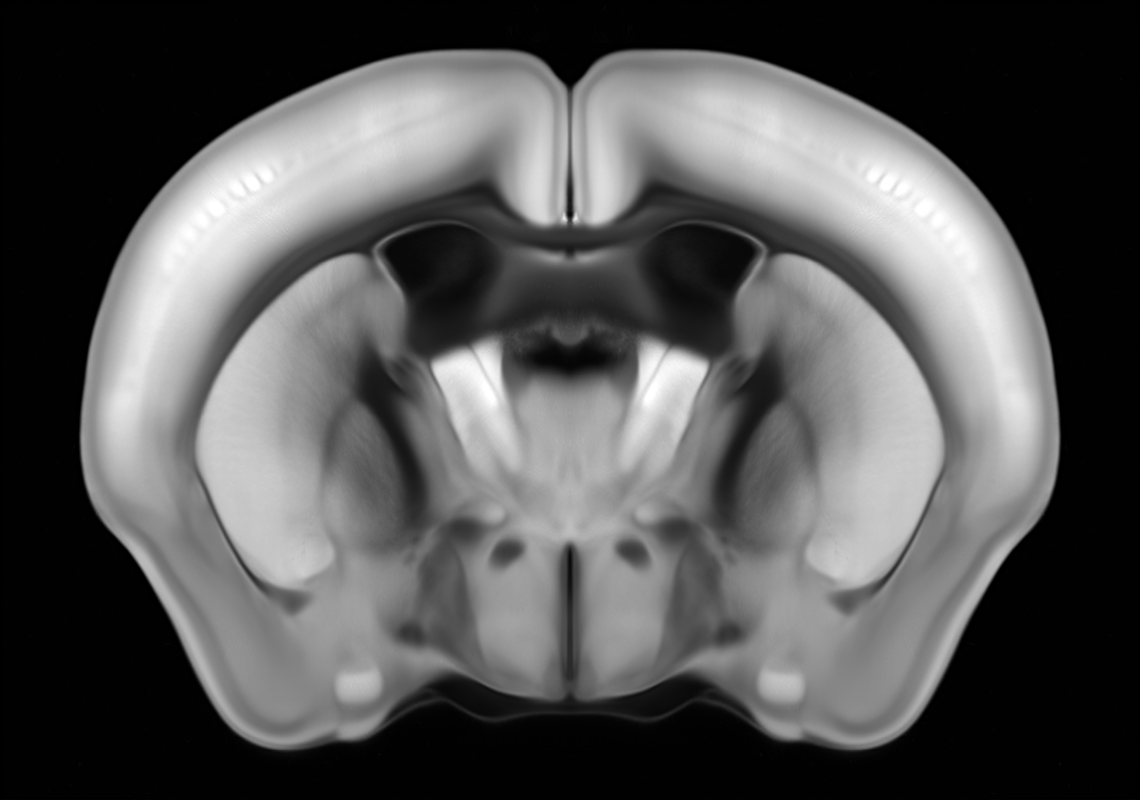

Supplement: Supplementary file 7 — Supplementary Data 5 [file 41467_2019_13057_MOESM7_ESM.zip › Suppl_File2_CCFbackground/AllenCCF_Z051.tif]

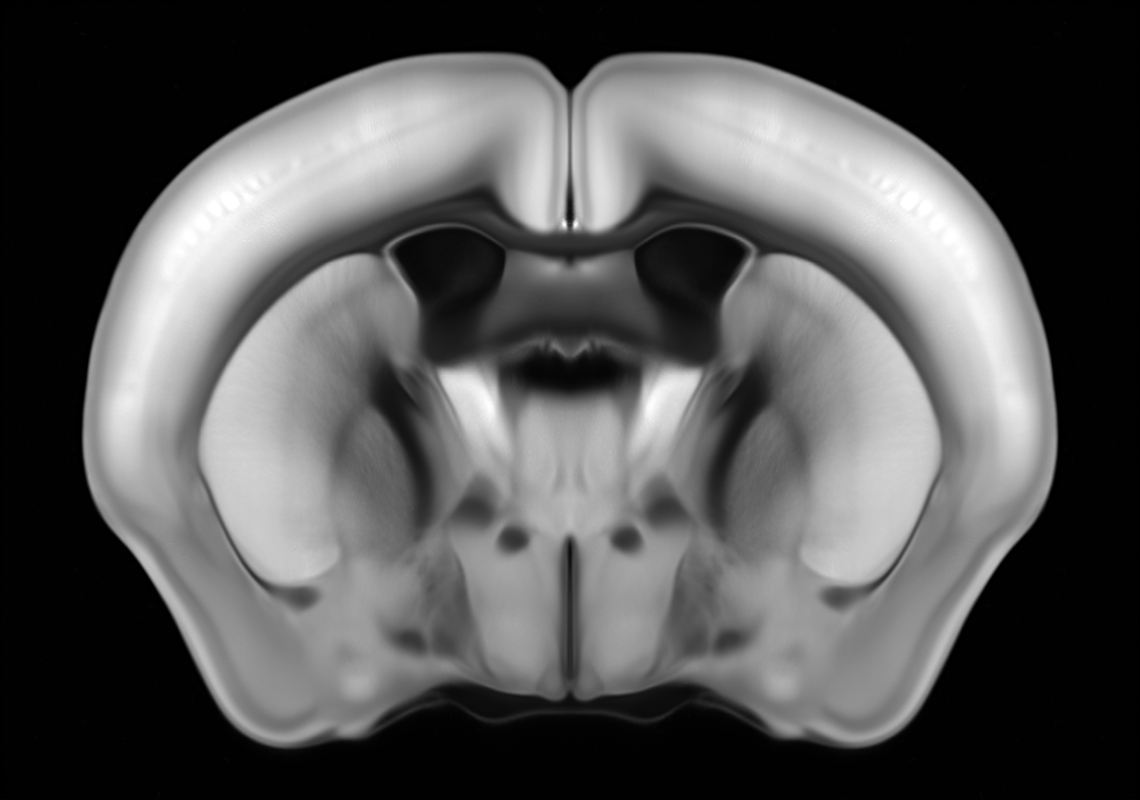

Supplement: Supplementary file 7 — Supplementary Data 5 [file 41467_2019_13057_MOESM7_ESM.zip › Suppl_File2_CCFbackground/AllenCCF_Z050.tif]

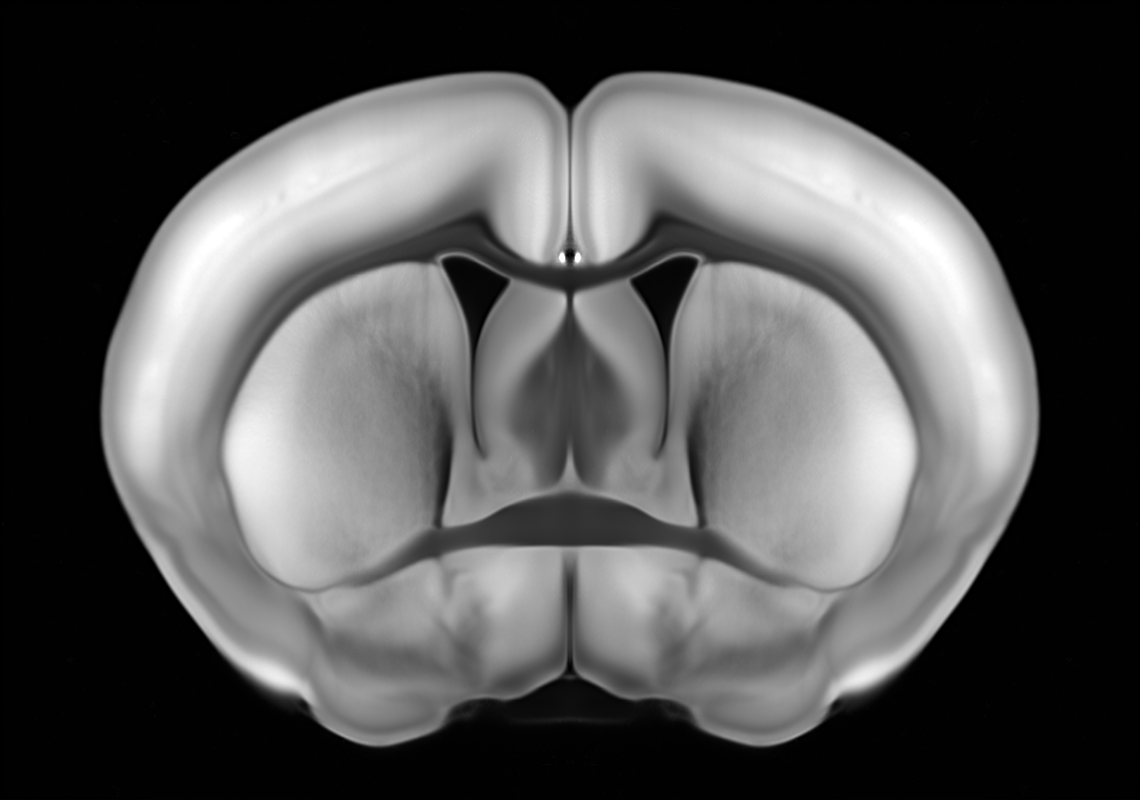

Supplement: Supplementary file 7 — Supplementary Data 5 [file 41467_2019_13057_MOESM7_ESM.zip › Suppl_File2_CCFbackground/AllenCCF_Z044.tif]

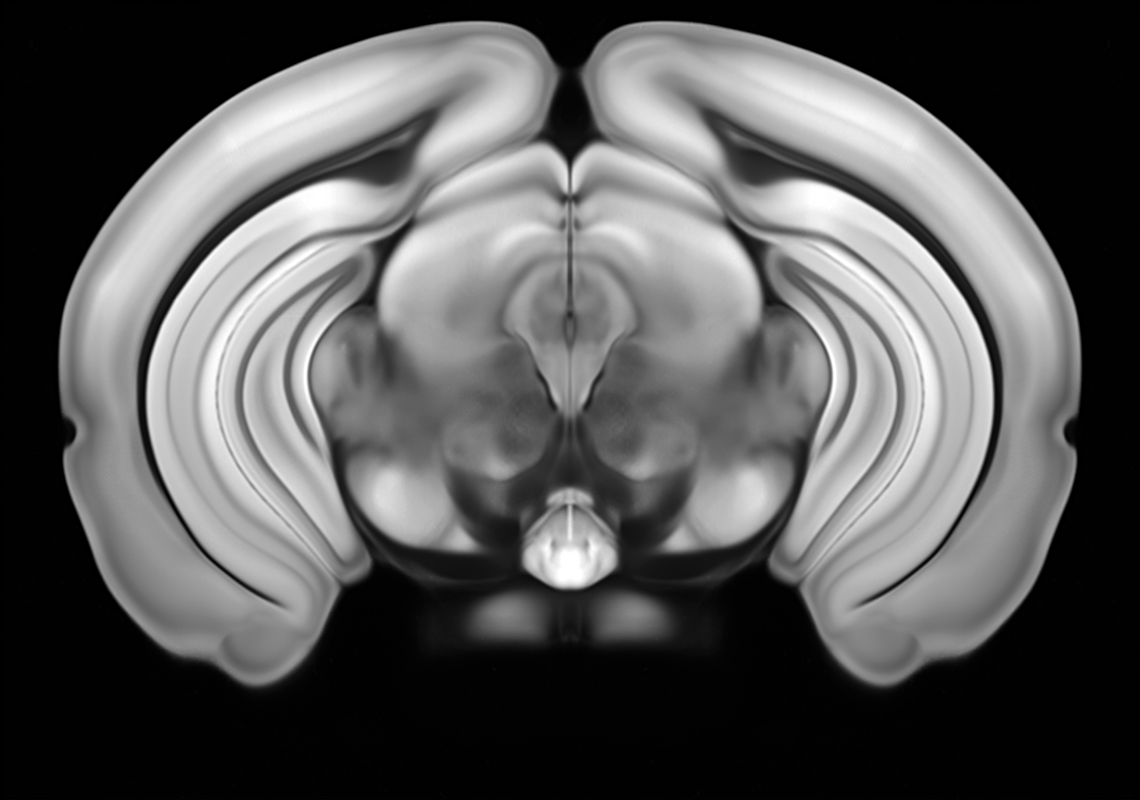

Supplement: Supplementary file 7 — Supplementary Data 5 [file 41467_2019_13057_MOESM7_ESM.zip › Suppl_File2_CCFbackground/AllenCCF_Z078.tif]

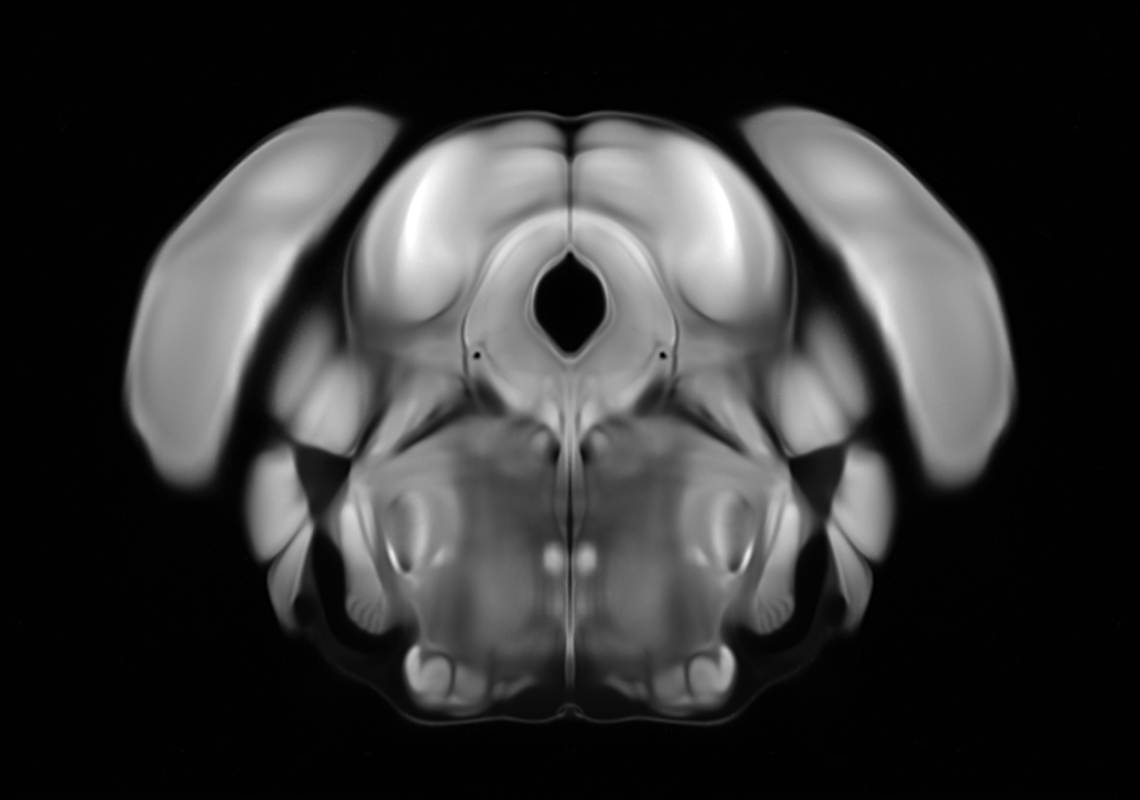

Supplement: Supplementary file 7 — Supplementary Data 5 [file 41467_2019_13057_MOESM7_ESM.zip › Suppl_File2_CCFbackground/AllenCCF_Z093.tif]

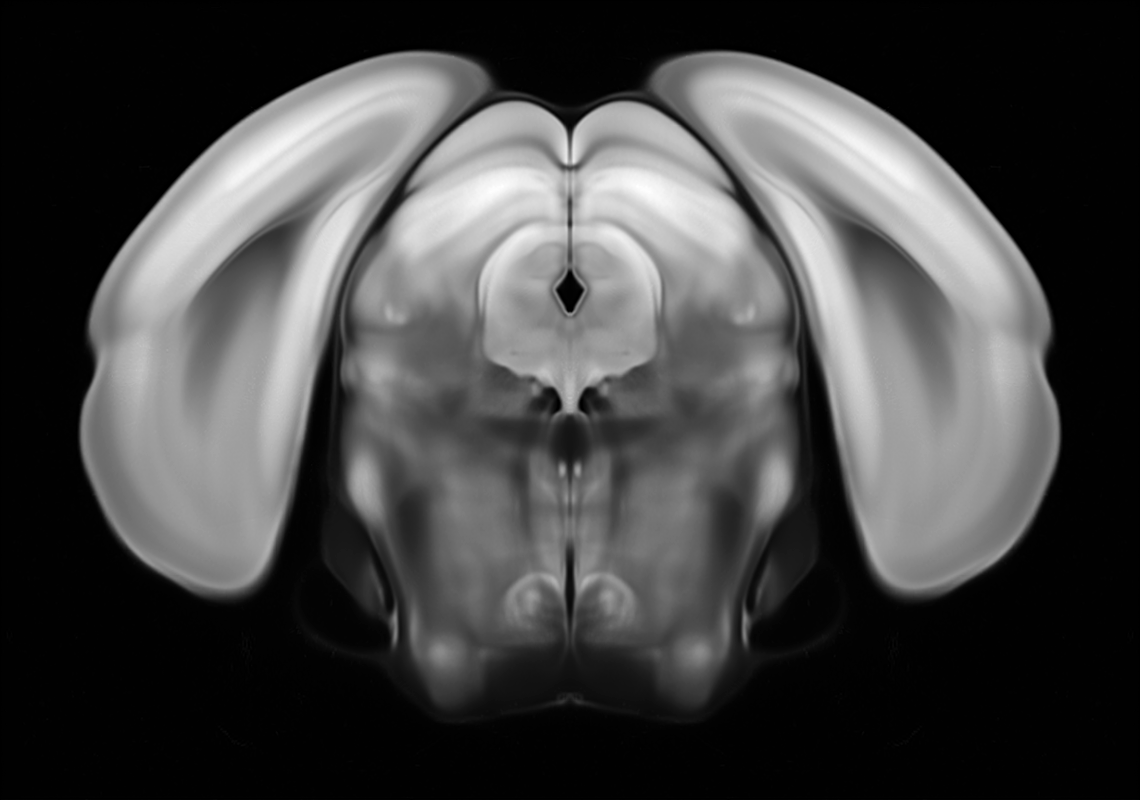

Supplement: Supplementary file 7 — Supplementary Data 5 [file 41467_2019_13057_MOESM7_ESM.zip › Suppl_File2_CCFbackground/AllenCCF_Z087.tif]

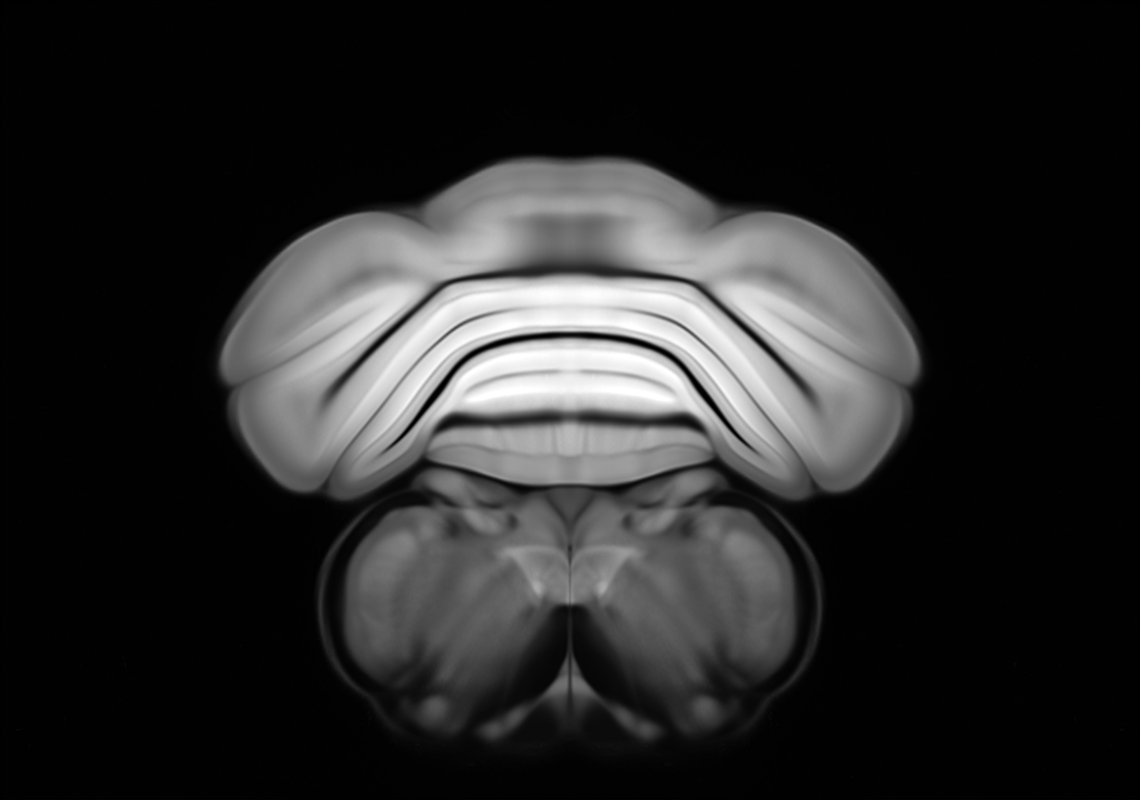

Supplement: Supplementary file 7 — Supplementary Data 5 [file 41467_2019_13057_MOESM7_ESM.zip › Suppl_File2_CCFbackground/AllenCCF_Z118.tif]

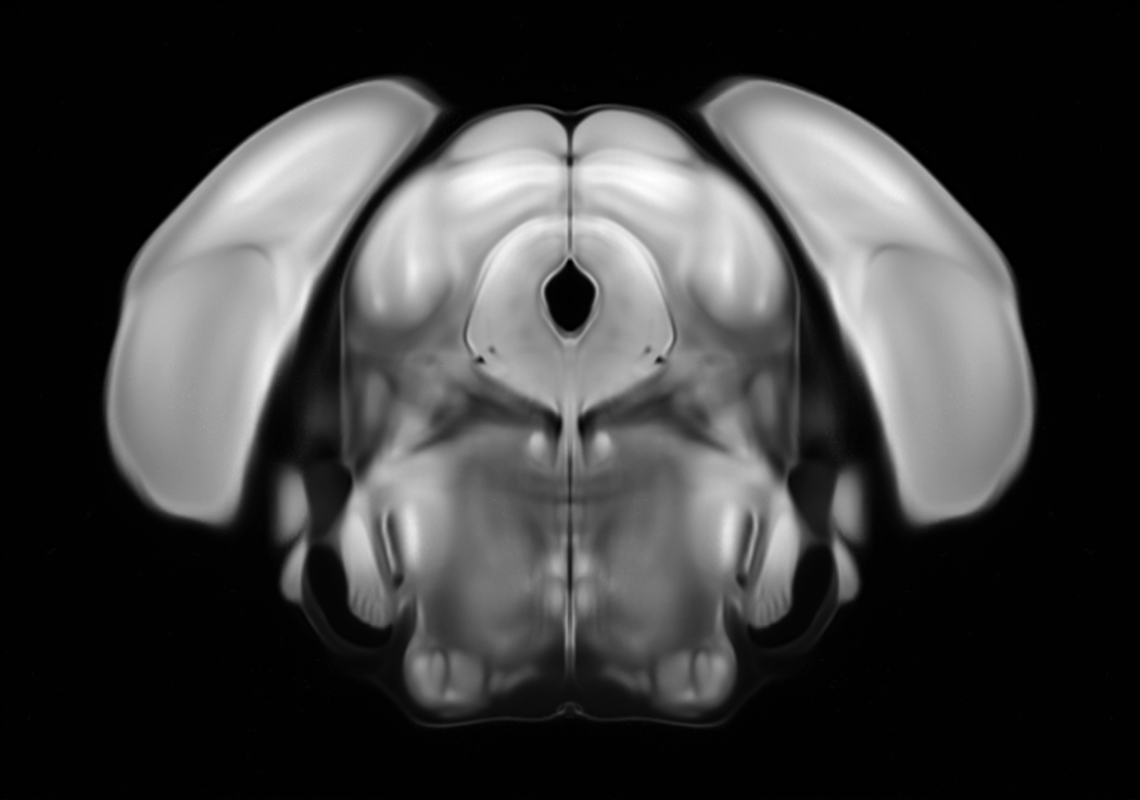

Supplement: Supplementary file 7 — Supplementary Data 5 [file 41467_2019_13057_MOESM7_ESM.zip › Suppl_File2_CCFbackground/AllenCCF_Z091.tif]

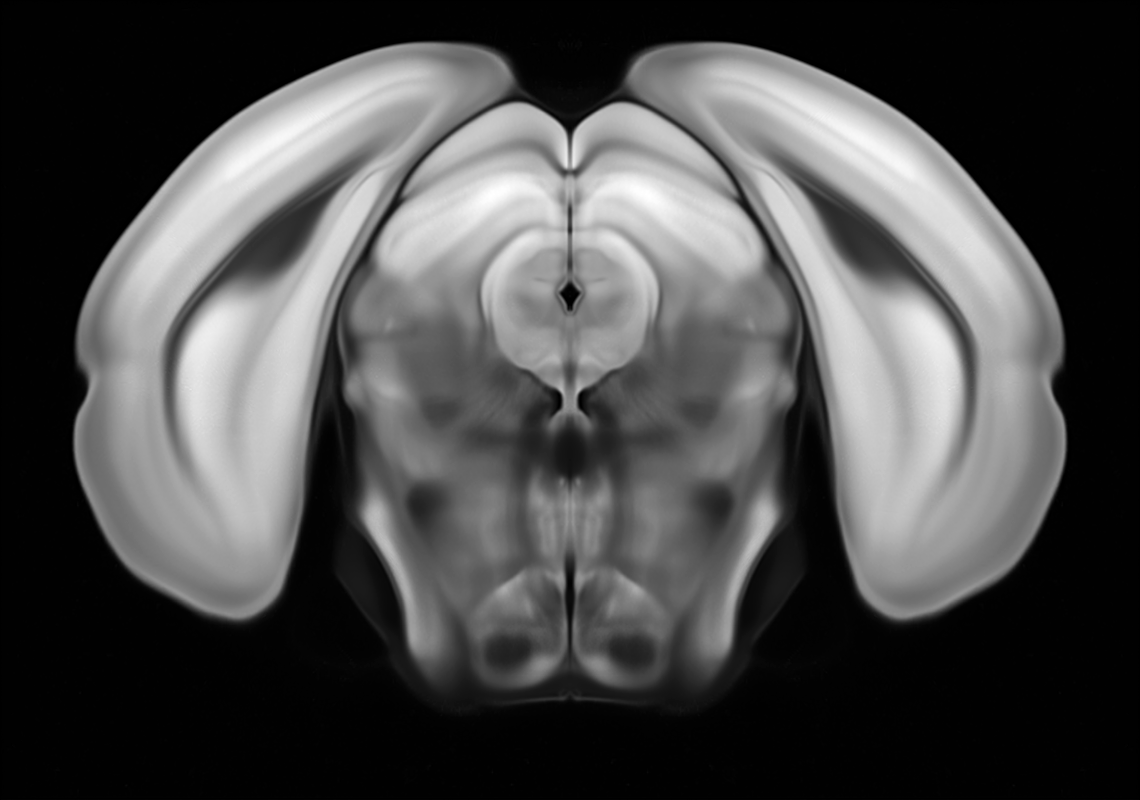

Supplement: Supplementary file 7 — Supplementary Data 5 [file 41467_2019_13057_MOESM7_ESM.zip › Suppl_File2_CCFbackground/AllenCCF_Z085.tif]

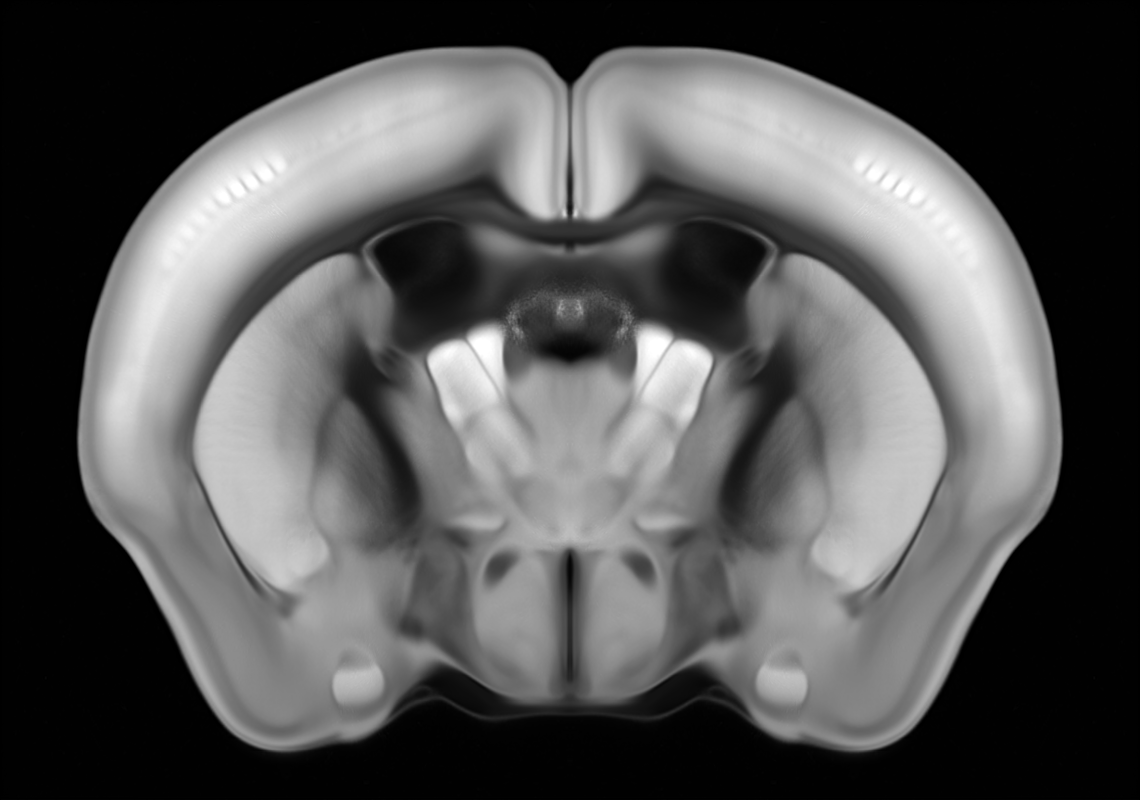

Supplement: Supplementary file 7 — Supplementary Data 5 [file 41467_2019_13057_MOESM7_ESM.zip › Suppl_File2_CCFbackground/AllenCCF_Z052.tif]

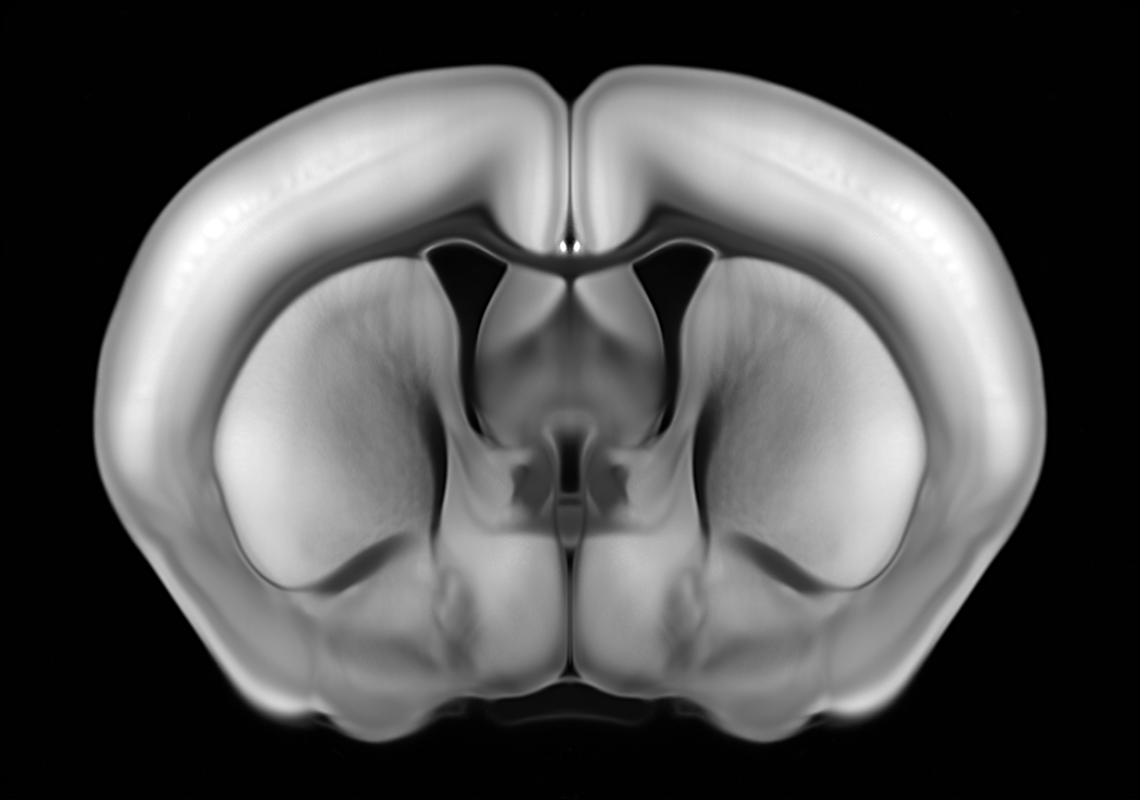

Supplement: Supplementary file 7 — Supplementary Data 5 [file 41467_2019_13057_MOESM7_ESM.zip › Suppl_File2_CCFbackground/AllenCCF_Z046.tif]

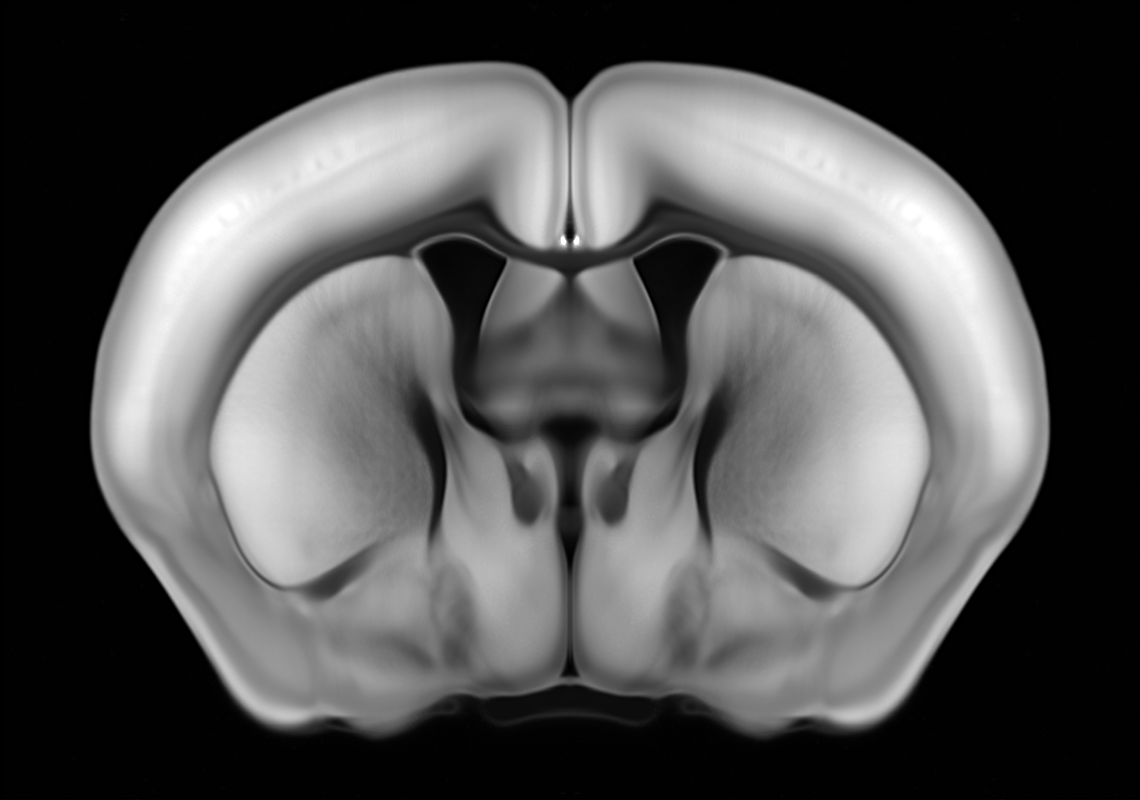

Supplement: Supplementary file 7 — Supplementary Data 5 [file 41467_2019_13057_MOESM7_ESM.zip › Suppl_File2_CCFbackground/AllenCCF_Z047.tif]

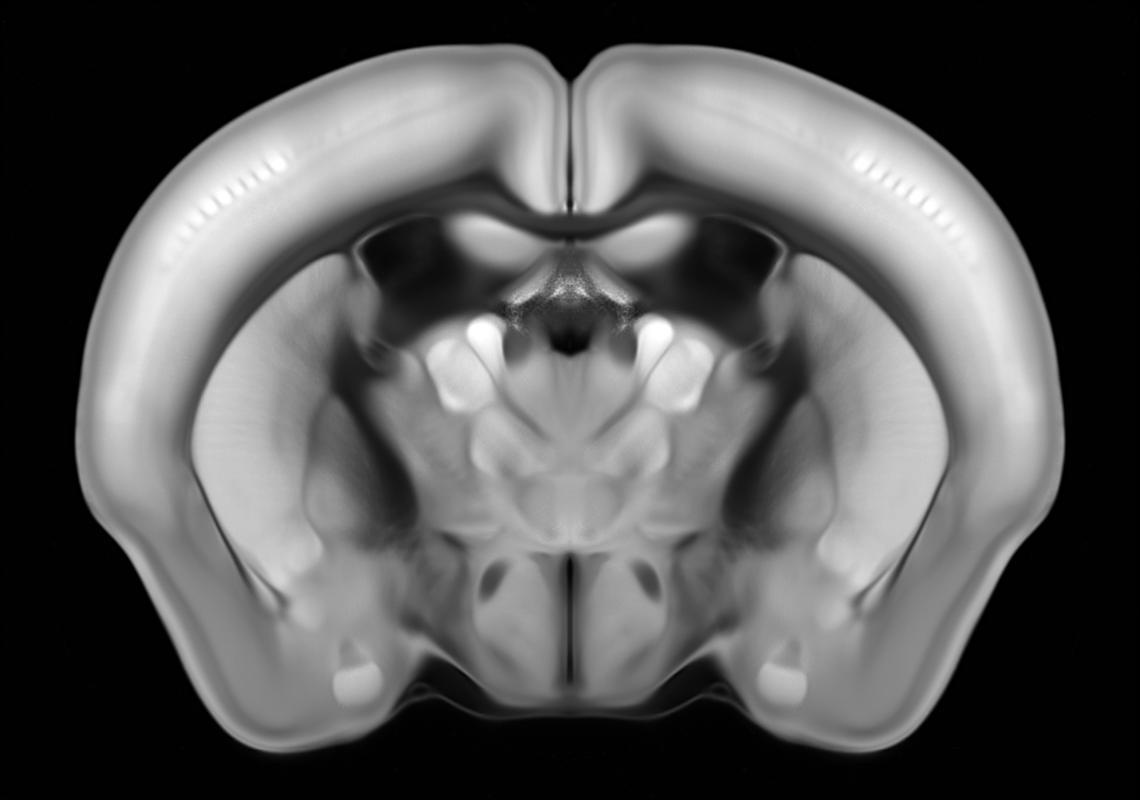

Supplement: Supplementary file 7 — Supplementary Data 5 [file 41467_2019_13057_MOESM7_ESM.zip › Suppl_File2_CCFbackground/AllenCCF_Z053.tif]

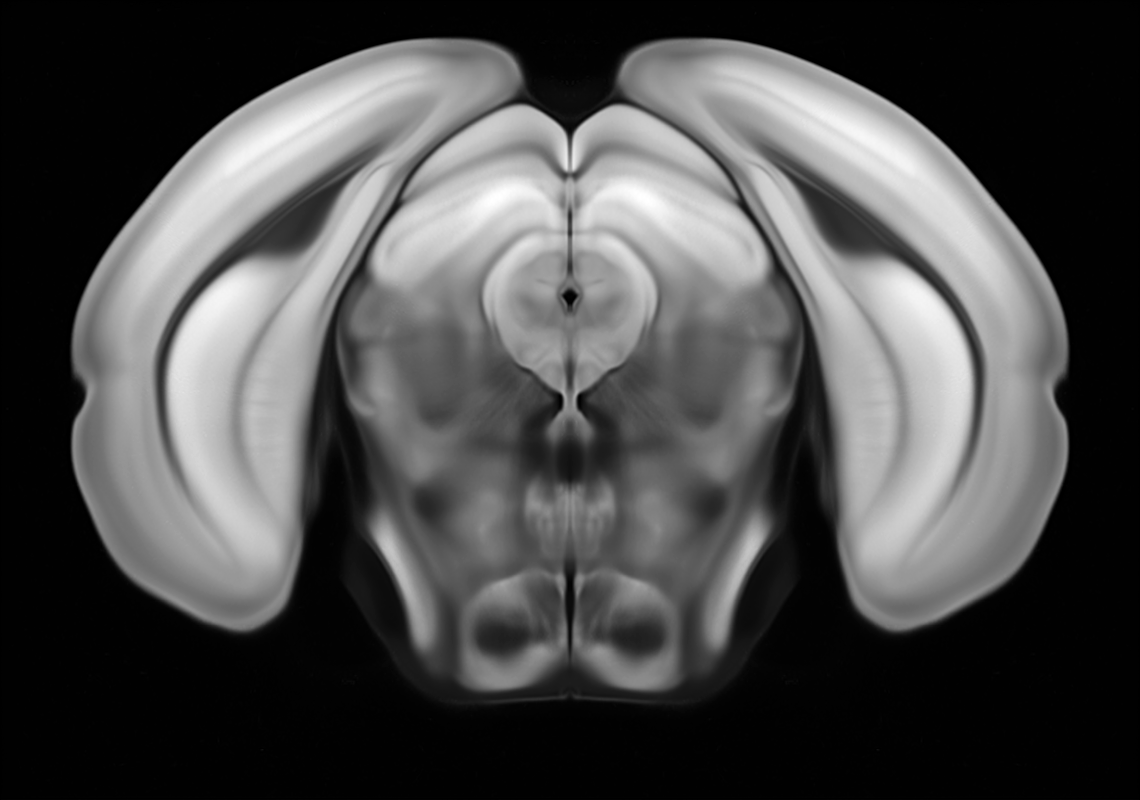

Supplement: Supplementary file 7 — Supplementary Data 5 [file 41467_2019_13057_MOESM7_ESM.zip › Suppl_File2_CCFbackground/AllenCCF_Z084.tif]

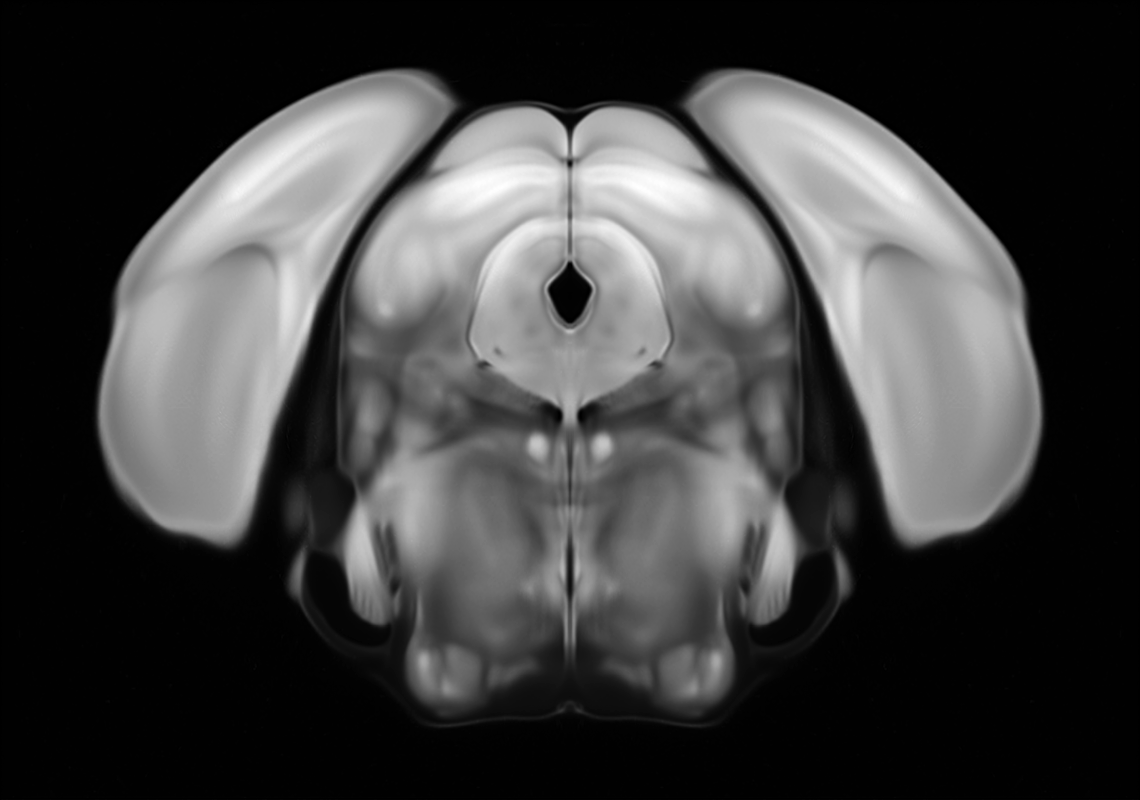

Supplement: Supplementary file 7 — Supplementary Data 5 [file 41467_2019_13057_MOESM7_ESM.zip › Suppl_File2_CCFbackground/AllenCCF_Z090.tif]

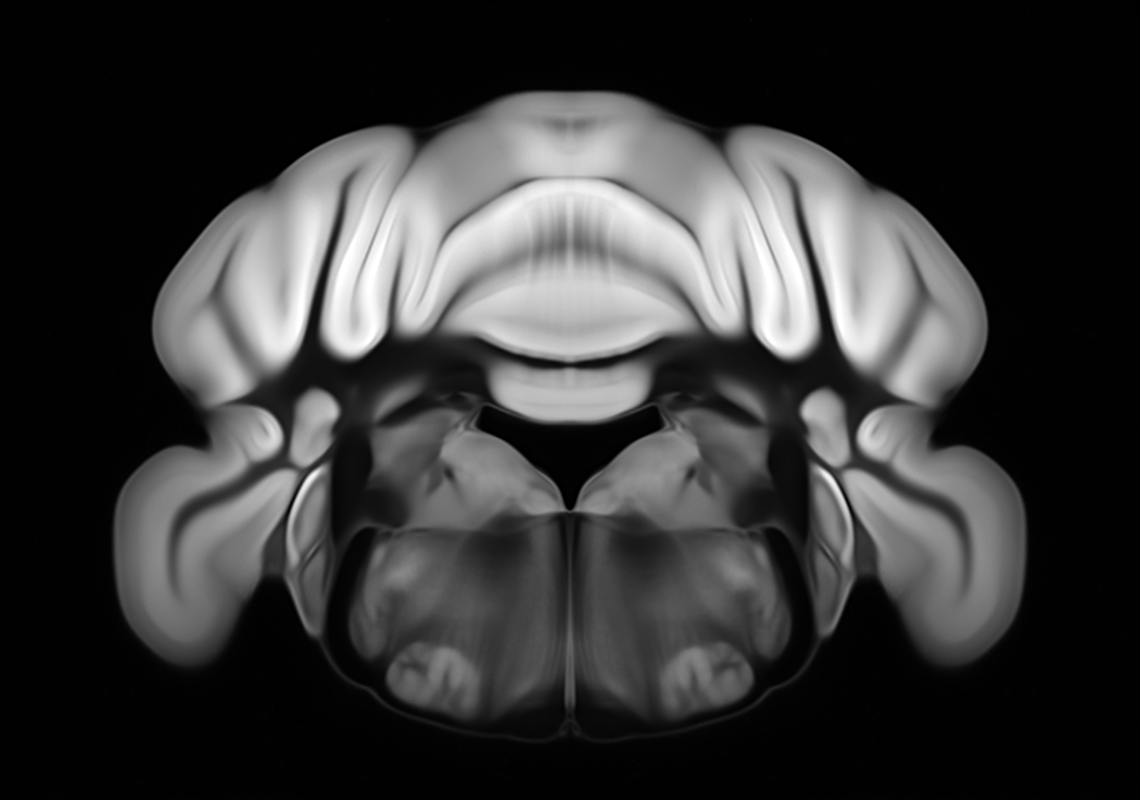

Supplement: Supplementary file 7 — Supplementary Data 5 [file 41467_2019_13057_MOESM7_ESM.zip › Suppl_File2_CCFbackground/AllenCCF_Z102.tif]

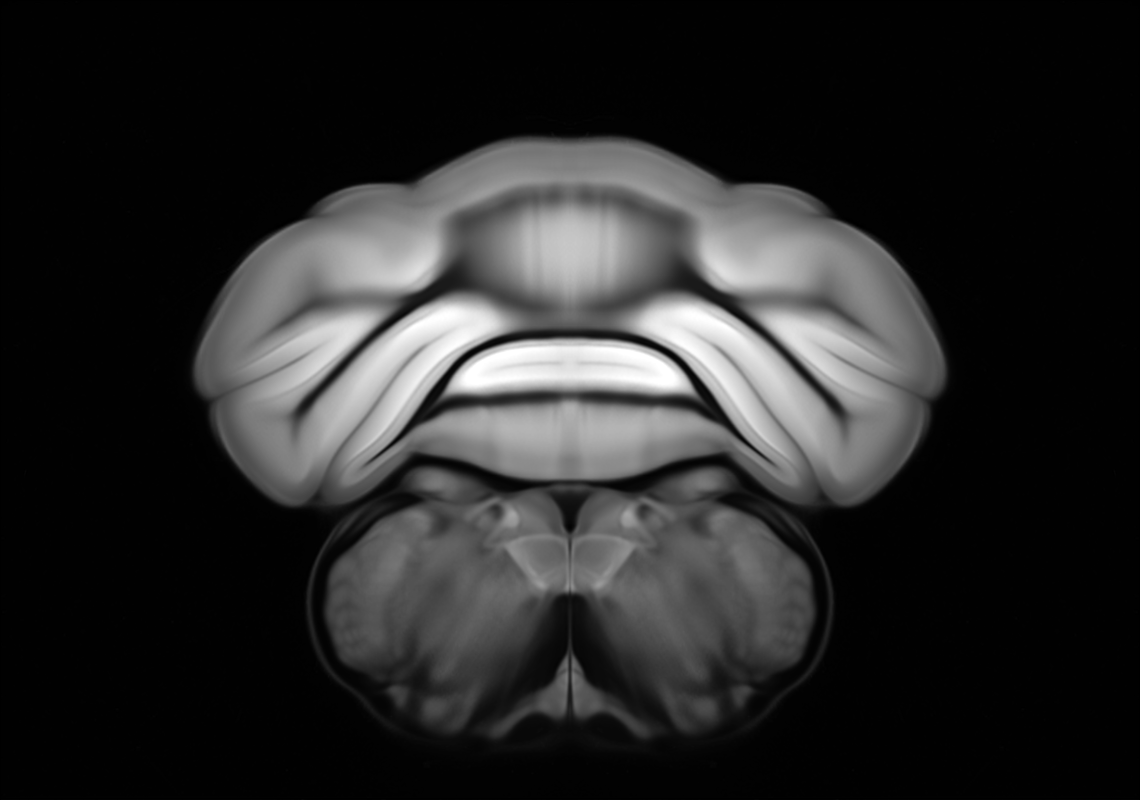

Supplement: Supplementary file 7 — Supplementary Data 5 [file 41467_2019_13057_MOESM7_ESM.zip › Suppl_File2_CCFbackground/AllenCCF_Z116.tif]

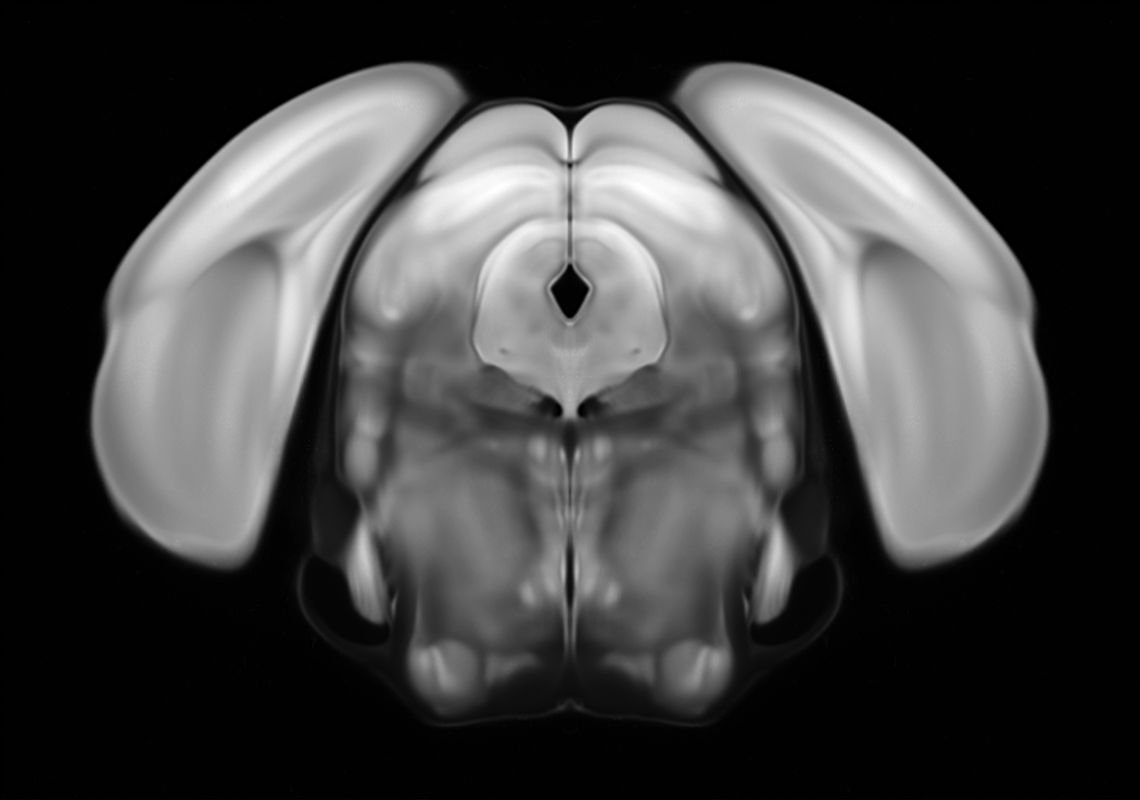

Supplement: Supplementary file 7 — Supplementary Data 5 [file 41467_2019_13057_MOESM7_ESM.zip › Suppl_File2_CCFbackground/AllenCCF_Z089.tif]

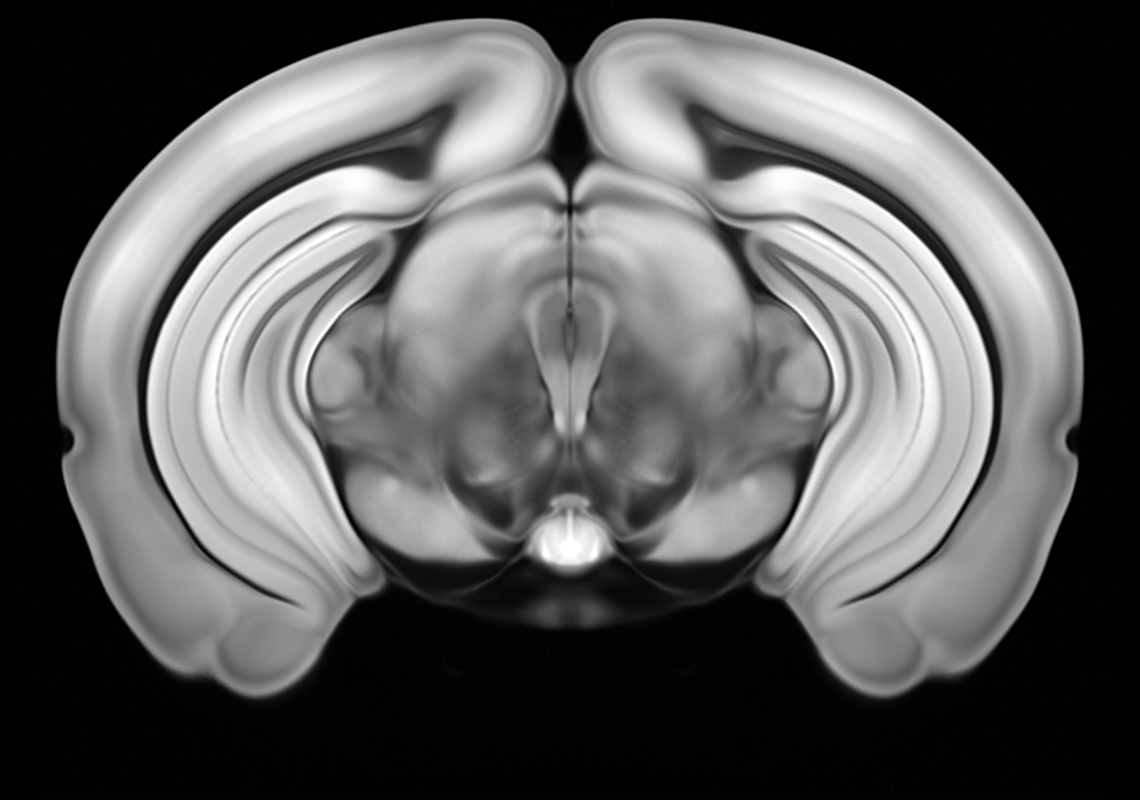

Supplement: Supplementary file 7 — Supplementary Data 5 [file 41467_2019_13057_MOESM7_ESM.zip › Suppl_File2_CCFbackground/AllenCCF_Z076.tif]

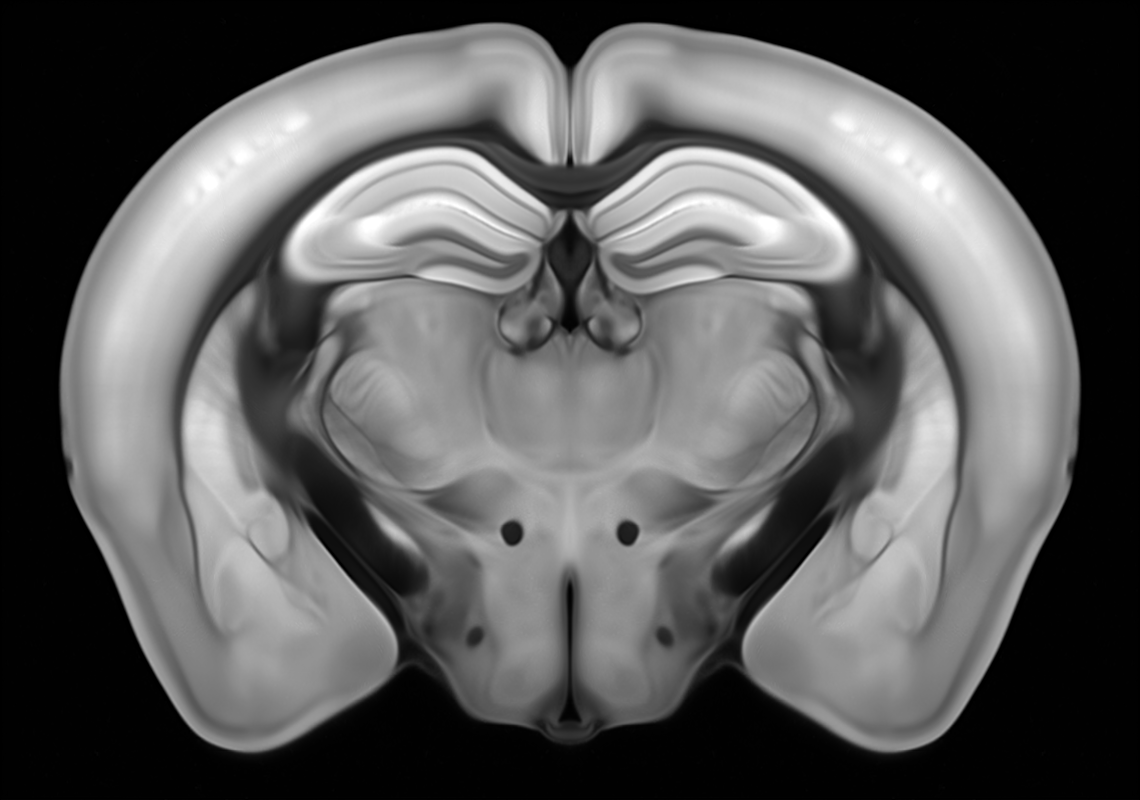

Supplement: Supplementary file 7 — Supplementary Data 5 [file 41467_2019_13057_MOESM7_ESM.zip › Suppl_File2_CCFbackground/AllenCCF_Z062.tif]

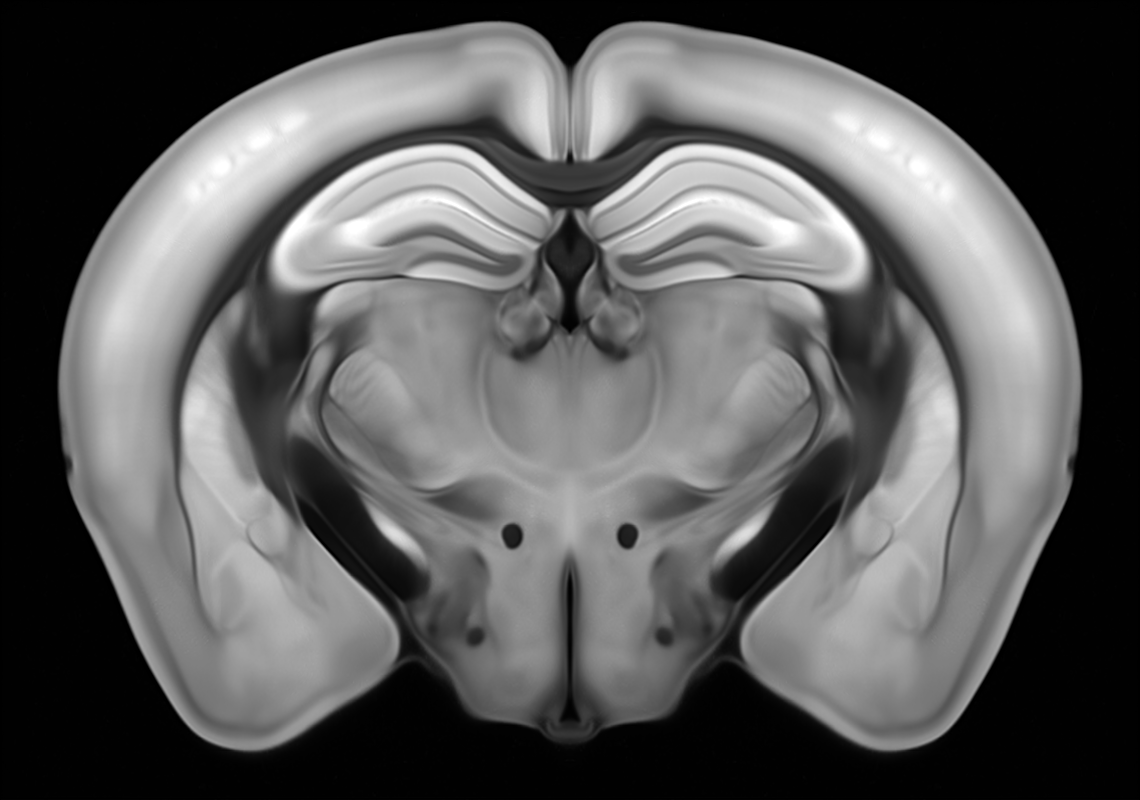

Supplement: Supplementary file 7 — Supplementary Data 5 [file 41467_2019_13057_MOESM7_ESM.zip › Suppl_File2_CCFbackground/AllenCCF_Z063.tif]

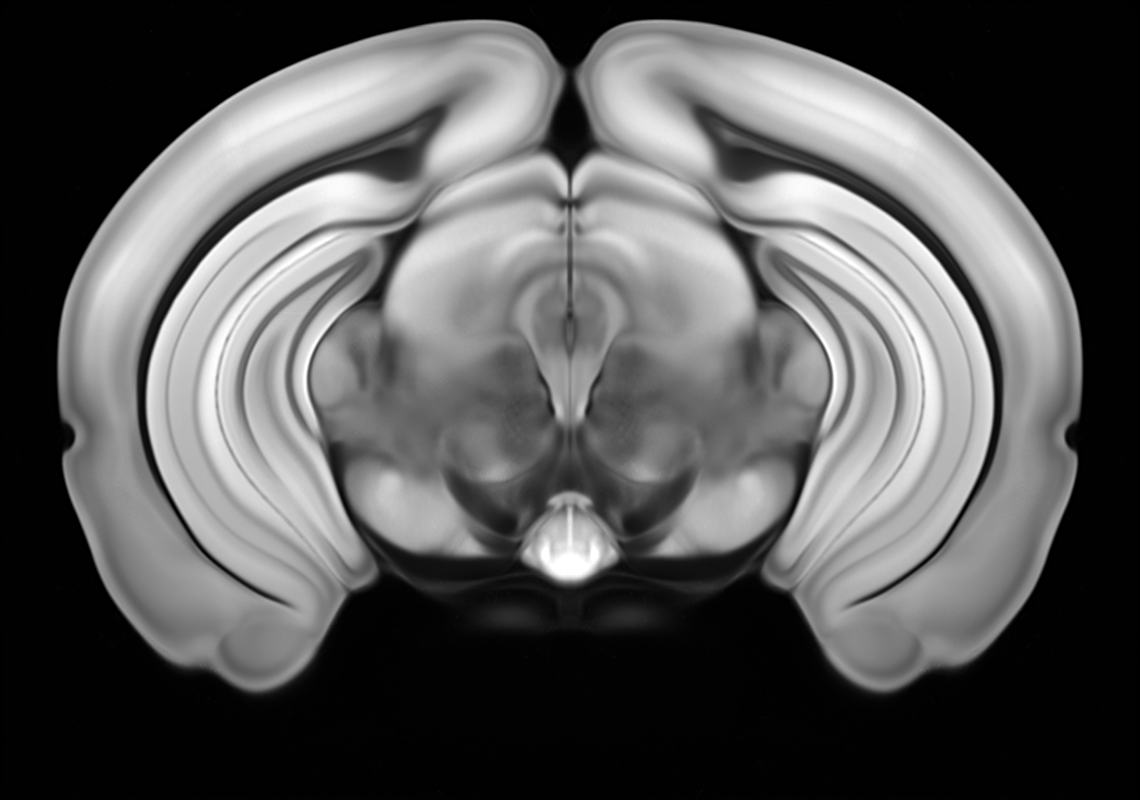

Supplement: Supplementary file 7 — Supplementary Data 5 [file 41467_2019_13057_MOESM7_ESM.zip › Suppl_File2_CCFbackground/AllenCCF_Z077.tif]

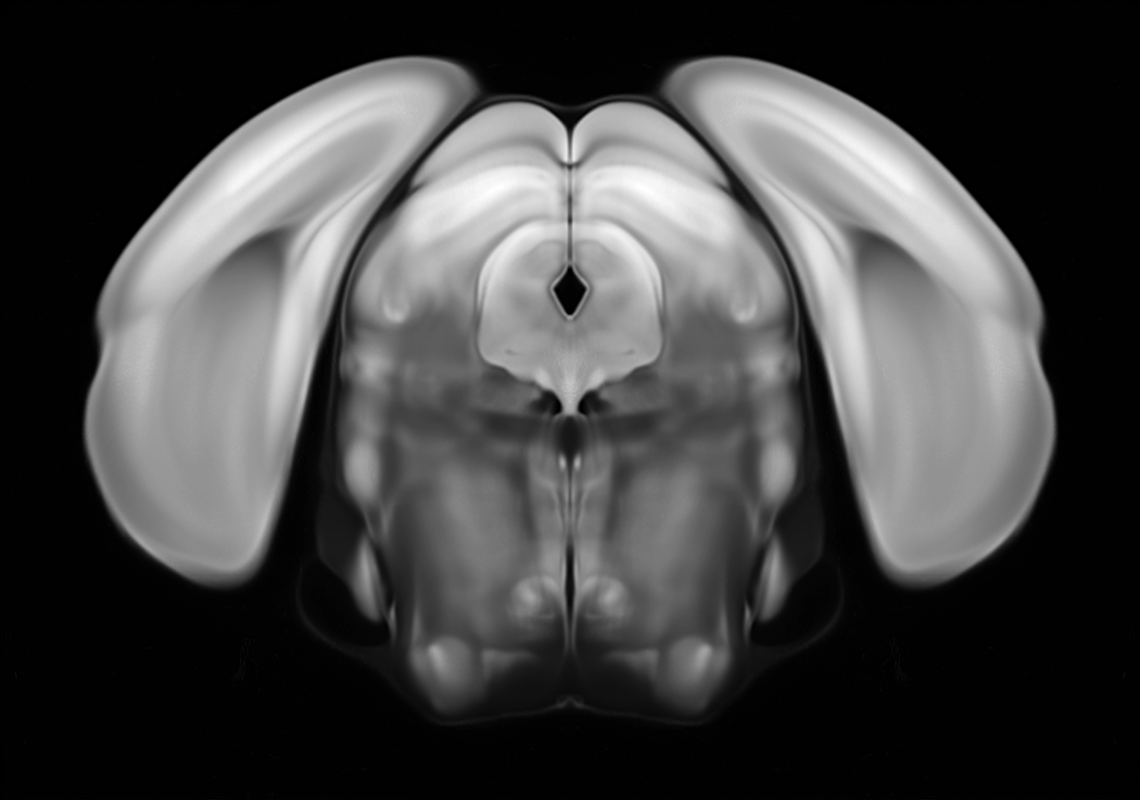

Supplement: Supplementary file 7 — Supplementary Data 5 [file 41467_2019_13057_MOESM7_ESM.zip › Suppl_File2_CCFbackground/AllenCCF_Z088.tif]

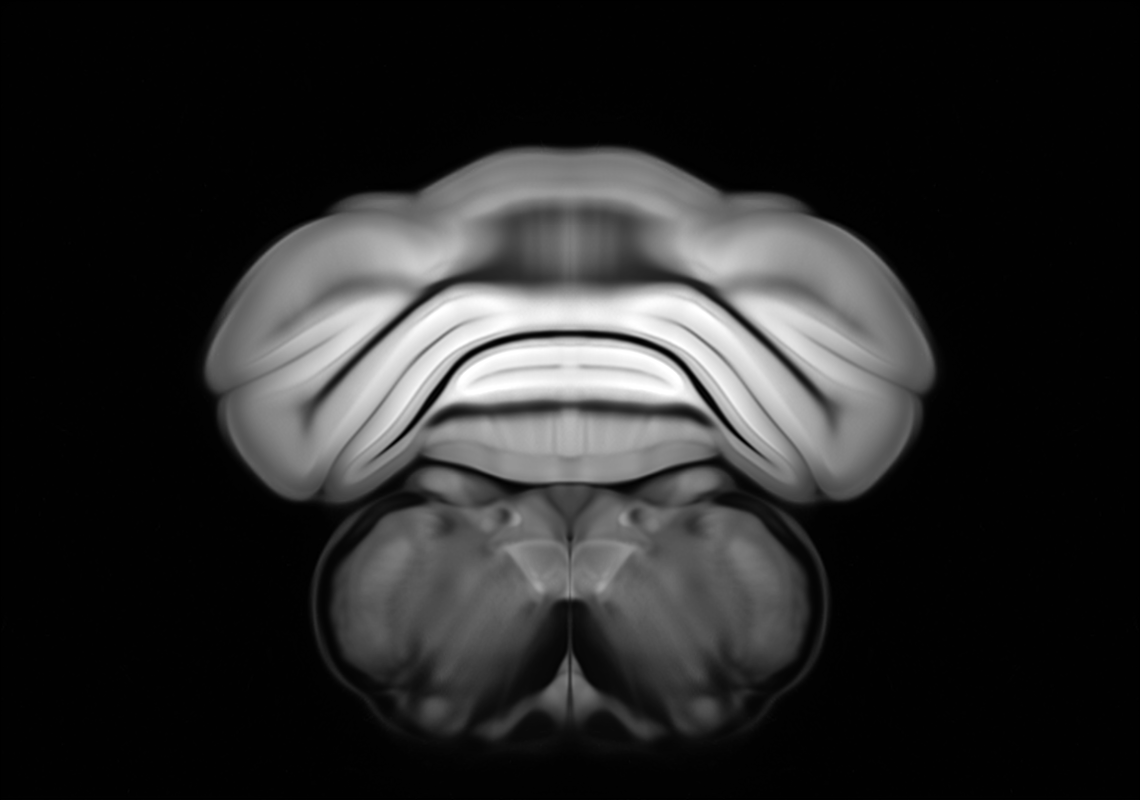

Supplement: Supplementary file 7 — Supplementary Data 5 [file 41467_2019_13057_MOESM7_ESM.zip › Suppl_File2_CCFbackground/AllenCCF_Z117.tif]

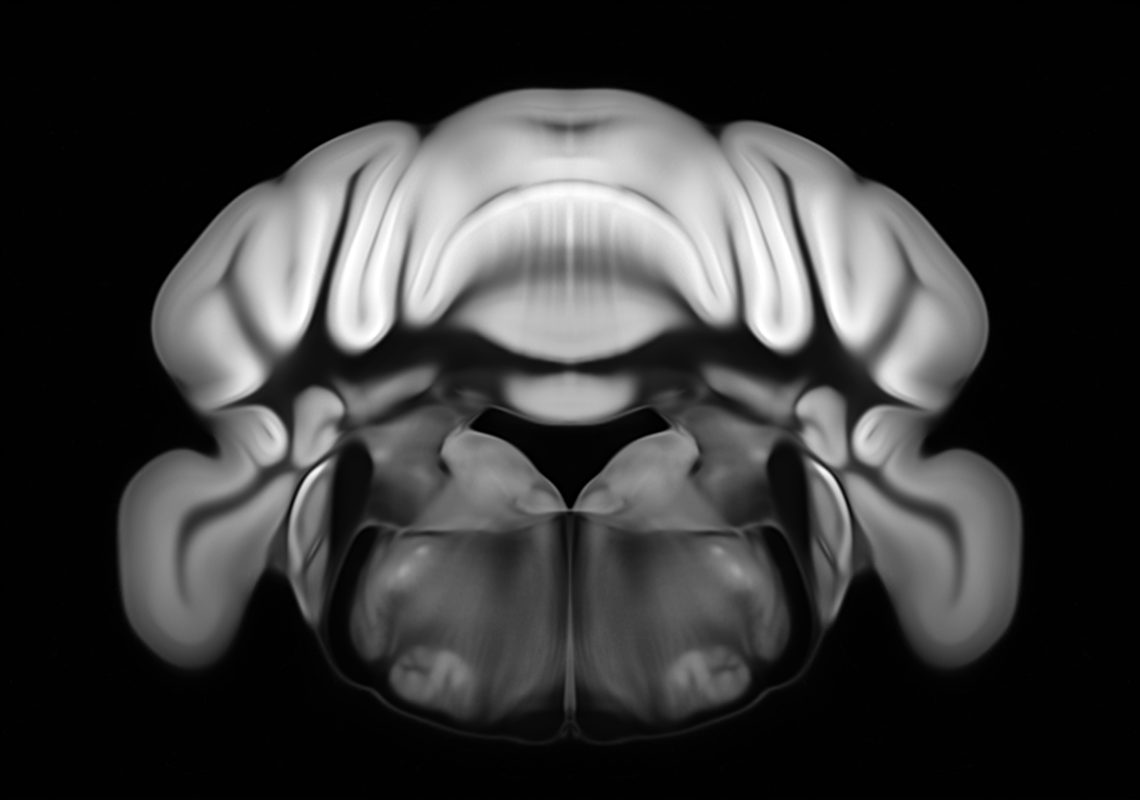

Supplement: Supplementary file 7 — Supplementary Data 5 [file 41467_2019_13057_MOESM7_ESM.zip › Suppl_File2_CCFbackground/AllenCCF_Z103.tif]

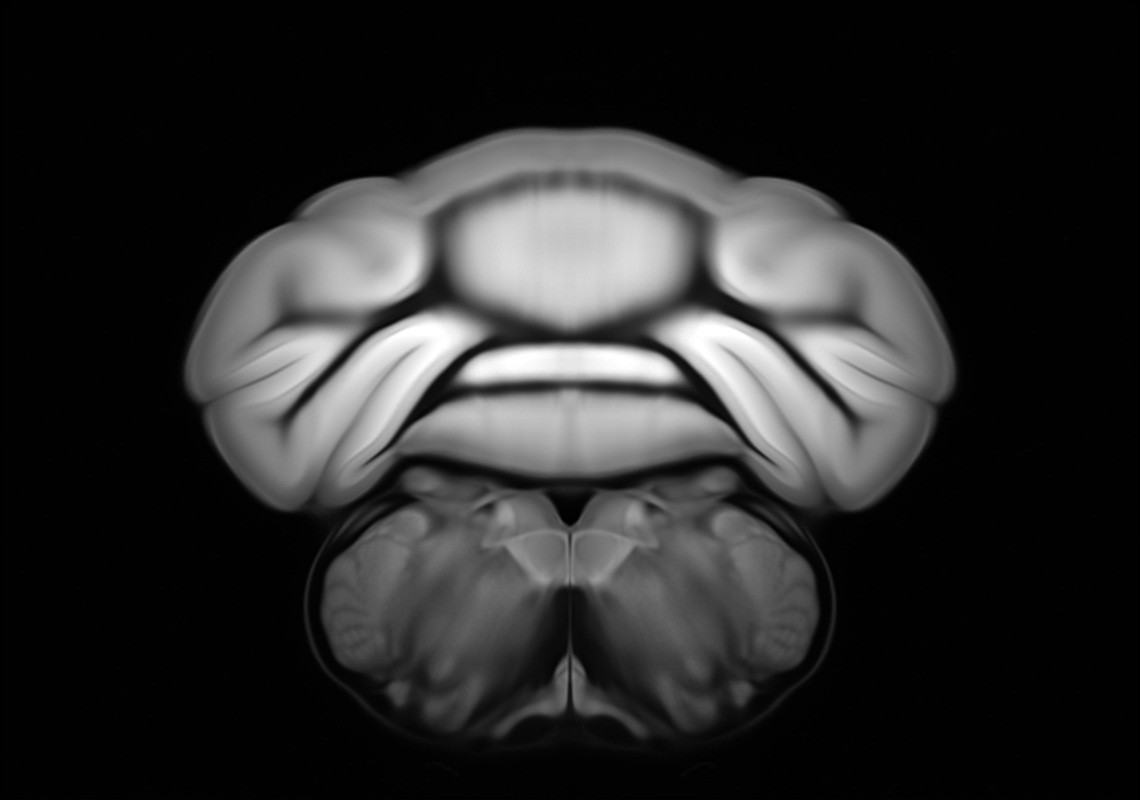

Supplement: Supplementary file 7 — Supplementary Data 5 [file 41467_2019_13057_MOESM7_ESM.zip › Suppl_File2_CCFbackground/AllenCCF_Z115.tif]

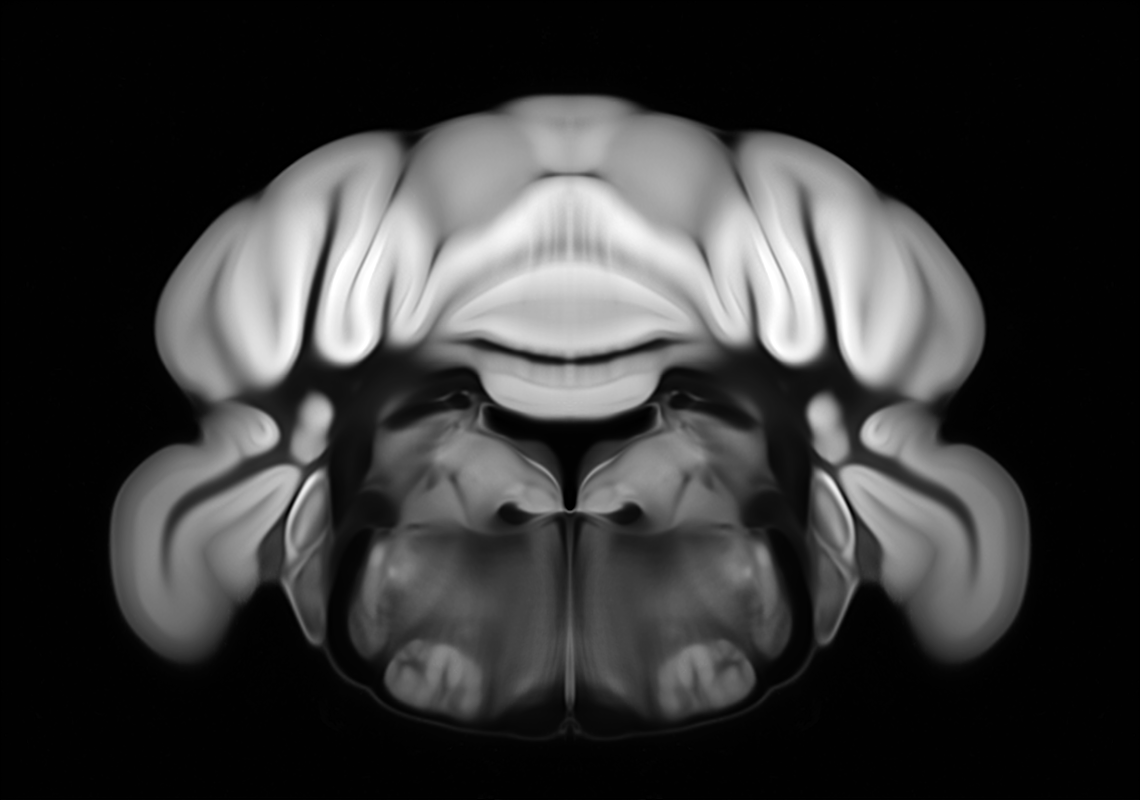

Supplement: Supplementary file 7 — Supplementary Data 5 [file 41467_2019_13057_MOESM7_ESM.zip › Suppl_File2_CCFbackground/AllenCCF_Z101.tif]

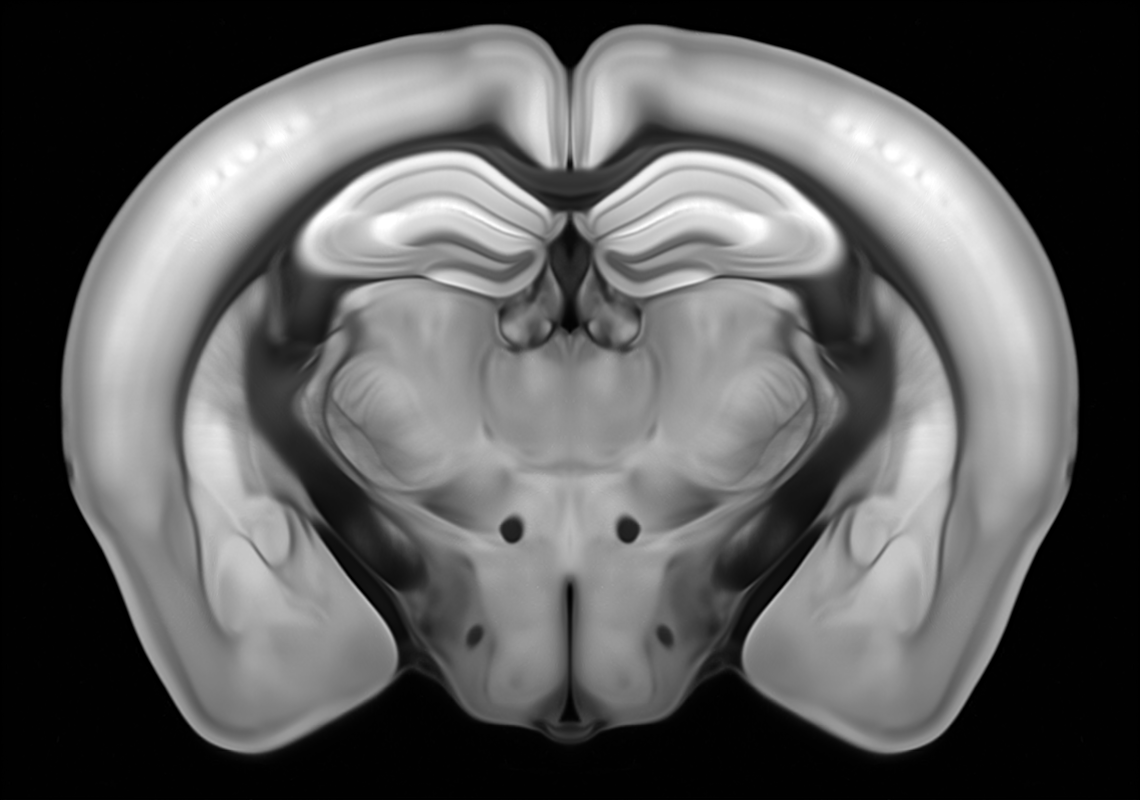

Supplement: Supplementary file 7 — Supplementary Data 5 [file 41467_2019_13057_MOESM7_ESM.zip › Suppl_File2_CCFbackground/AllenCCF_Z061.tif]
