# Supplementary figures and images for: Enhanced and unified anatomical labeling for a common mouse brain atlas (part 1 of 3)
Source: Nat Commun. 2019 Nov 7;10:5067. doi: 10.1038/s41467-019-13057-w (PMC6838086; doi:10.1038/s41467-019-13057-w)

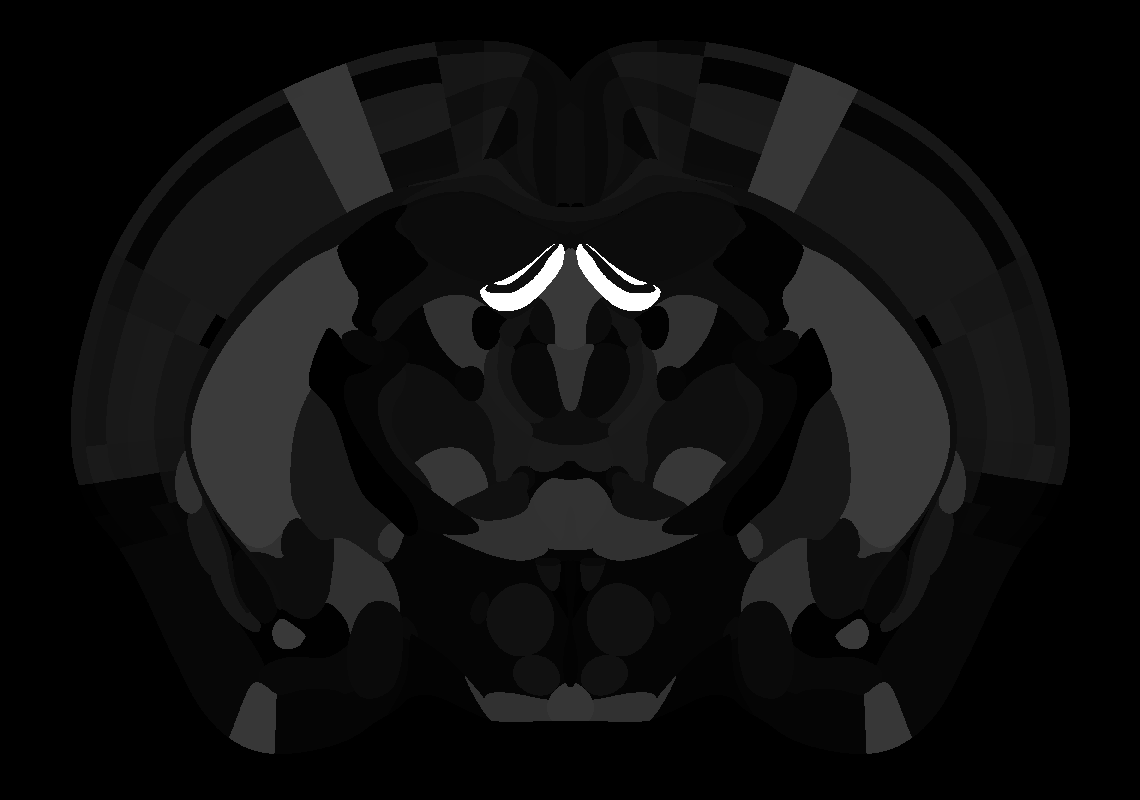

Supplement: Supplementary file 6 — Supplementary Data 4 [file 41467_2019_13057_MOESM6_ESM.zip › Suppl_File1_Labels/55_AP-1.1.tif]

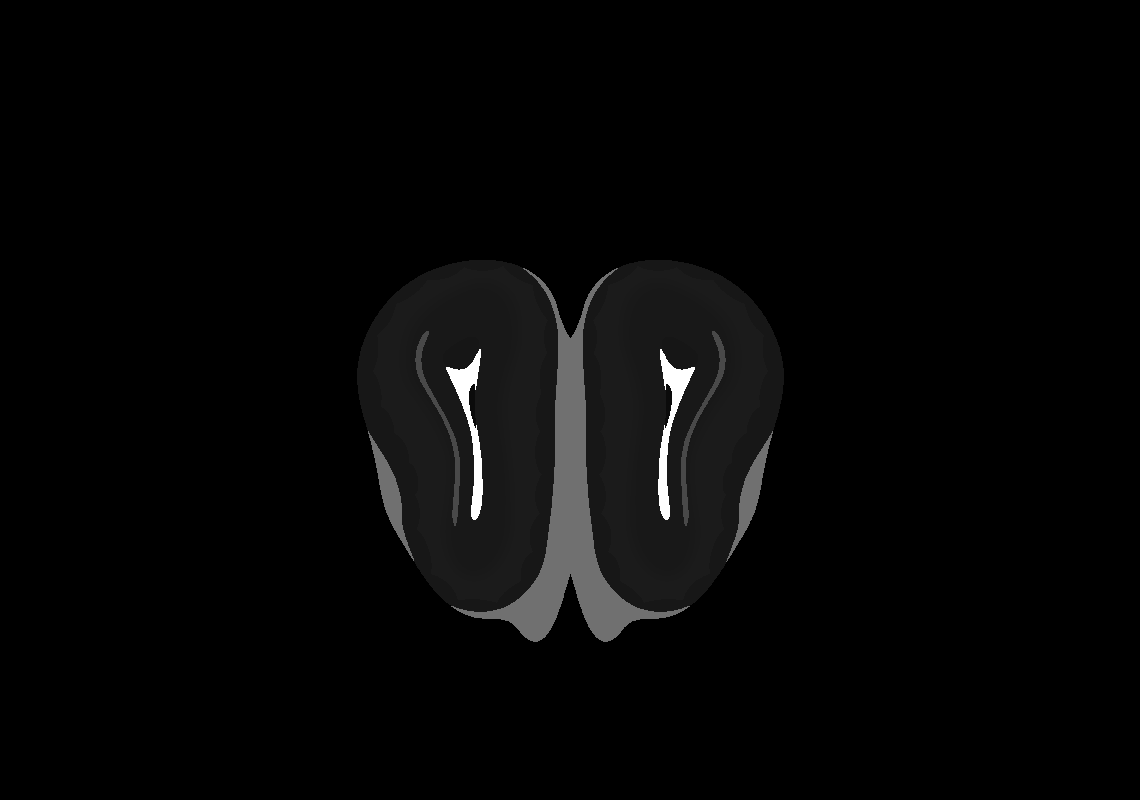

Supplement: Supplementary file 6 — Supplementary Data 4 [file 41467_2019_13057_MOESM6_ESM.zip › Suppl_File1_Labels/6_AP+3.8.tif]

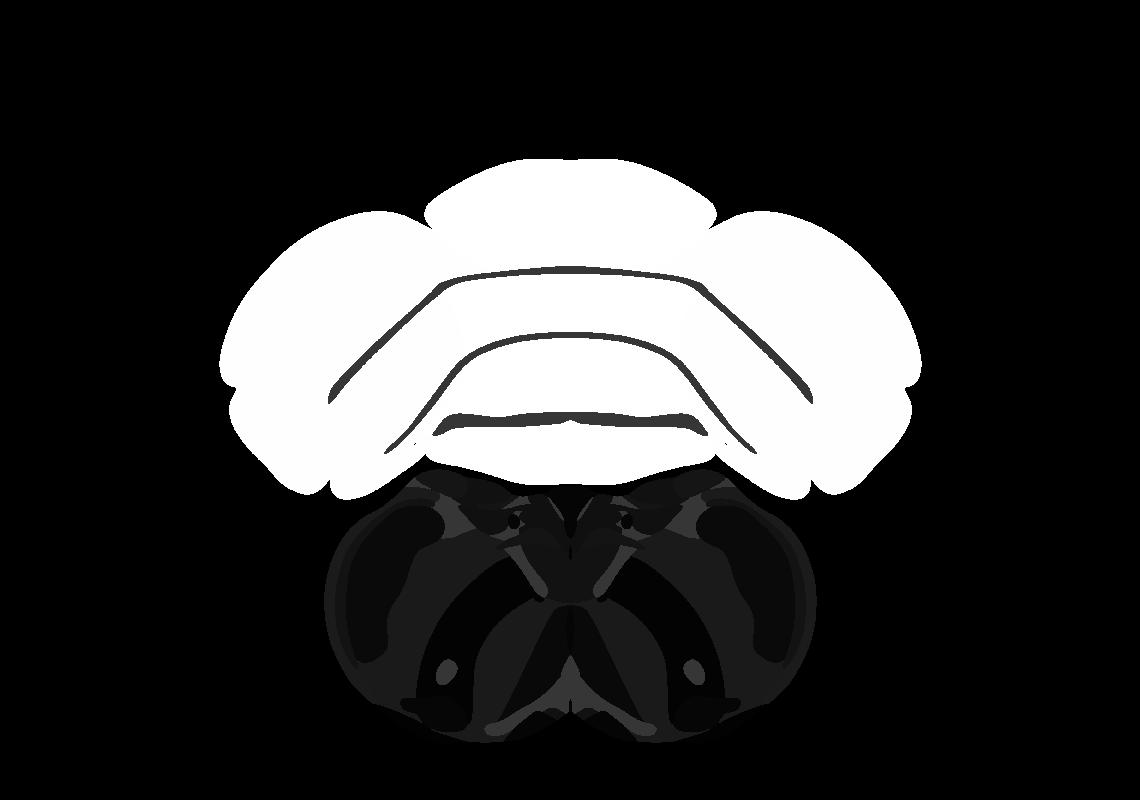

Supplement: Supplementary file 6 — Supplementary Data 4 [file 41467_2019_13057_MOESM6_ESM.zip › Suppl_File1_Labels/118_AP-7.4.tif]

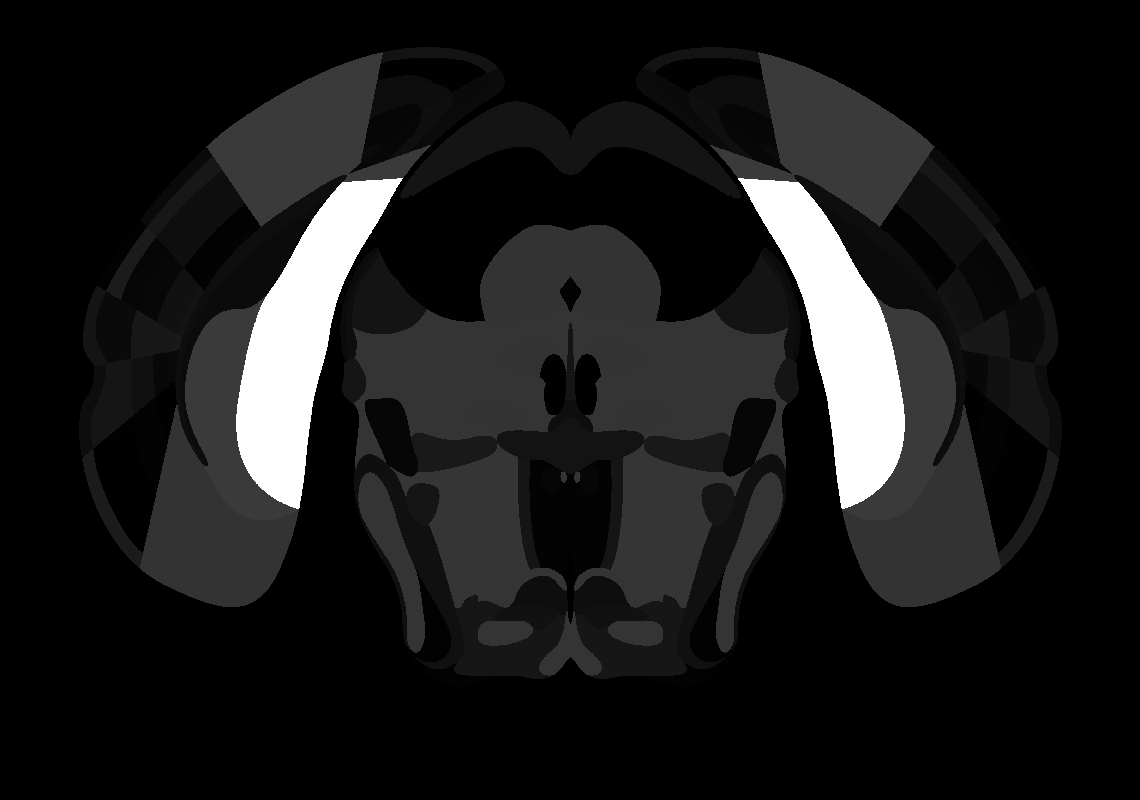

Supplement: Supplementary file 6 — Supplementary Data 4 [file 41467_2019_13057_MOESM6_ESM.zip › Suppl_File1_Labels/86_AP-4.2.tif]

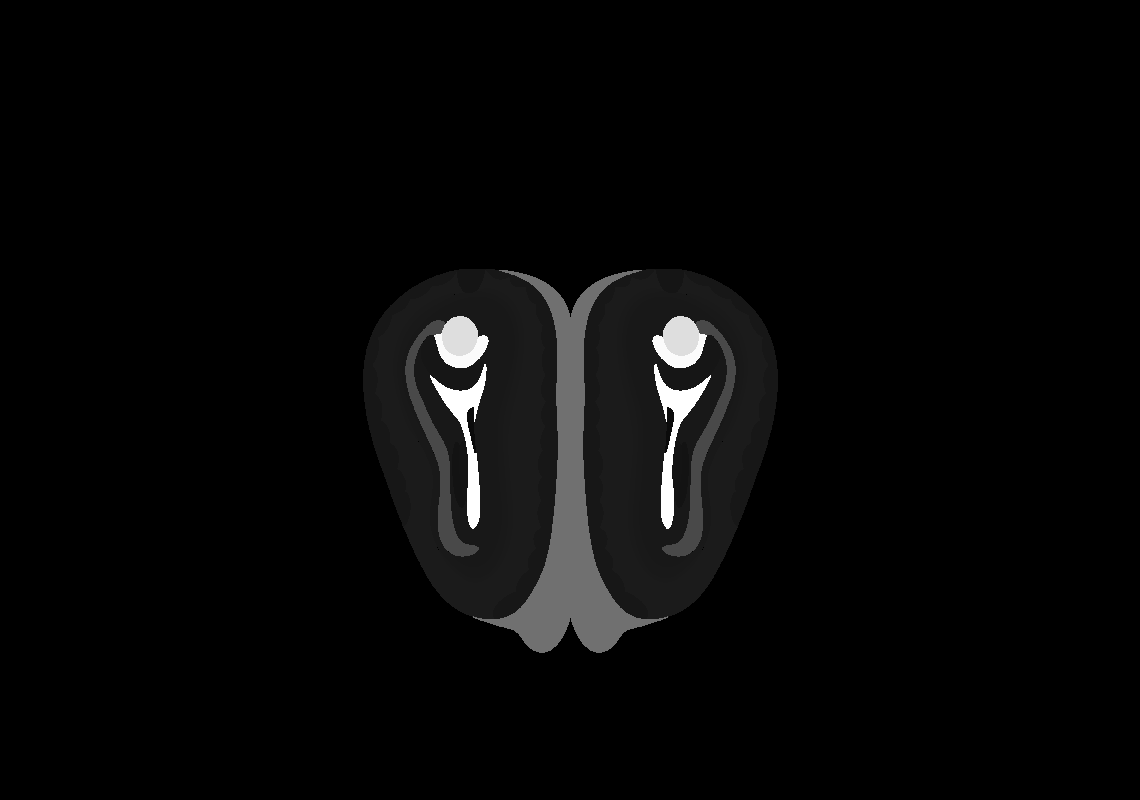

Supplement: Supplementary file 6 — Supplementary Data 4 [file 41467_2019_13057_MOESM6_ESM.zip › Suppl_File1_Labels/8_AP+3.6.tif]

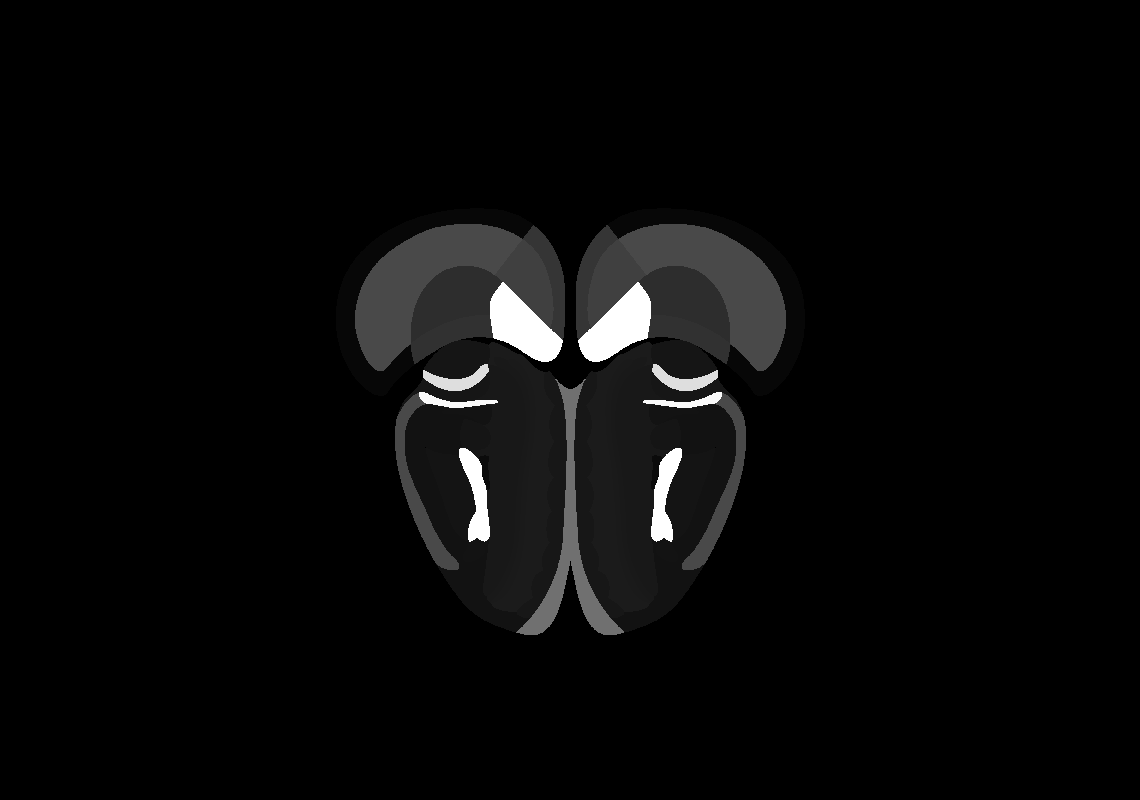

Supplement: Supplementary file 6 — Supplementary Data 4 [file 41467_2019_13057_MOESM6_ESM.zip › Suppl_File1_Labels/13_AP+3.1.tif]

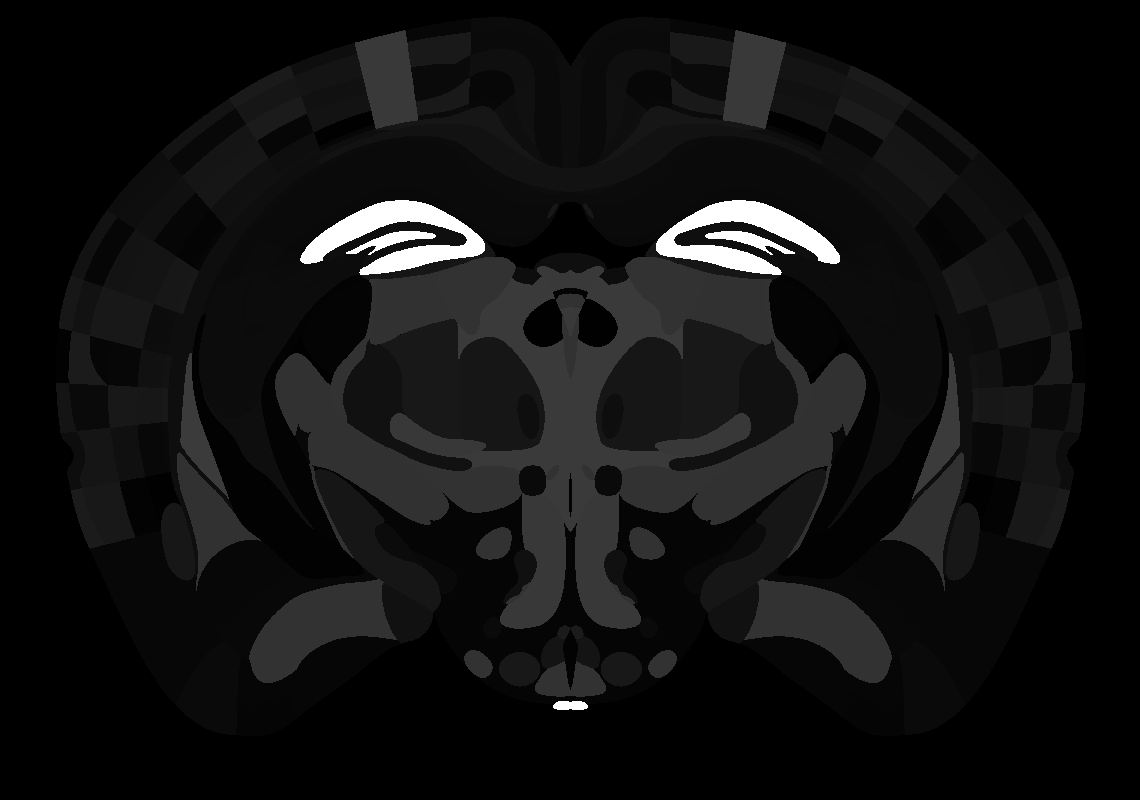

Supplement: Supplementary file 6 — Supplementary Data 4 [file 41467_2019_13057_MOESM6_ESM.zip › Suppl_File1_Labels/68_AP-2.4.tif]

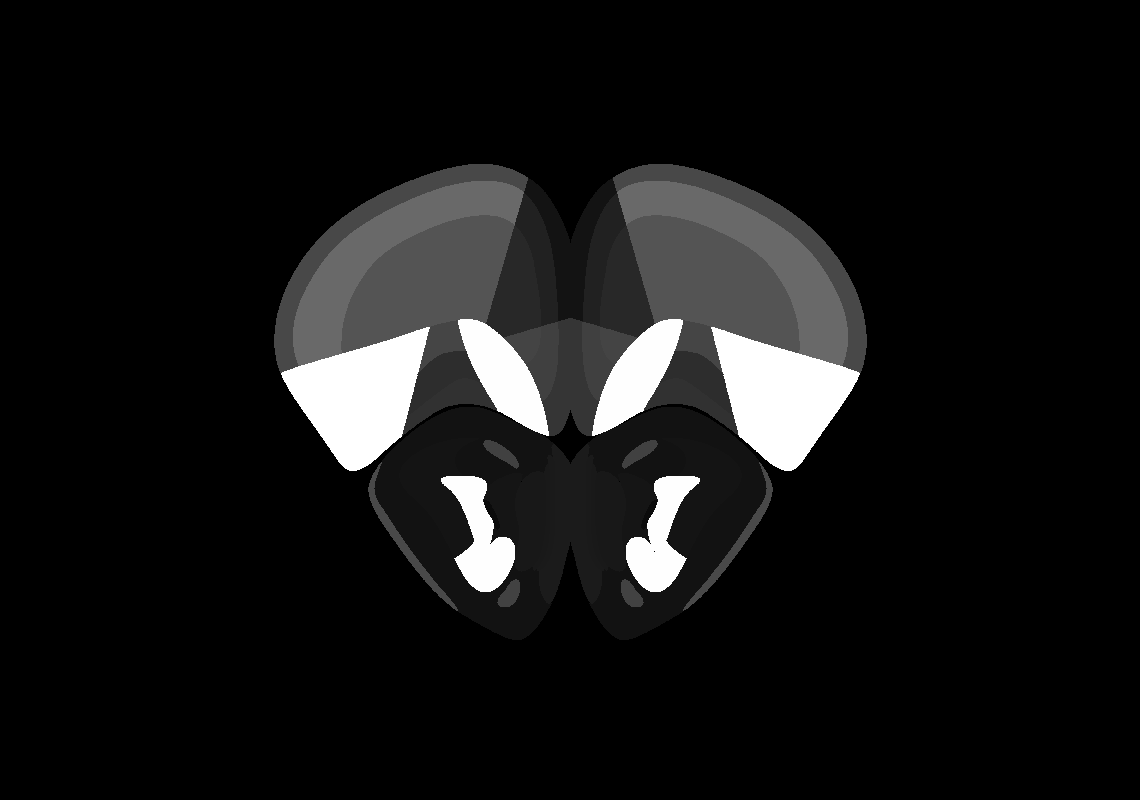

Supplement: Supplementary file 6 — Supplementary Data 4 [file 41467_2019_13057_MOESM6_ESM.zip › Suppl_File1_Labels/19_AP+2.5.tif]

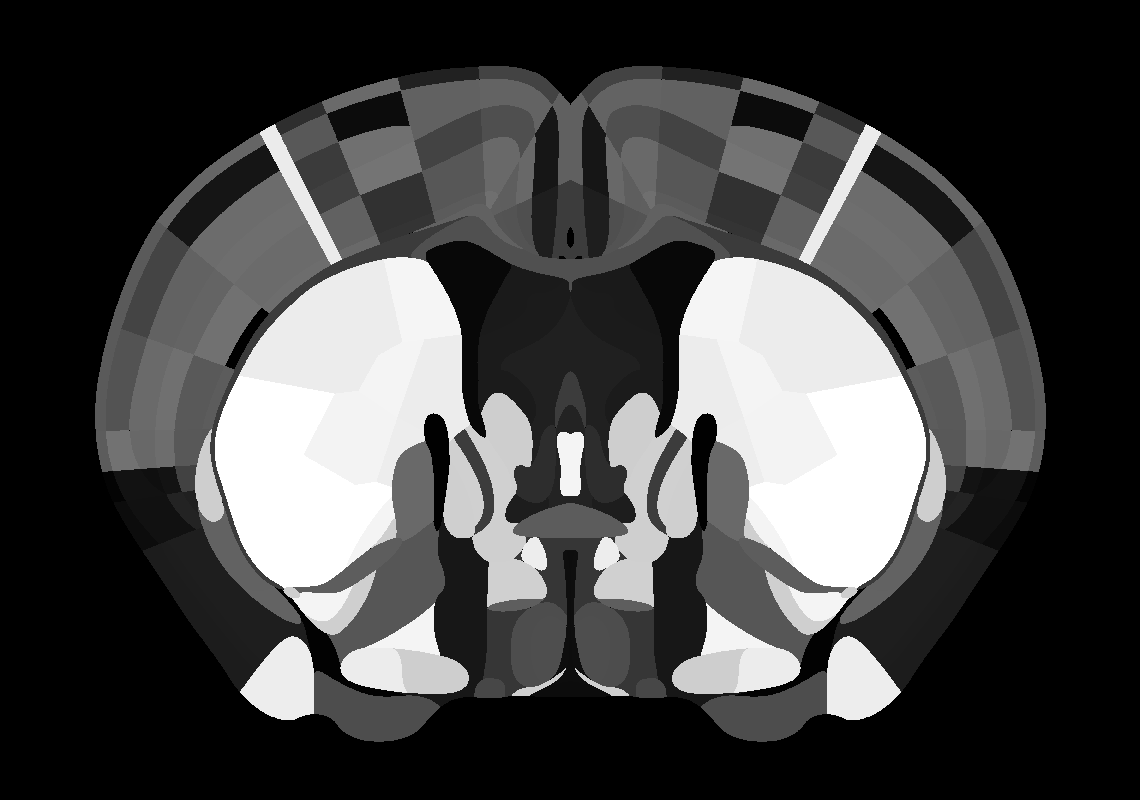

Supplement: Supplementary file 6 — Supplementary Data 4 [file 41467_2019_13057_MOESM6_ESM.zip › Suppl_File1_Labels/46_AP-0.2.tif]

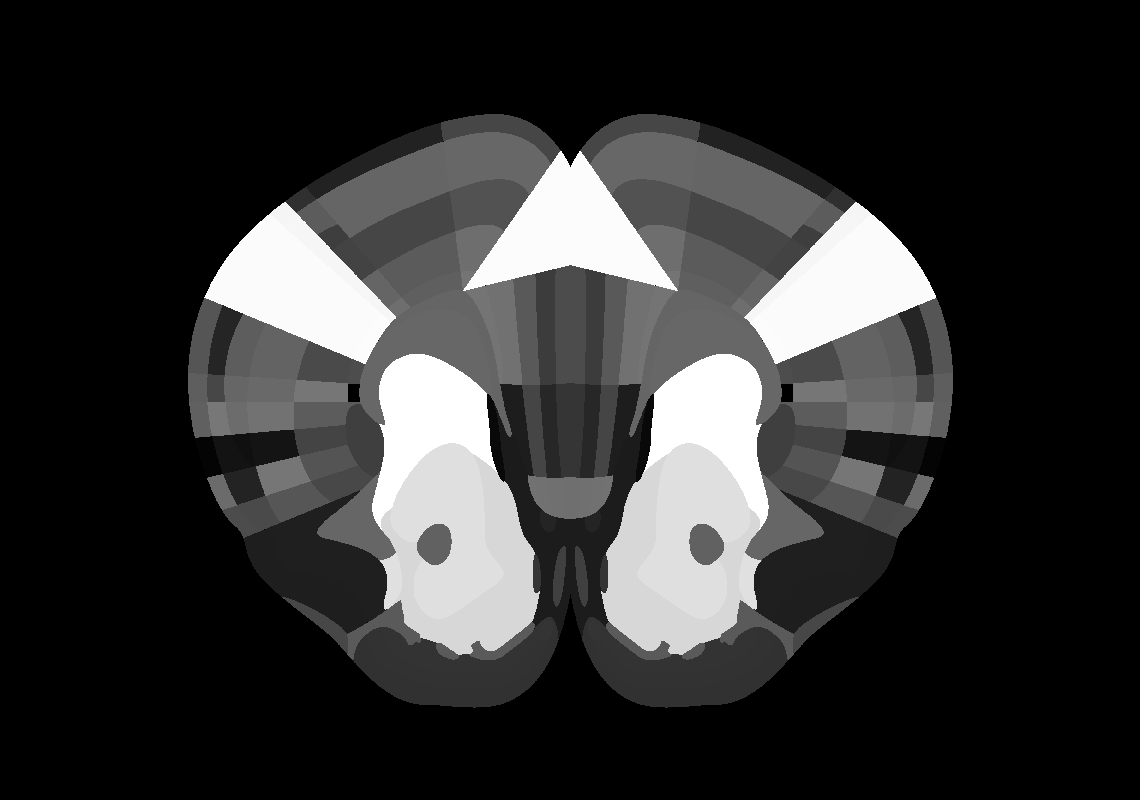

Supplement: Supplementary file 6 — Supplementary Data 4 [file 41467_2019_13057_MOESM6_ESM.zip › Suppl_File1_Labels/30_AP+1.4.tif]

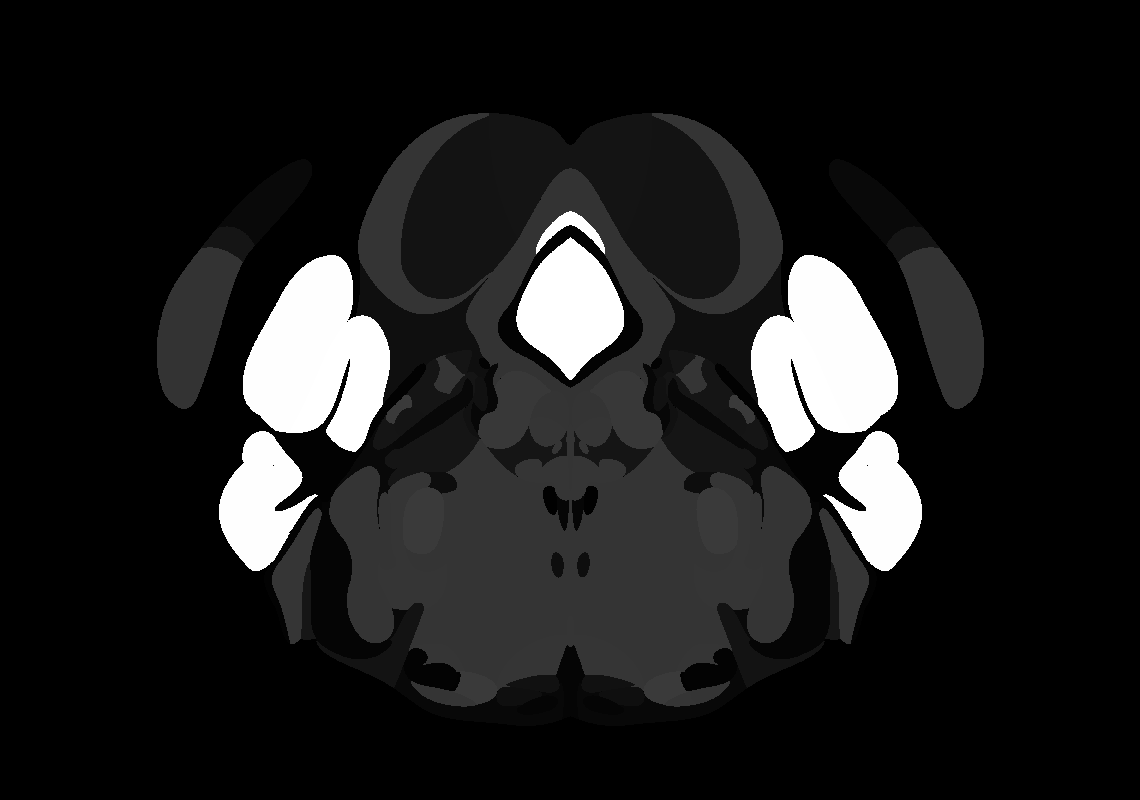

Supplement: Supplementary file 6 — Supplementary Data 4 [file 41467_2019_13057_MOESM6_ESM.zip › Suppl_File1_Labels/95_AP-5.1.tif]

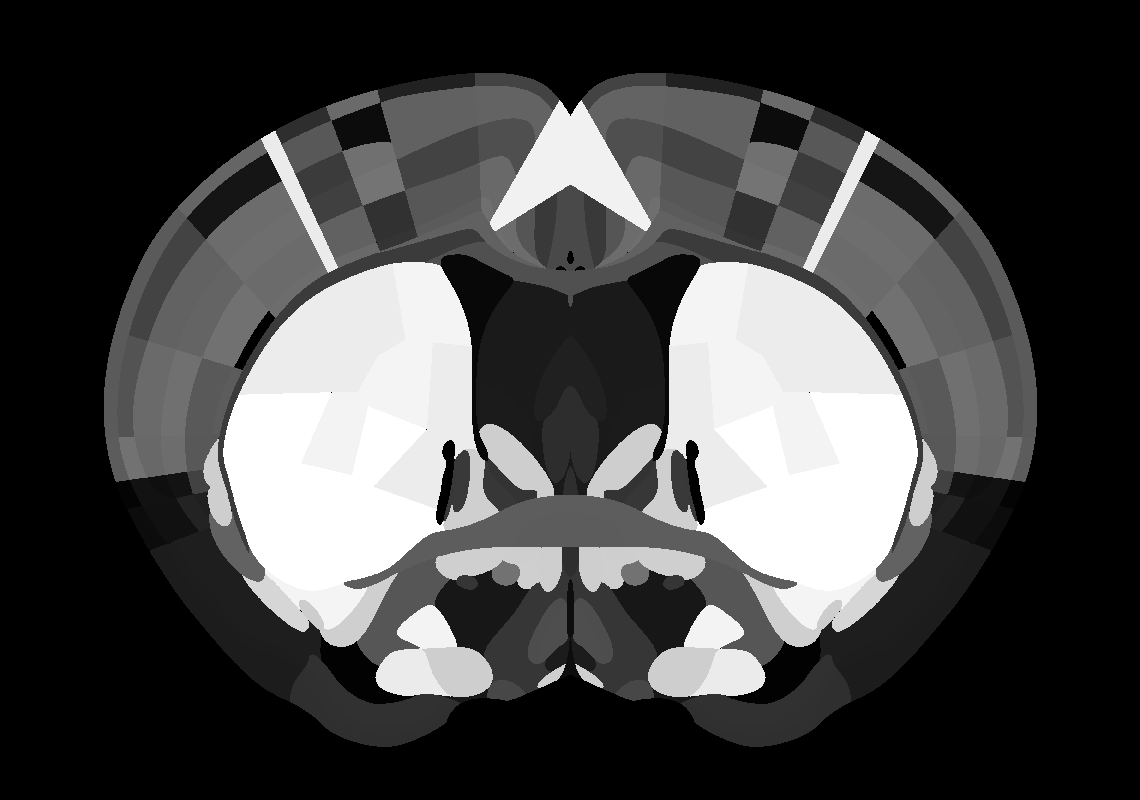

Supplement: Supplementary file 6 — Supplementary Data 4 [file 41467_2019_13057_MOESM6_ESM.zip › Suppl_File1_Labels/44_AP+0.0.tif]

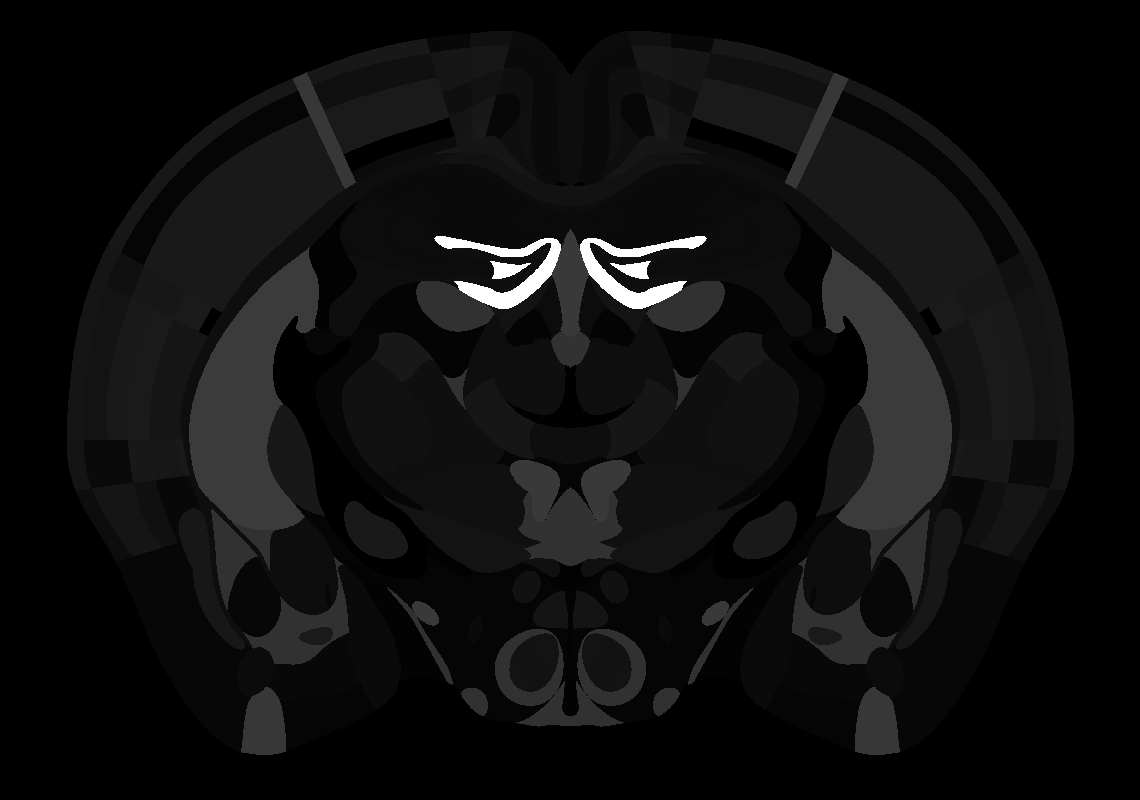

Supplement: Supplementary file 6 — Supplementary Data 4 [file 41467_2019_13057_MOESM6_ESM.zip › Suppl_File1_Labels/58_AP-1.4.tif]

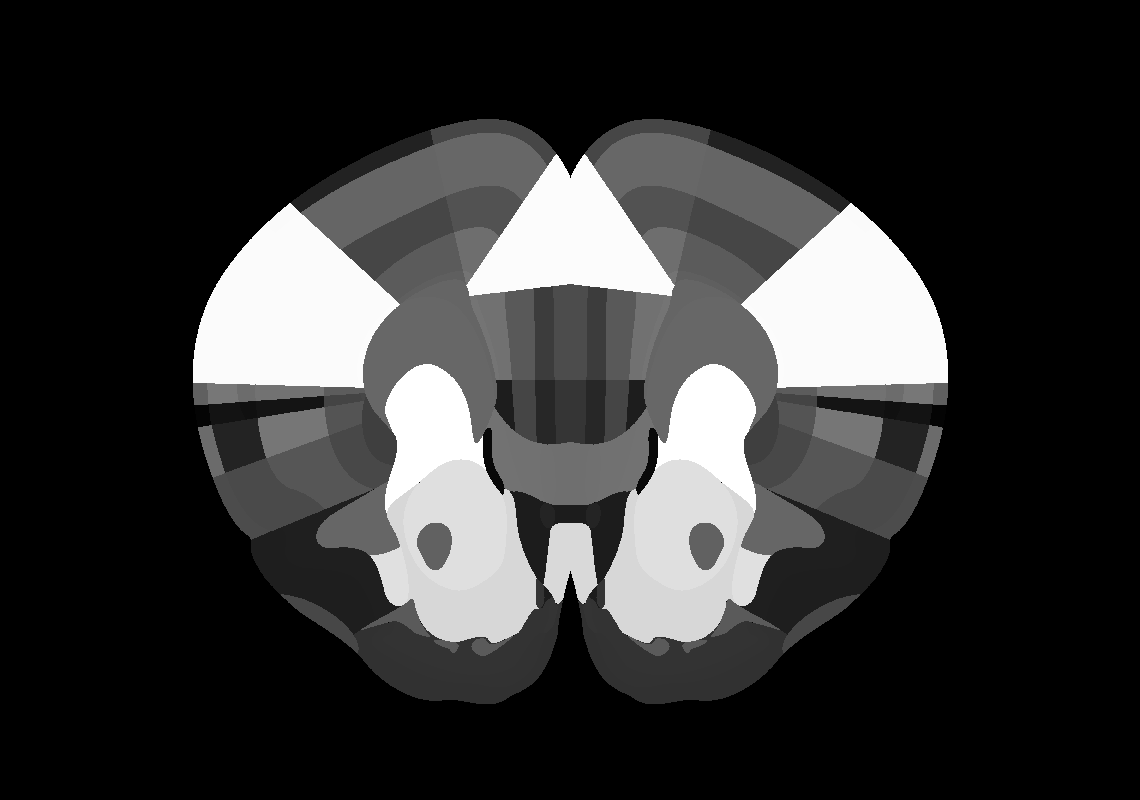

Supplement: Supplementary file 6 — Supplementary Data 4 [file 41467_2019_13057_MOESM6_ESM.zip › Suppl_File1_Labels/29_AP+1.5.tif]

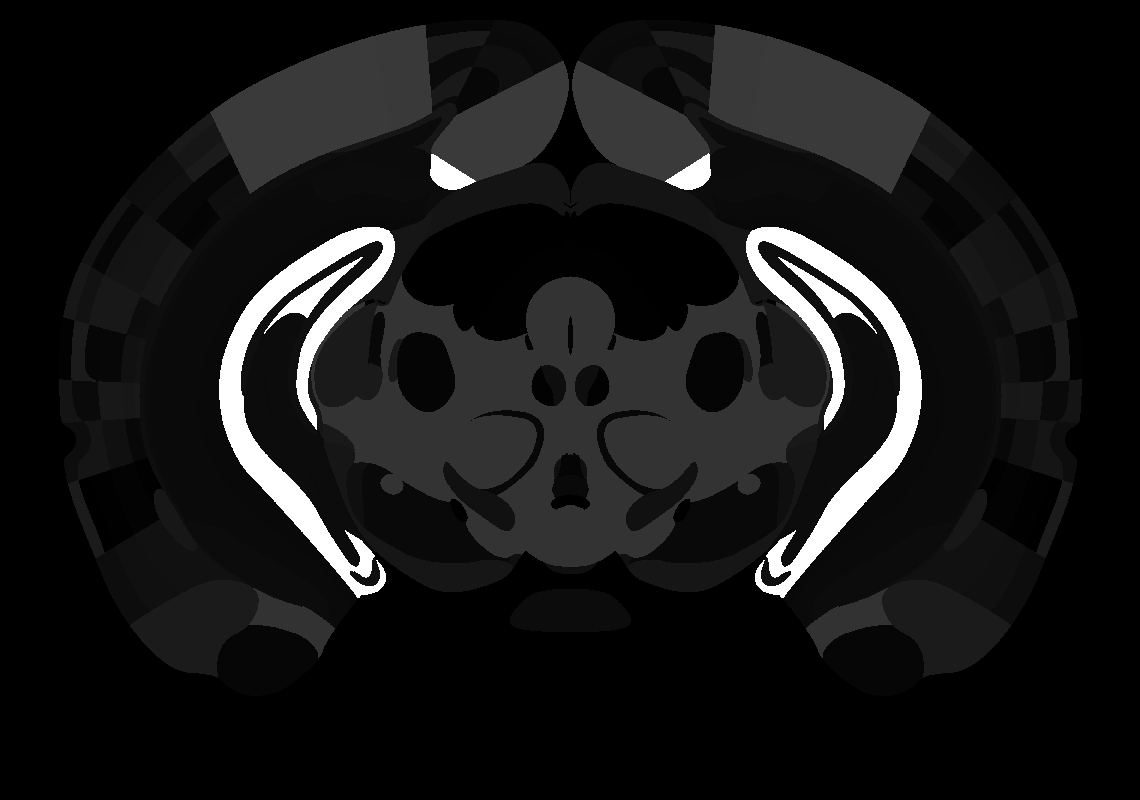

Supplement: Supplementary file 6 — Supplementary Data 4 [file 41467_2019_13057_MOESM6_ESM.zip › Suppl_File1_Labels/76_AP-3.2.tif]

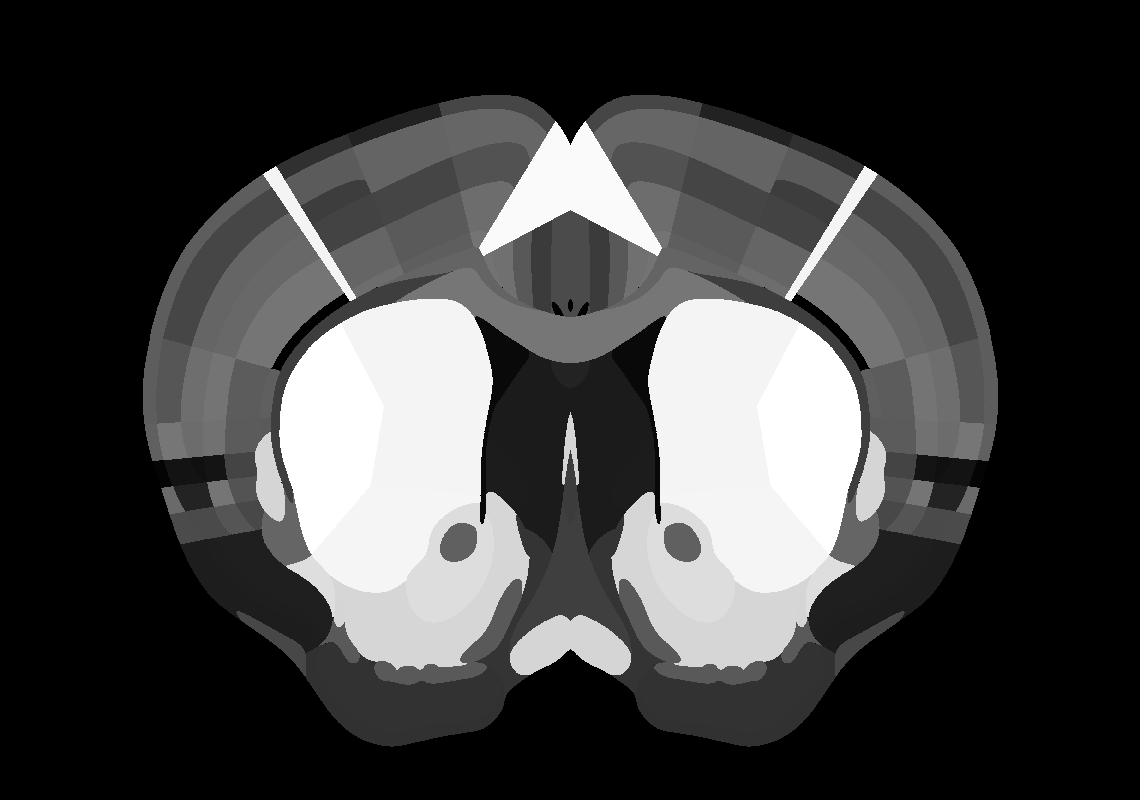

Supplement: Supplementary file 6 — Supplementary Data 4 [file 41467_2019_13057_MOESM6_ESM.zip › Suppl_File1_Labels/37_AP+0.7.tif]

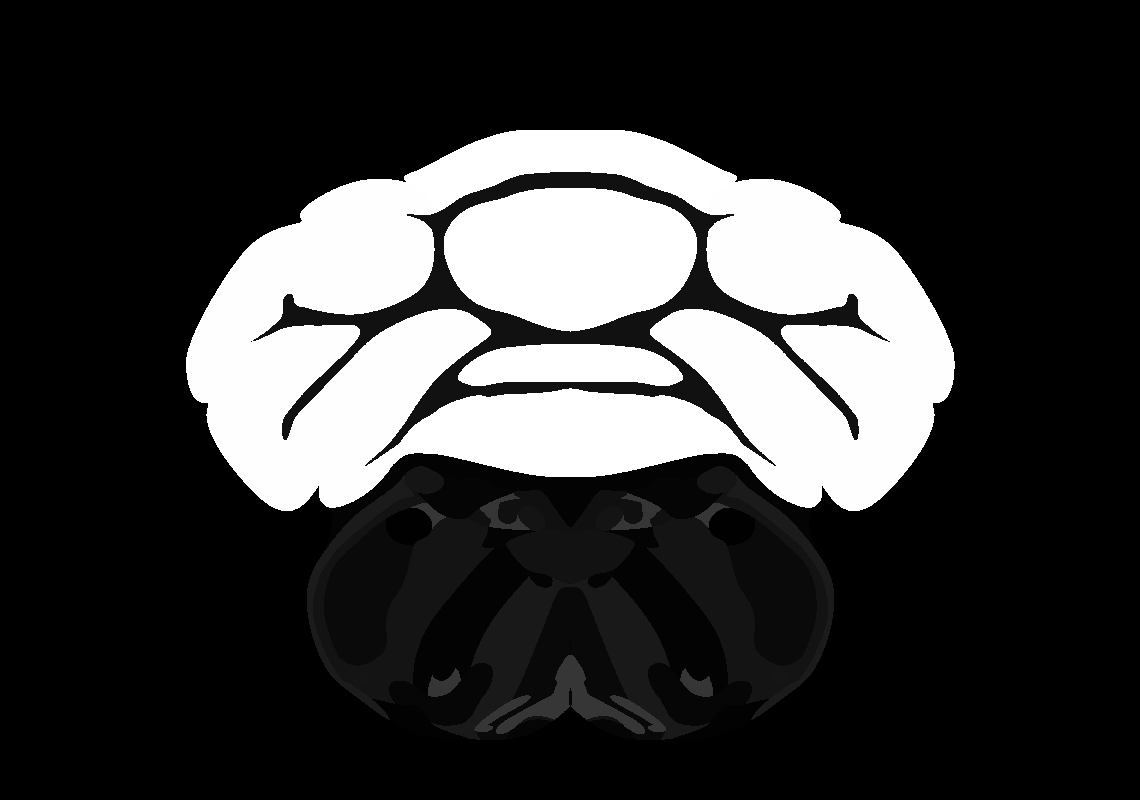

Supplement: Supplementary file 6 — Supplementary Data 4 [file 41467_2019_13057_MOESM6_ESM.zip › Suppl_File1_Labels/115_AP-7.1.tif]

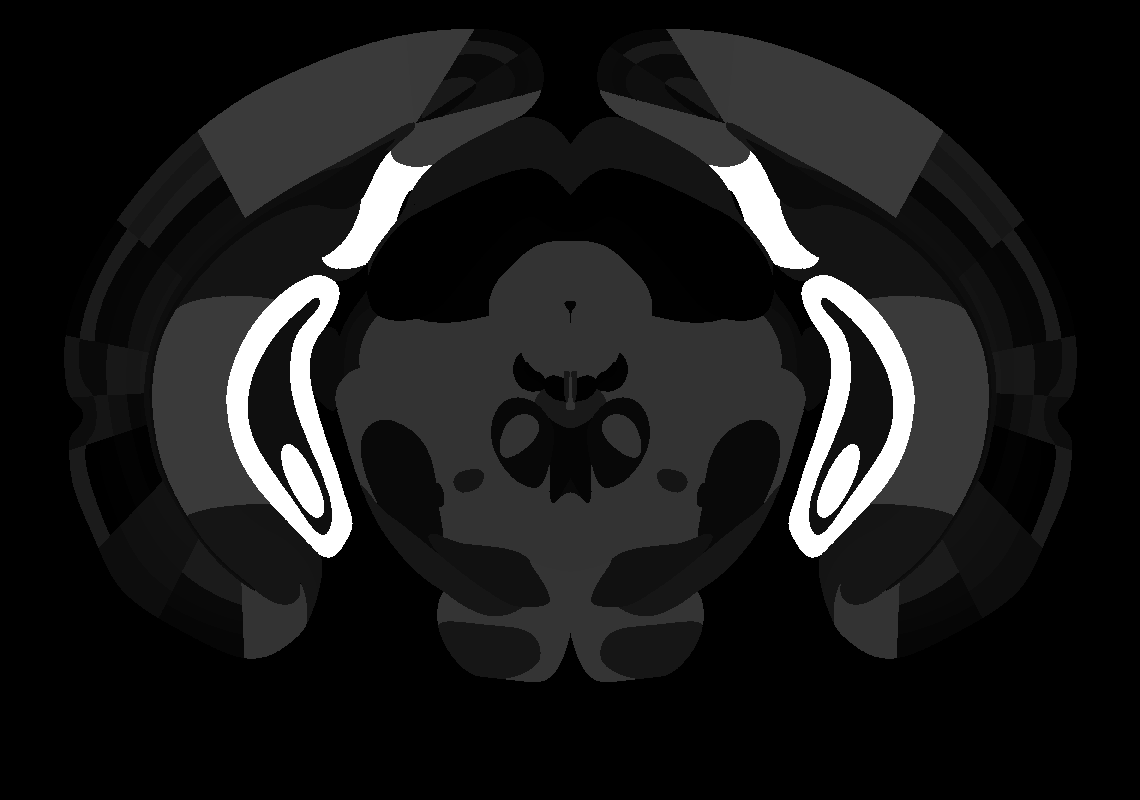

Supplement: Supplementary file 6 — Supplementary Data 4 [file 41467_2019_13057_MOESM6_ESM.zip › Suppl_File1_Labels/81_AP-3.7.tif]

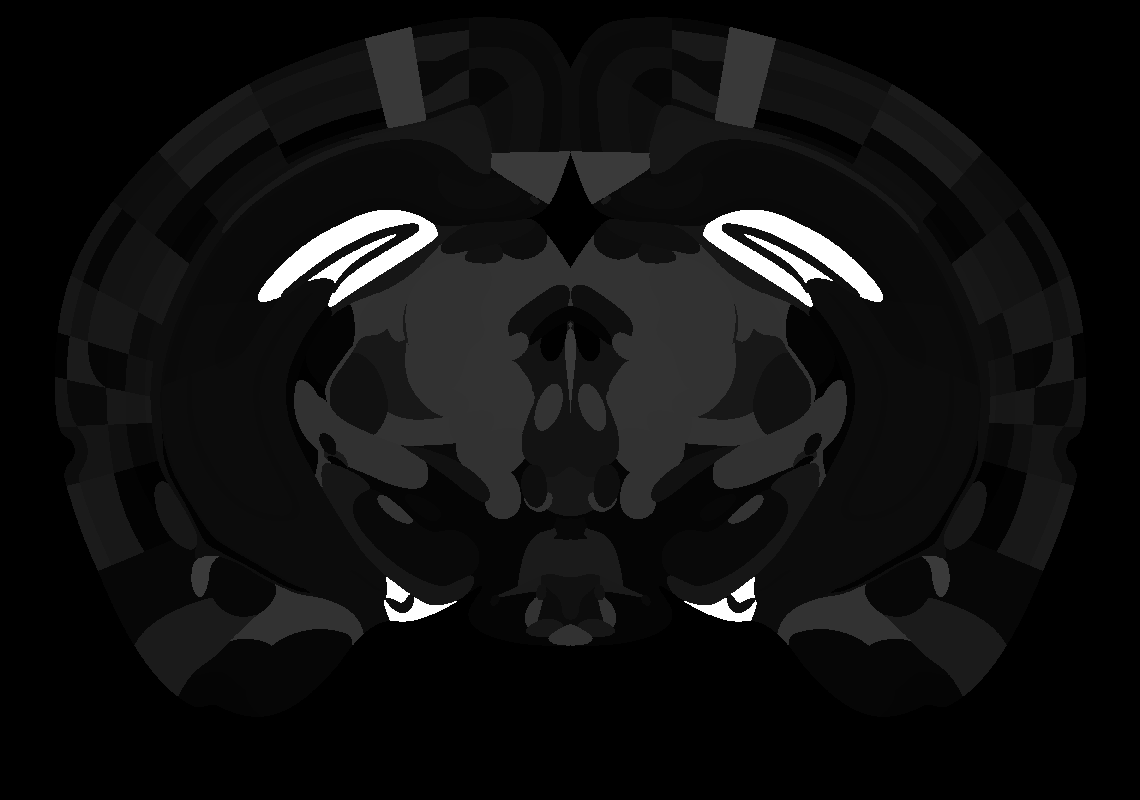

Supplement: Supplementary file 6 — Supplementary Data 4 [file 41467_2019_13057_MOESM6_ESM.zip › Suppl_File1_Labels/72_AP-2.8.tif]

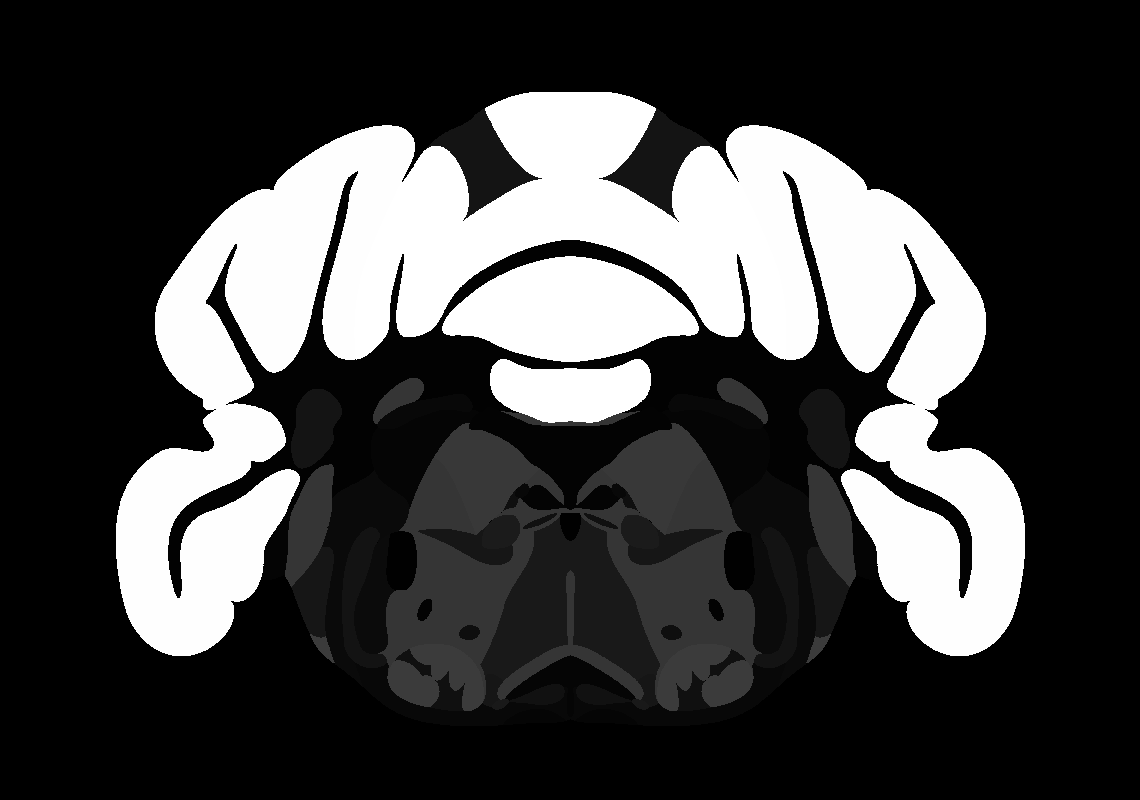

Supplement: Supplementary file 6 — Supplementary Data 4 [file 41467_2019_13057_MOESM6_ESM.zip › Suppl_File1_Labels/102_AP-5.8.tif]

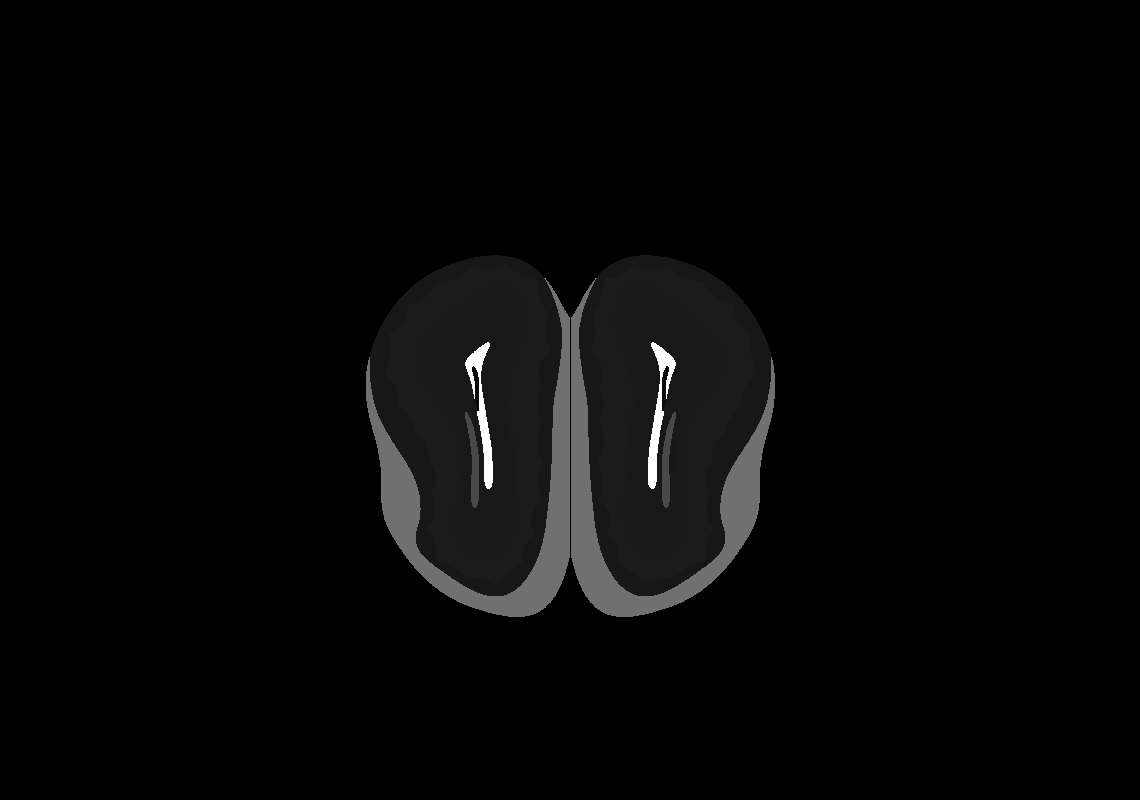

Supplement: Supplementary file 6 — Supplementary Data 4 [file 41467_2019_13057_MOESM6_ESM.zip › Suppl_File1_Labels/2_AP+4.2.tif]

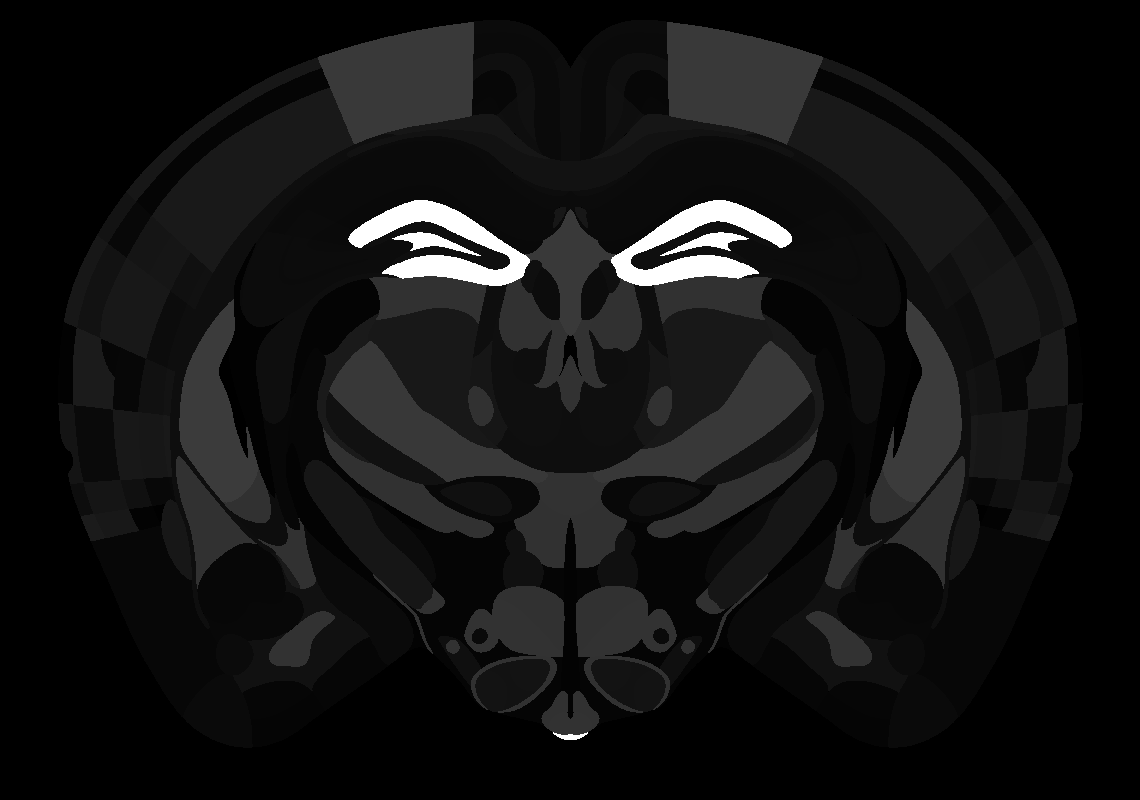

Supplement: Supplementary file 6 — Supplementary Data 4 [file 41467_2019_13057_MOESM6_ESM.zip › Suppl_File1_Labels/65_AP-2.1.tif]

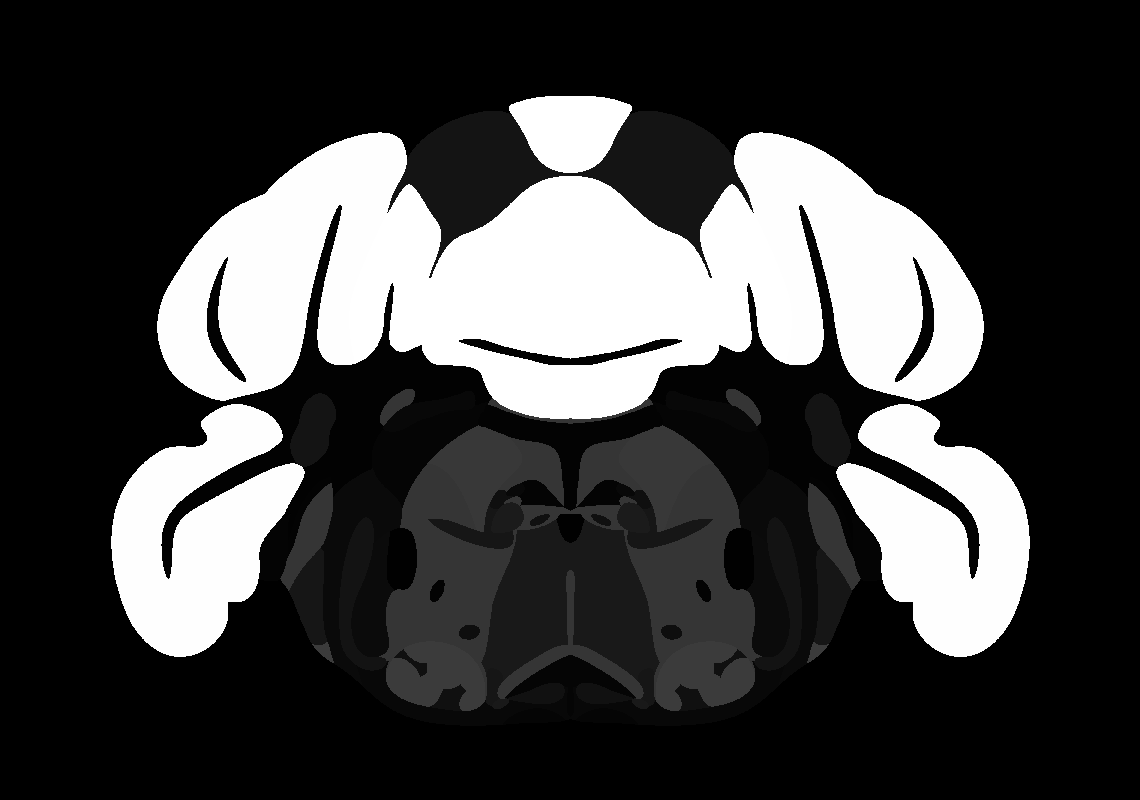

Supplement: Supplementary file 6 — Supplementary Data 4 [file 41467_2019_13057_MOESM6_ESM.zip › Suppl_File1_Labels/101_AP-5.7.tif]

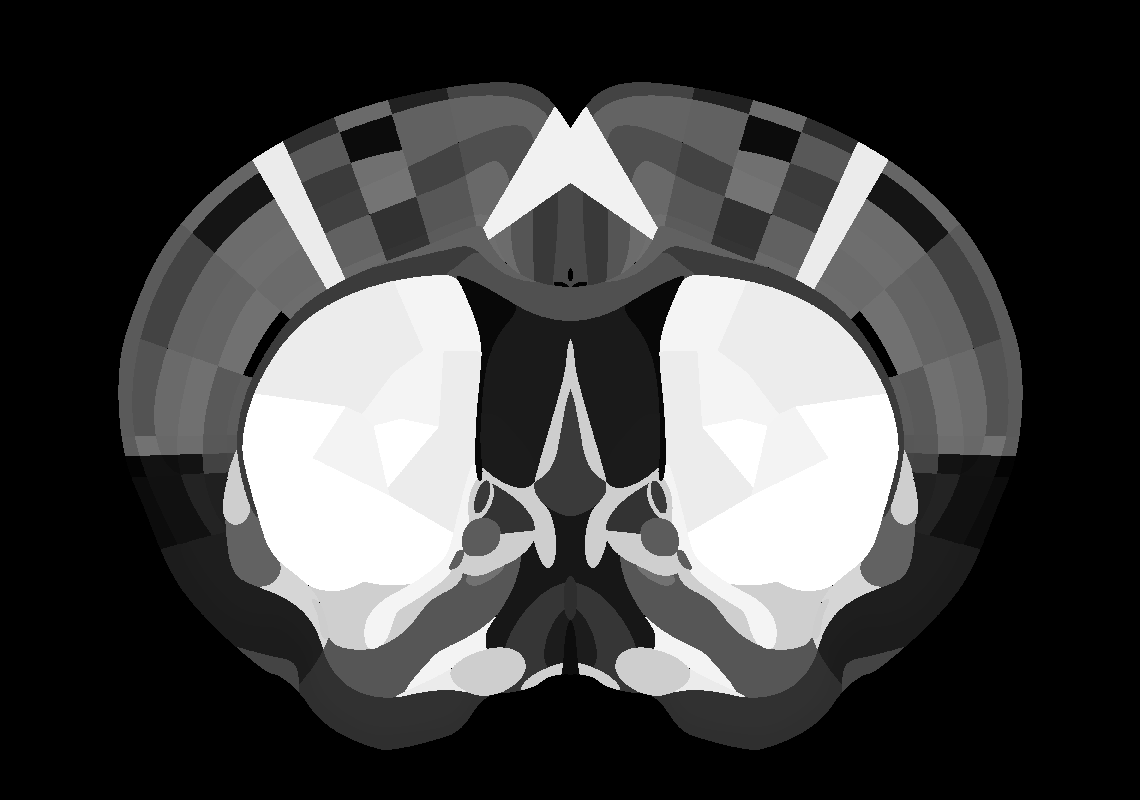

Supplement: Supplementary file 6 — Supplementary Data 4 [file 41467_2019_13057_MOESM6_ESM.zip › Suppl_File1_Labels/41_AP+0.3.tif]

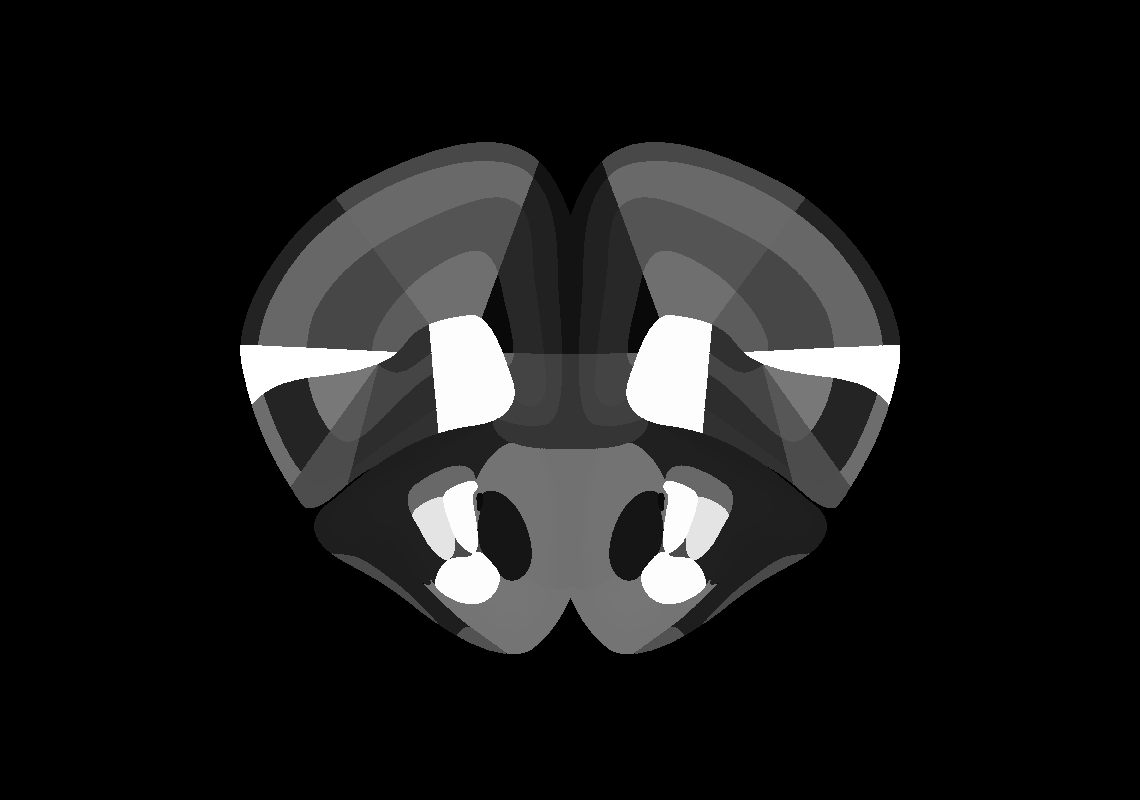

Supplement: Supplementary file 6 — Supplementary Data 4 [file 41467_2019_13057_MOESM6_ESM.zip › Suppl_File1_Labels/23_AP+2.1.tif]

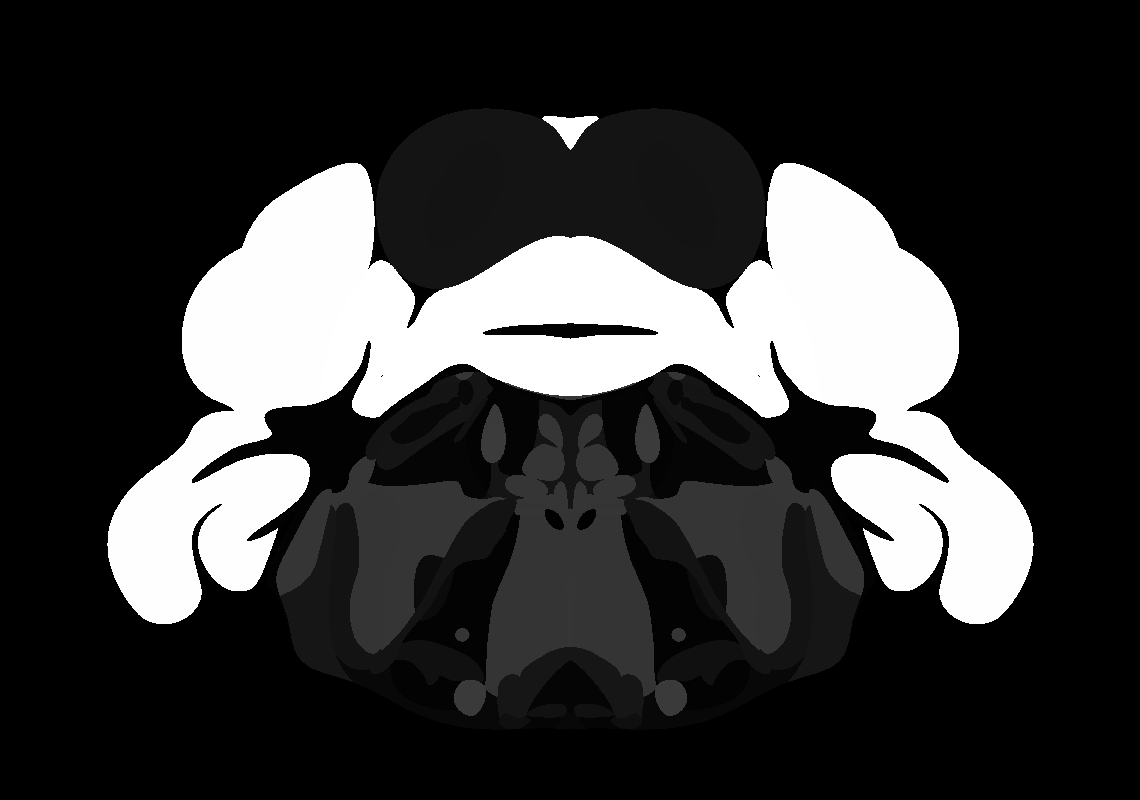

Supplement: Supplementary file 6 — Supplementary Data 4 [file 41467_2019_13057_MOESM6_ESM.zip › Suppl_File1_Labels/98_AP-5.4.tif]

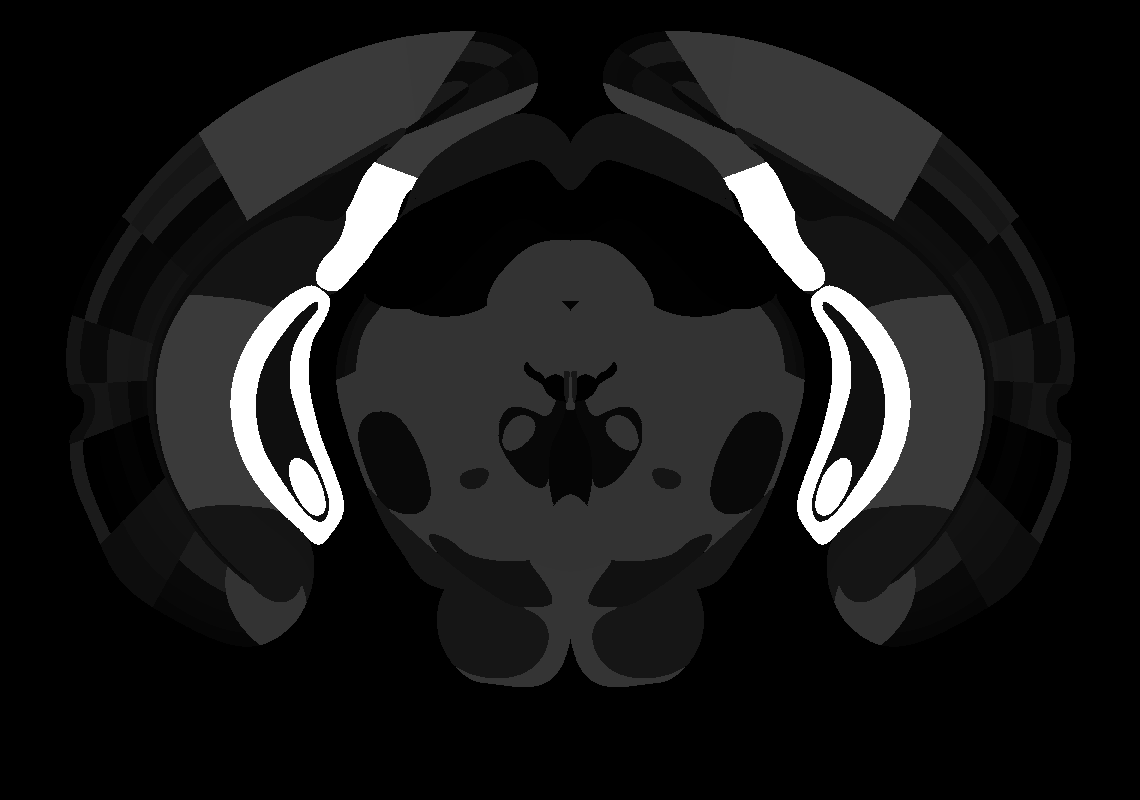

Supplement: Supplementary file 6 — Supplementary Data 4 [file 41467_2019_13057_MOESM6_ESM.zip › Suppl_File1_Labels/82_AP-3.8.tif]

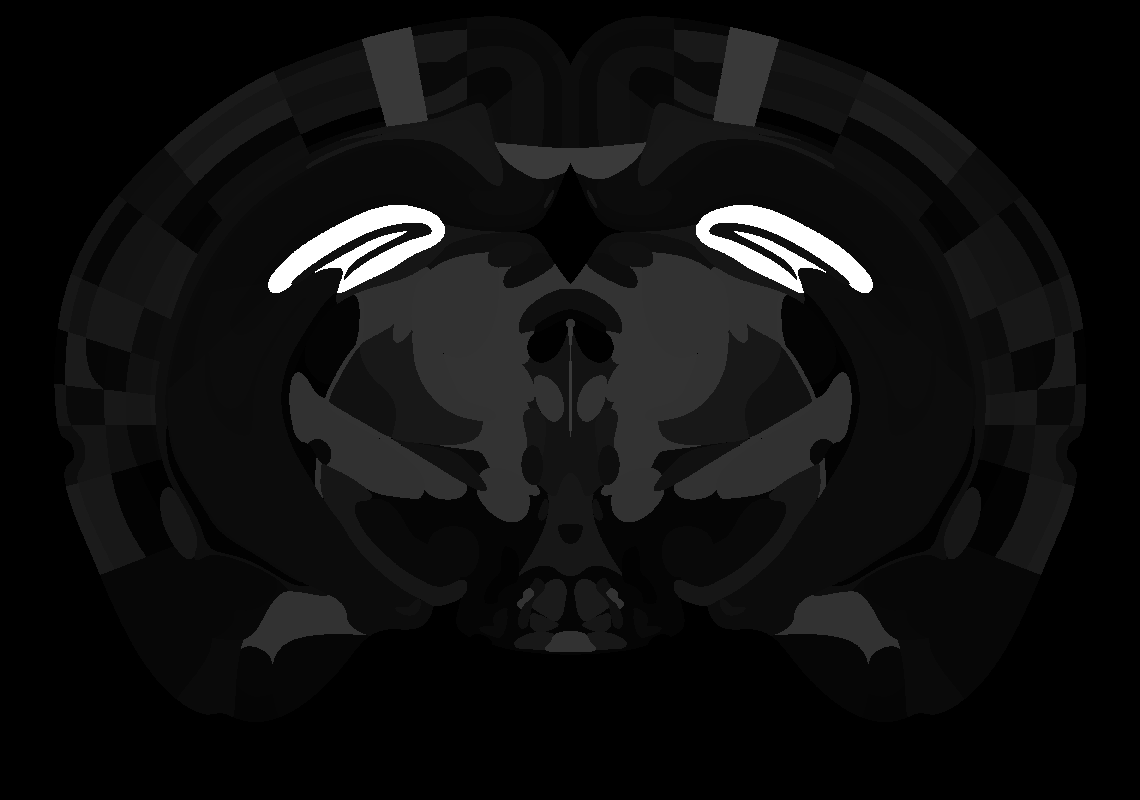

Supplement: Supplementary file 6 — Supplementary Data 4 [file 41467_2019_13057_MOESM6_ESM.zip › Suppl_File1_Labels/71_AP-2.7.tif]

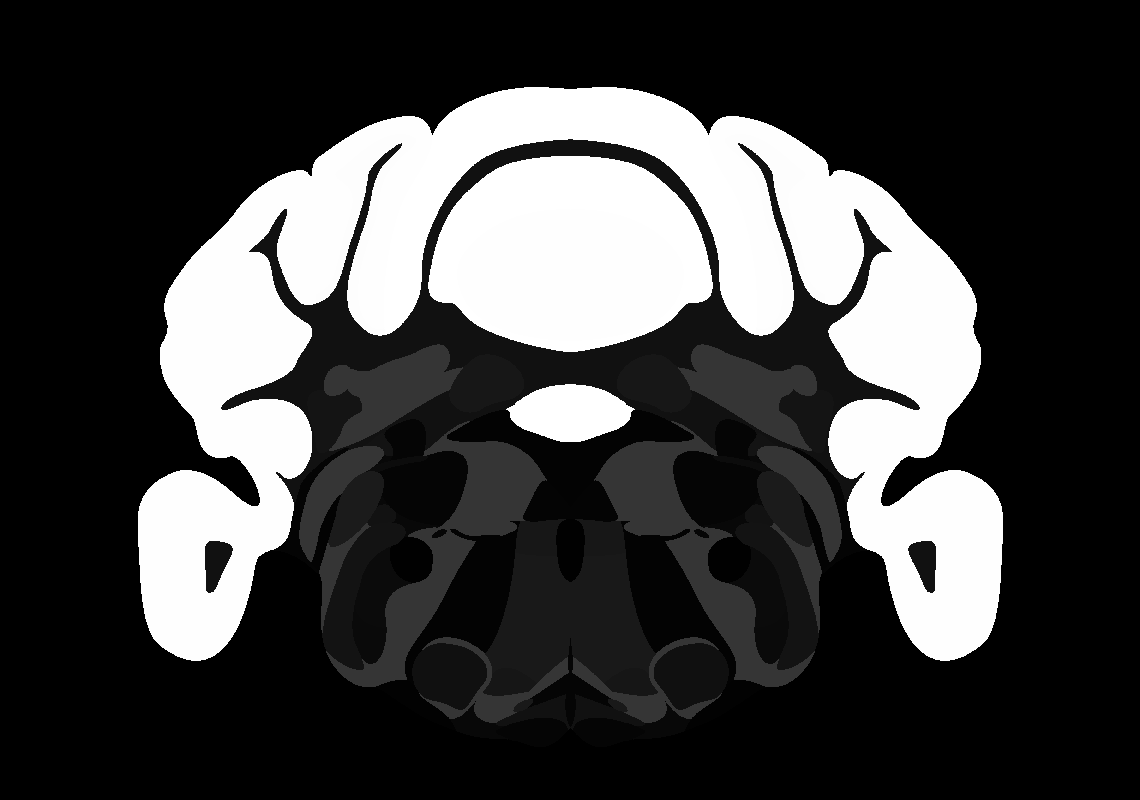

Supplement: Supplementary file 6 — Supplementary Data 4 [file 41467_2019_13057_MOESM6_ESM.zip › Suppl_File1_Labels/106_AP-6.2.tif]

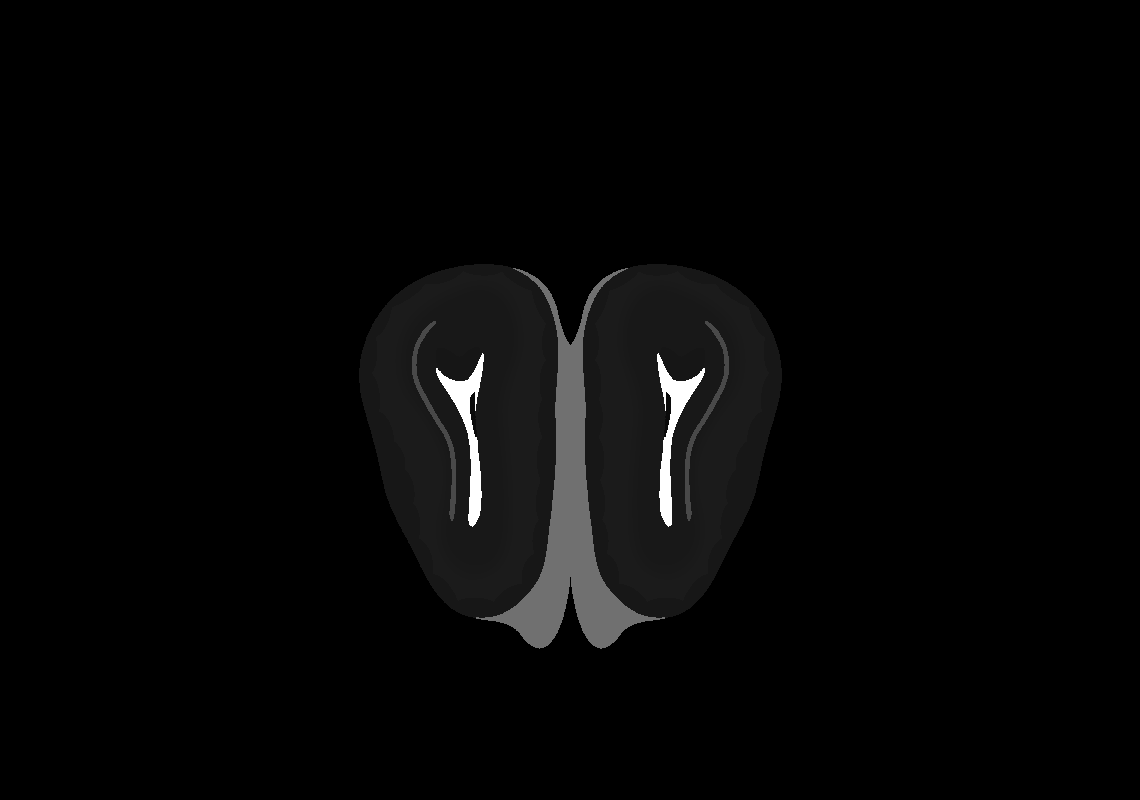

Supplement: Supplementary file 6 — Supplementary Data 4 [file 41467_2019_13057_MOESM6_ESM.zip › Suppl_File1_Labels/7_AP+3.7.tif]

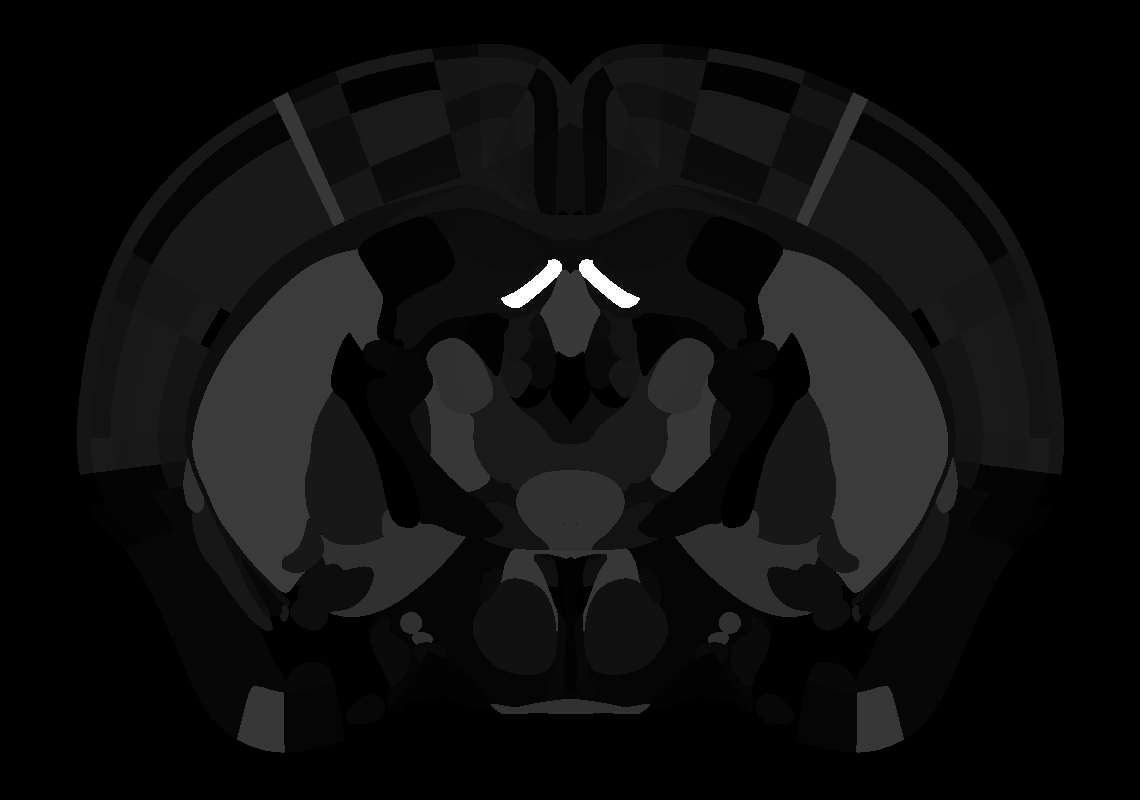

Supplement: Supplementary file 6 — Supplementary Data 4 [file 41467_2019_13057_MOESM6_ESM.zip › Suppl_File1_Labels/53_AP-0.9.tif]

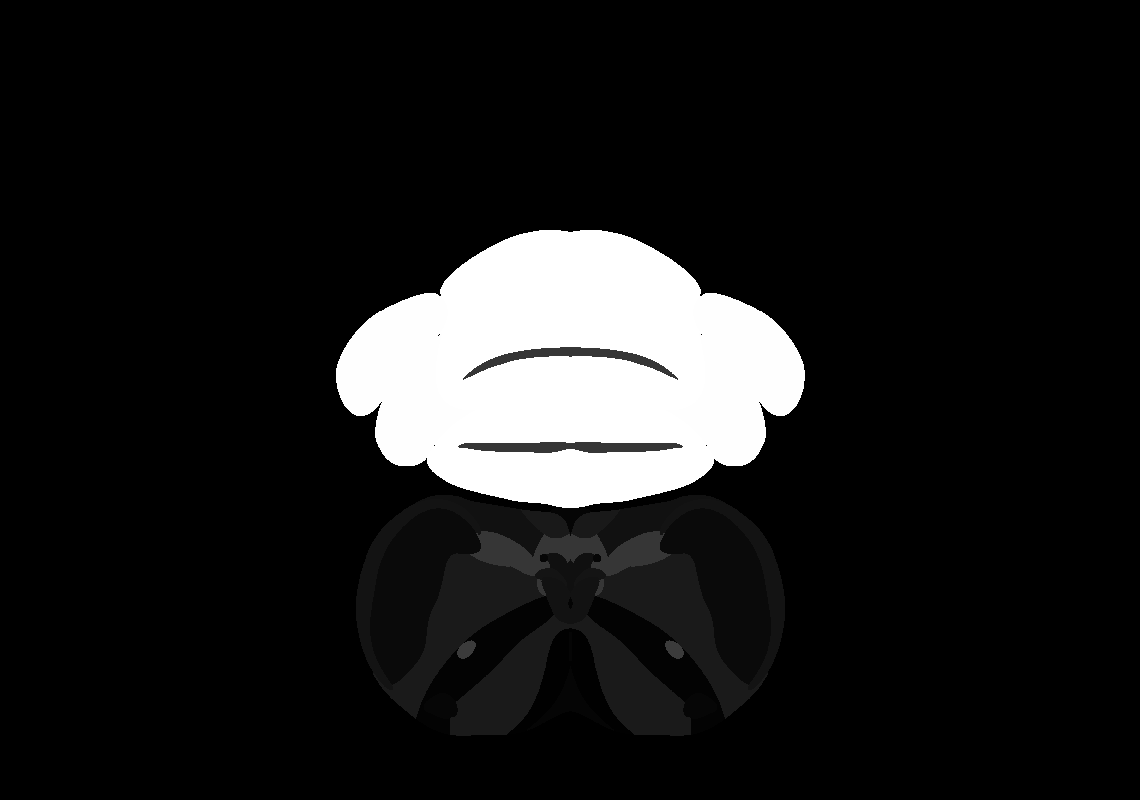

Supplement: Supplementary file 6 — Supplementary Data 4 [file 41467_2019_13057_MOESM6_ESM.zip › Suppl_File1_Labels/123_AP-7.9.tif]

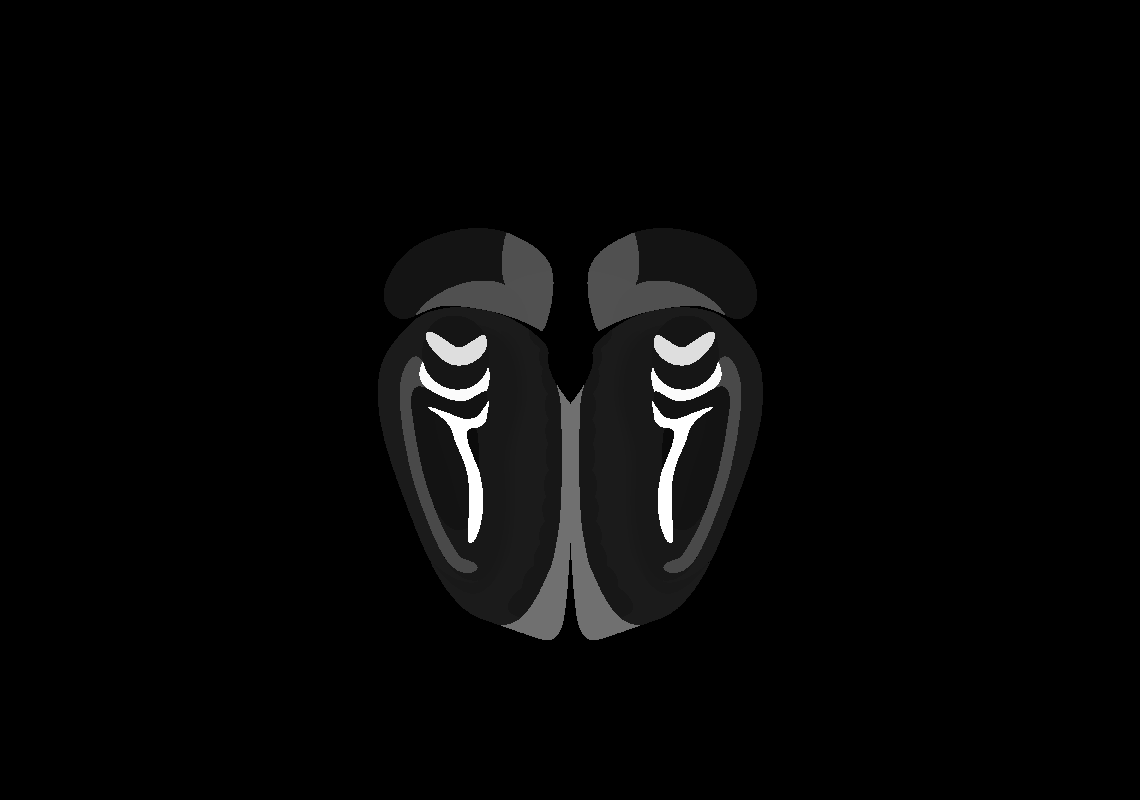

Supplement: Supplementary file 6 — Supplementary Data 4 [file 41467_2019_13057_MOESM6_ESM.zip › Suppl_File1_Labels/11_AP+3.3.tif]

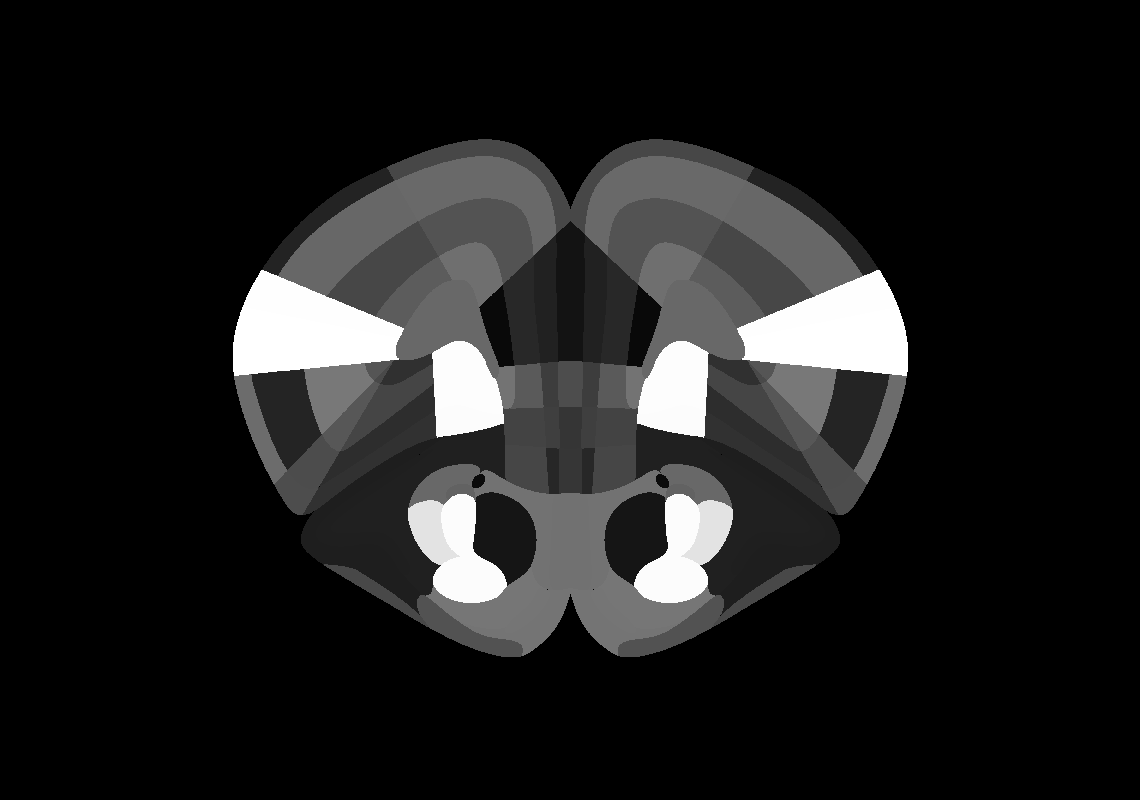

Supplement: Supplementary file 6 — Supplementary Data 4 [file 41467_2019_13057_MOESM6_ESM.zip › Suppl_File1_Labels/24_AP+2.0.tif]

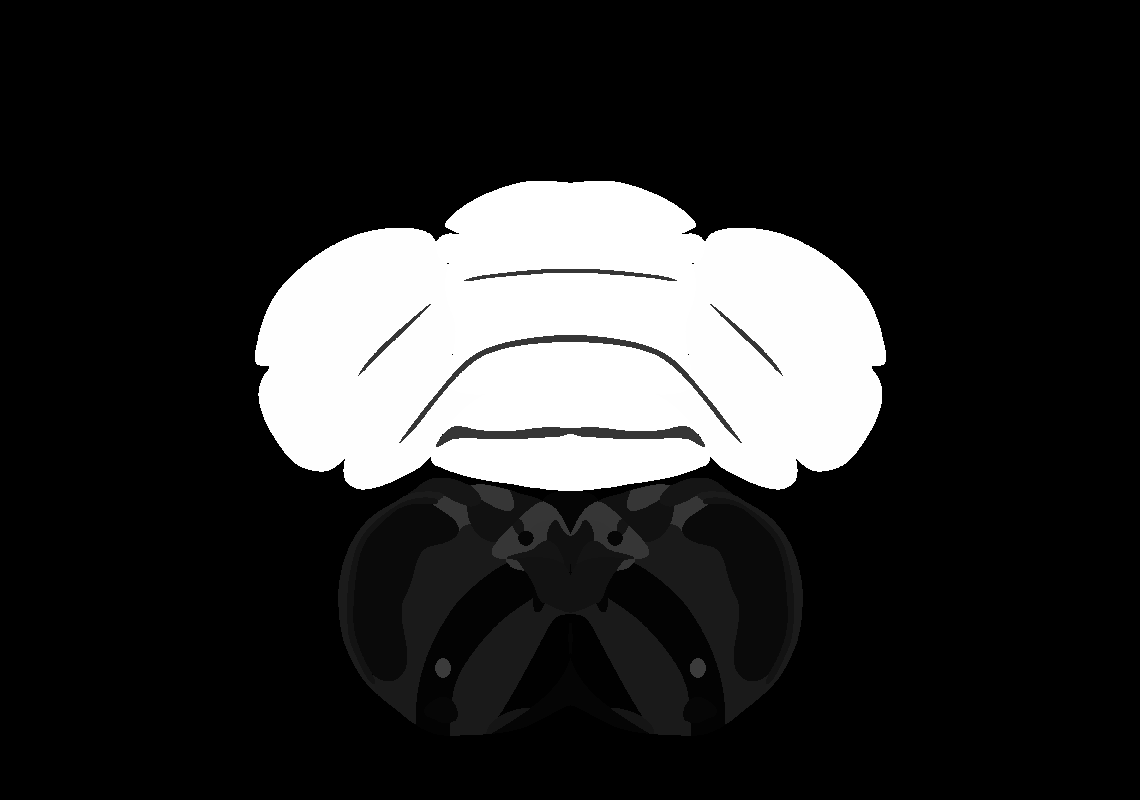

Supplement: Supplementary file 6 — Supplementary Data 4 [file 41467_2019_13057_MOESM6_ESM.zip › Suppl_File1_Labels/120_AP-7.6.tif]

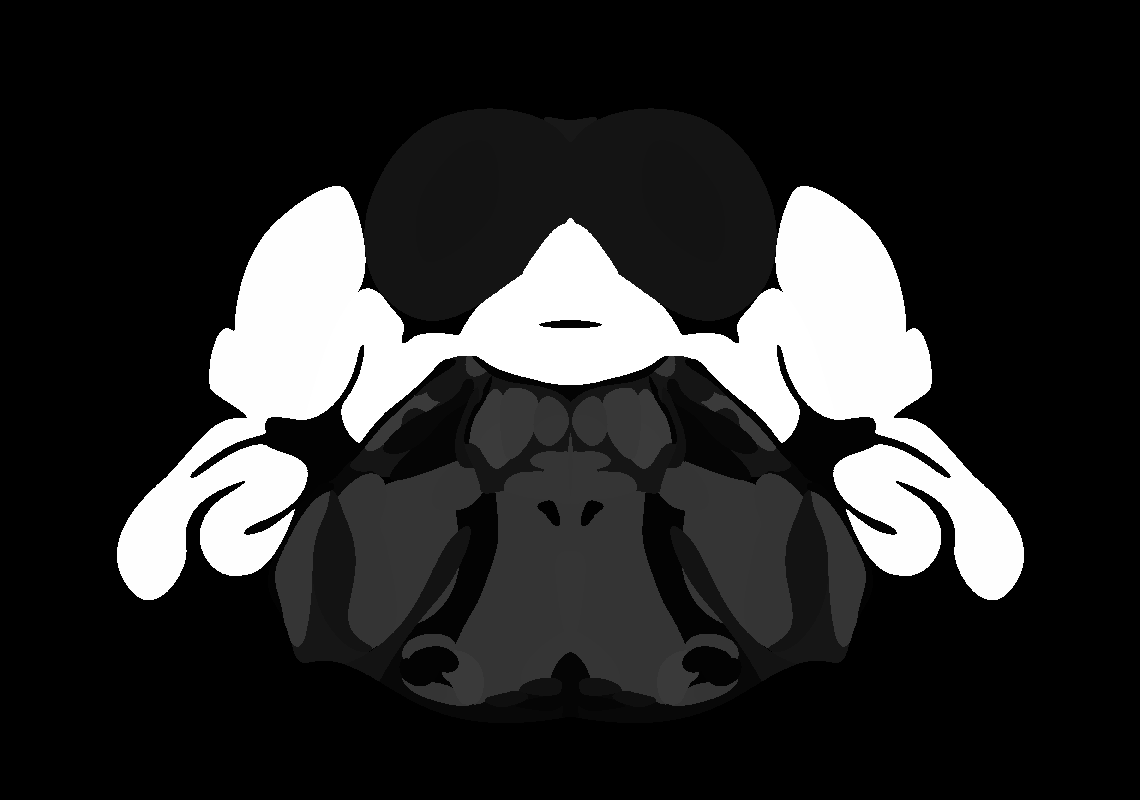

Supplement: Supplementary file 6 — Supplementary Data 4 [file 41467_2019_13057_MOESM6_ESM.zip › Suppl_File1_Labels/97_AP-5.3.tif]

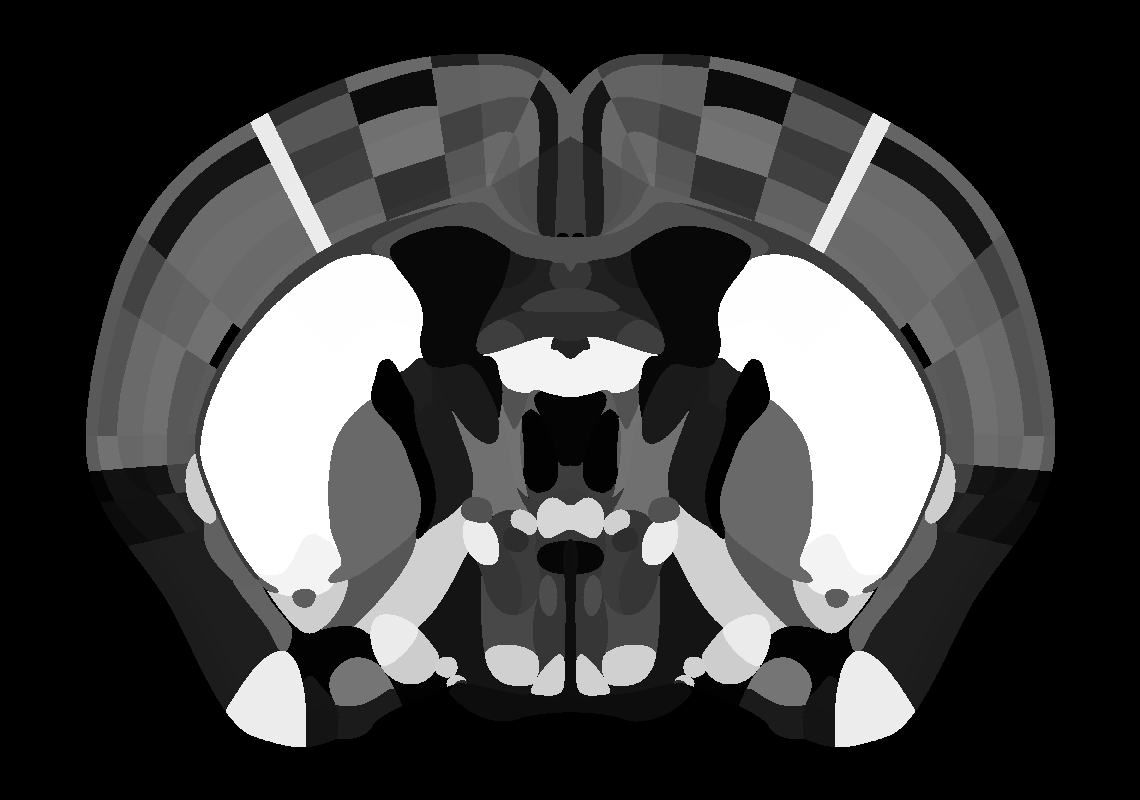

Supplement: Supplementary file 6 — Supplementary Data 4 [file 41467_2019_13057_MOESM6_ESM.zip › Suppl_File1_Labels/50_AP-0.6.tif]

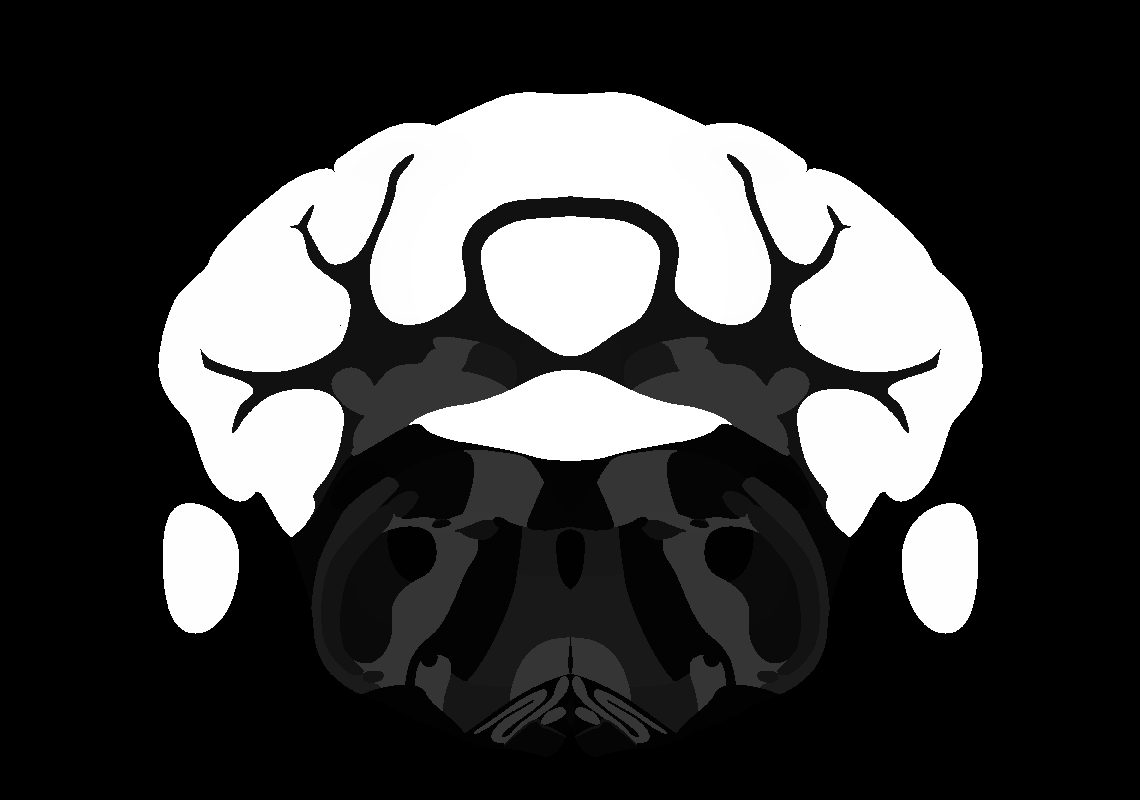

Supplement: Supplementary file 6 — Supplementary Data 4 [file 41467_2019_13057_MOESM6_ESM.zip › Suppl_File1_Labels/109_AP-6.5.tif]

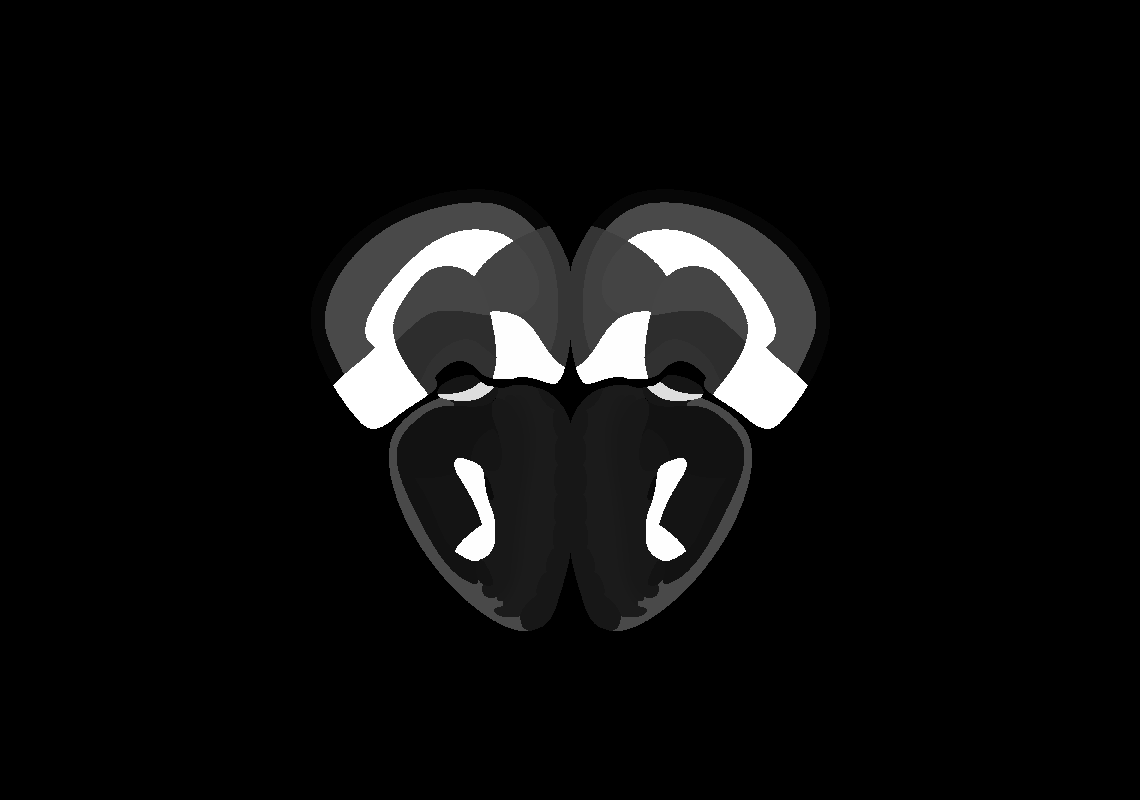

Supplement: Supplementary file 6 — Supplementary Data 4 [file 41467_2019_13057_MOESM6_ESM.zip › Suppl_File1_Labels/15_AP+2.9.tif]

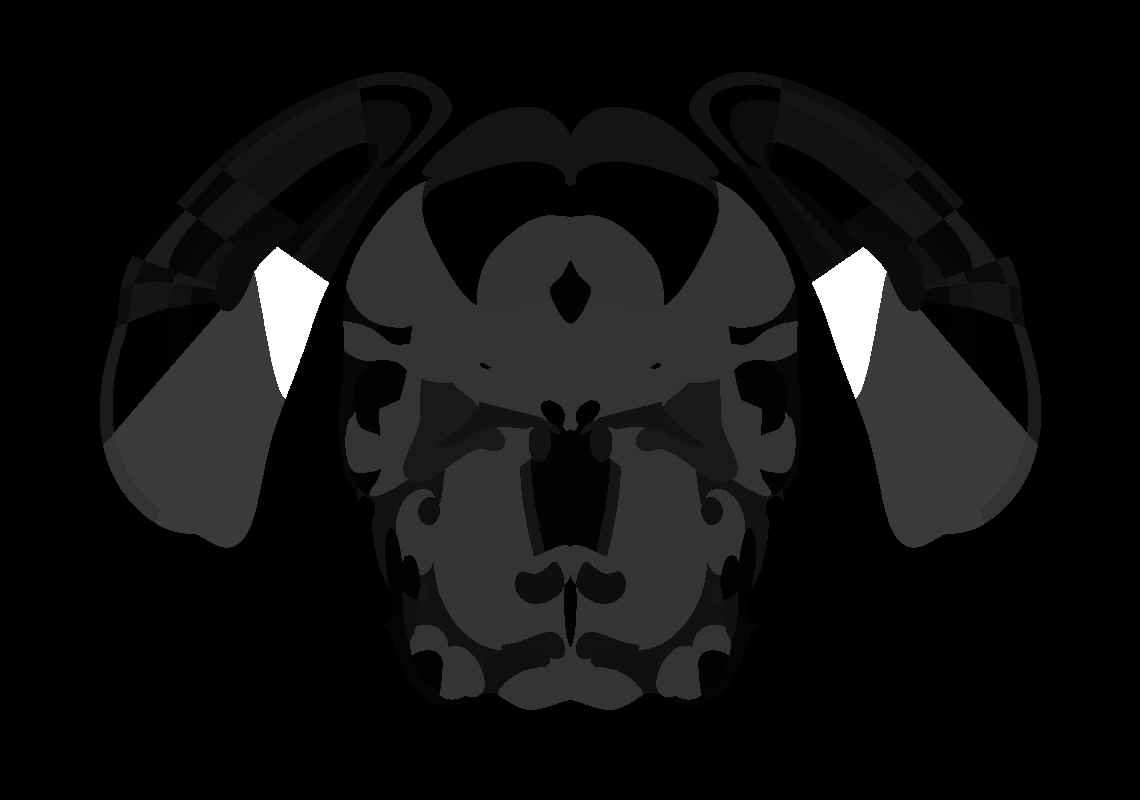

Supplement: Supplementary file 6 — Supplementary Data 4 [file 41467_2019_13057_MOESM6_ESM.zip › Suppl_File1_Labels/90_AP-4.6.tif]

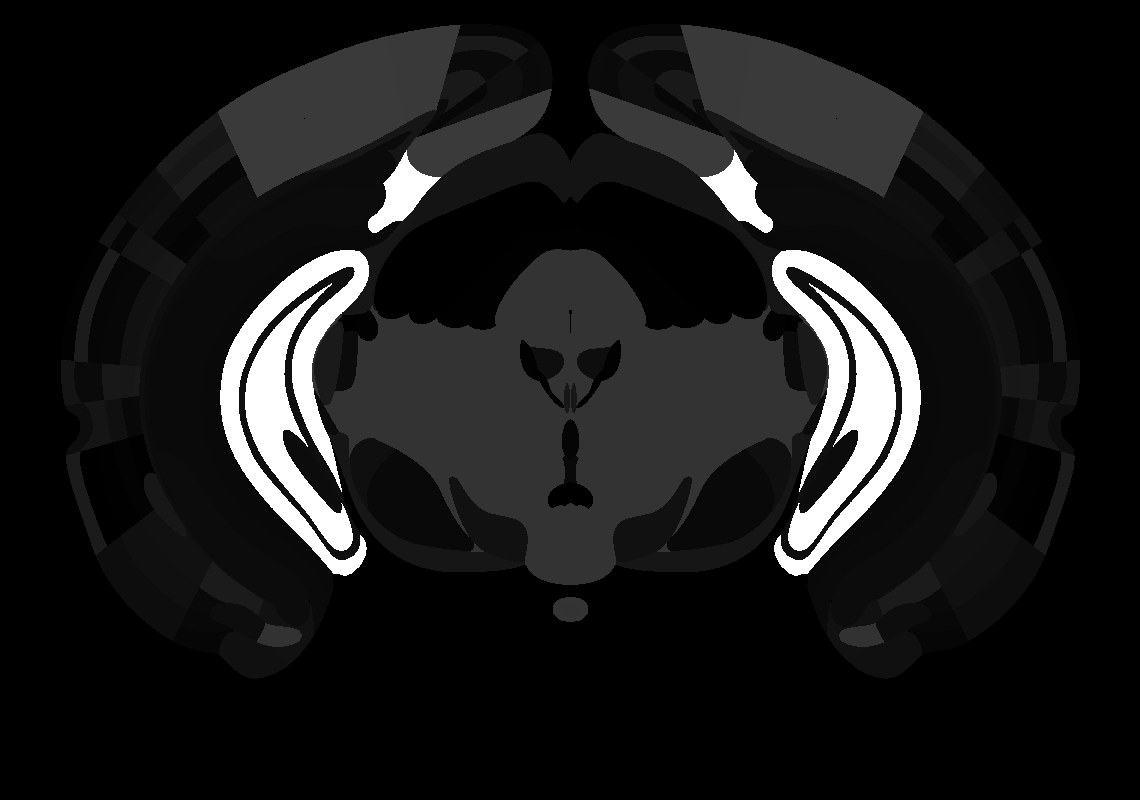

Supplement: Supplementary file 6 — Supplementary Data 4 [file 41467_2019_13057_MOESM6_ESM.zip › Suppl_File1_Labels/79_AP-3.5.tif]

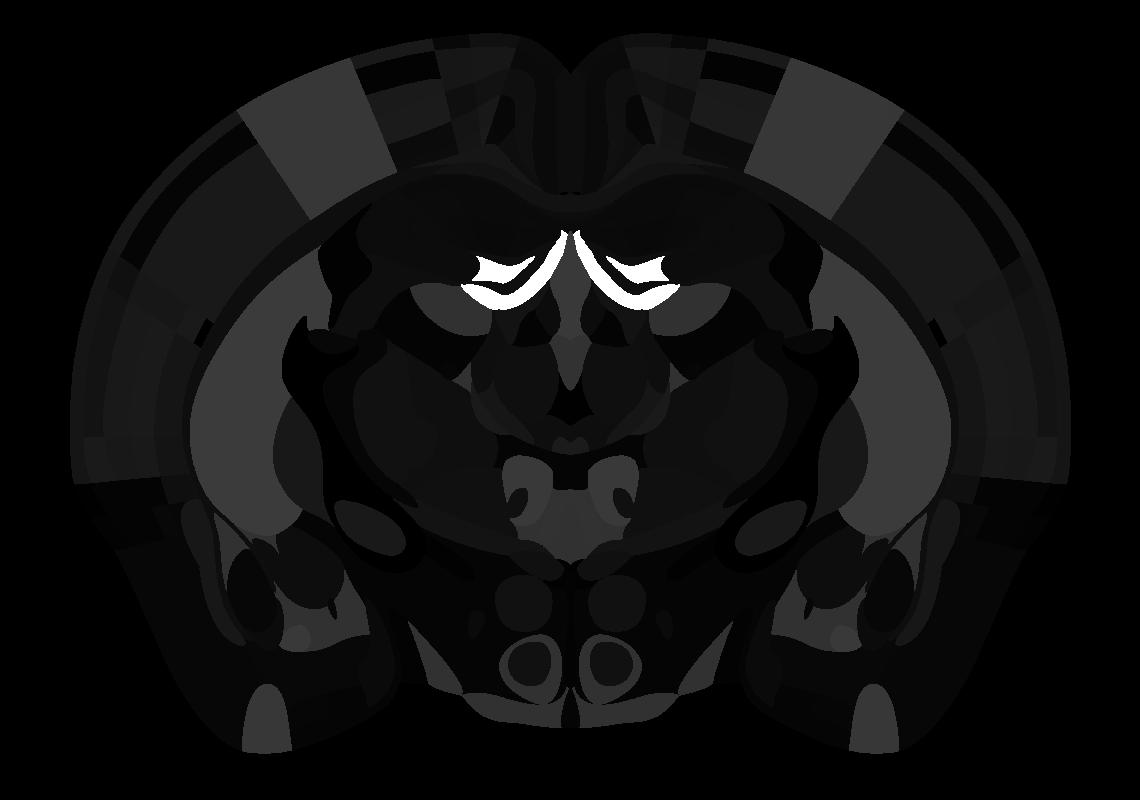

Supplement: Supplementary file 6 — Supplementary Data 4 [file 41467_2019_13057_MOESM6_ESM.zip › Suppl_File1_Labels/57_AP-1.3.tif]

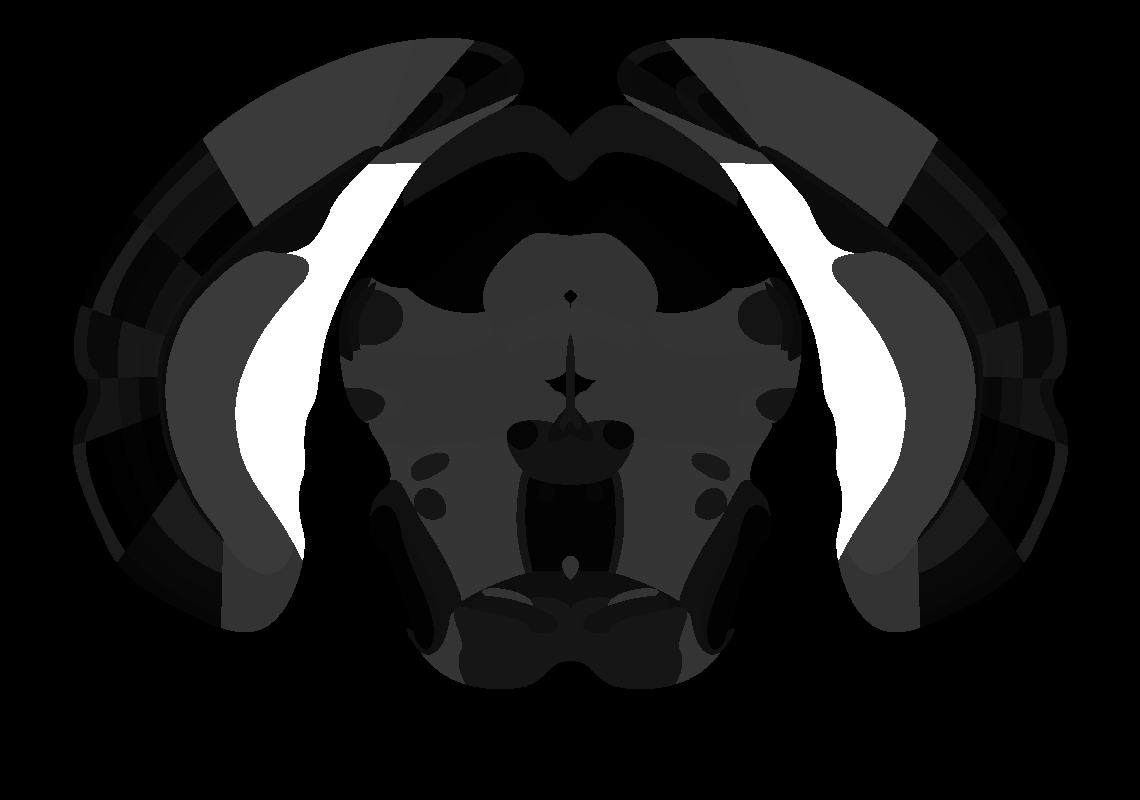

Supplement: Supplementary file 6 — Supplementary Data 4 [file 41467_2019_13057_MOESM6_ESM.zip › Suppl_File1_Labels/84_AP-4.0.tif]

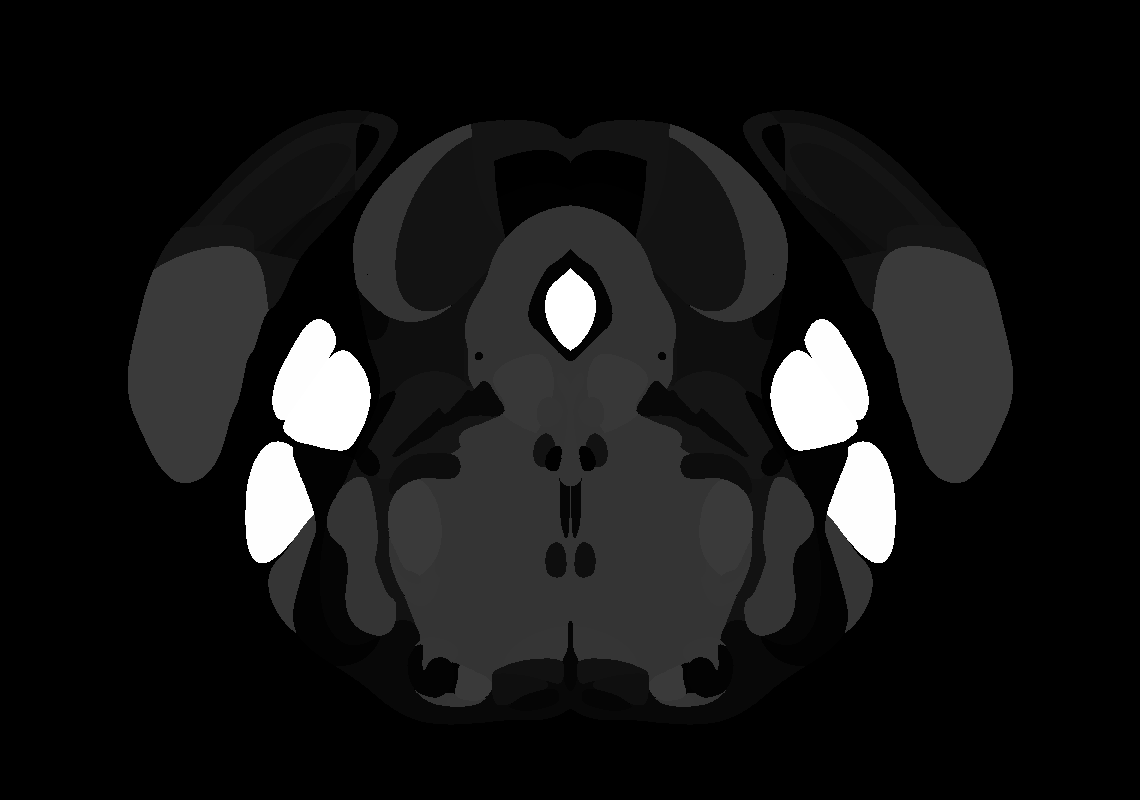

Supplement: Supplementary file 6 — Supplementary Data 4 [file 41467_2019_13057_MOESM6_ESM.zip › Suppl_File1_Labels/93_AP-4.9.tif]

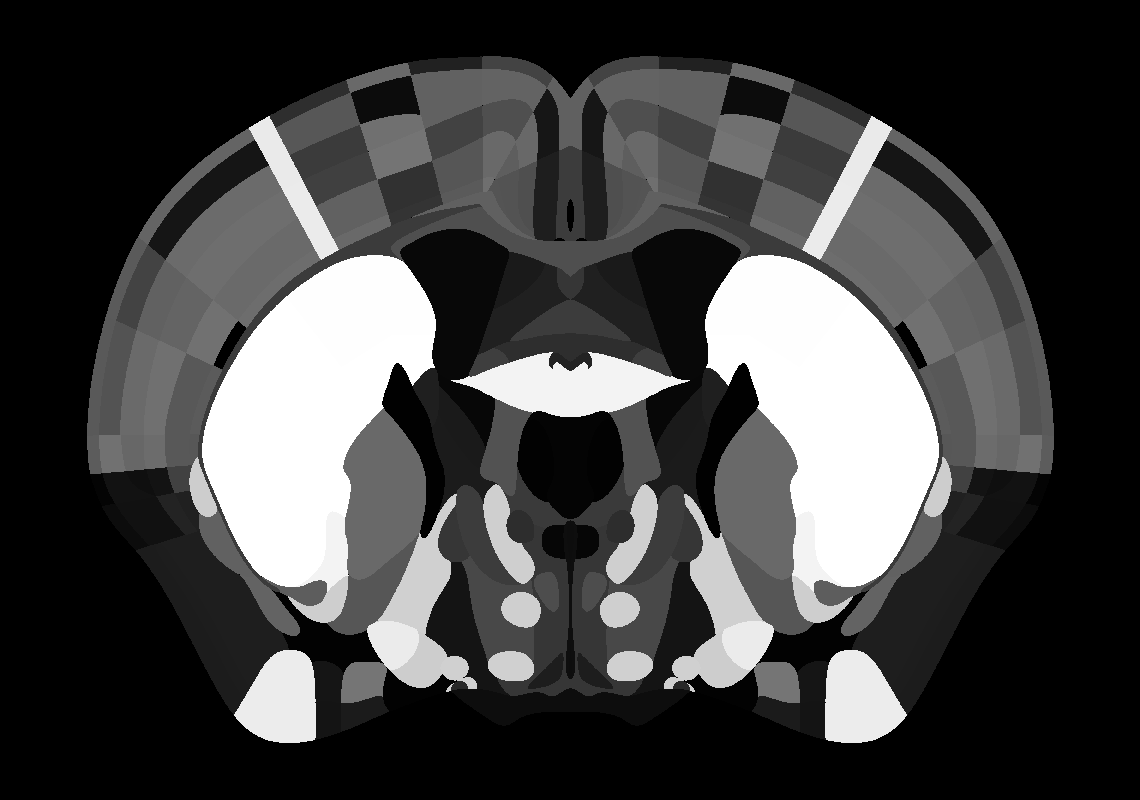

Supplement: Supplementary file 6 — Supplementary Data 4 [file 41467_2019_13057_MOESM6_ESM.zip › Suppl_File1_Labels/49_AP-0.5.tif]

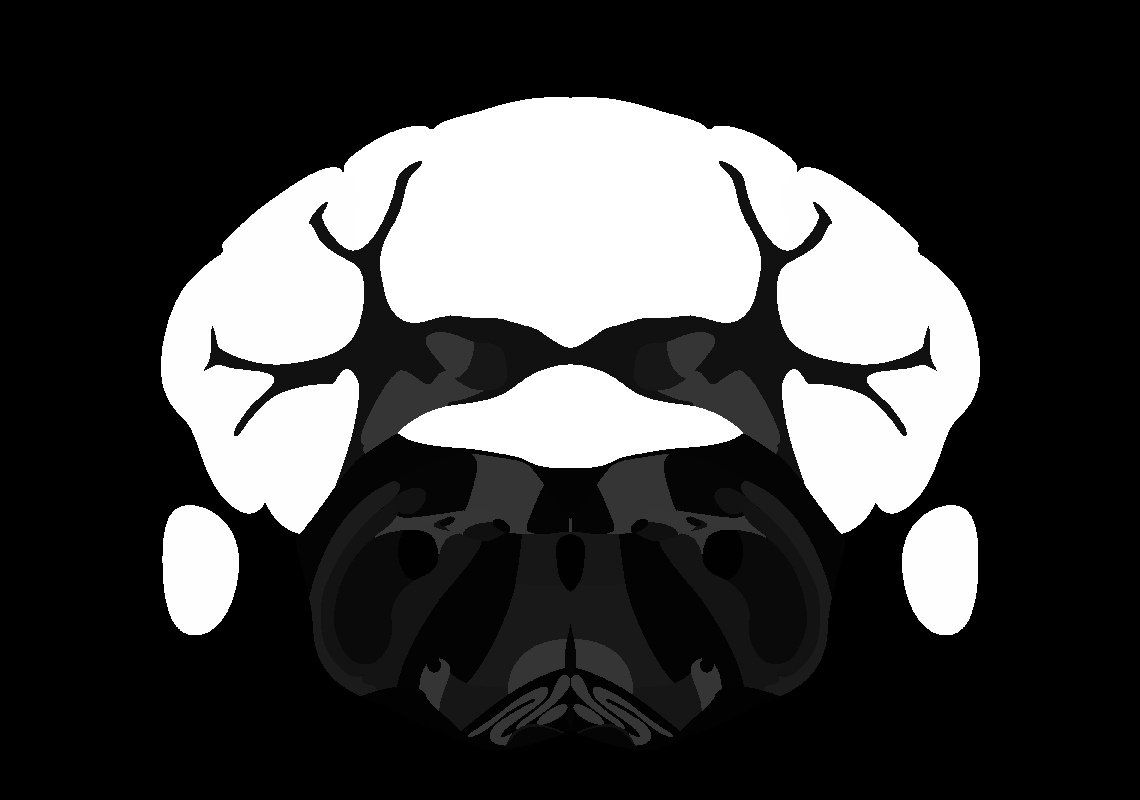

Supplement: Supplementary file 6 — Supplementary Data 4 [file 41467_2019_13057_MOESM6_ESM.zip › Suppl_File1_Labels/110_AP-6.6.tif]

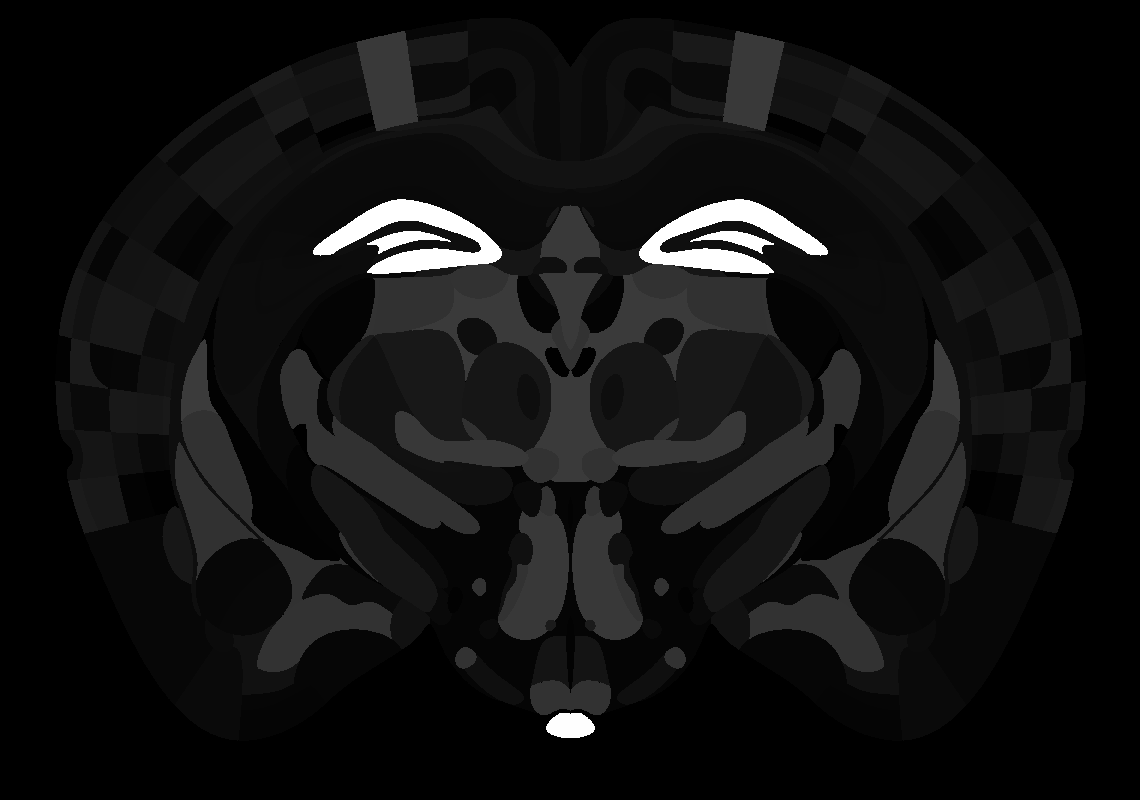

Supplement: Supplementary file 6 — Supplementary Data 4 [file 41467_2019_13057_MOESM6_ESM.zip › Suppl_File1_Labels/67_AP-2.3.tif]

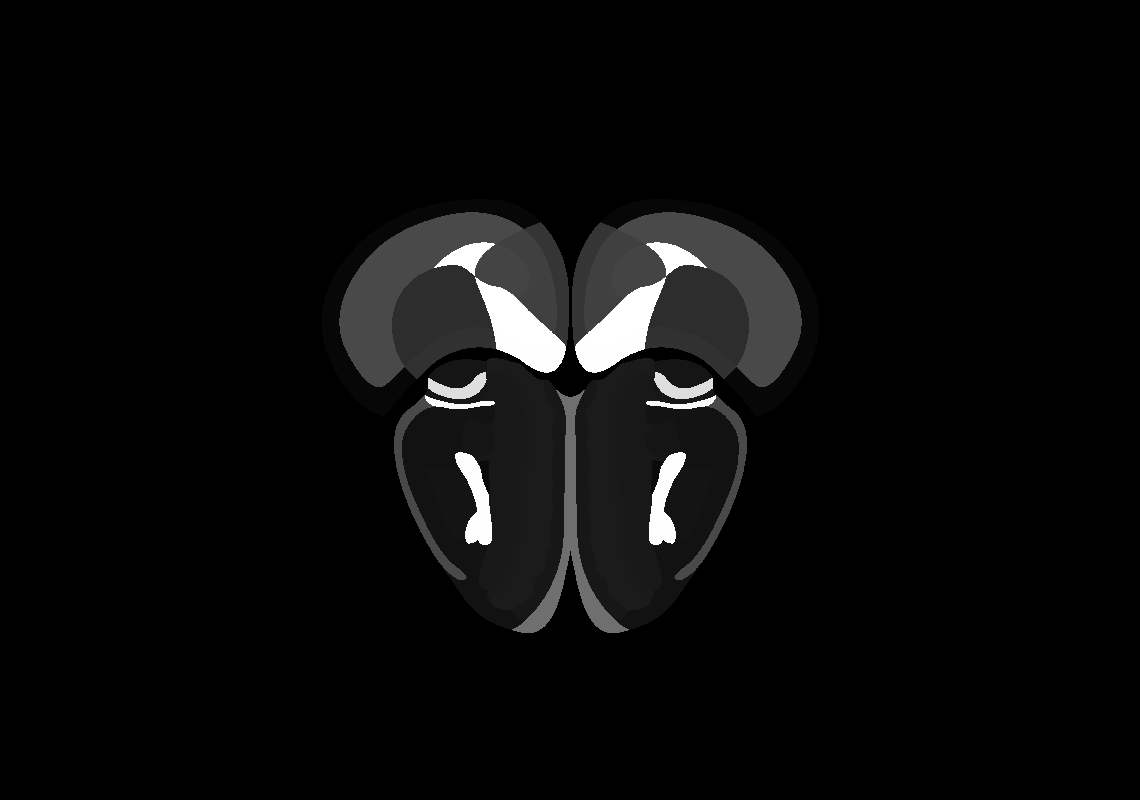

Supplement: Supplementary file 6 — Supplementary Data 4 [file 41467_2019_13057_MOESM6_ESM.zip › Suppl_File1_Labels/14_AP+3.0.tif]

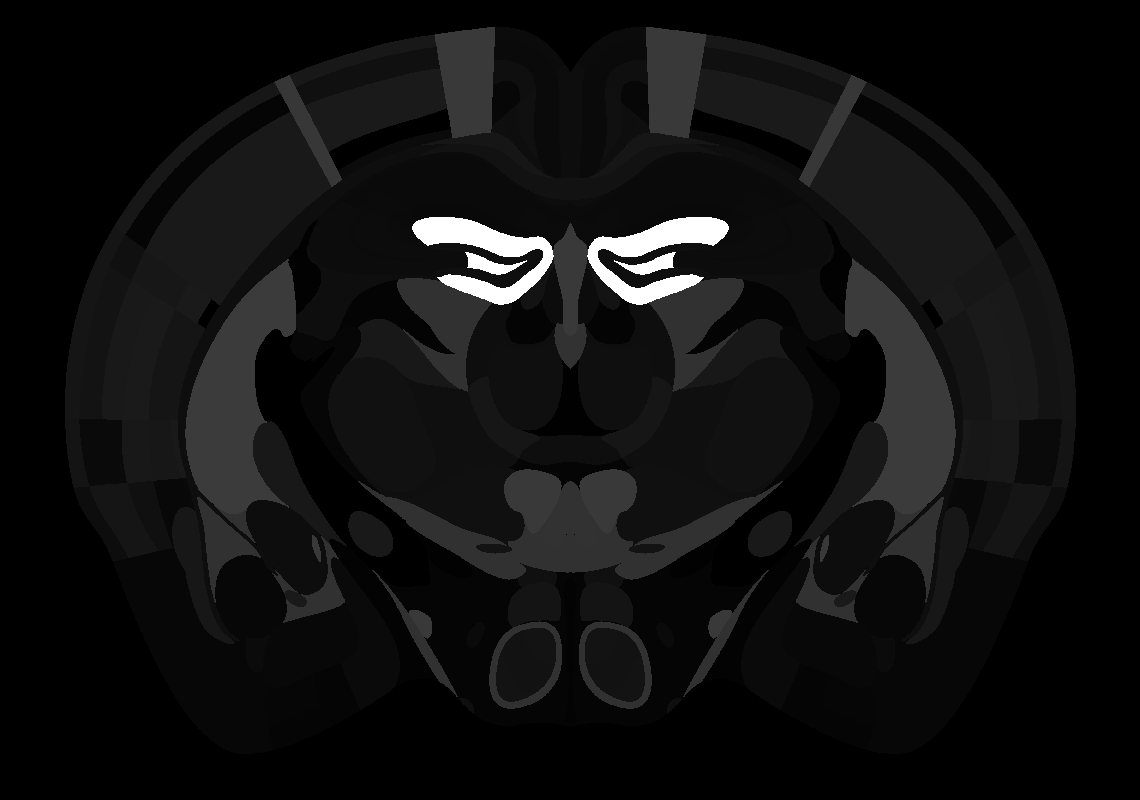

Supplement: Supplementary file 6 — Supplementary Data 4 [file 41467_2019_13057_MOESM6_ESM.zip › Suppl_File1_Labels/60_AP-1.6.tif]

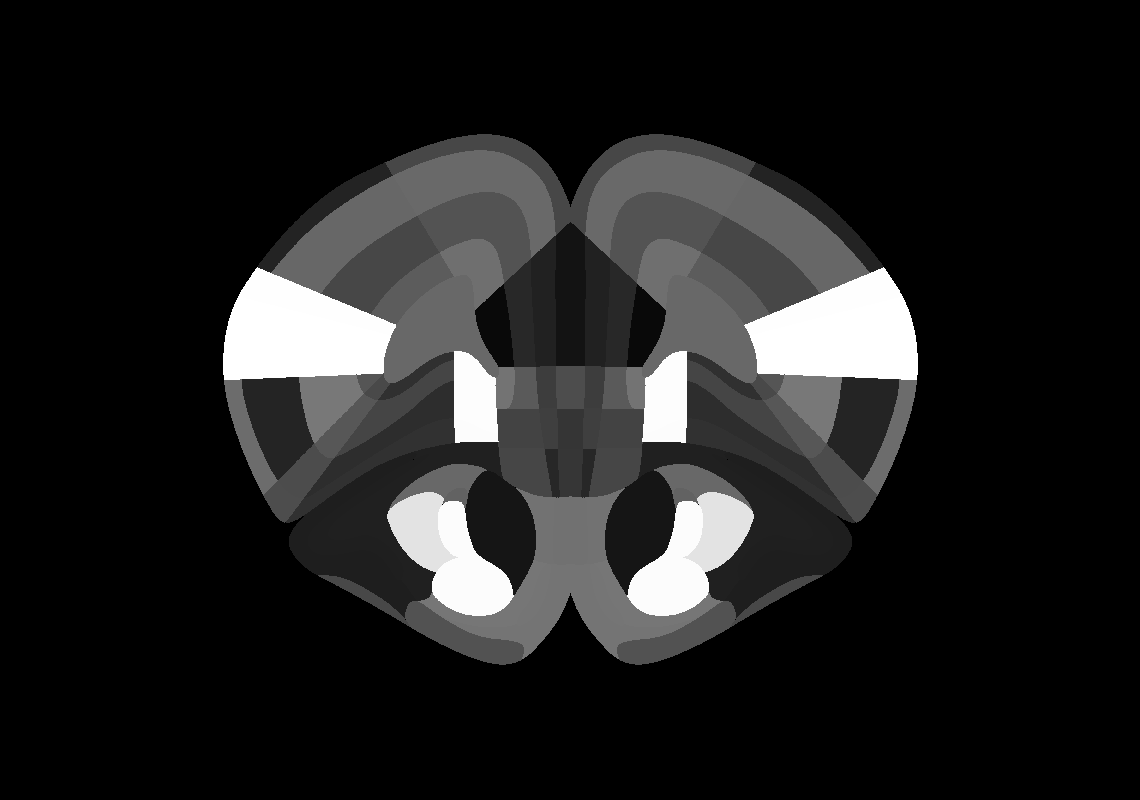

Supplement: Supplementary file 6 — Supplementary Data 4 [file 41467_2019_13057_MOESM6_ESM.zip › Suppl_File1_Labels/25_AP+1.9.tif]

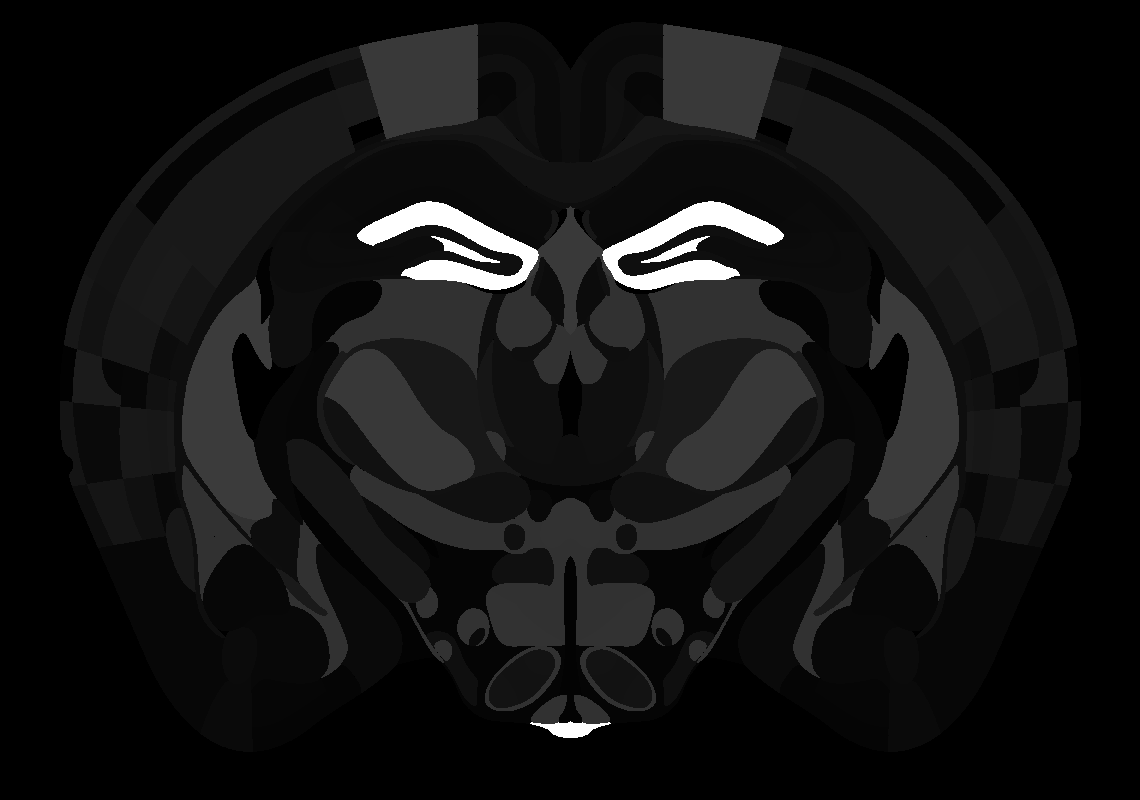

Supplement: Supplementary file 6 — Supplementary Data 4 [file 41467_2019_13057_MOESM6_ESM.zip › Suppl_File1_Labels/63_AP-1.9.tif]

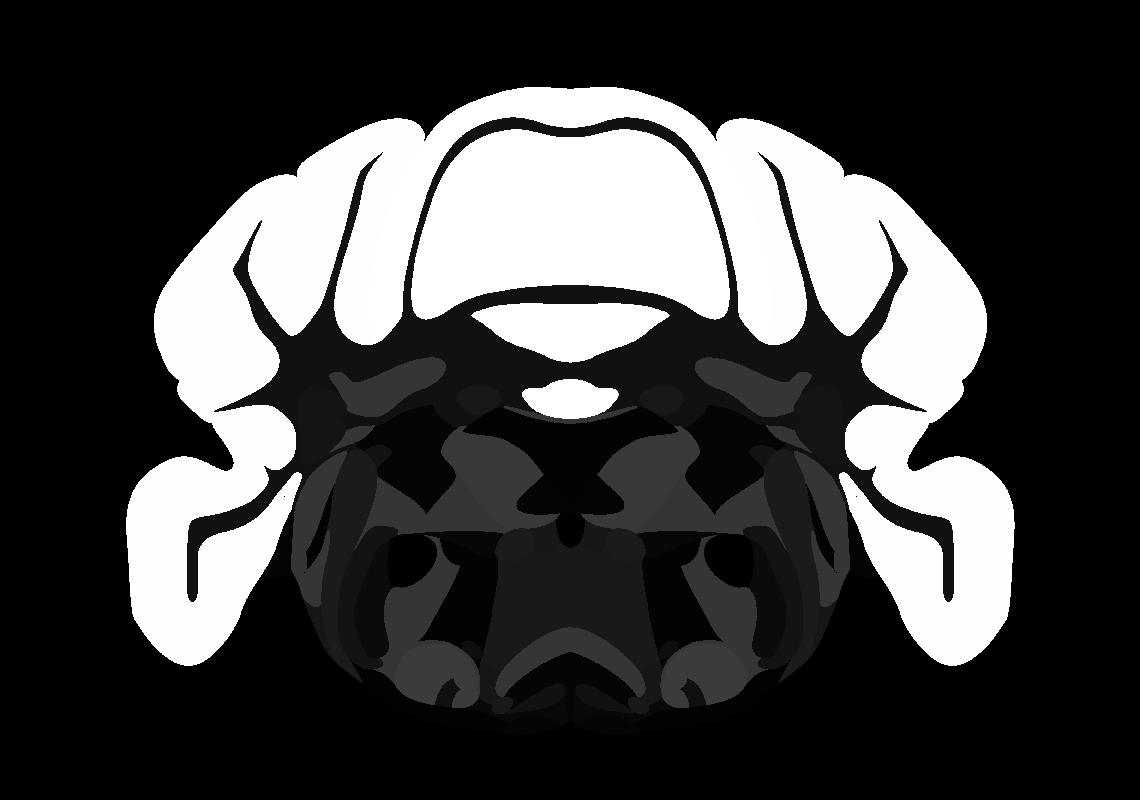

Supplement: Supplementary file 6 — Supplementary Data 4 [file 41467_2019_13057_MOESM6_ESM.zip › Suppl_File1_Labels/104_AP-6.0.tif]

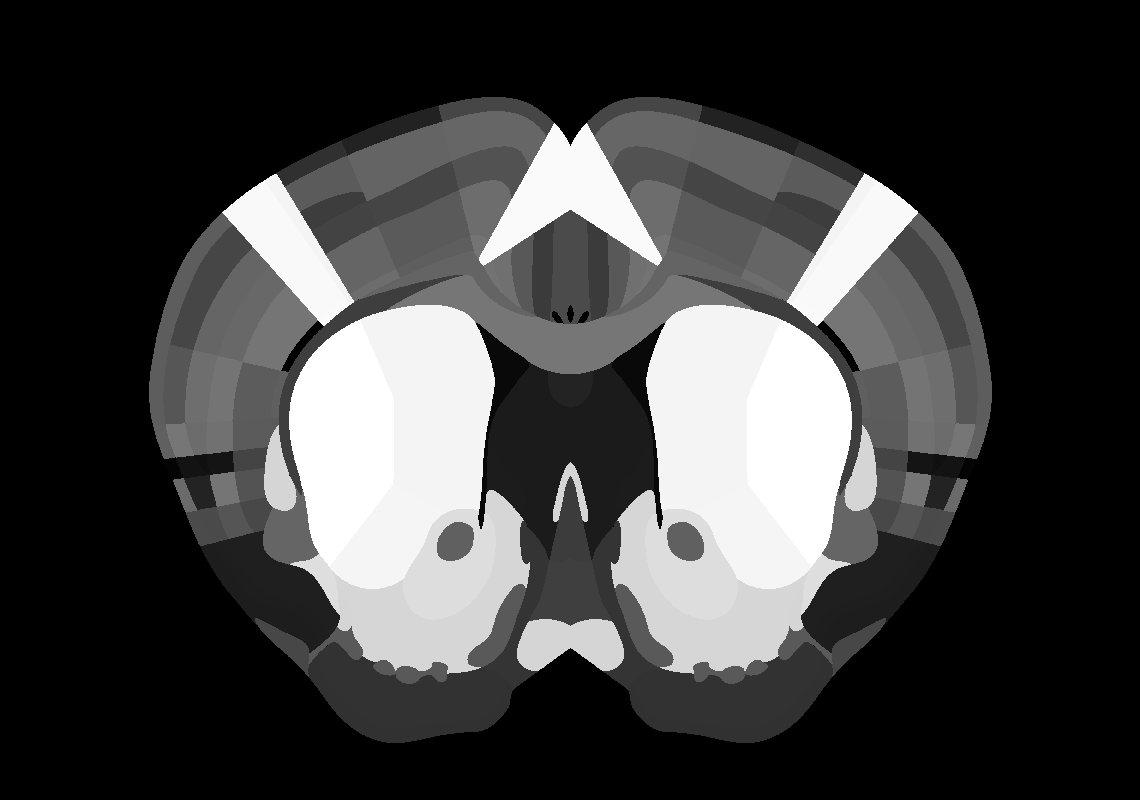

Supplement: Supplementary file 6 — Supplementary Data 4 [file 41467_2019_13057_MOESM6_ESM.zip › Suppl_File1_Labels/36_AP+0.8.tif]

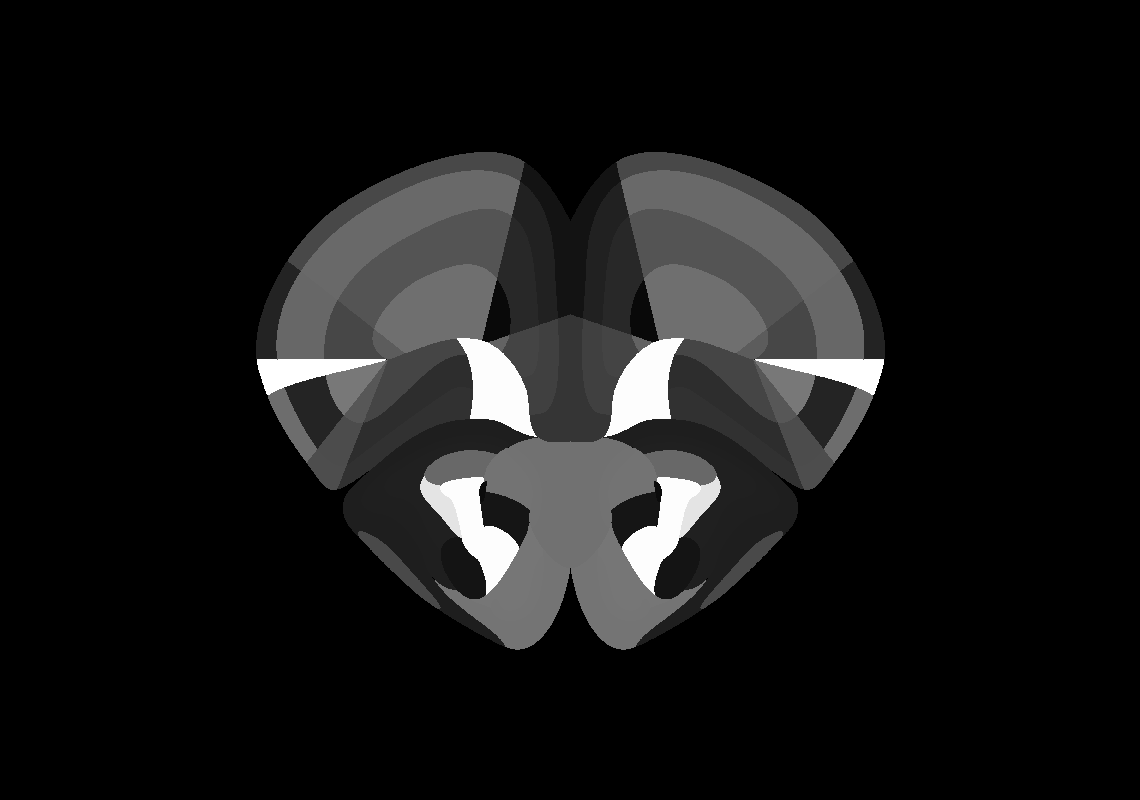

Supplement: Supplementary file 6 — Supplementary Data 4 [file 41467_2019_13057_MOESM6_ESM.zip › Suppl_File1_Labels/21_AP+2.3.tif]

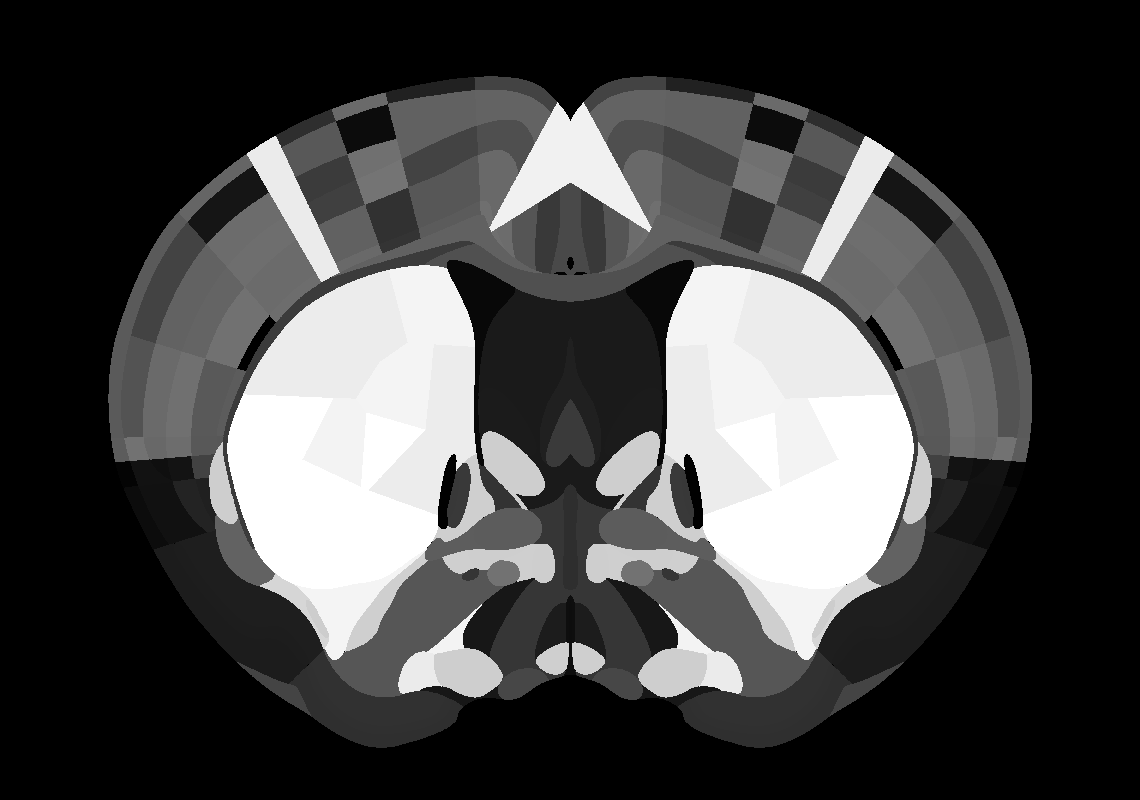

Supplement: Supplementary file 6 — Supplementary Data 4 [file 41467_2019_13057_MOESM6_ESM.zip › Suppl_File1_Labels/43_AP+0.1.tif]

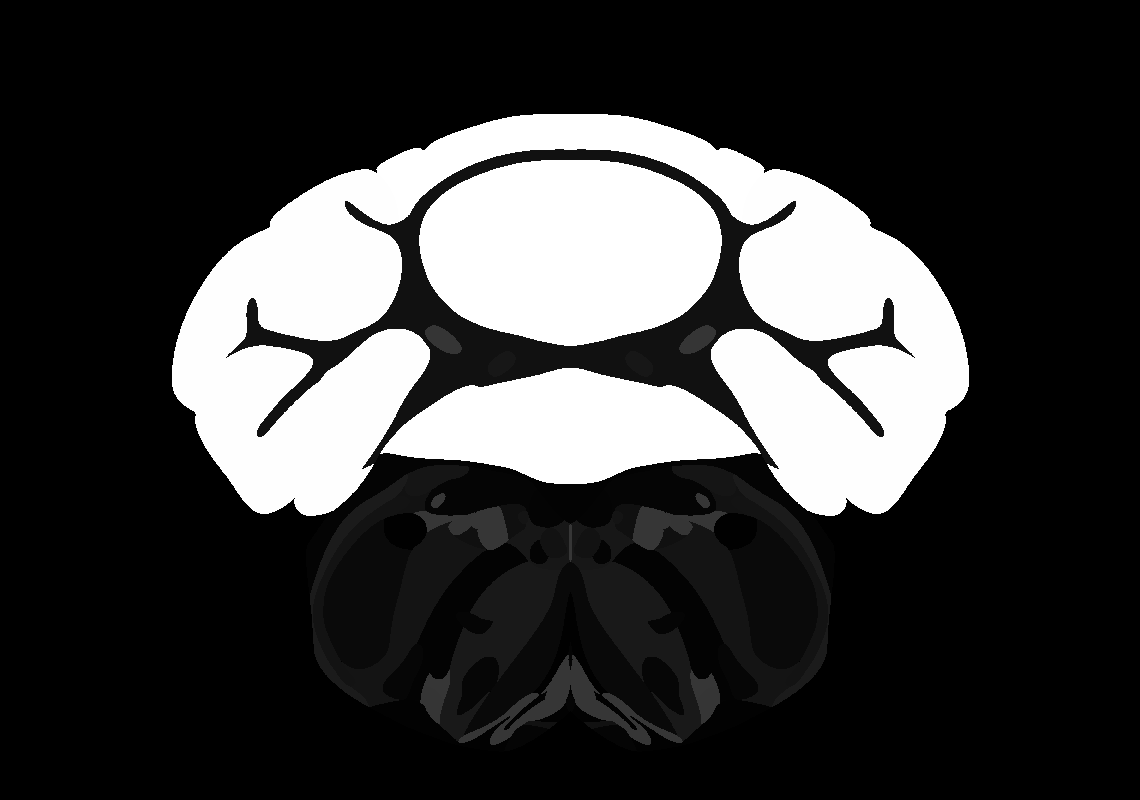

Supplement: Supplementary file 6 — Supplementary Data 4 [file 41467_2019_13057_MOESM6_ESM.zip › Suppl_File1_Labels/113_AP-6.9.tif]

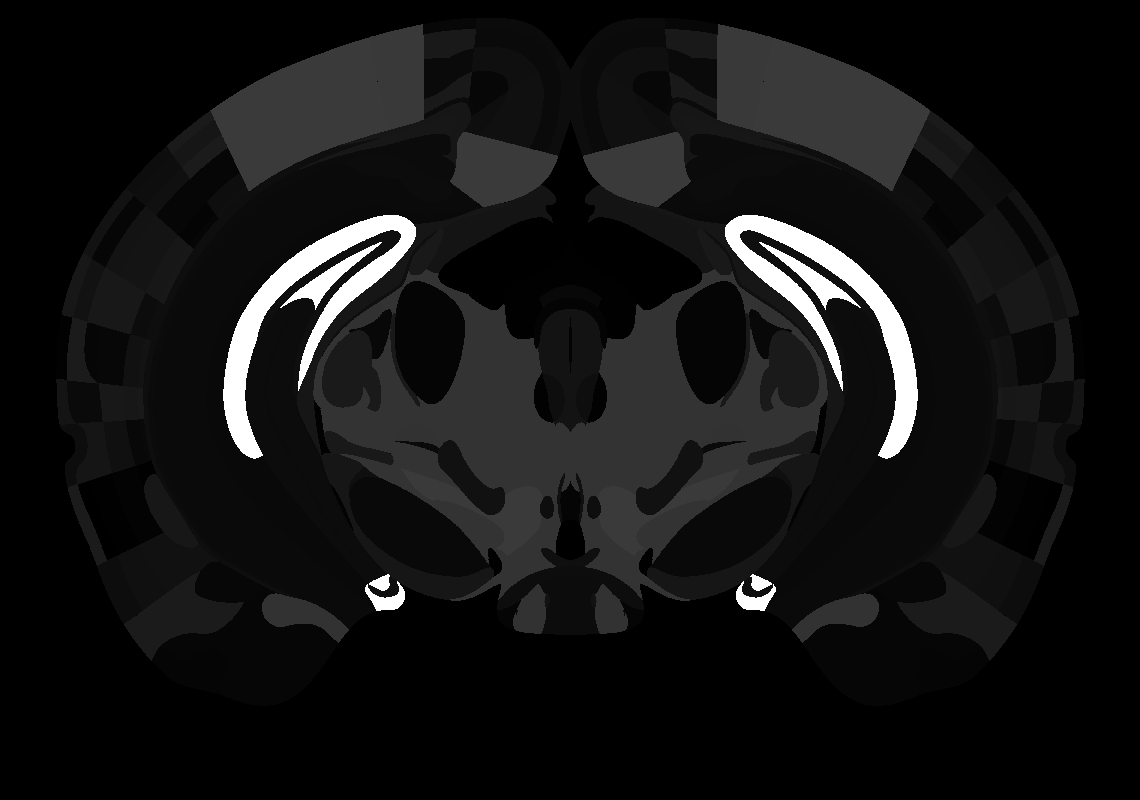

Supplement: Supplementary file 6 — Supplementary Data 4 [file 41467_2019_13057_MOESM6_ESM.zip › Suppl_File1_Labels/74_AP-3.0.tif]

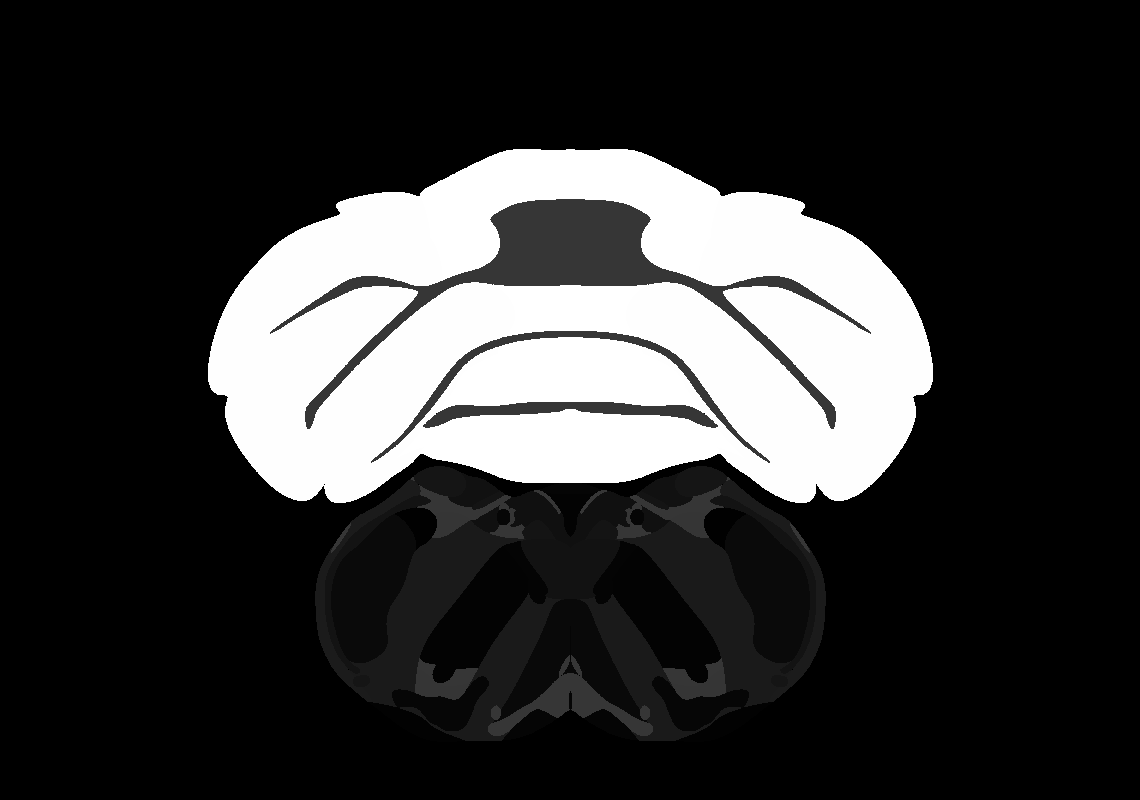

Supplement: Supplementary file 6 — Supplementary Data 4 [file 41467_2019_13057_MOESM6_ESM.zip › Suppl_File1_Labels/117_AP-7.3.tif]

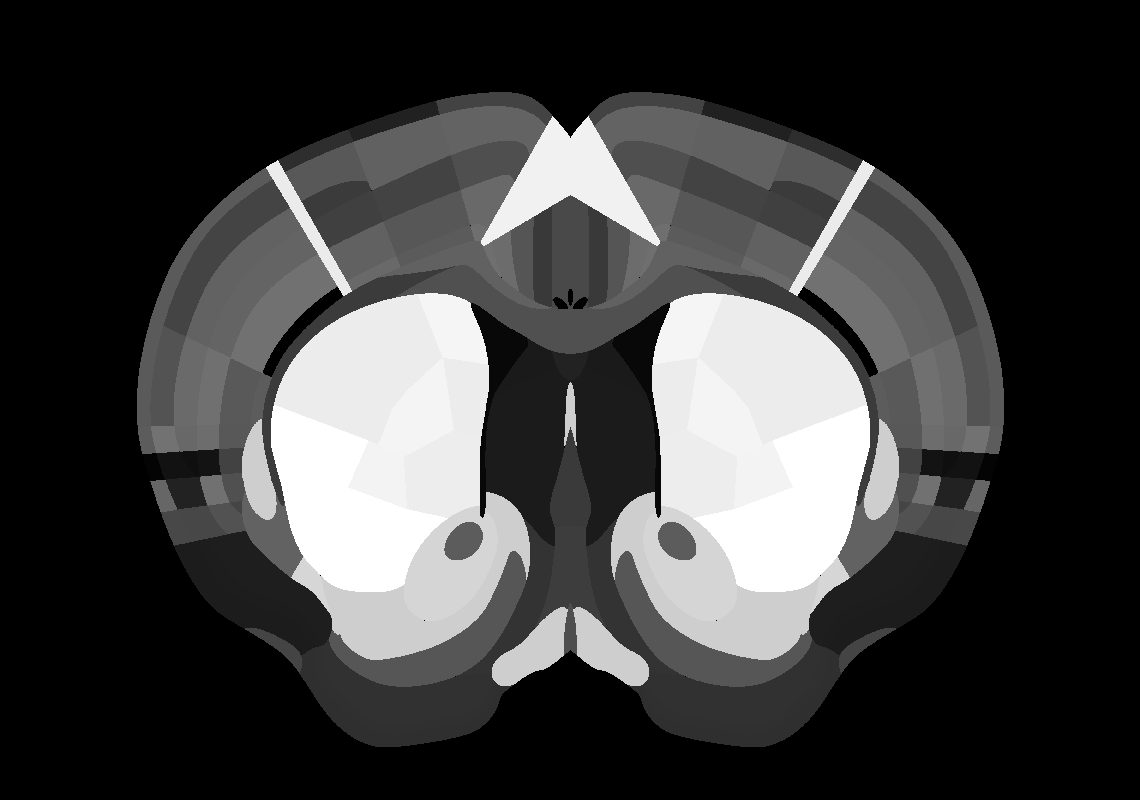

Supplement: Supplementary file 6 — Supplementary Data 4 [file 41467_2019_13057_MOESM6_ESM.zip › Suppl_File1_Labels/38_AP+0.6.tif]

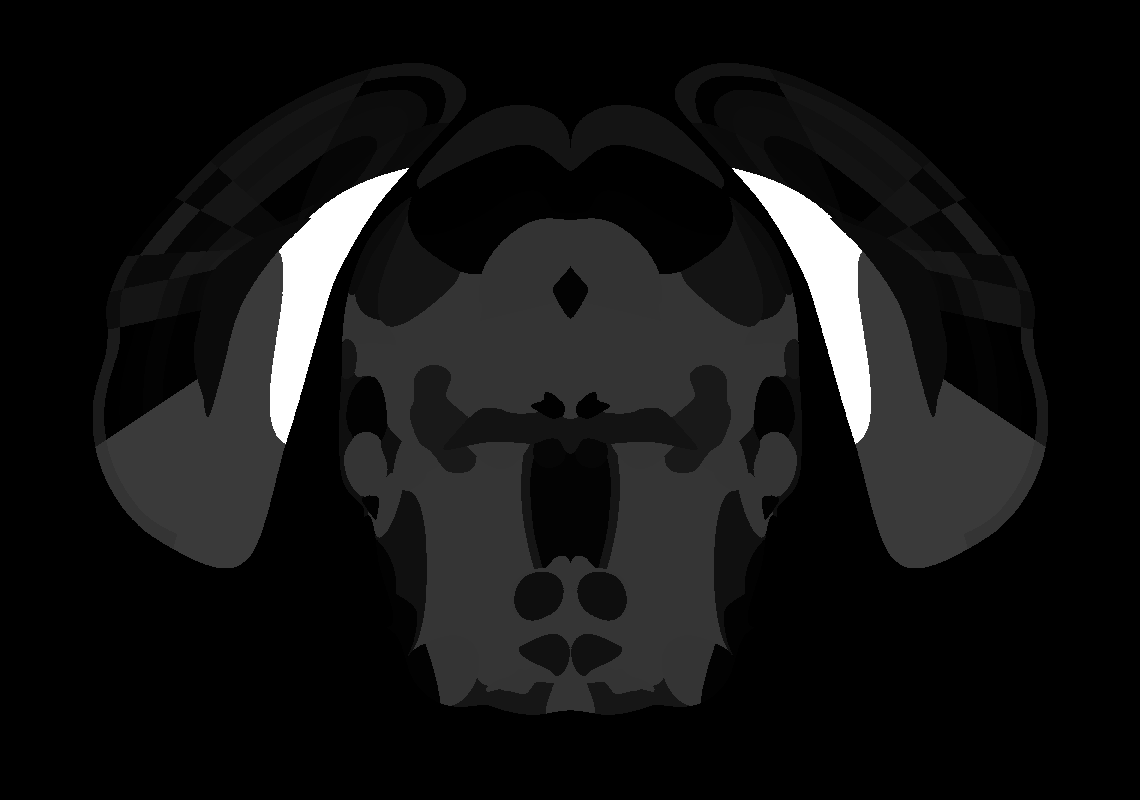

Supplement: Supplementary file 6 — Supplementary Data 4 [file 41467_2019_13057_MOESM6_ESM.zip › Suppl_File1_Labels/89_AP-4.5.tif]

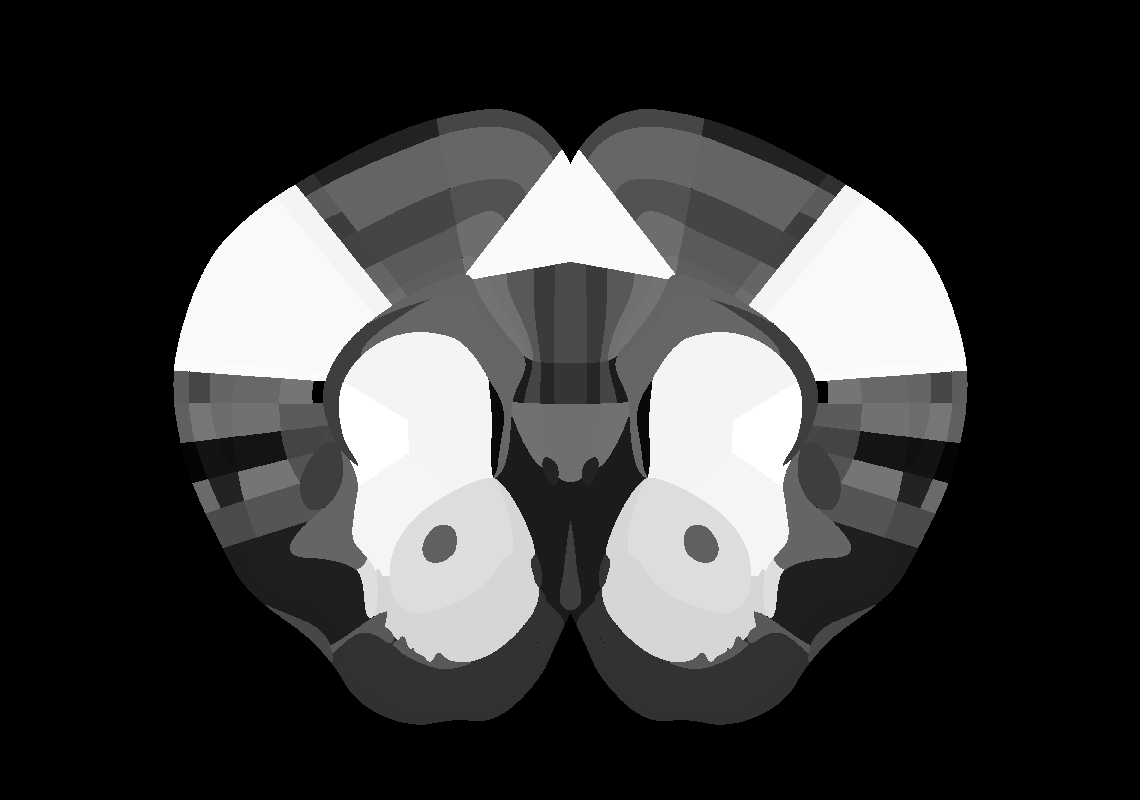

Supplement: Supplementary file 6 — Supplementary Data 4 [file 41467_2019_13057_MOESM6_ESM.zip › Suppl_File1_Labels/32_AP+1.2.tif]

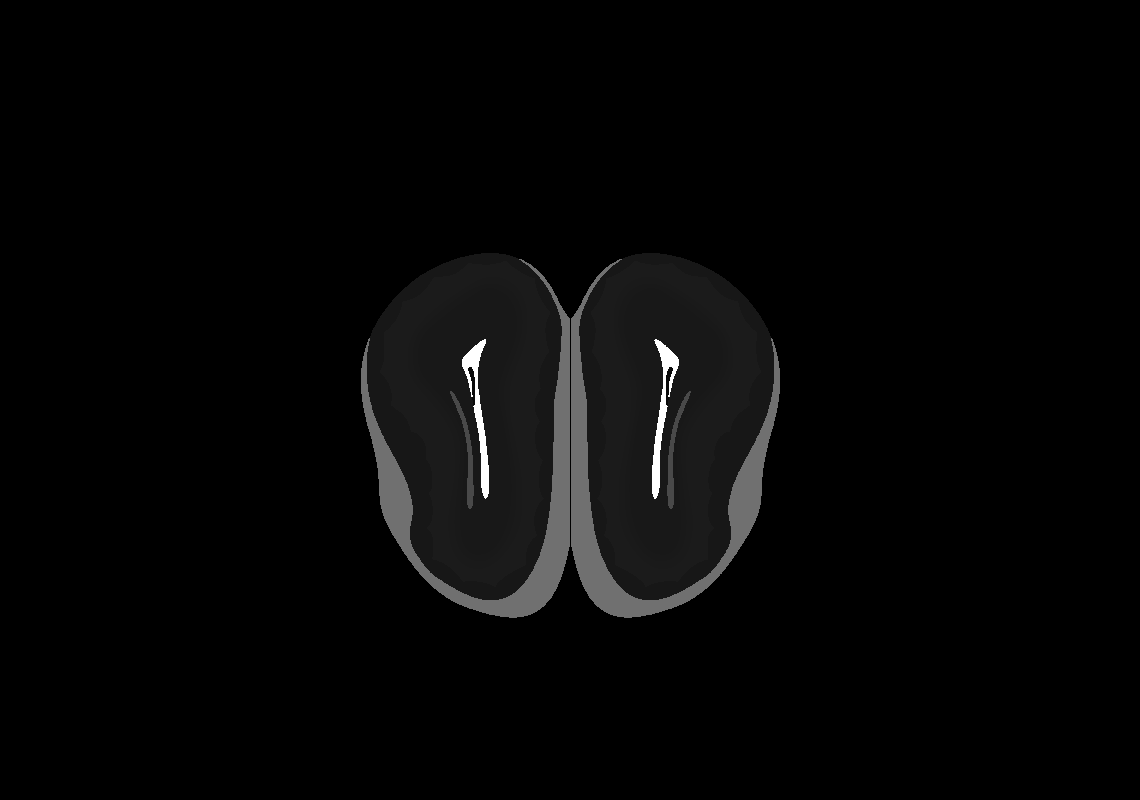

Supplement: Supplementary file 6 — Supplementary Data 4 [file 41467_2019_13057_MOESM6_ESM.zip › Suppl_File1_Labels/3_AP+4.1.tif]

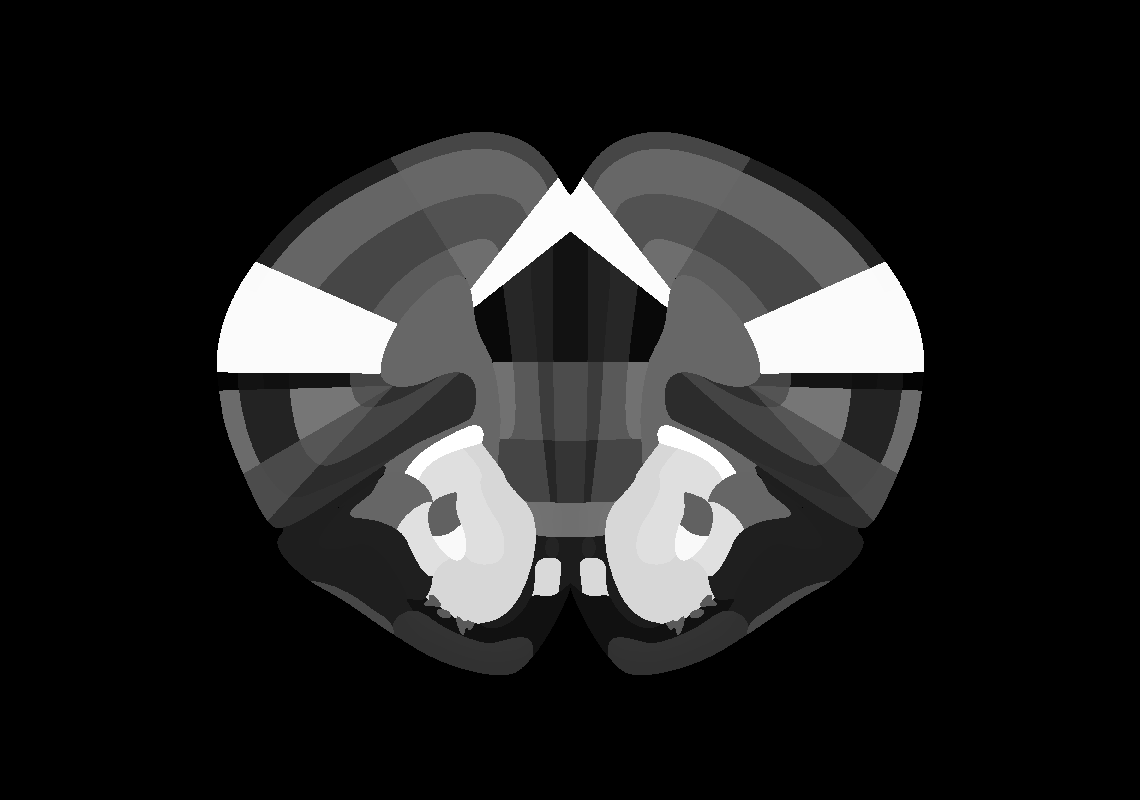

Supplement: Supplementary file 6 — Supplementary Data 4 [file 41467_2019_13057_MOESM6_ESM.zip › Suppl_File1_Labels/26_AP+1.8.tif]

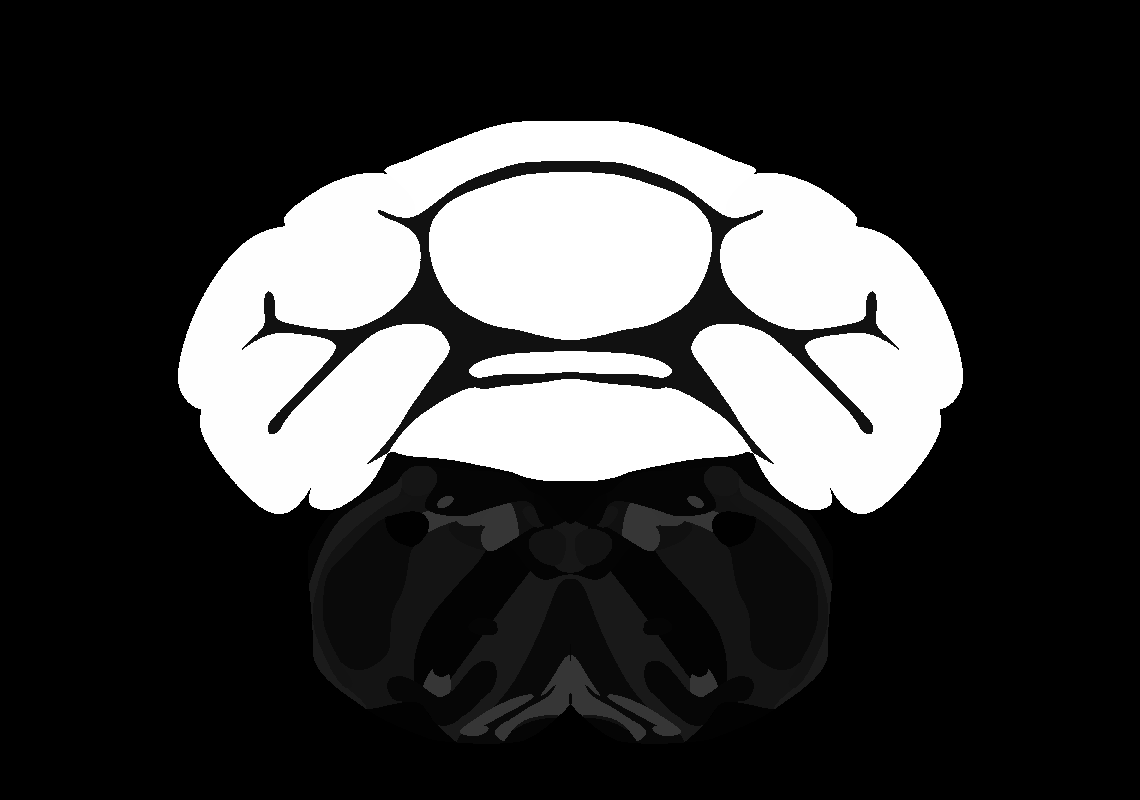

Supplement: Supplementary file 6 — Supplementary Data 4 [file 41467_2019_13057_MOESM6_ESM.zip › Suppl_File1_Labels/114_AP-7.0.tif]

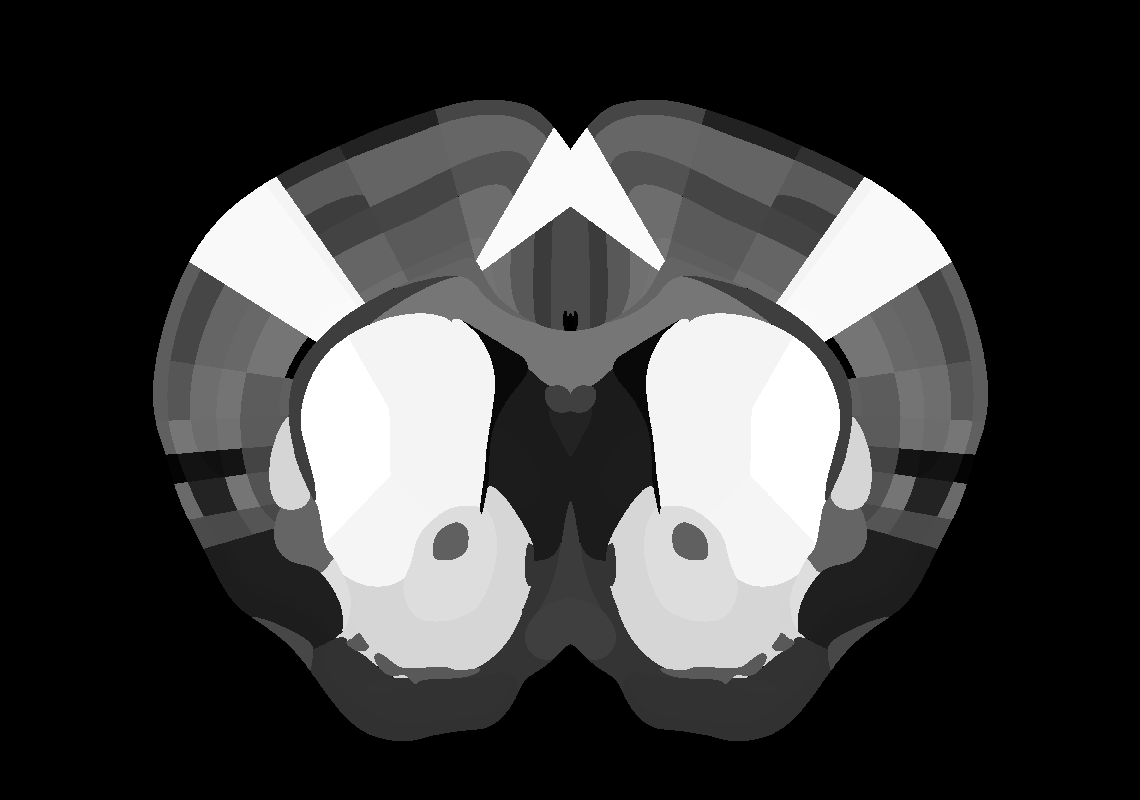

Supplement: Supplementary file 6 — Supplementary Data 4 [file 41467_2019_13057_MOESM6_ESM.zip › Suppl_File1_Labels/35_AP+0.9.tif]

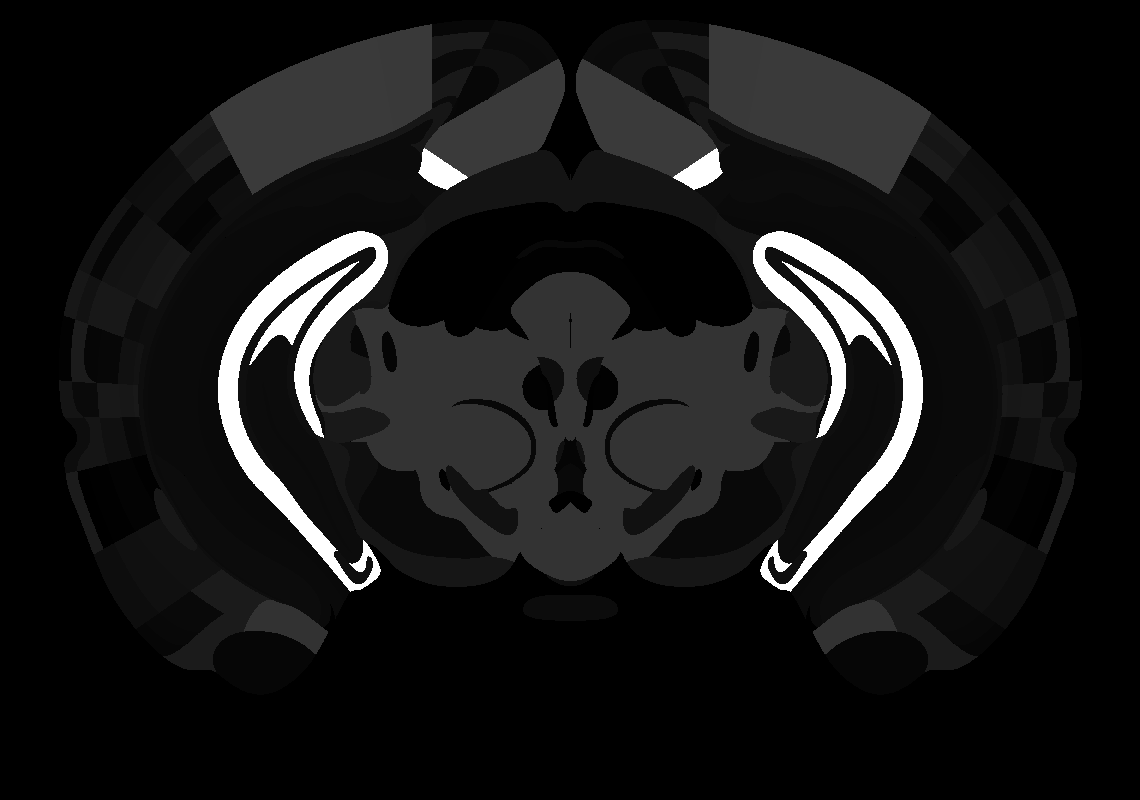

Supplement: Supplementary file 6 — Supplementary Data 4 [file 41467_2019_13057_MOESM6_ESM.zip › Suppl_File1_Labels/77_AP-3.3.tif]

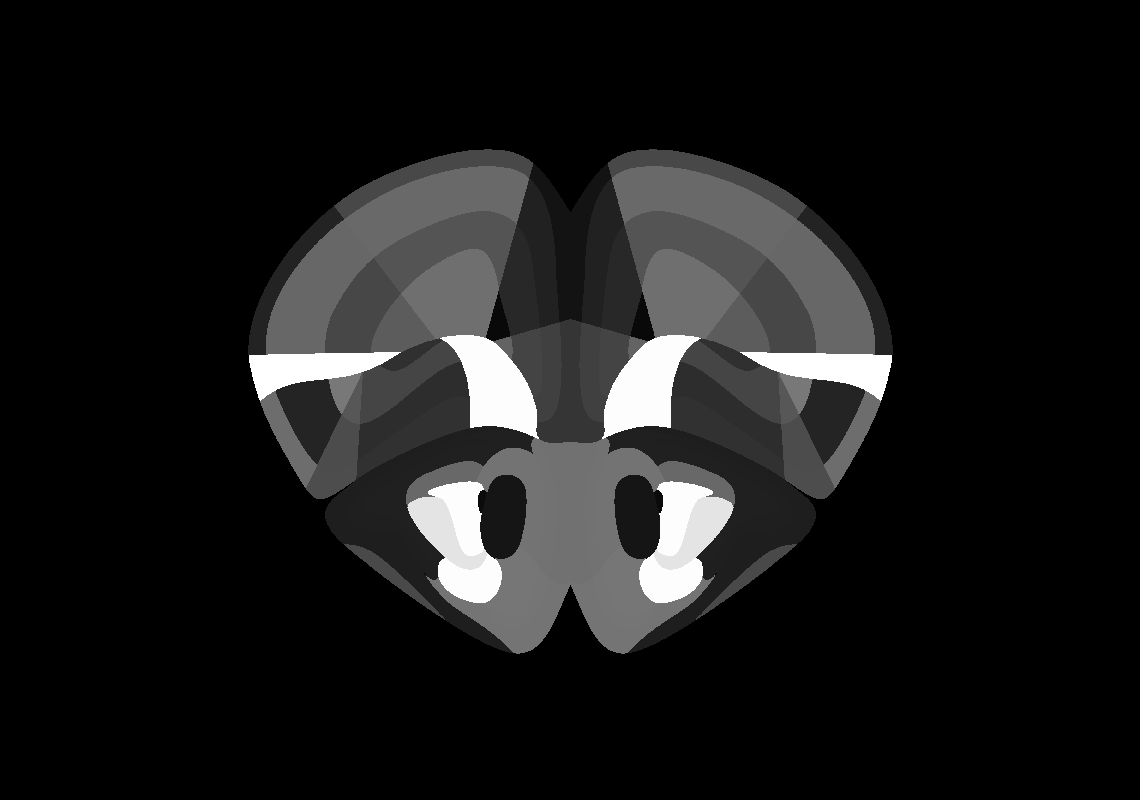

Supplement: Supplementary file 6 — Supplementary Data 4 [file 41467_2019_13057_MOESM6_ESM.zip › Suppl_File1_Labels/22_AP+2.2.tif]

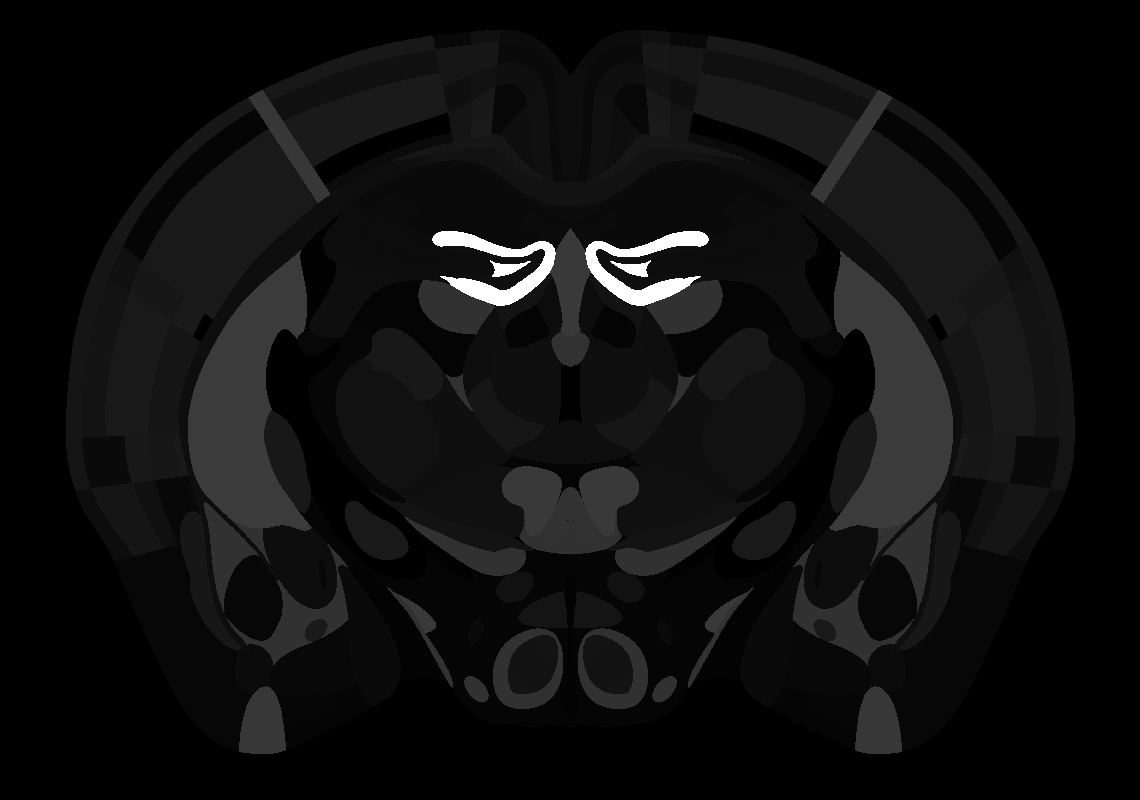

Supplement: Supplementary file 6 — Supplementary Data 4 [file 41467_2019_13057_MOESM6_ESM.zip › Suppl_File1_Labels/59_AP-1.5.tif]

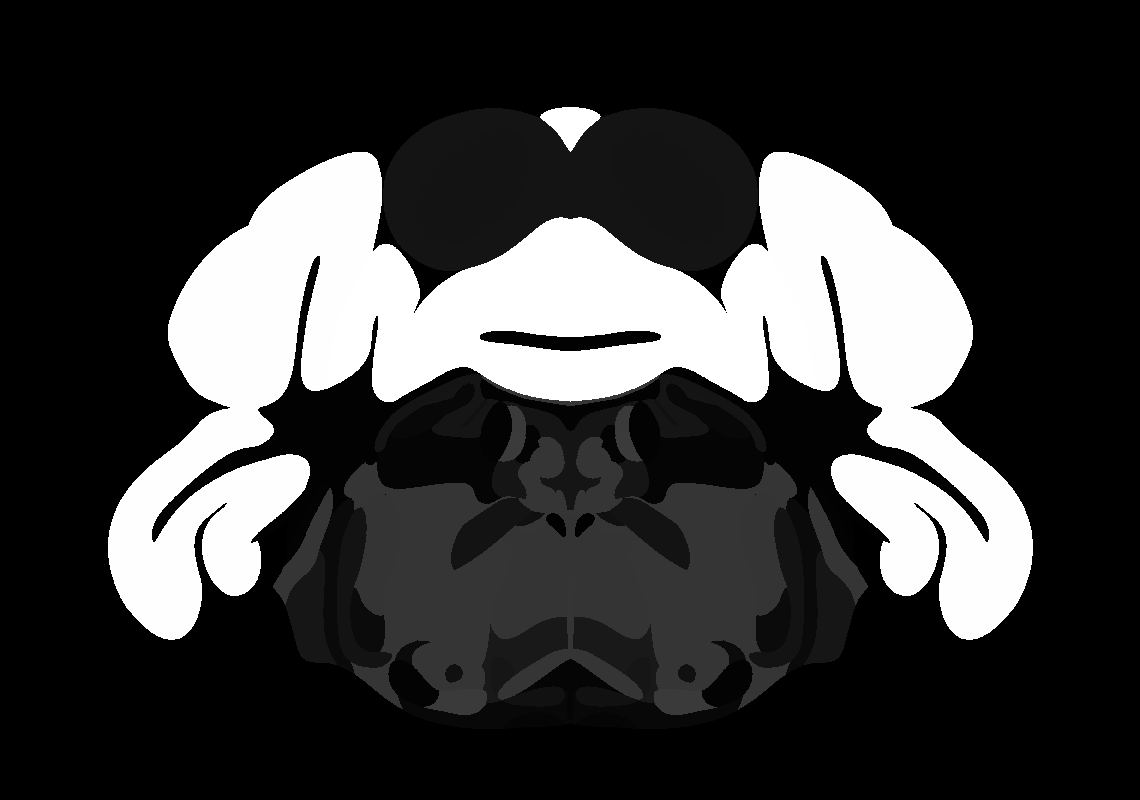

Supplement: Supplementary file 6 — Supplementary Data 4 [file 41467_2019_13057_MOESM6_ESM.zip › Suppl_File1_Labels/99_AP-5.5.tif]

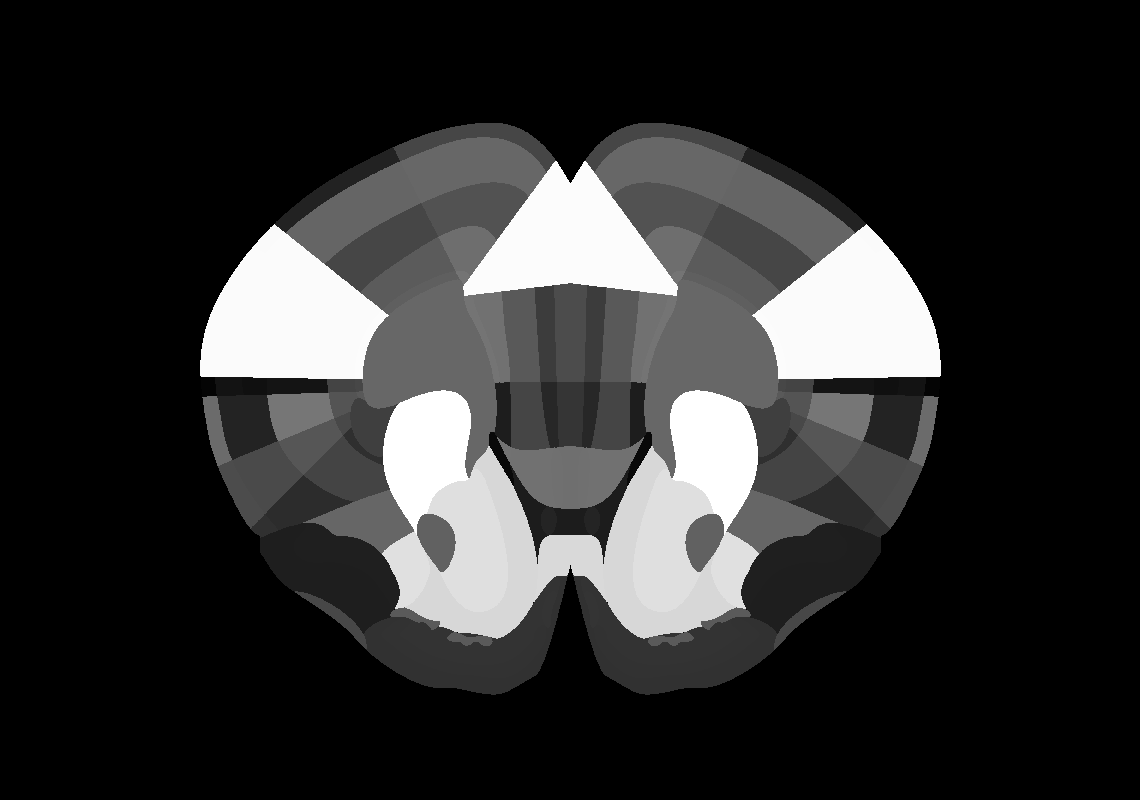

Supplement: Supplementary file 6 — Supplementary Data 4 [file 41467_2019_13057_MOESM6_ESM.zip › Suppl_File1_Labels/28_AP+1.6.tif]

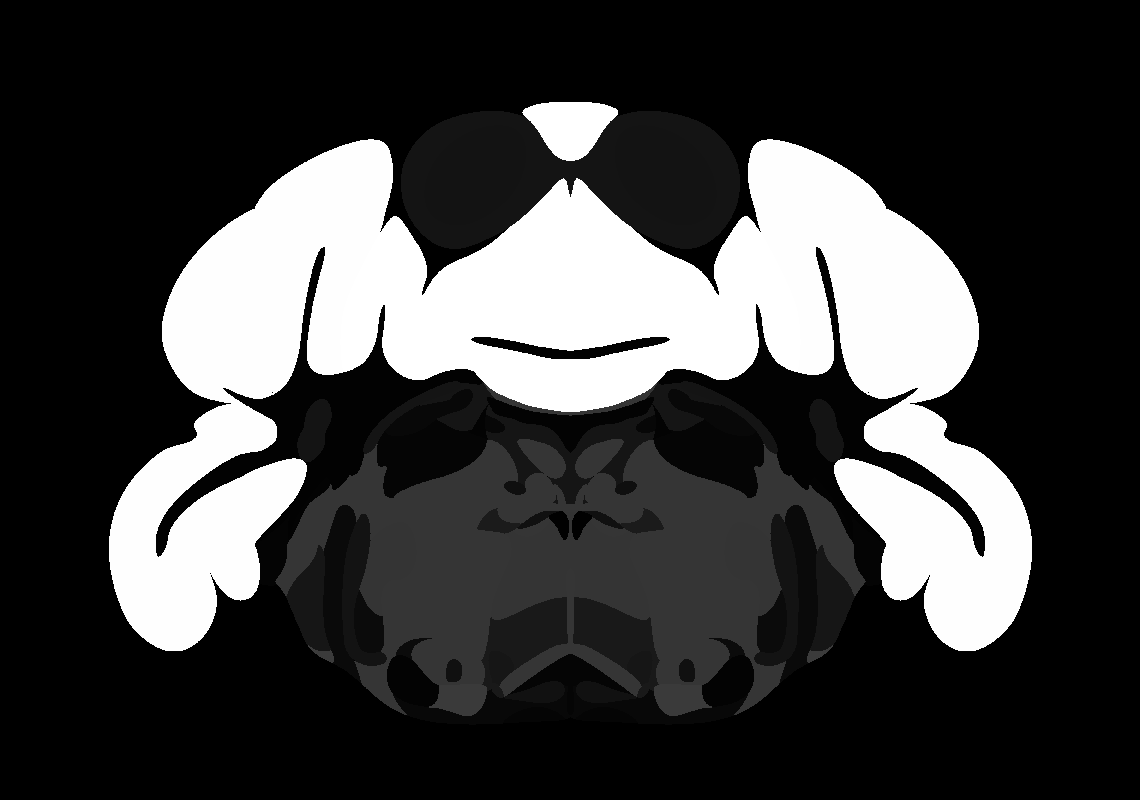

Supplement: Supplementary file 6 — Supplementary Data 4 [file 41467_2019_13057_MOESM6_ESM.zip › Suppl_File1_Labels/100_AP-5.6.tif]

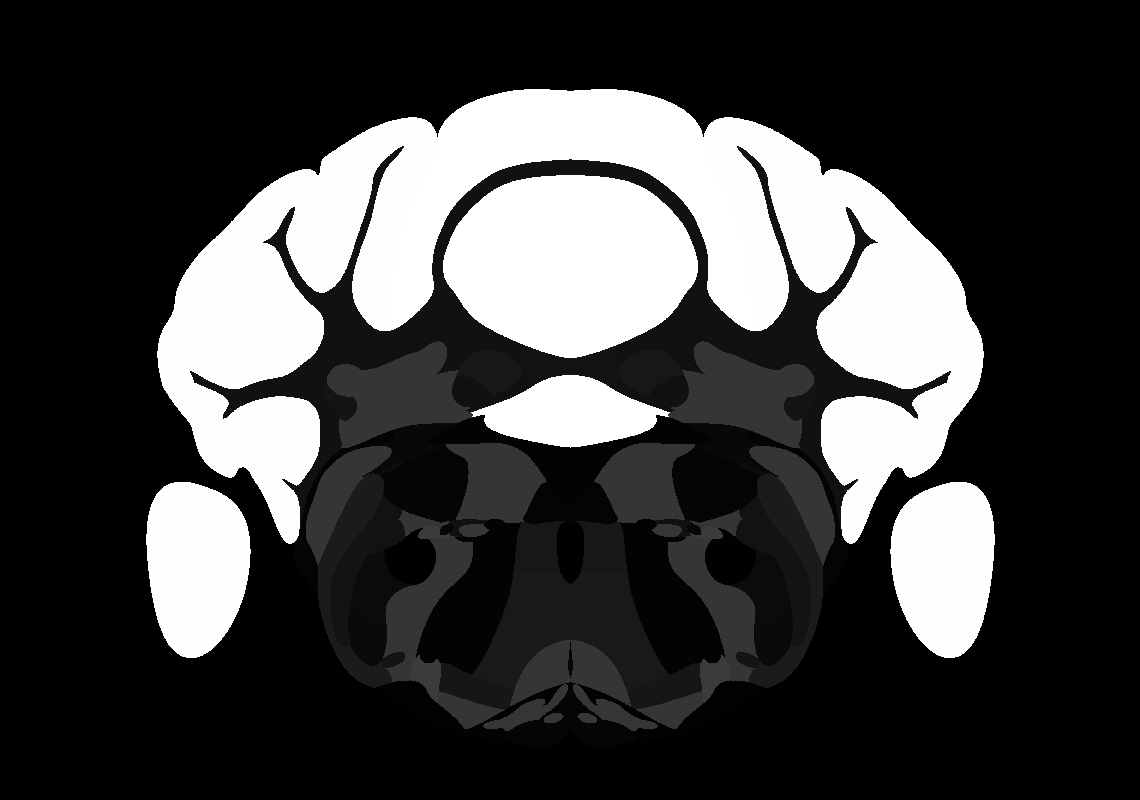

Supplement: Supplementary file 6 — Supplementary Data 4 [file 41467_2019_13057_MOESM6_ESM.zip › Suppl_File1_Labels/107_AP-6.3.tif]

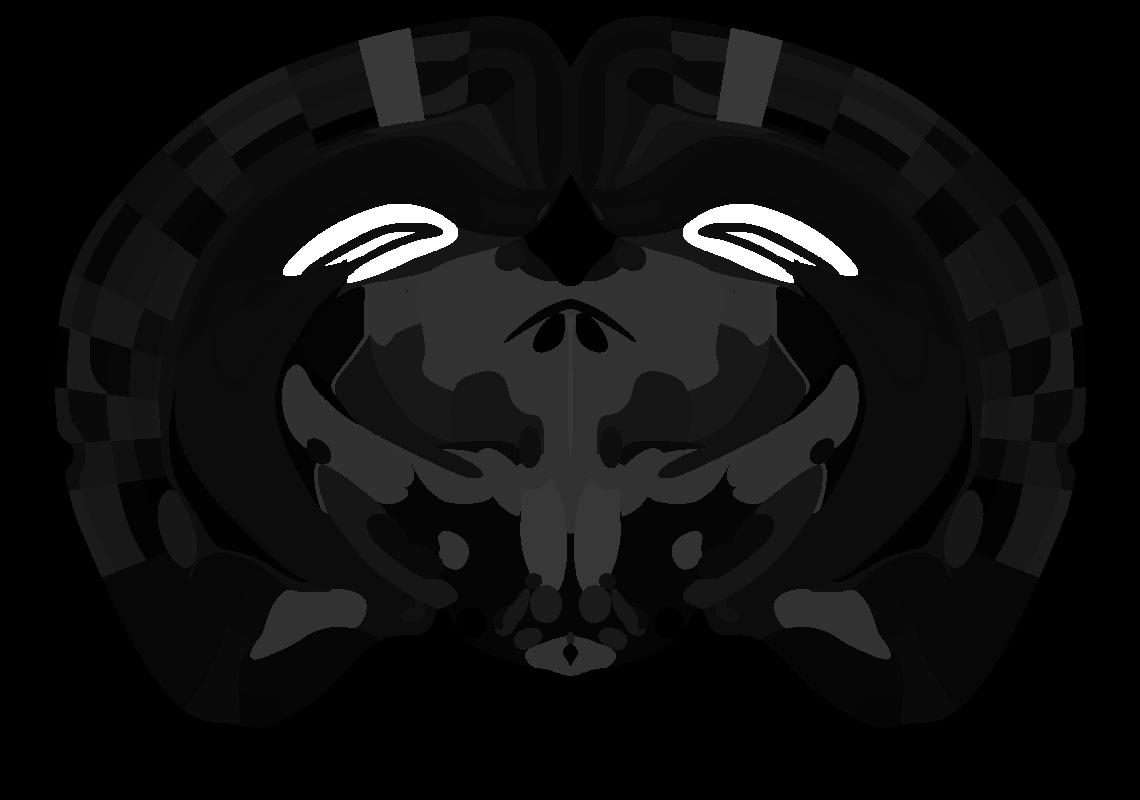

Supplement: Supplementary file 6 — Supplementary Data 4 [file 41467_2019_13057_MOESM6_ESM.zip › Suppl_File1_Labels/70_AP-2.6.tif]

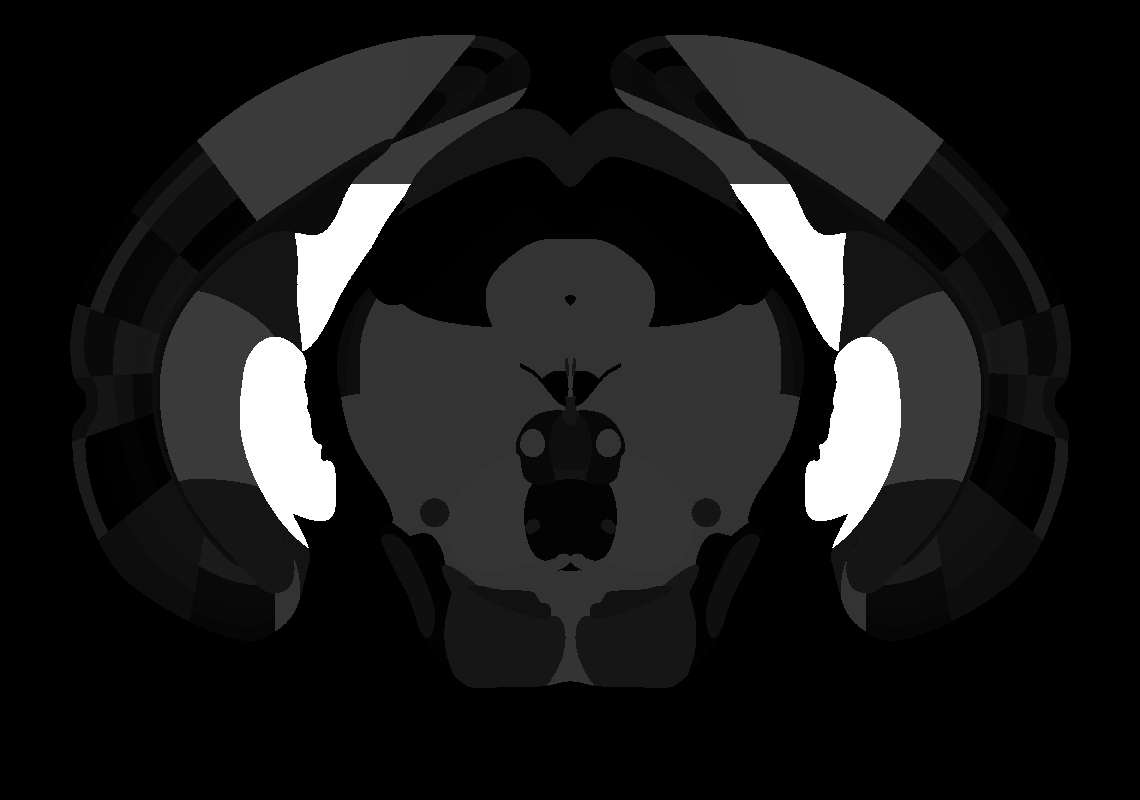

Supplement: Supplementary file 6 — Supplementary Data 4 [file 41467_2019_13057_MOESM6_ESM.zip › Suppl_File1_Labels/83_AP-3.9.tif]

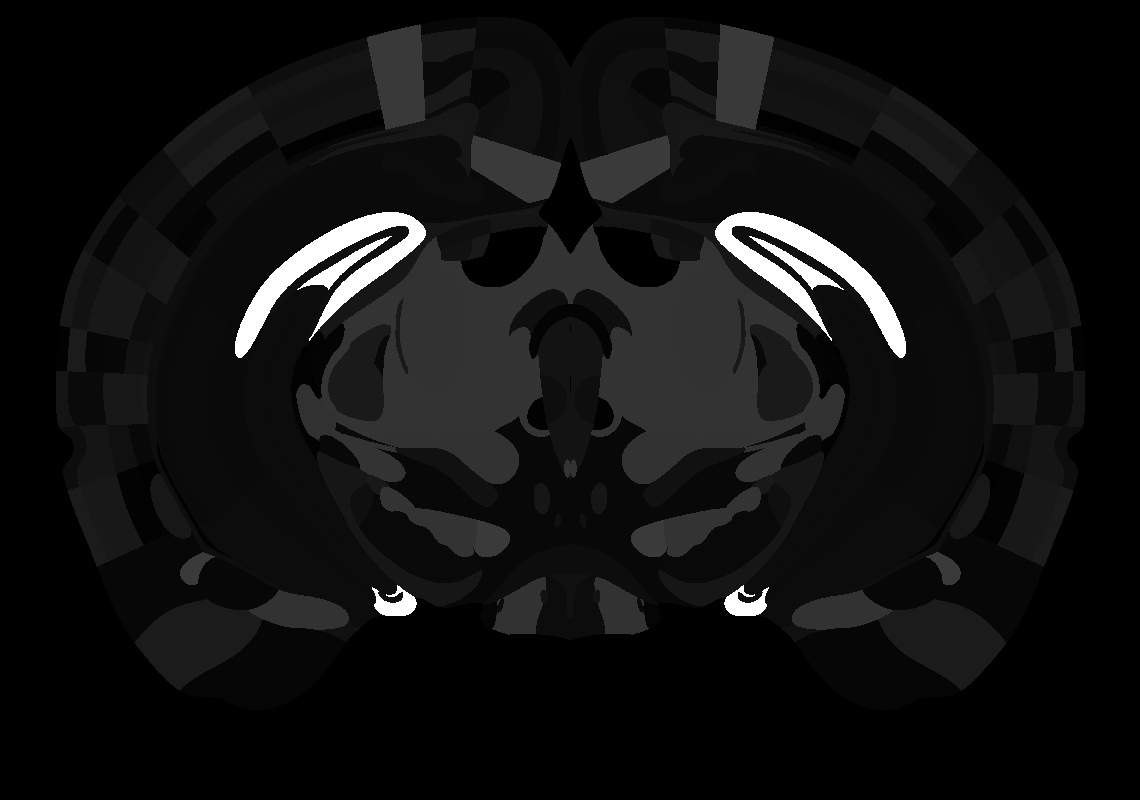

Supplement: Supplementary file 6 — Supplementary Data 4 [file 41467_2019_13057_MOESM6_ESM.zip › Suppl_File1_Labels/73_AP-2.9.tif]

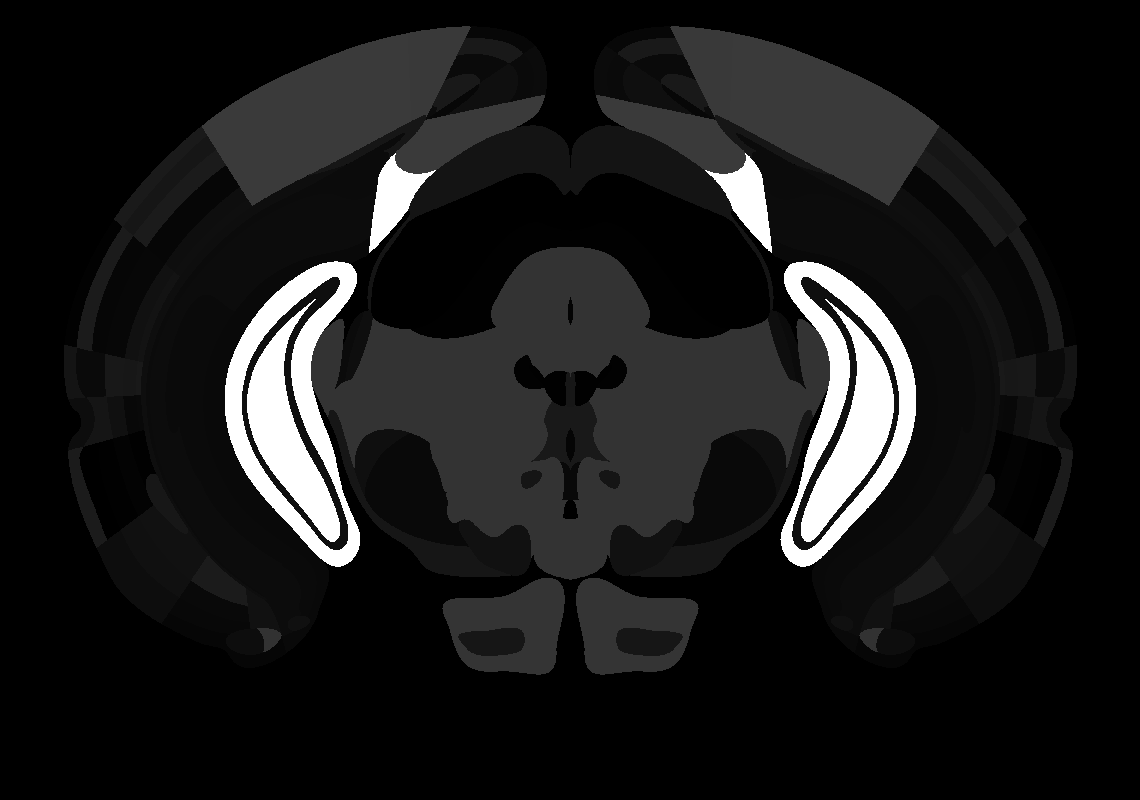

Supplement: Supplementary file 6 — Supplementary Data 4 [file 41467_2019_13057_MOESM6_ESM.zip › Suppl_File1_Labels/80_AP-3.6.tif]

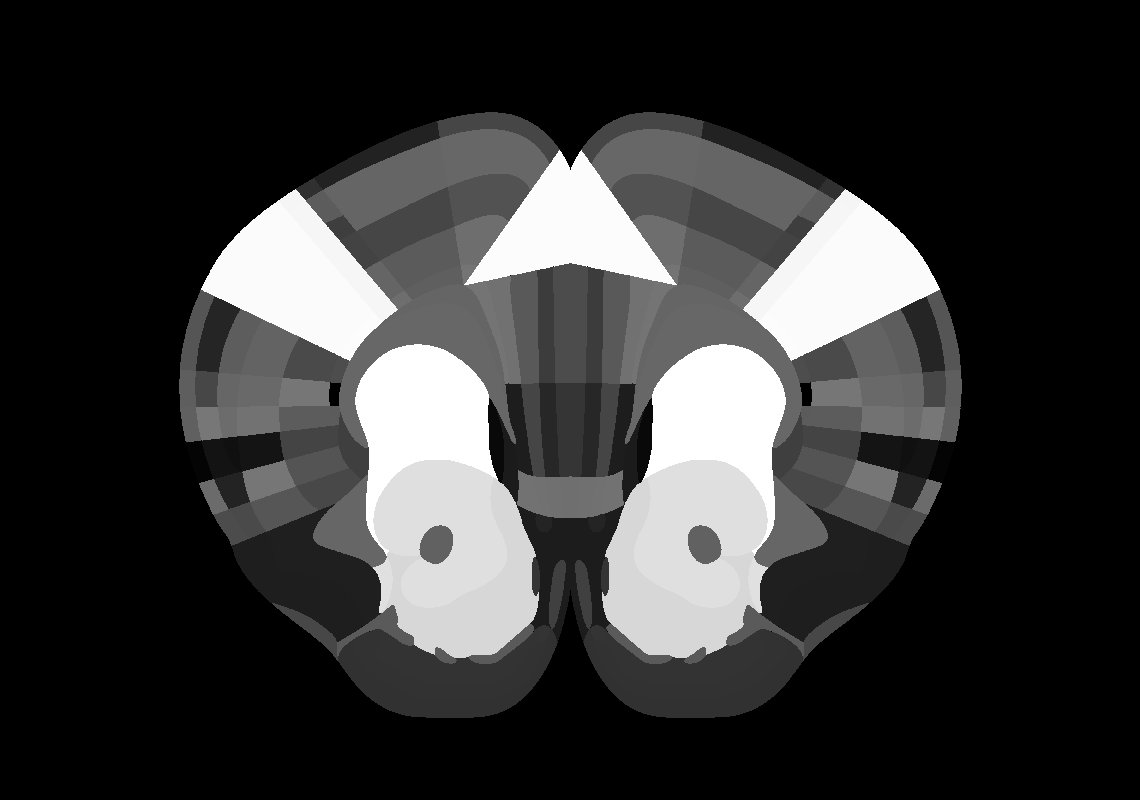

Supplement: Supplementary file 6 — Supplementary Data 4 [file 41467_2019_13057_MOESM6_ESM.zip › Suppl_File1_Labels/31_AP+1.3.tif]

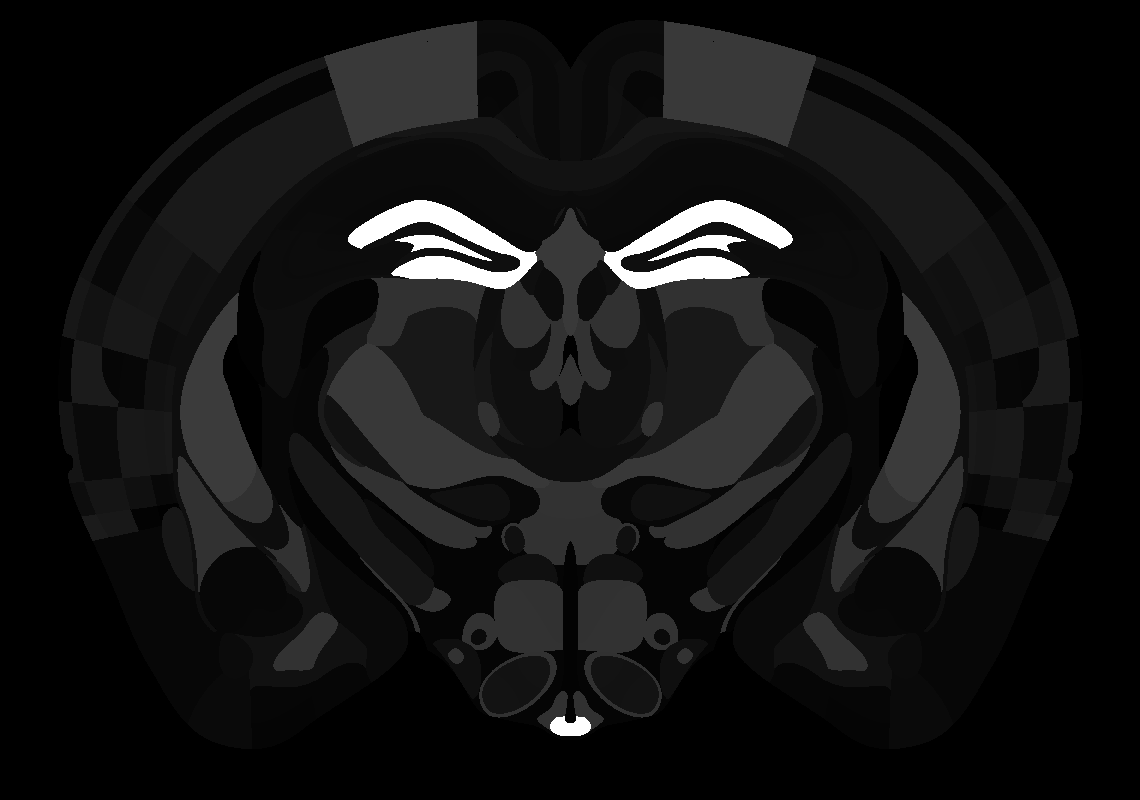

Supplement: Supplementary file 6 — Supplementary Data 4 [file 41467_2019_13057_MOESM6_ESM.zip › Suppl_File1_Labels/64_AP-2.0.tif]

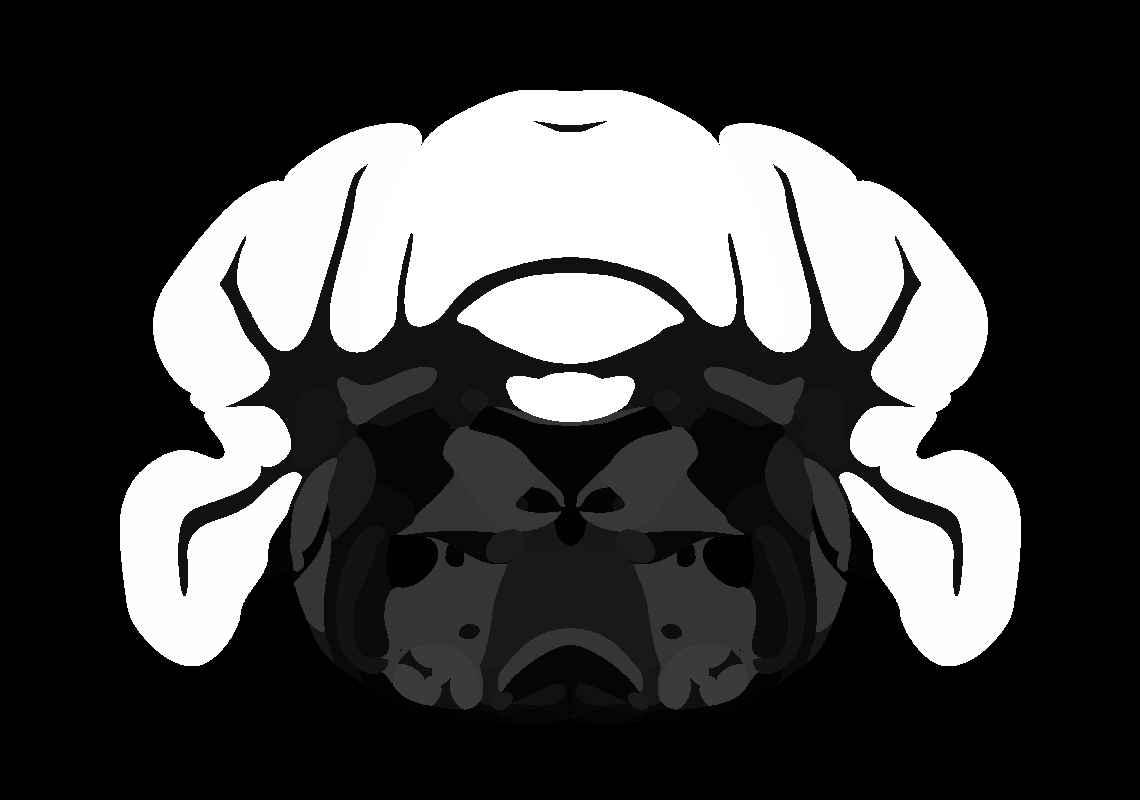

Supplement: Supplementary file 6 — Supplementary Data 4 [file 41467_2019_13057_MOESM6_ESM.zip › Suppl_File1_Labels/103_AP-5.9.tif]

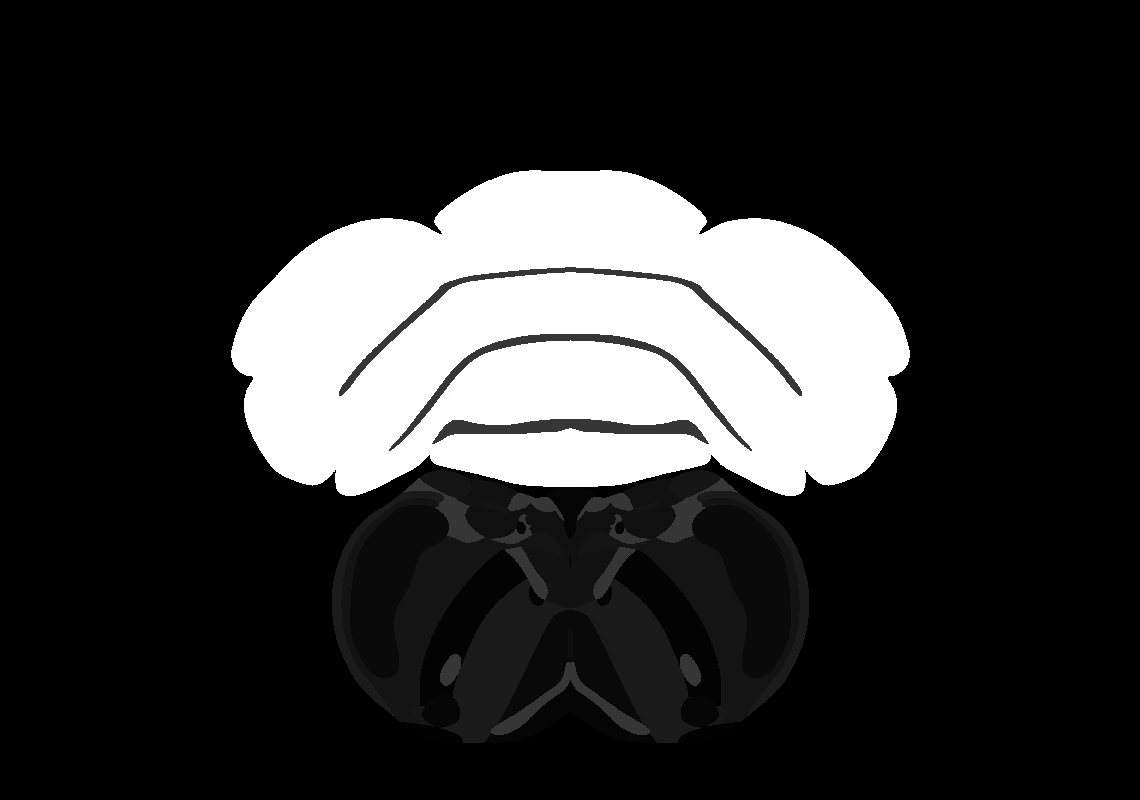

Supplement: Supplementary file 6 — Supplementary Data 4 [file 41467_2019_13057_MOESM6_ESM.zip › Suppl_File1_Labels/119_AP-7.5.tif]

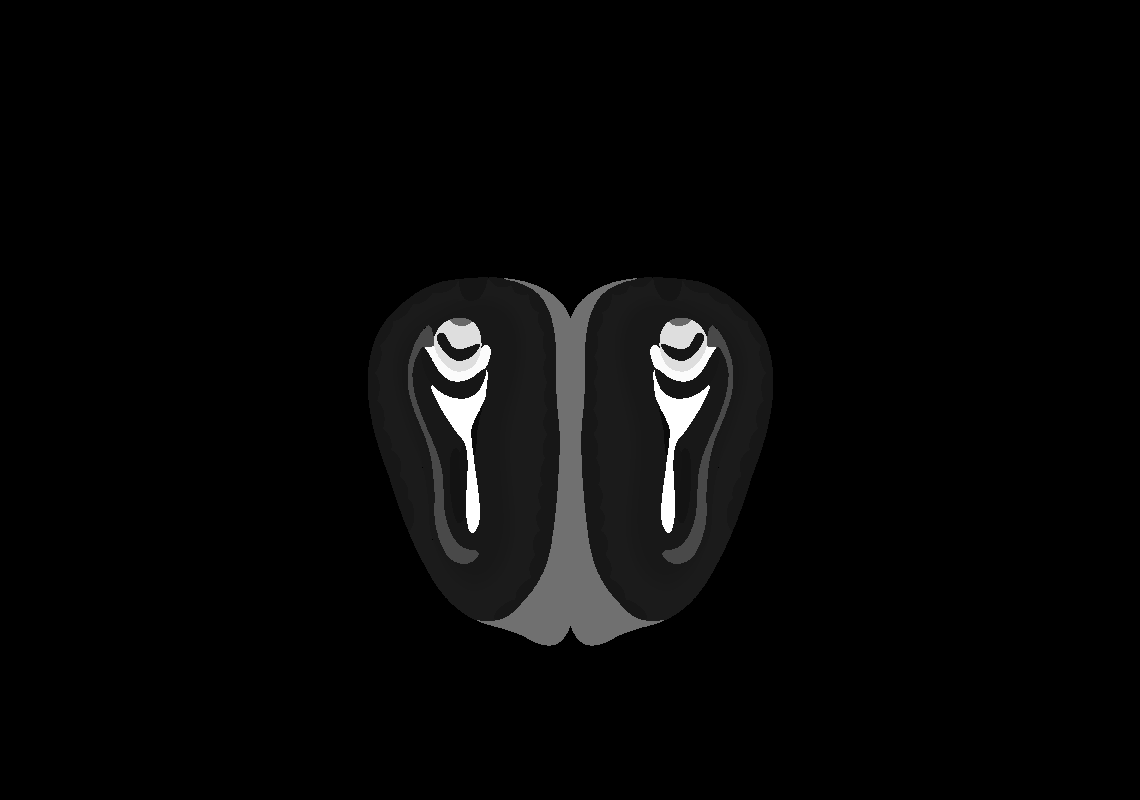

Supplement: Supplementary file 6 — Supplementary Data 4 [file 41467_2019_13057_MOESM6_ESM.zip › Suppl_File1_Labels/9_AP+3.5.tif]

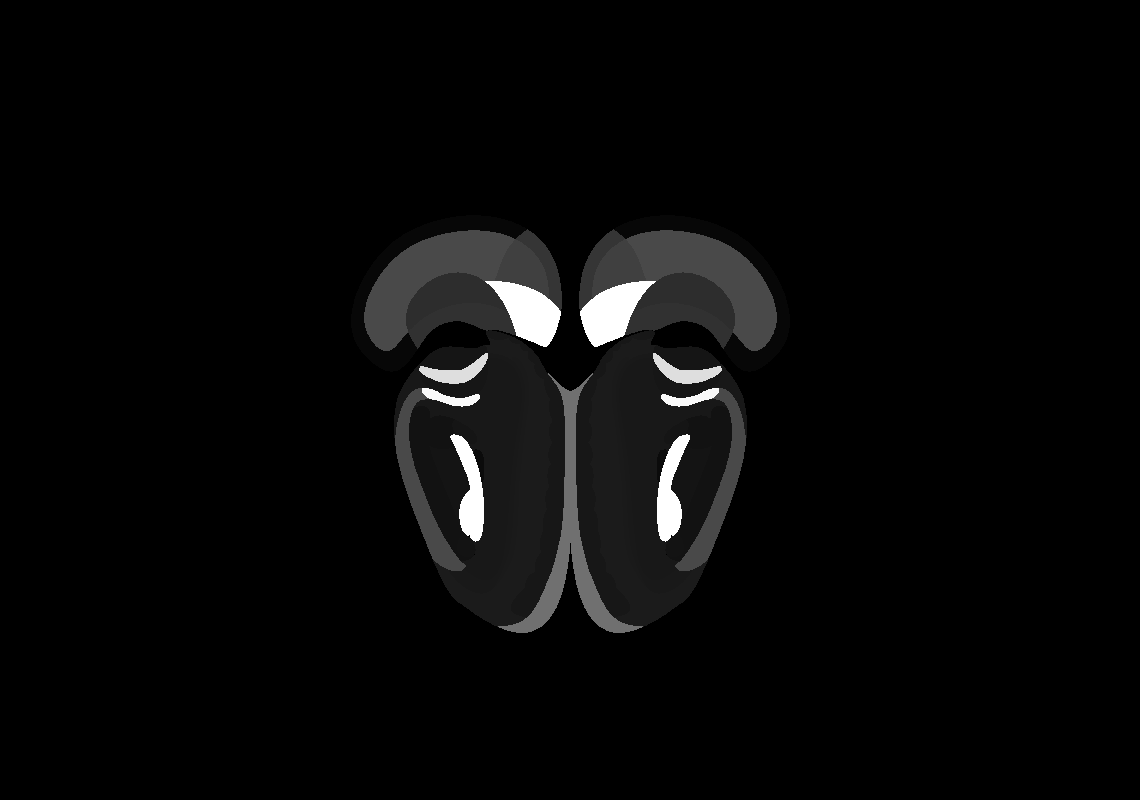

Supplement: Supplementary file 6 — Supplementary Data 4 [file 41467_2019_13057_MOESM6_ESM.zip › Suppl_File1_Labels/12_AP+3.2.tif]

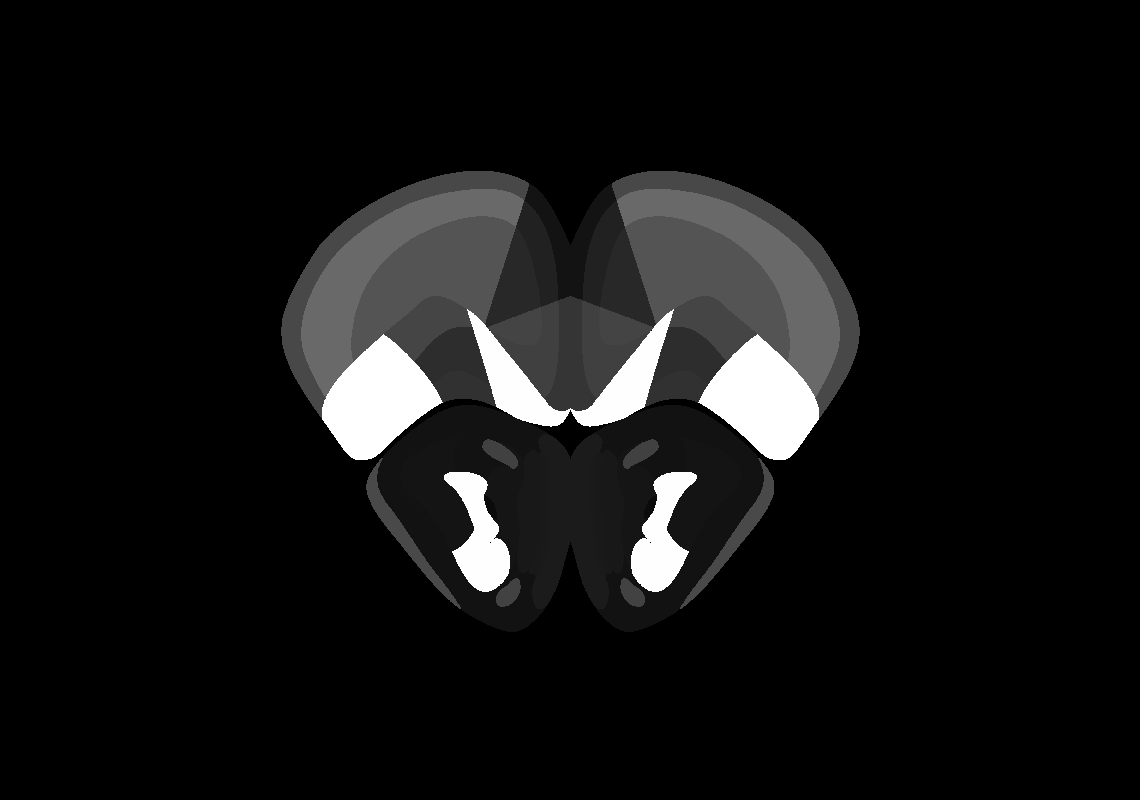

Supplement: Supplementary file 6 — Supplementary Data 4 [file 41467_2019_13057_MOESM6_ESM.zip › Suppl_File1_Labels/18_AP+2.6.tif]

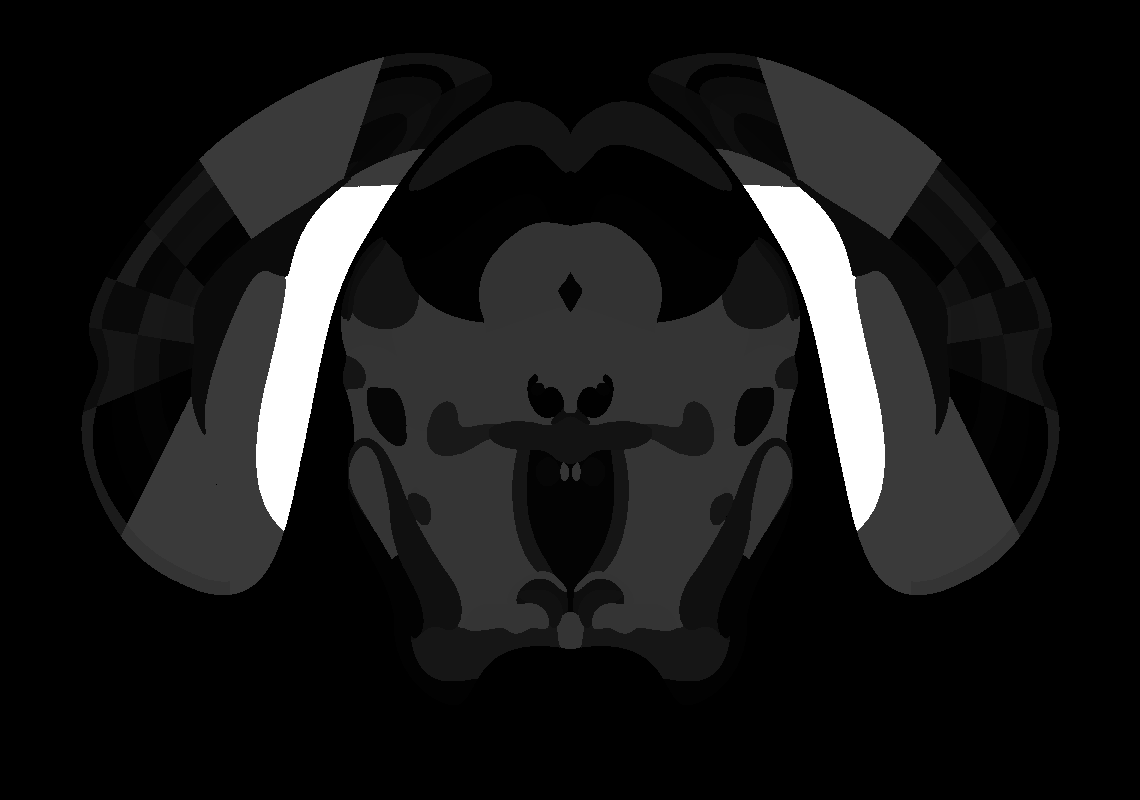

Supplement: Supplementary file 6 — Supplementary Data 4 [file 41467_2019_13057_MOESM6_ESM.zip › Suppl_File1_Labels/87_AP-4.3.tif]

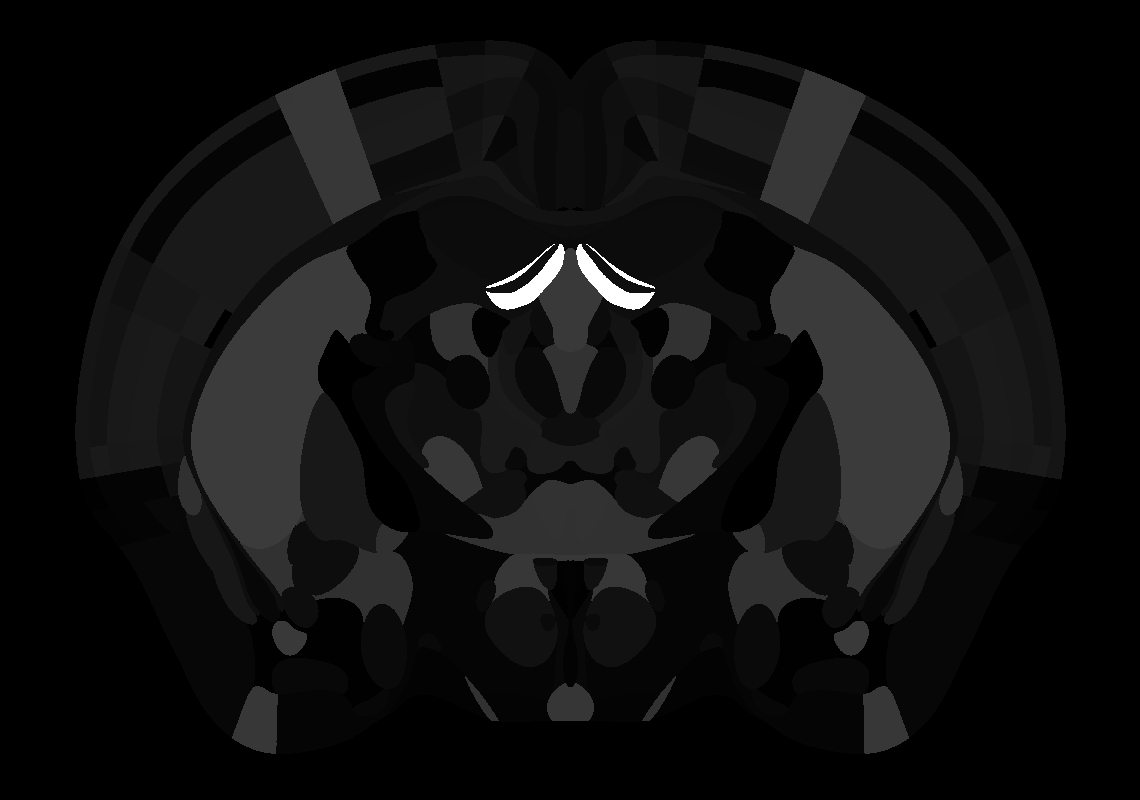

Supplement: Supplementary file 6 — Supplementary Data 4 [file 41467_2019_13057_MOESM6_ESM.zip › Suppl_File1_Labels/54_AP-1.0.tif]

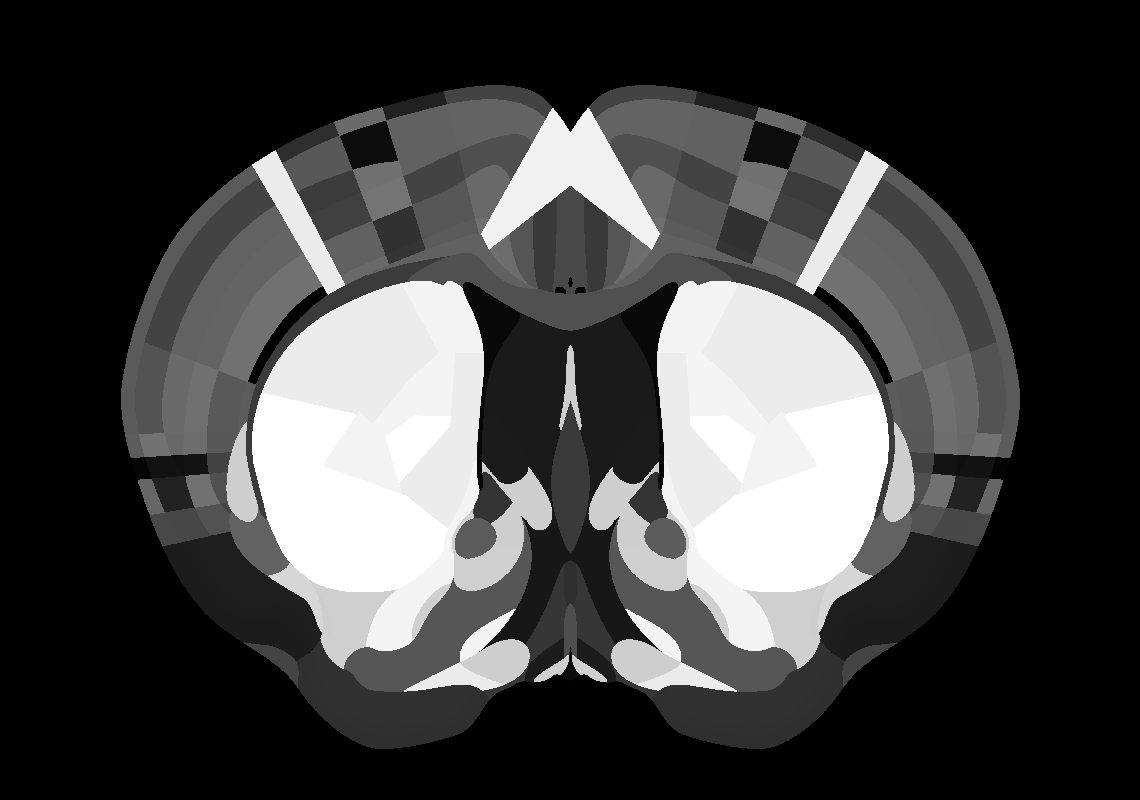

Supplement: Supplementary file 6 — Supplementary Data 4 [file 41467_2019_13057_MOESM6_ESM.zip › Suppl_File1_Labels/40_AP+0.4.tif]

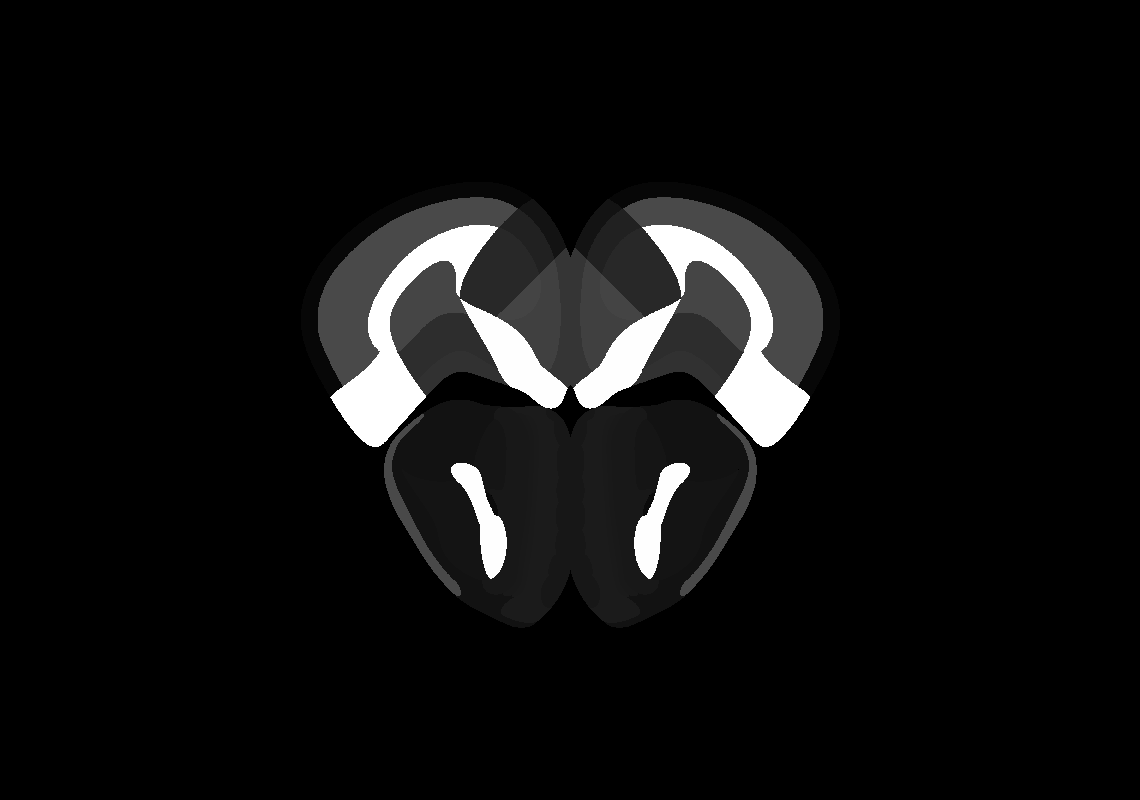

Supplement: Supplementary file 6 — Supplementary Data 4 [file 41467_2019_13057_MOESM6_ESM.zip › Suppl_File1_Labels/16_AP+2.8.tif]

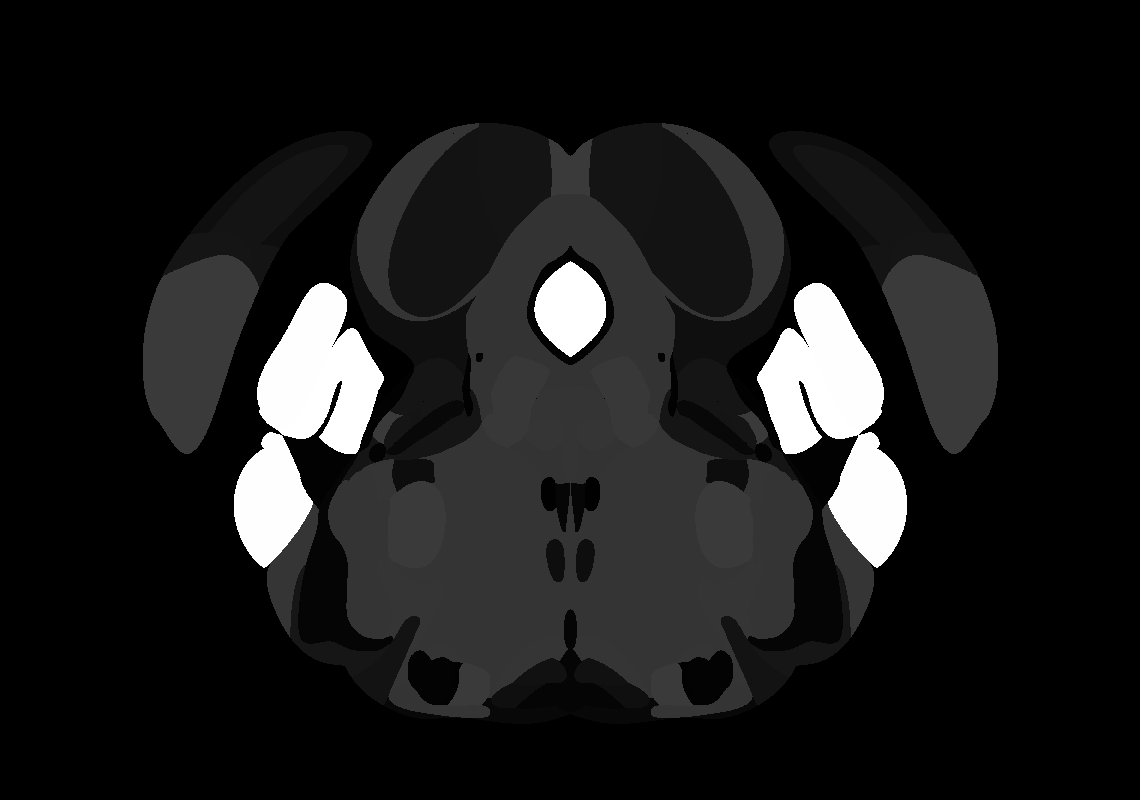

Supplement: Supplementary file 6 — Supplementary Data 4 [file 41467_2019_13057_MOESM6_ESM.zip › Suppl_File1_Labels/94_AP-5.0.tif]

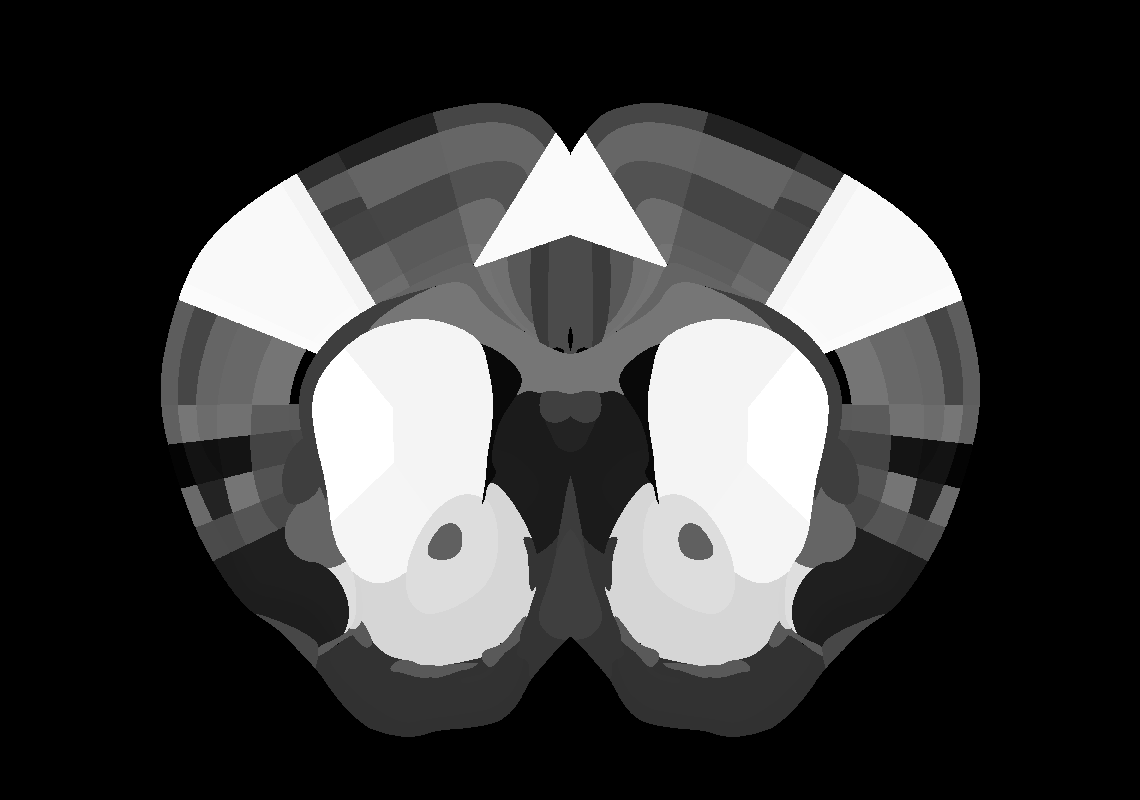

Supplement: Supplementary file 6 — Supplementary Data 4 [file 41467_2019_13057_MOESM6_ESM.zip › Suppl_File1_Labels/34_AP+1.0.tif]

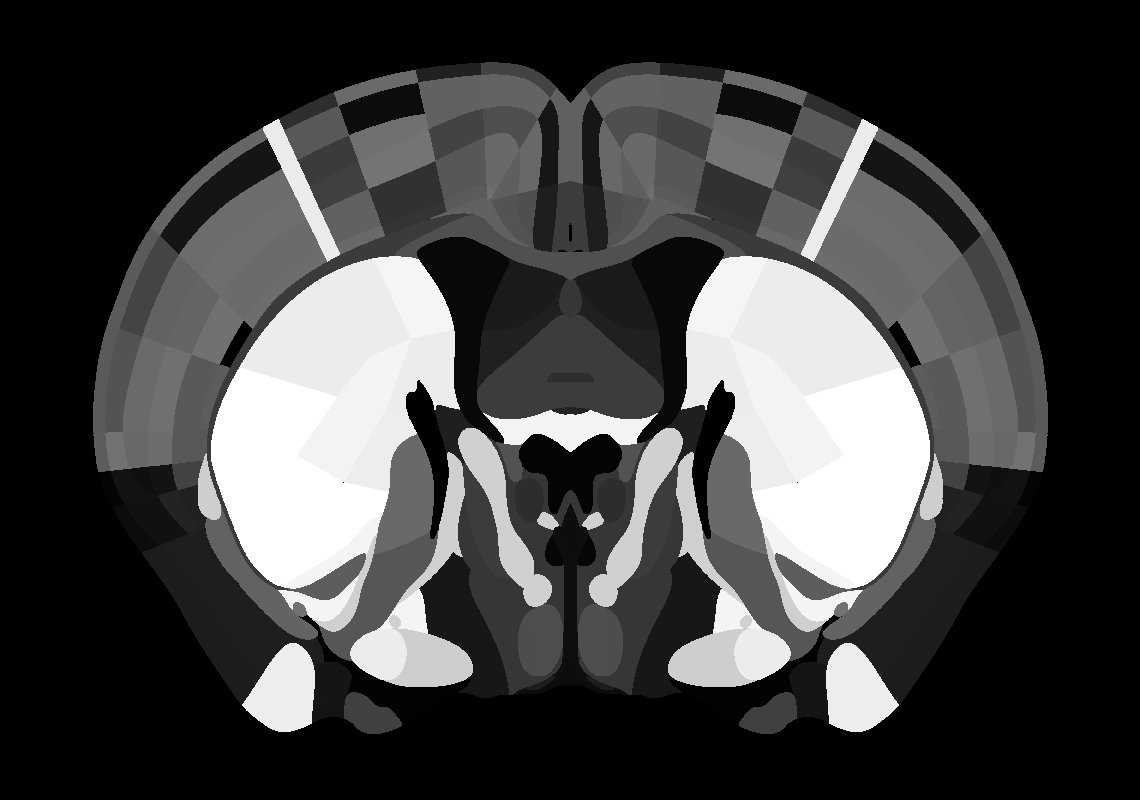

Supplement: Supplementary file 6 — Supplementary Data 4 [file 41467_2019_13057_MOESM6_ESM.zip › Suppl_File1_Labels/47_AP-0.3.tif]

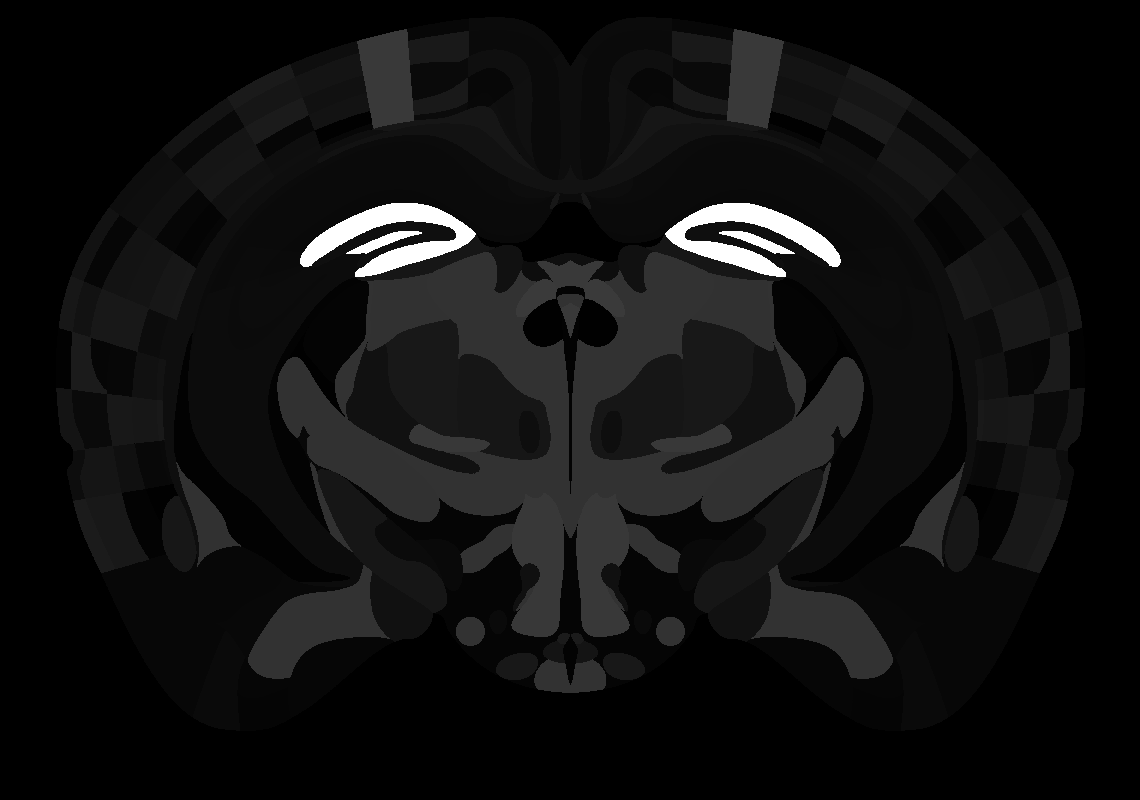

Supplement: Supplementary file 6 — Supplementary Data 4 [file 41467_2019_13057_MOESM6_ESM.zip › Suppl_File1_Labels/69_AP-2.5.tif]

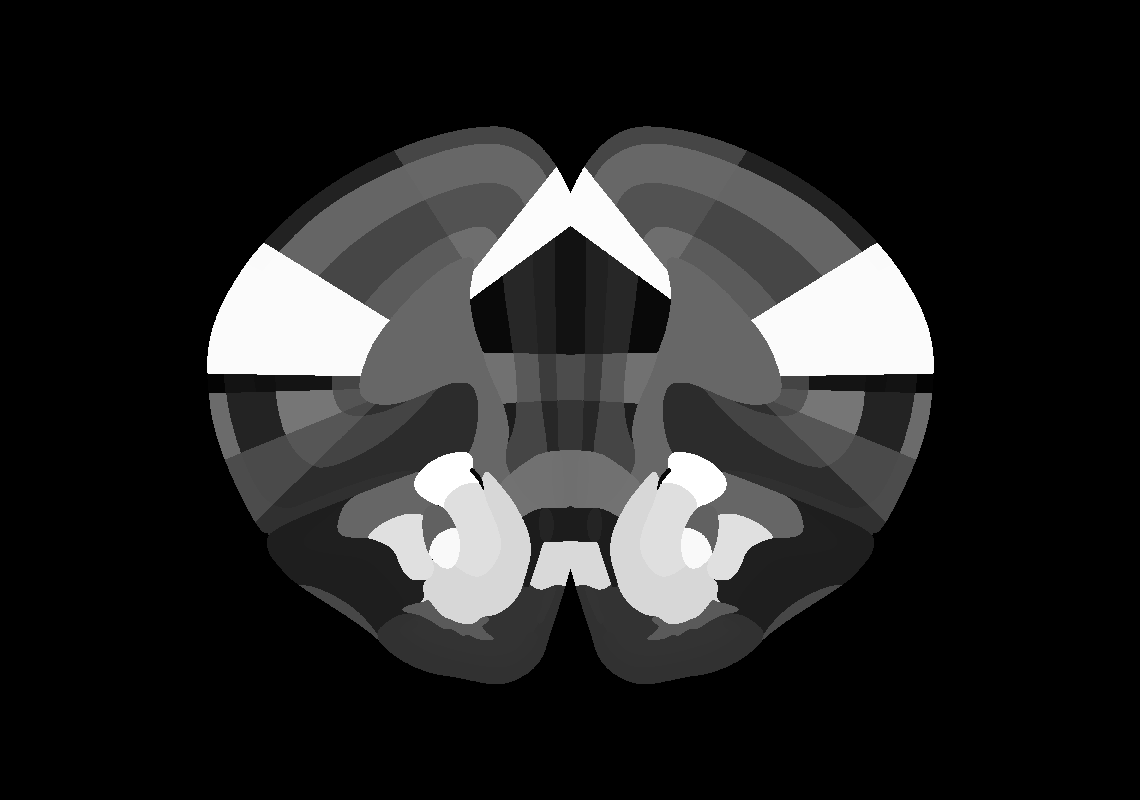

Supplement: Supplementary file 6 — Supplementary Data 4 [file 41467_2019_13057_MOESM6_ESM.zip › Suppl_File1_Labels/27_AP+1.7.tif]

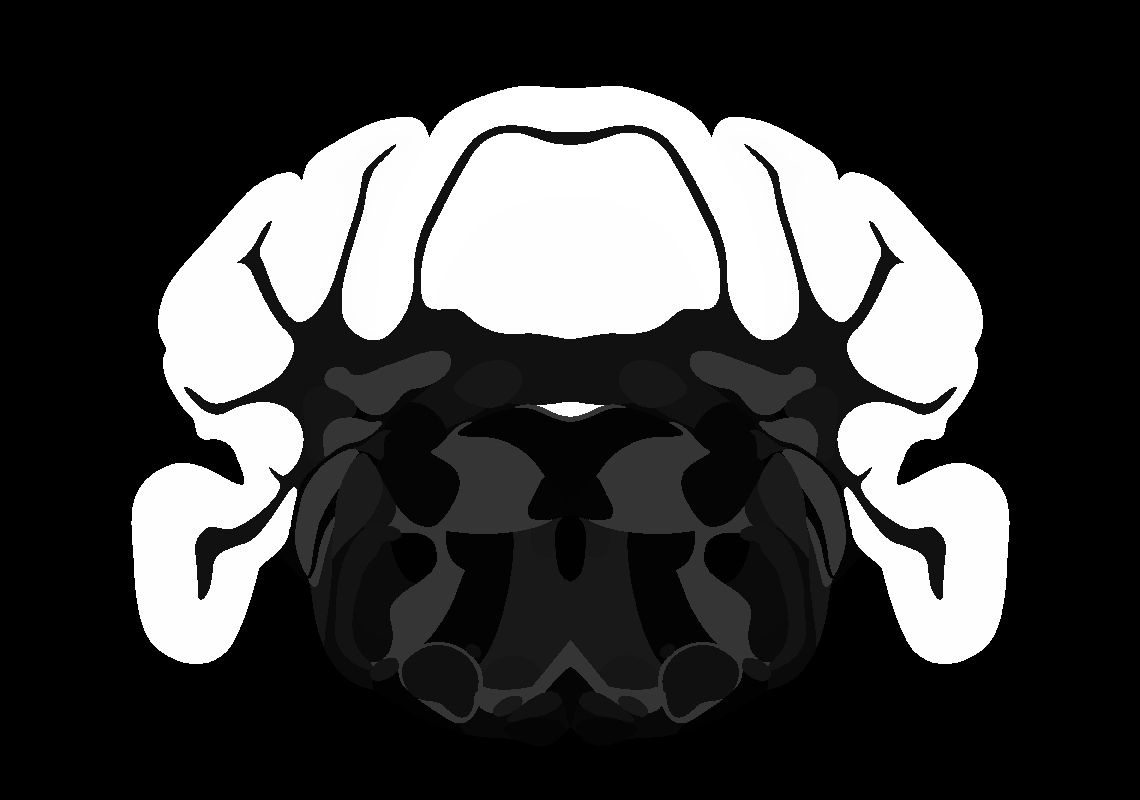

Supplement: Supplementary file 6 — Supplementary Data 4 [file 41467_2019_13057_MOESM6_ESM.zip › Suppl_File1_Labels/105_AP-6.1.tif]

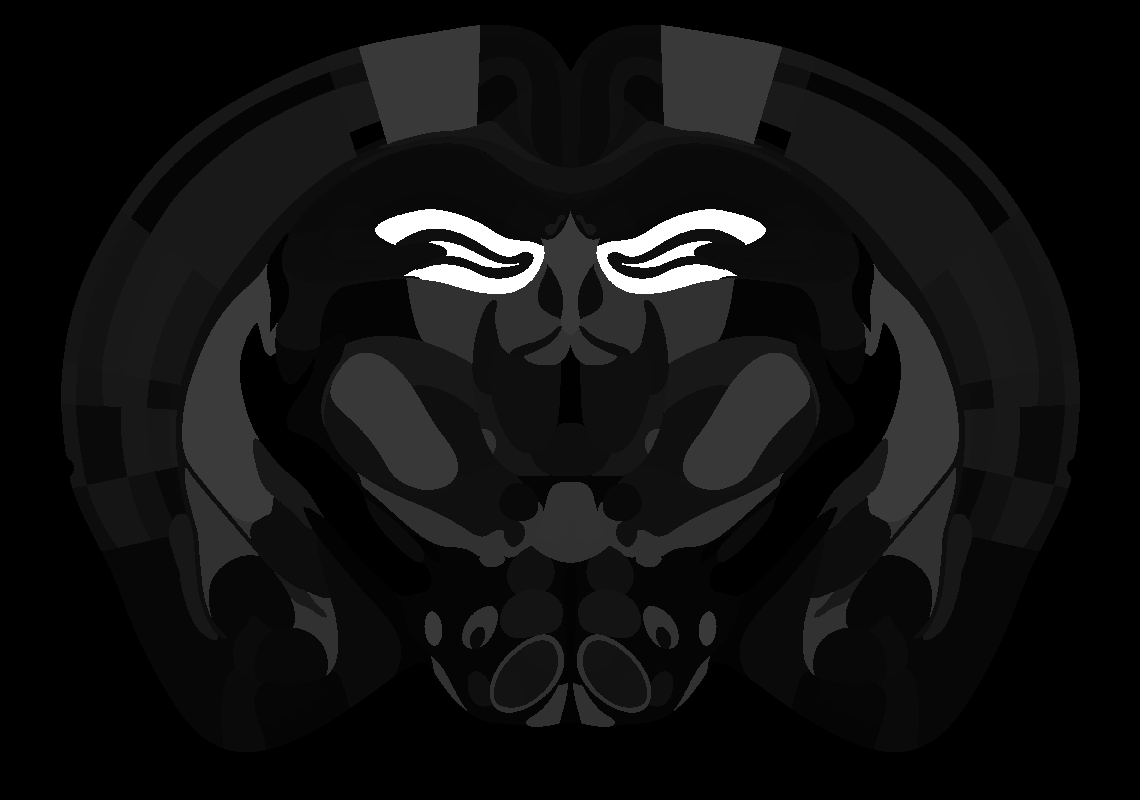

Supplement: Supplementary file 6 — Supplementary Data 4 [file 41467_2019_13057_MOESM6_ESM.zip › Suppl_File1_Labels/62_AP-1.8.tif]

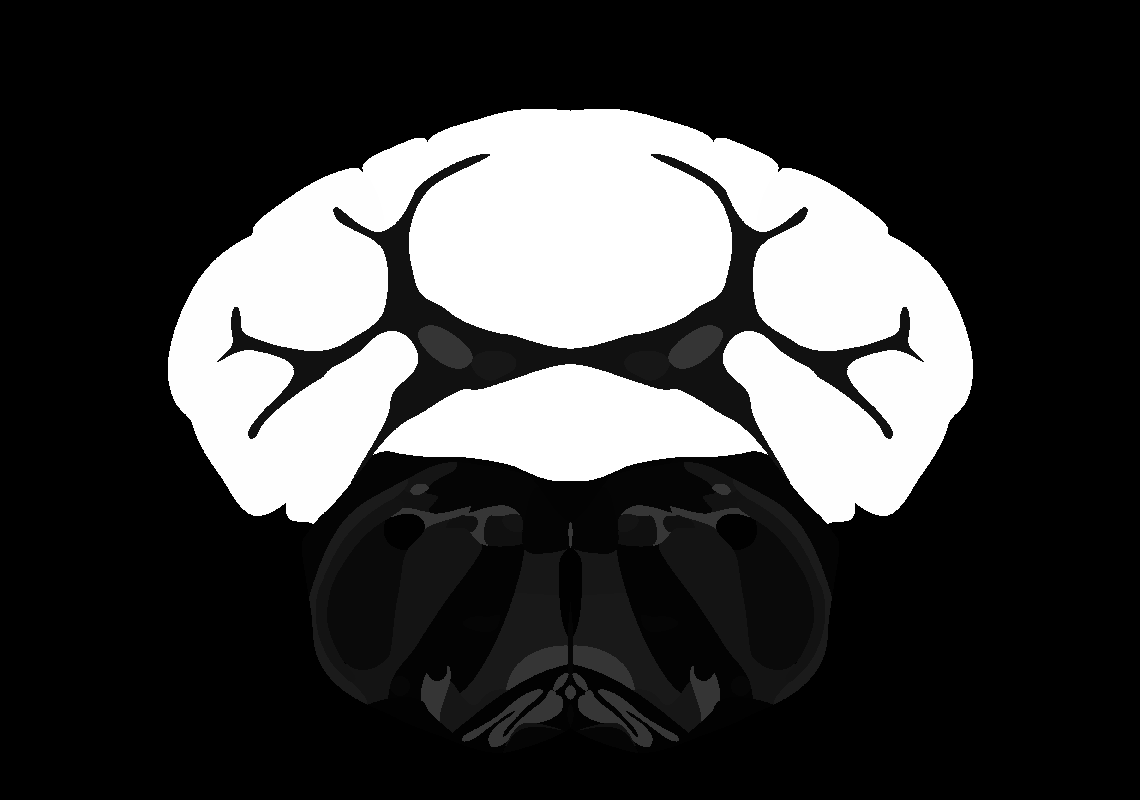

Supplement: Supplementary file 6 — Supplementary Data 4 [file 41467_2019_13057_MOESM6_ESM.zip › Suppl_File1_Labels/112_AP-6.8.tif]

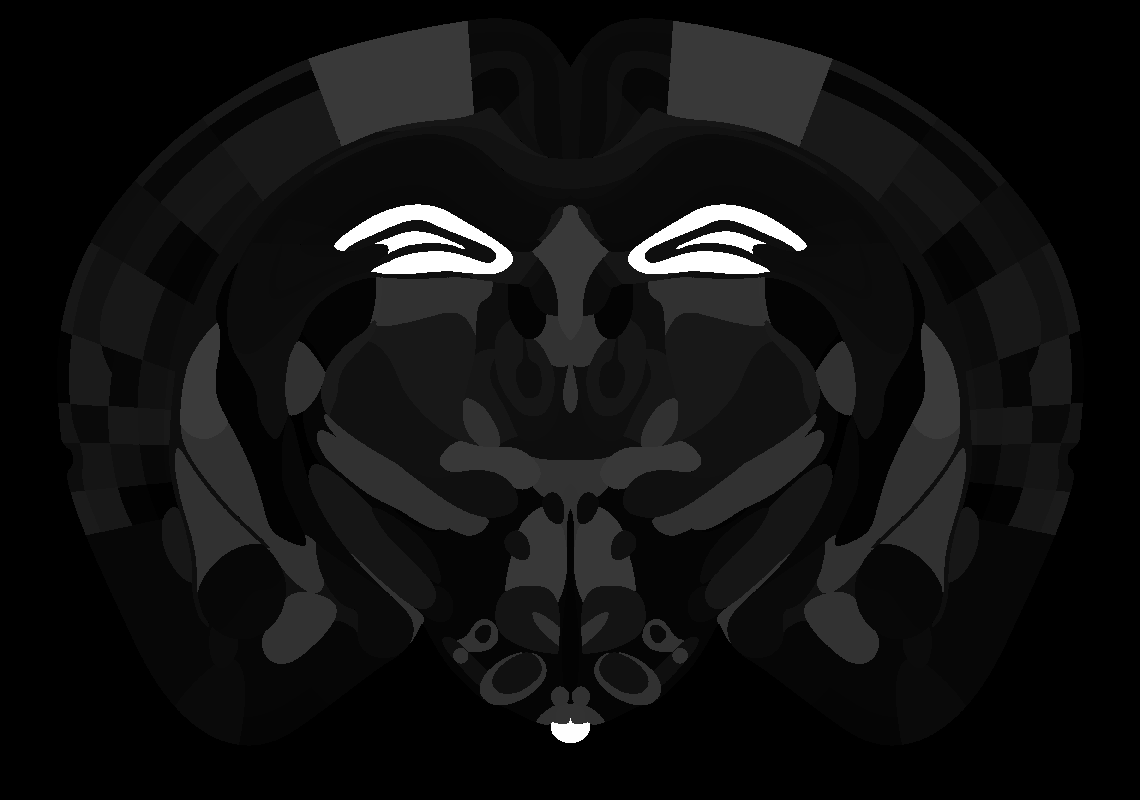

Supplement: Supplementary file 6 — Supplementary Data 4 [file 41467_2019_13057_MOESM6_ESM.zip › Suppl_File1_Labels/66_AP-2.2.tif]

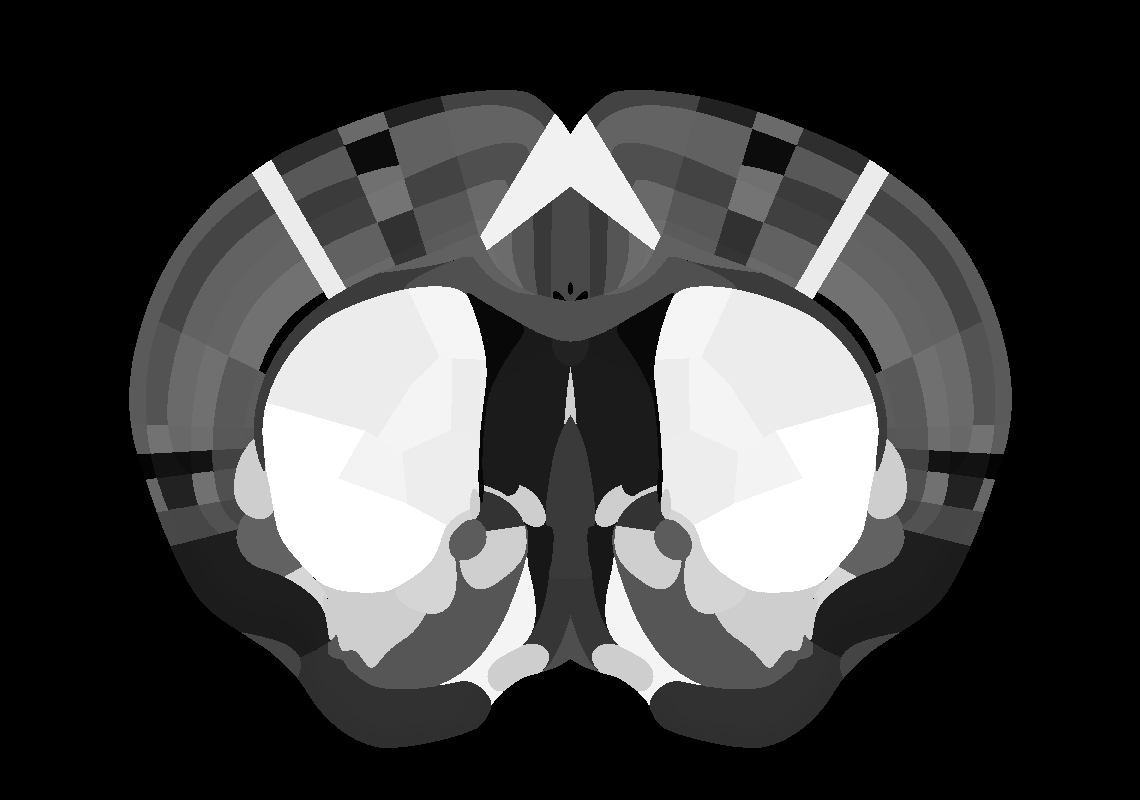

Supplement: Supplementary file 6 — Supplementary Data 4 [file 41467_2019_13057_MOESM6_ESM.zip › Suppl_File1_Labels/39_AP+0.5.tif]

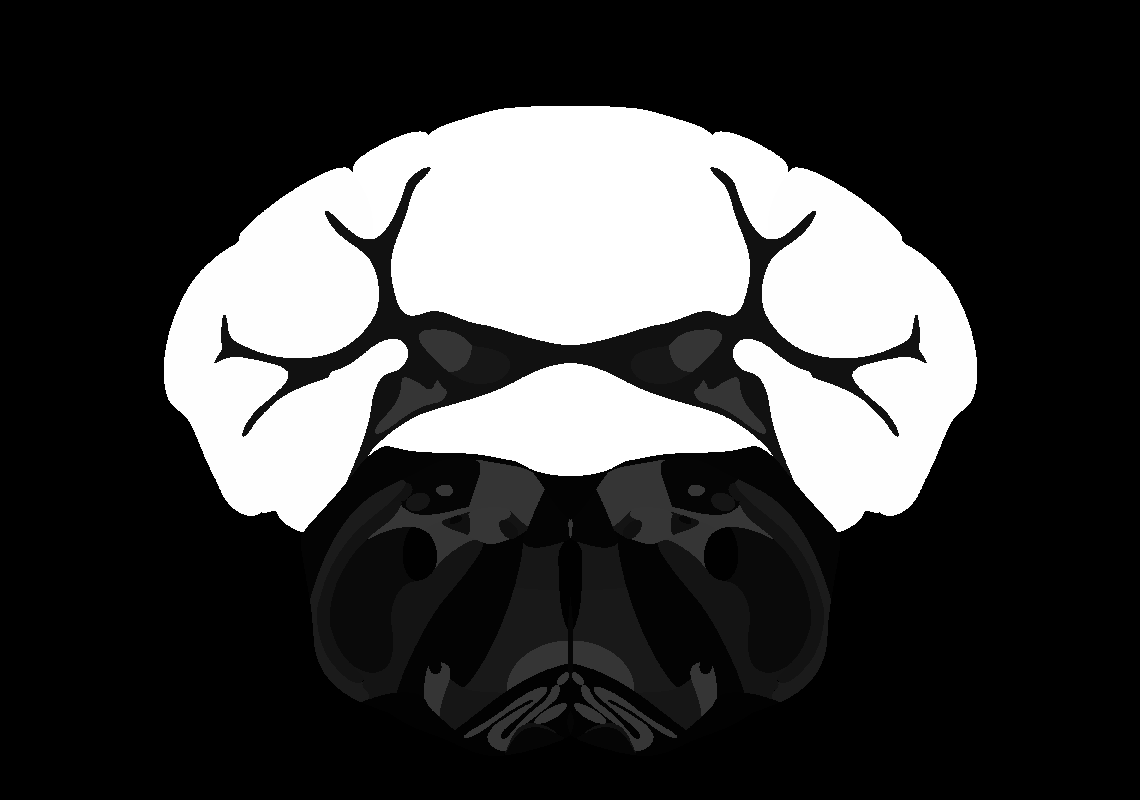

Supplement: Supplementary file 6 — Supplementary Data 4 [file 41467_2019_13057_MOESM6_ESM.zip › Suppl_File1_Labels/111_AP-6.7.tif]

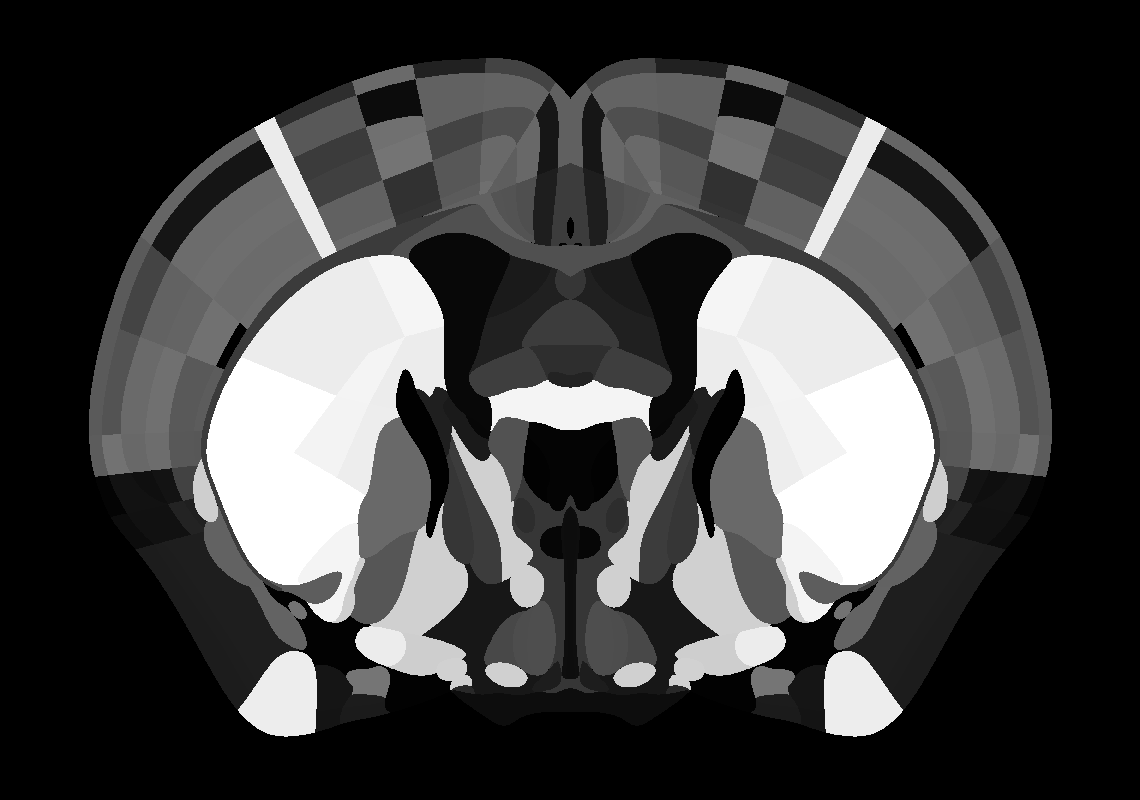

Supplement: Supplementary file 6 — Supplementary Data 4 [file 41467_2019_13057_MOESM6_ESM.zip › Suppl_File1_Labels/48_AP-0.4.tif]

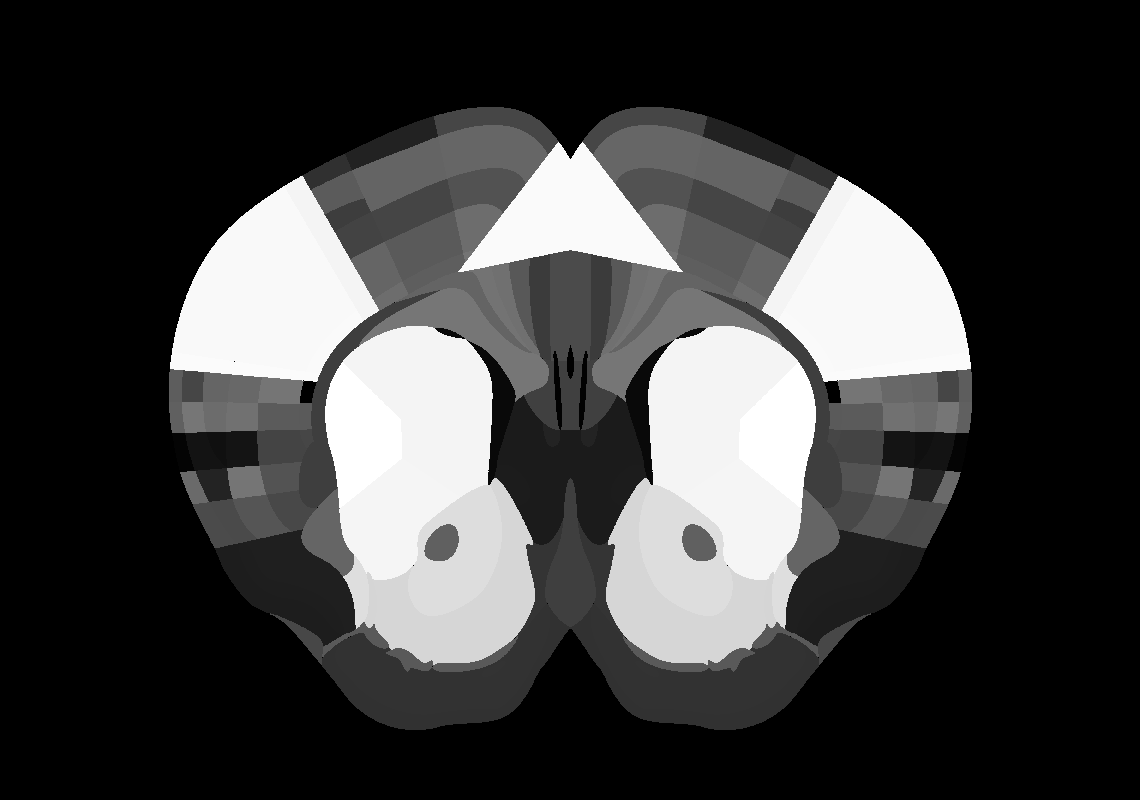

Supplement: Supplementary file 6 — Supplementary Data 4 [file 41467_2019_13057_MOESM6_ESM.zip › Suppl_File1_Labels/33_AP+1.1.tif]
