# Supplementary figures and images for: Enhanced and unified anatomical labeling for a common mouse brain atlas (part 3 of 3)
Source: Nat Commun. 2019 Nov 7;10:5067. doi: 10.1038/s41467-019-13057-w (PMC6838086; doi:10.1038/s41467-019-13057-w)

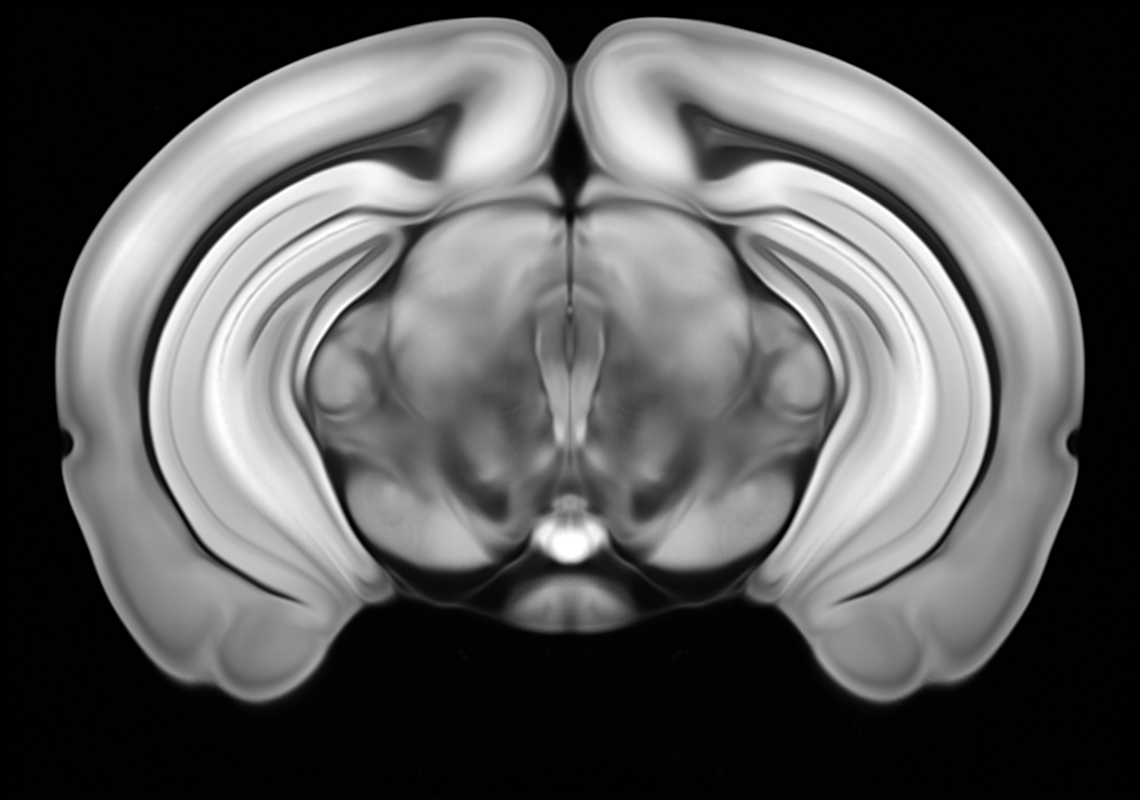

Supplement: Supplementary file 7 — Supplementary Data 5 [file 41467_2019_13057_MOESM7_ESM.zip › Suppl_File2_CCFbackground/AllenCCF_Z075.tif]

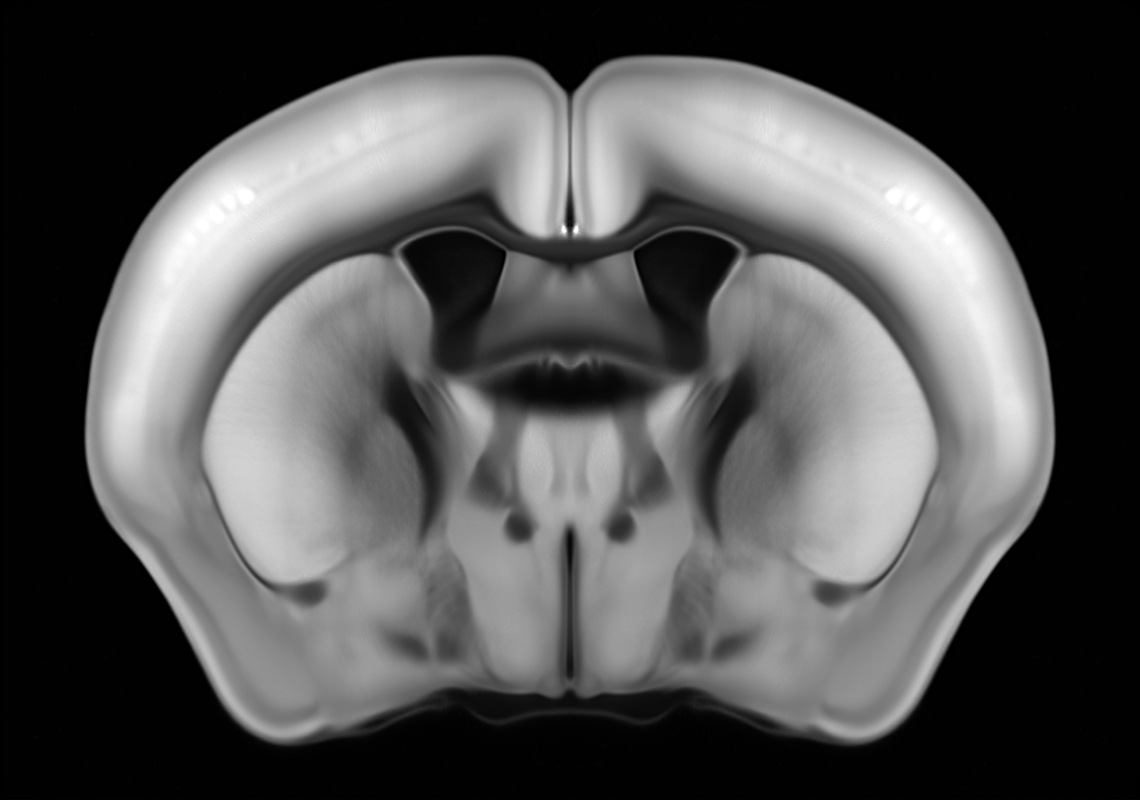

Supplement: Supplementary file 7 — Supplementary Data 5 [file 41467_2019_13057_MOESM7_ESM.zip › Suppl_File2_CCFbackground/AllenCCF_Z049.tif]

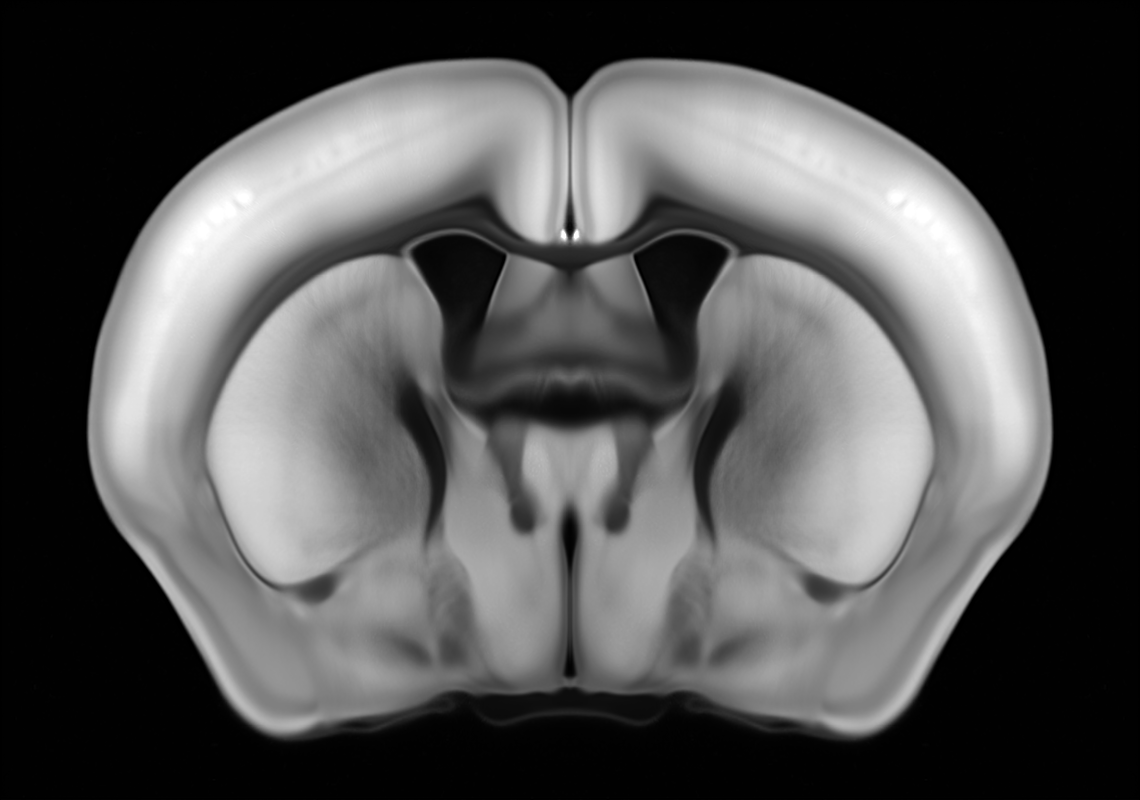

Supplement: Supplementary file 7 — Supplementary Data 5 [file 41467_2019_13057_MOESM7_ESM.zip › Suppl_File2_CCFbackground/AllenCCF_Z048.tif]

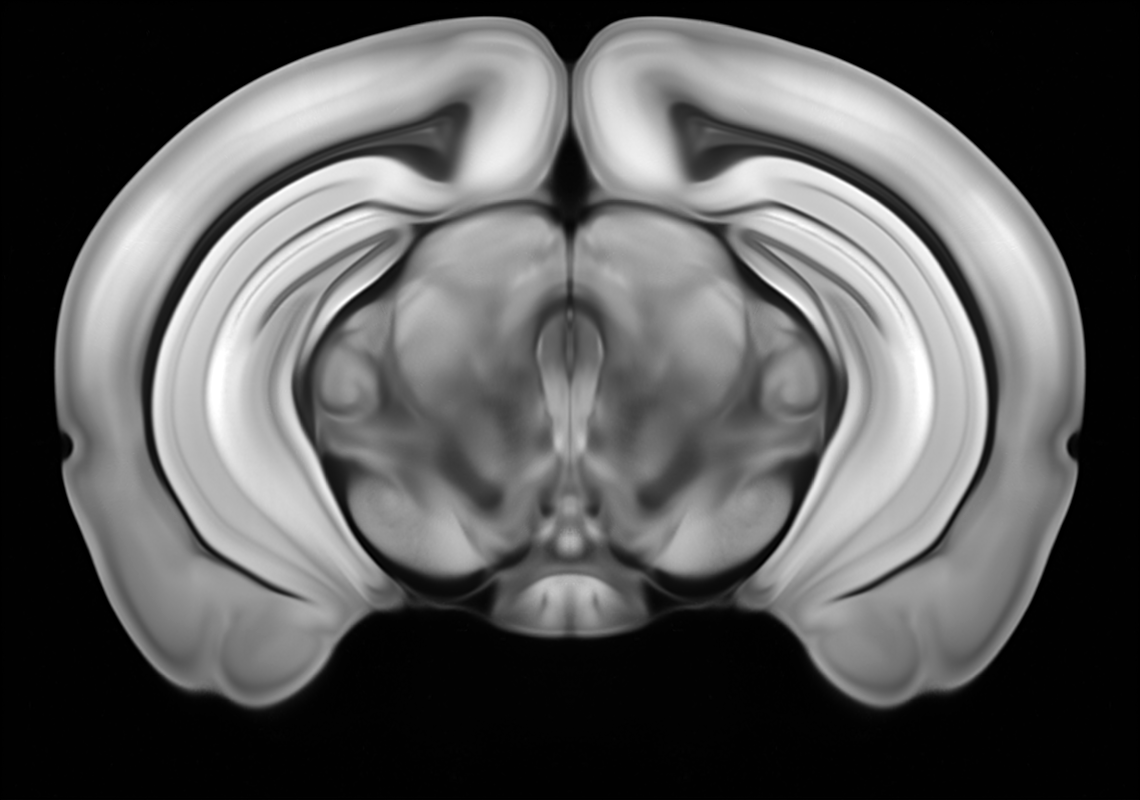

Supplement: Supplementary file 7 — Supplementary Data 5 [file 41467_2019_13057_MOESM7_ESM.zip › Suppl_File2_CCFbackground/AllenCCF_Z074.tif]

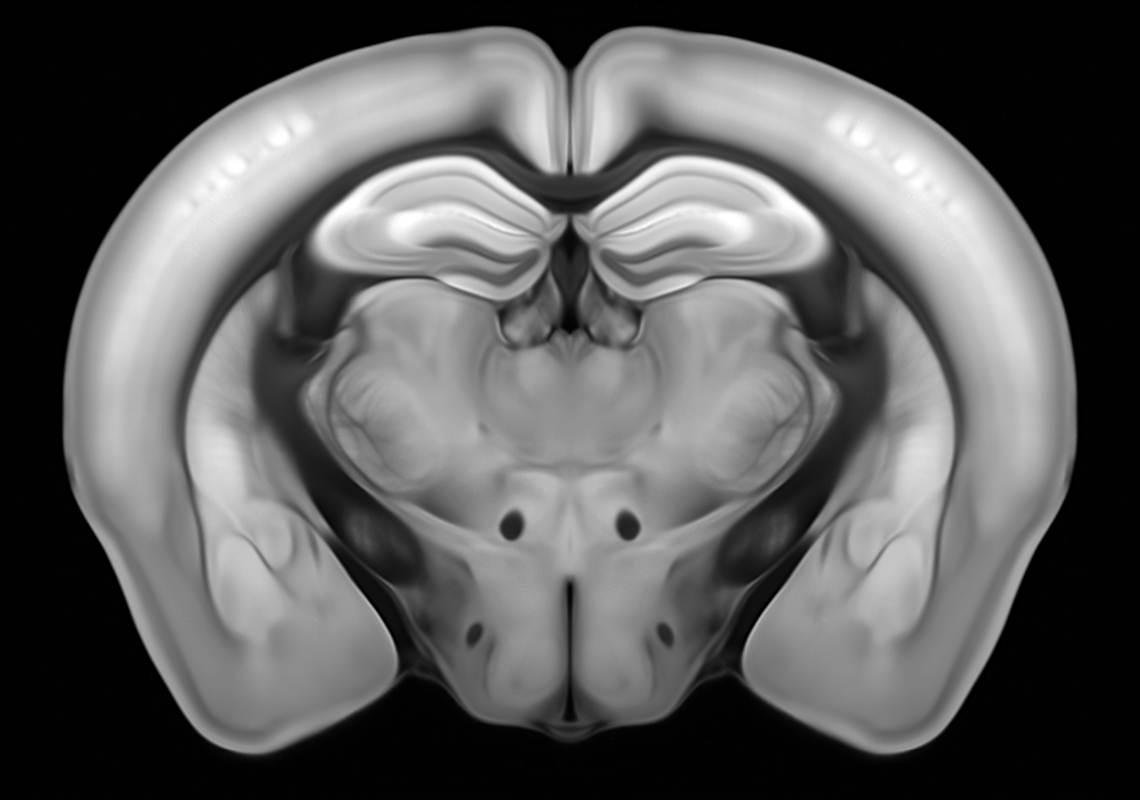

Supplement: Supplementary file 7 — Supplementary Data 5 [file 41467_2019_13057_MOESM7_ESM.zip › Suppl_File2_CCFbackground/AllenCCF_Z060.tif]

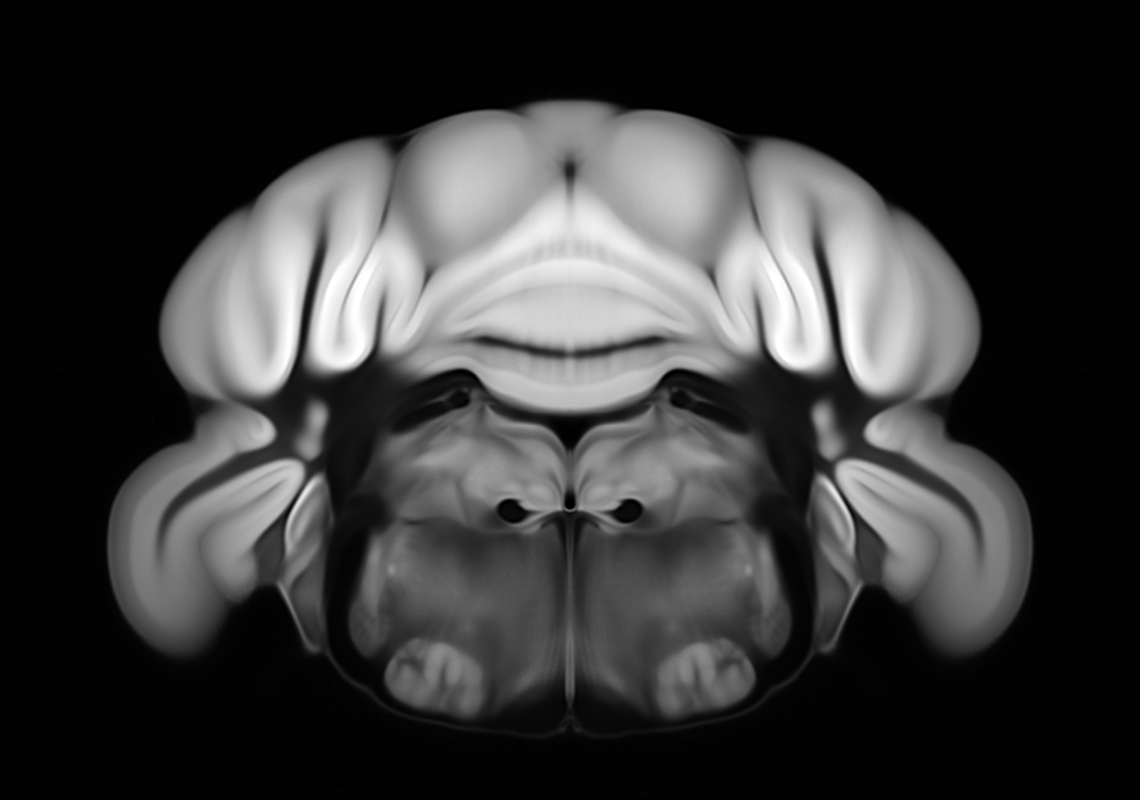

Supplement: Supplementary file 7 — Supplementary Data 5 [file 41467_2019_13057_MOESM7_ESM.zip › Suppl_File2_CCFbackground/AllenCCF_Z100.tif]

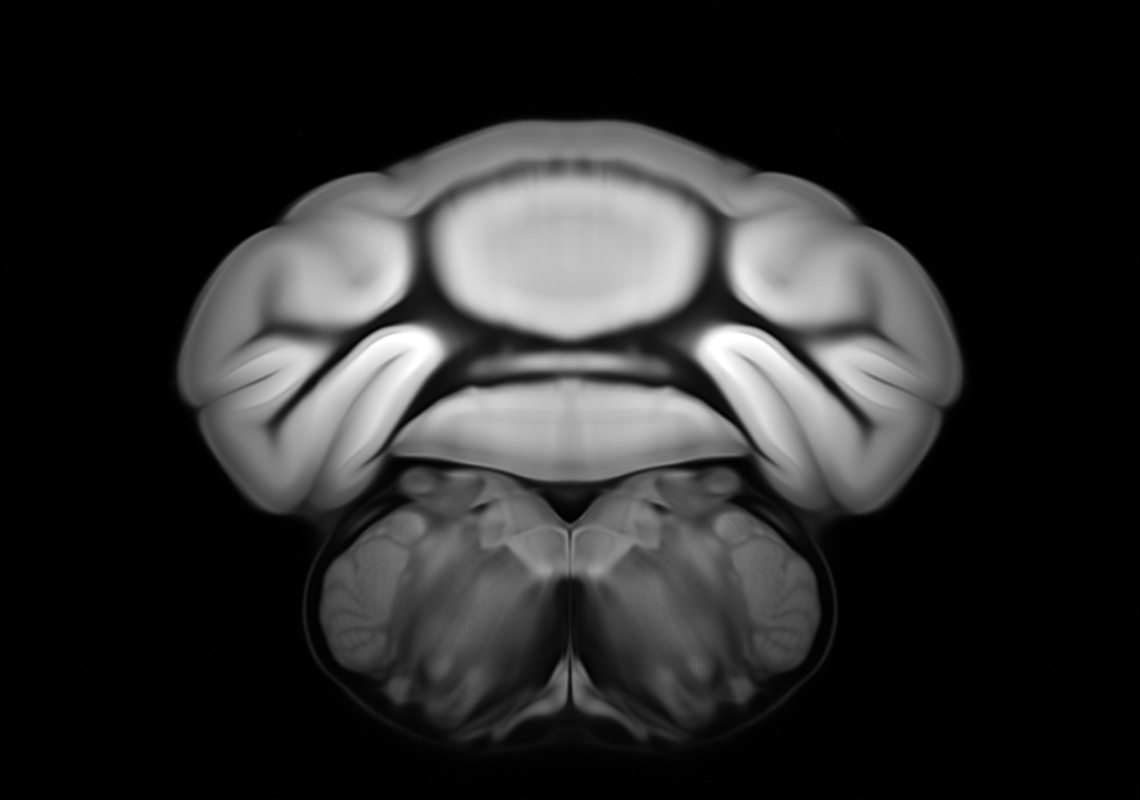

Supplement: Supplementary file 7 — Supplementary Data 5 [file 41467_2019_13057_MOESM7_ESM.zip › Suppl_File2_CCFbackground/AllenCCF_Z114.tif]

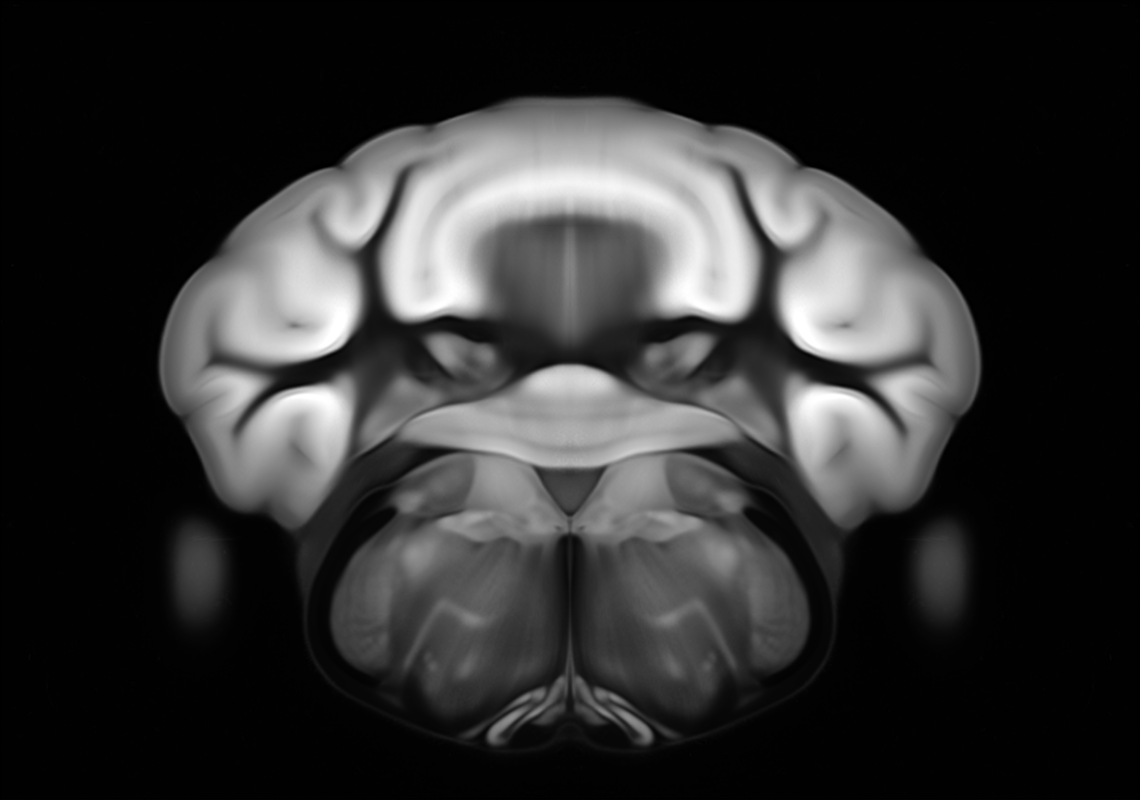

Supplement: Supplementary file 7 — Supplementary Data 5 [file 41467_2019_13057_MOESM7_ESM.zip › Suppl_File2_CCFbackground/AllenCCF_Z110.tif]

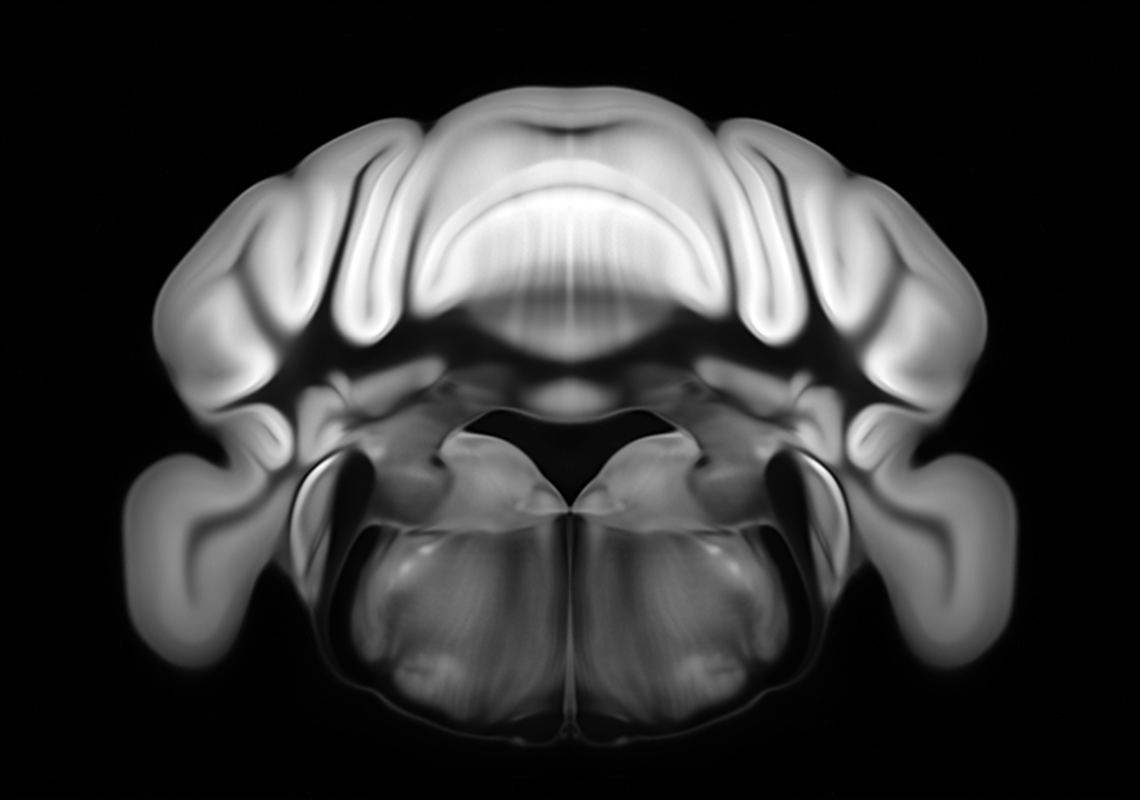

Supplement: Supplementary file 7 — Supplementary Data 5 [file 41467_2019_13057_MOESM7_ESM.zip › Suppl_File2_CCFbackground/AllenCCF_Z104.tif]

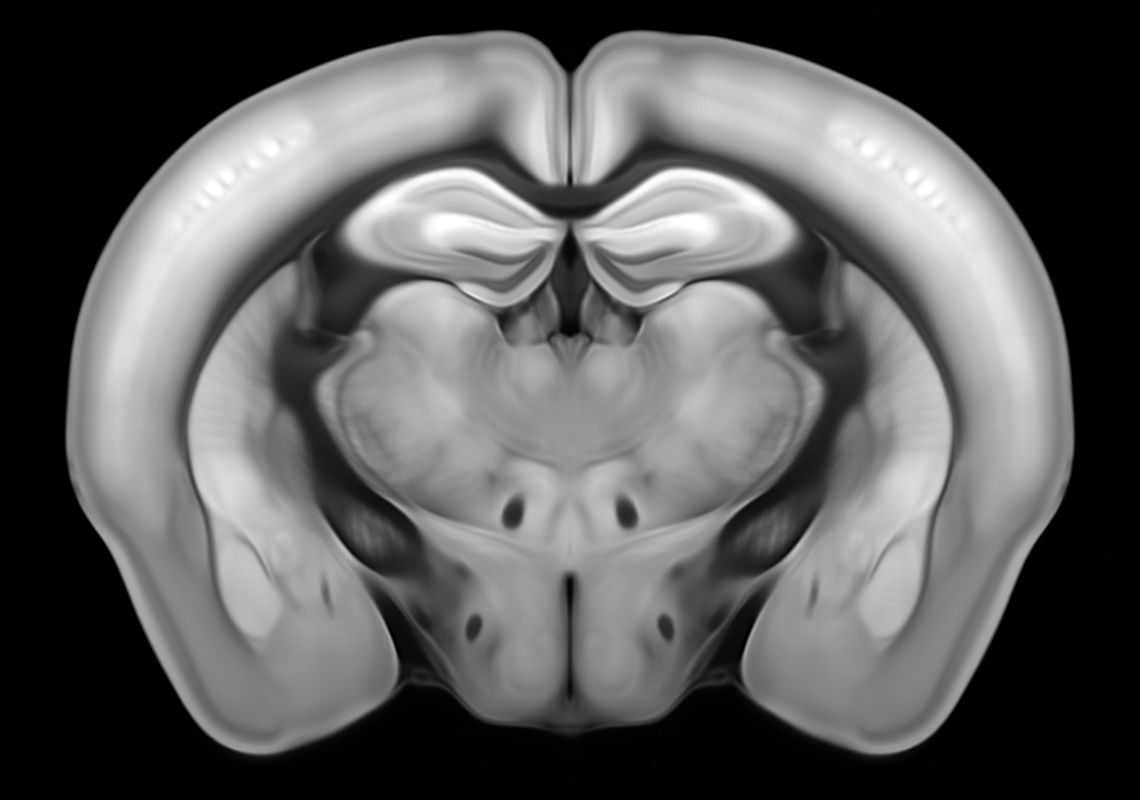

Supplement: Supplementary file 7 — Supplementary Data 5 [file 41467_2019_13057_MOESM7_ESM.zip › Suppl_File2_CCFbackground/AllenCCF_Z058.tif]

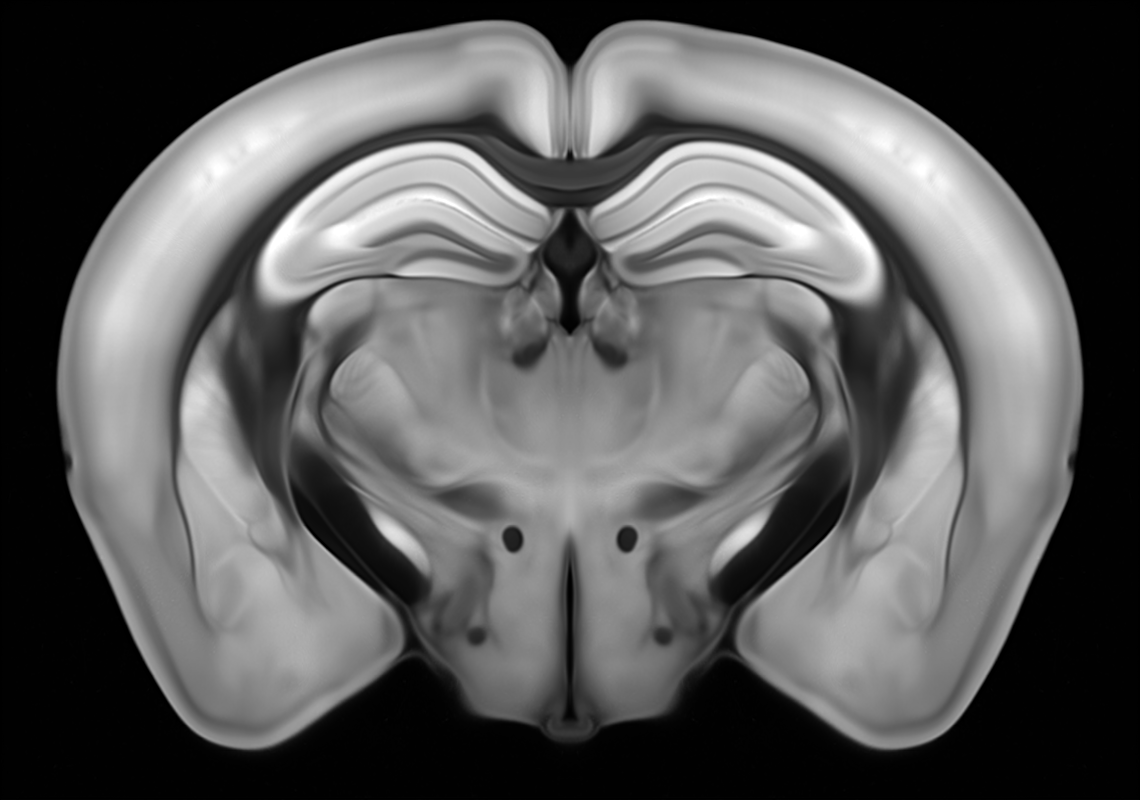

Supplement: Supplementary file 7 — Supplementary Data 5 [file 41467_2019_13057_MOESM7_ESM.zip › Suppl_File2_CCFbackground/AllenCCF_Z064.tif]

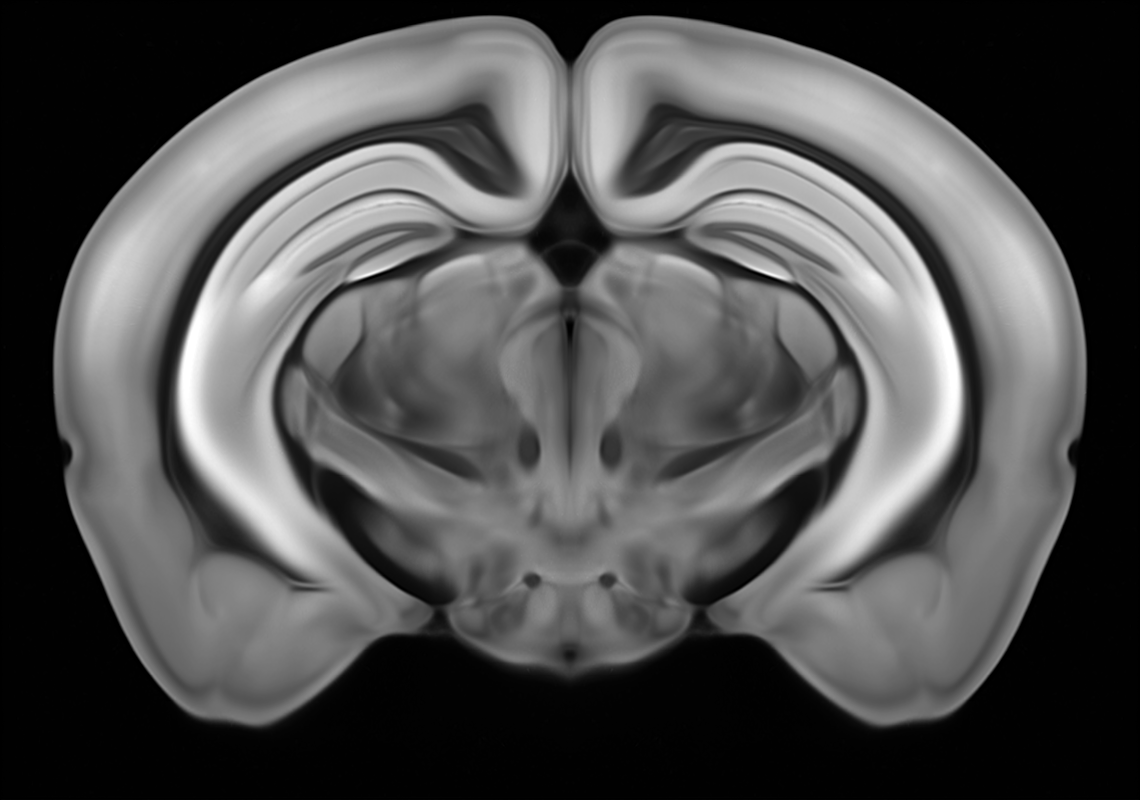

Supplement: Supplementary file 7 — Supplementary Data 5 [file 41467_2019_13057_MOESM7_ESM.zip › Suppl_File2_CCFbackground/AllenCCF_Z070.tif]

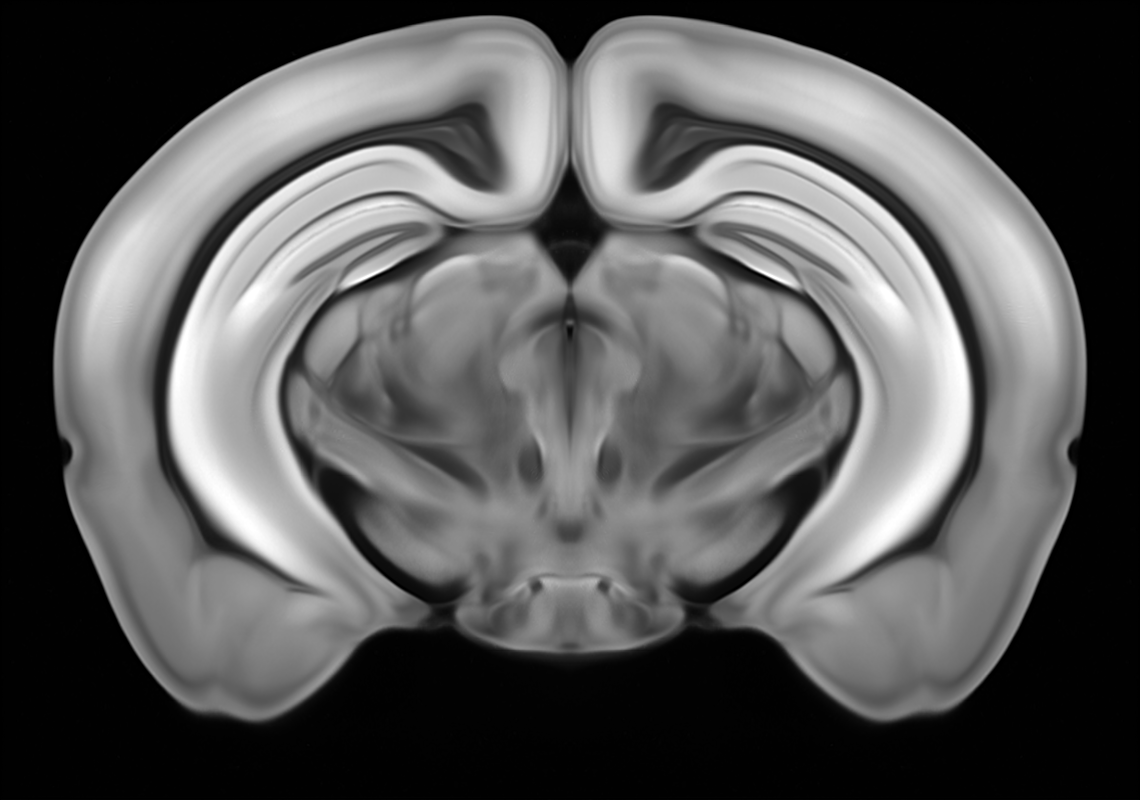

Supplement: Supplementary file 7 — Supplementary Data 5 [file 41467_2019_13057_MOESM7_ESM.zip › Suppl_File2_CCFbackground/AllenCCF_Z071.tif]

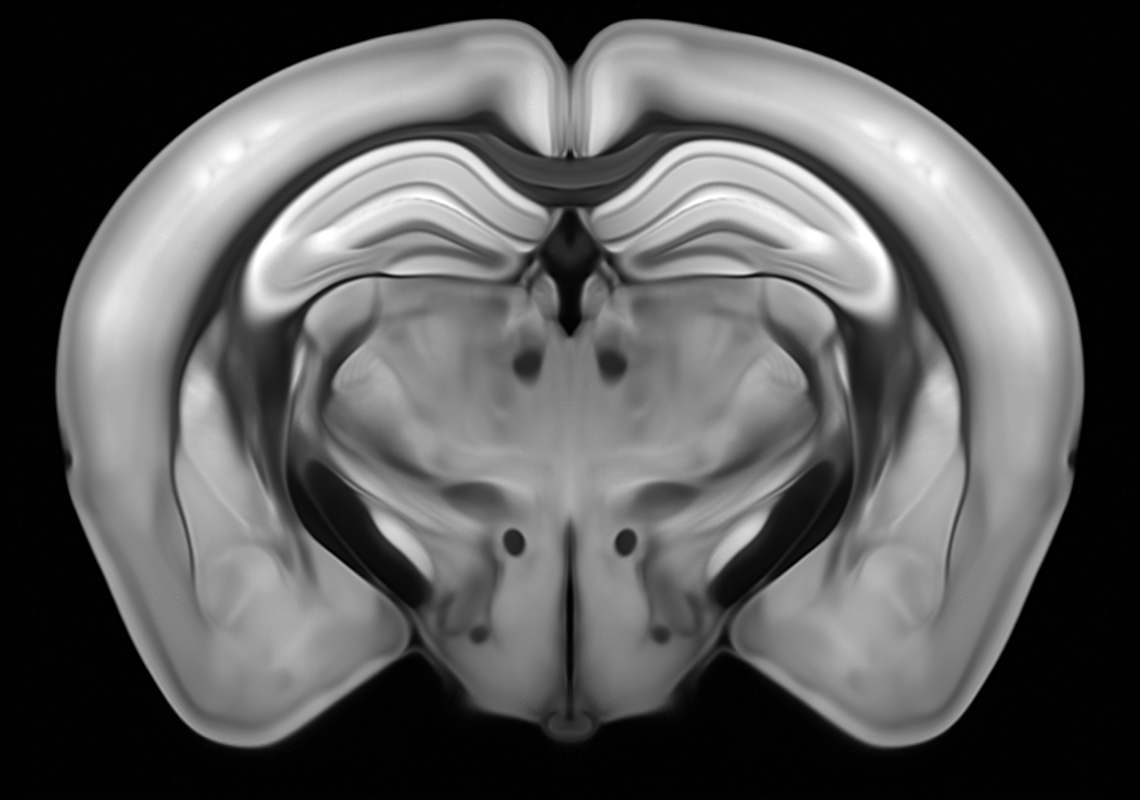

Supplement: Supplementary file 7 — Supplementary Data 5 [file 41467_2019_13057_MOESM7_ESM.zip › Suppl_File2_CCFbackground/AllenCCF_Z065.tif]

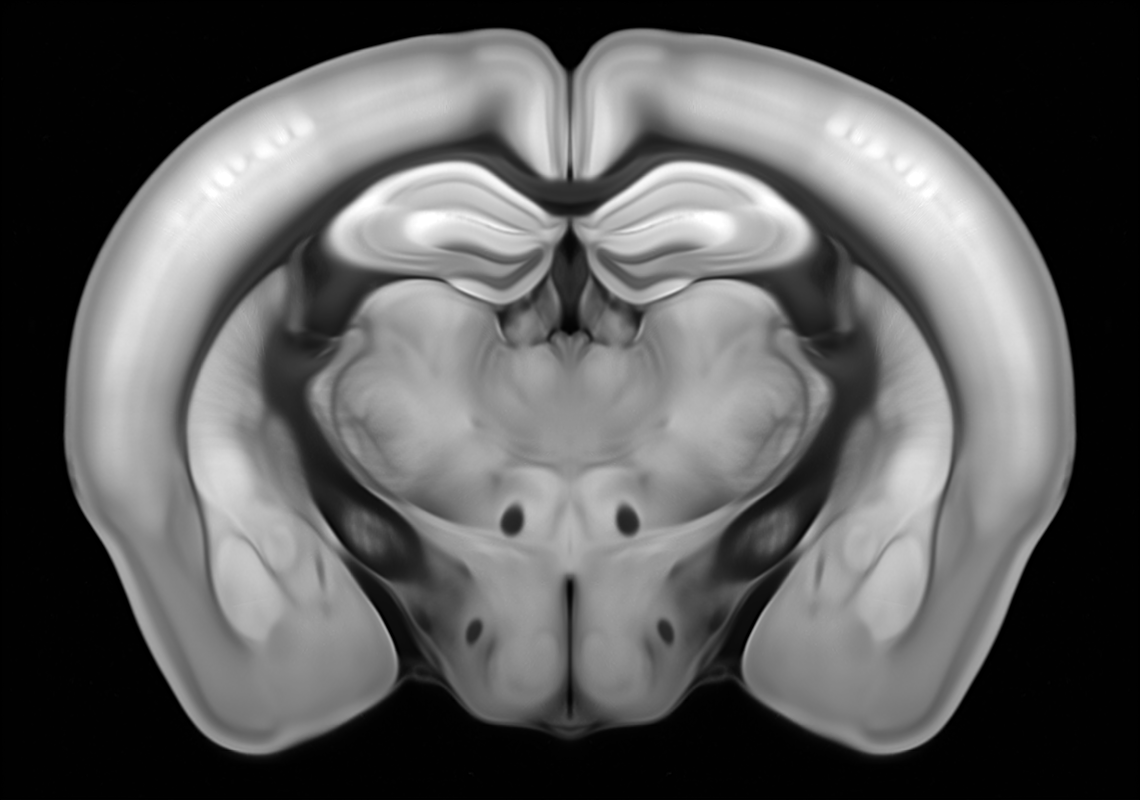

Supplement: Supplementary file 7 — Supplementary Data 5 [file 41467_2019_13057_MOESM7_ESM.zip › Suppl_File2_CCFbackground/AllenCCF_Z059.tif]

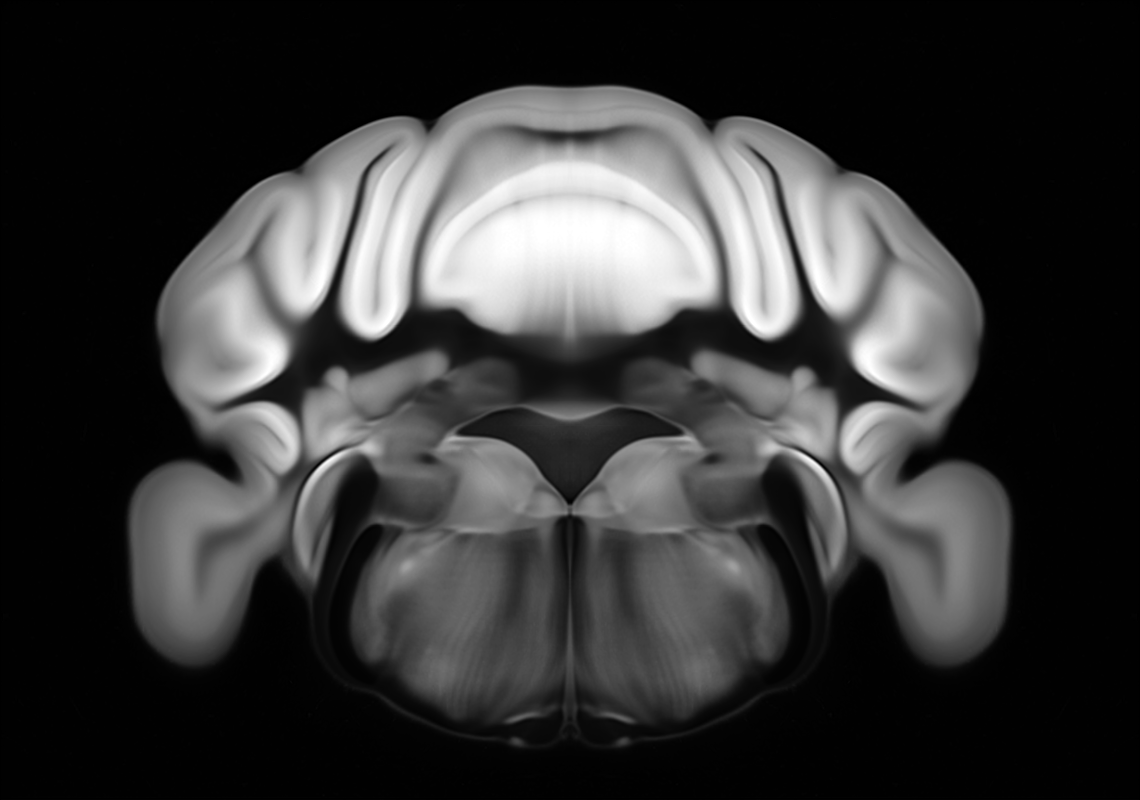

Supplement: Supplementary file 7 — Supplementary Data 5 [file 41467_2019_13057_MOESM7_ESM.zip › Suppl_File2_CCFbackground/AllenCCF_Z105.tif]

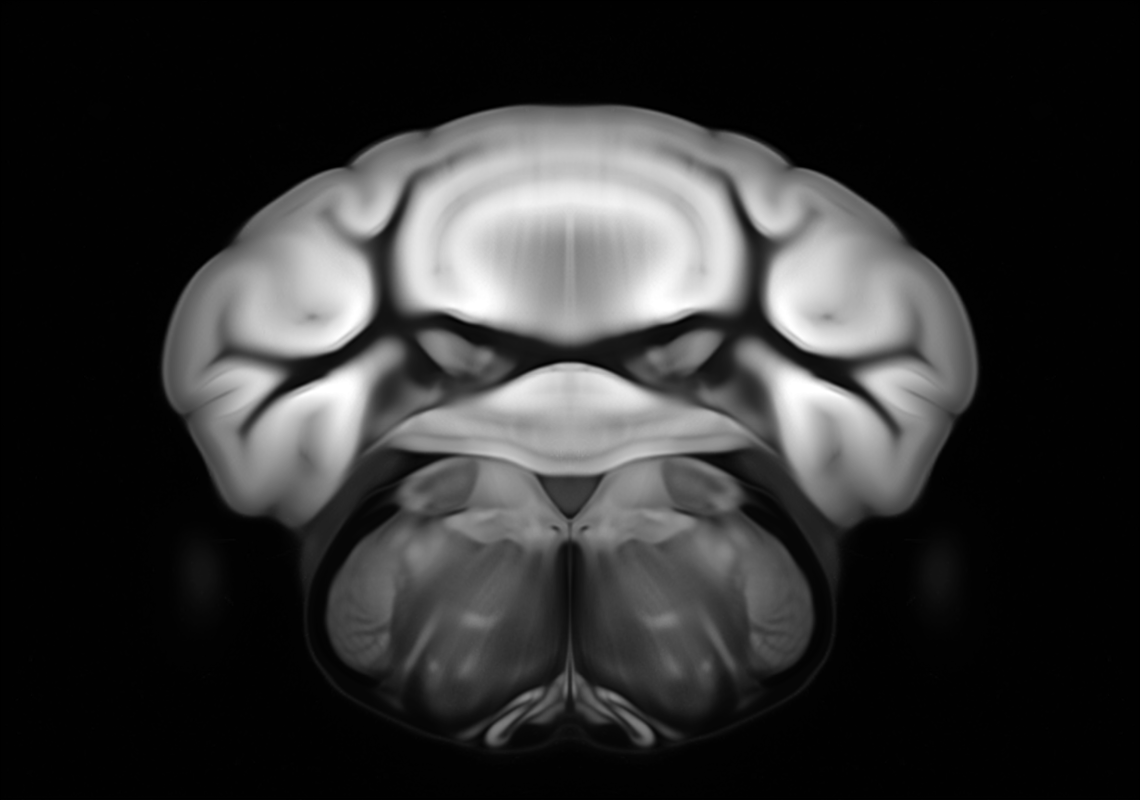

Supplement: Supplementary file 7 — Supplementary Data 5 [file 41467_2019_13057_MOESM7_ESM.zip › Suppl_File2_CCFbackground/AllenCCF_Z111.tif]

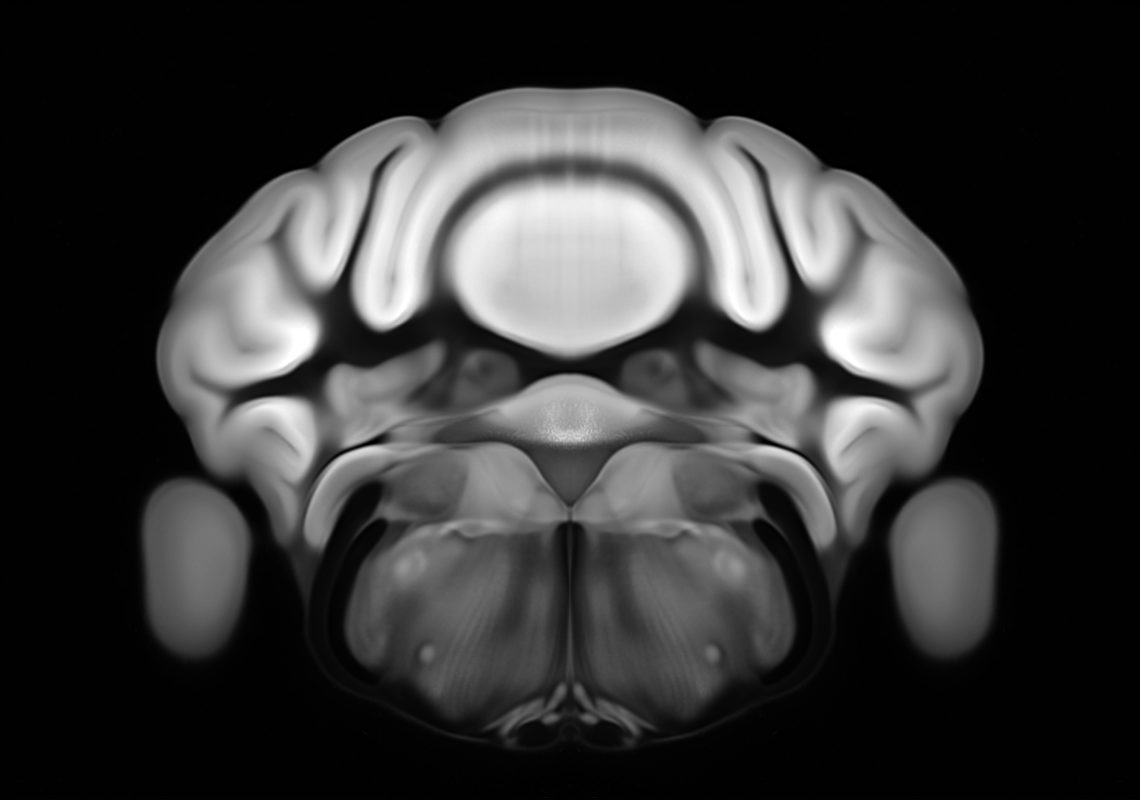

Supplement: Supplementary file 7 — Supplementary Data 5 [file 41467_2019_13057_MOESM7_ESM.zip › Suppl_File2_CCFbackground/AllenCCF_Z107.tif]

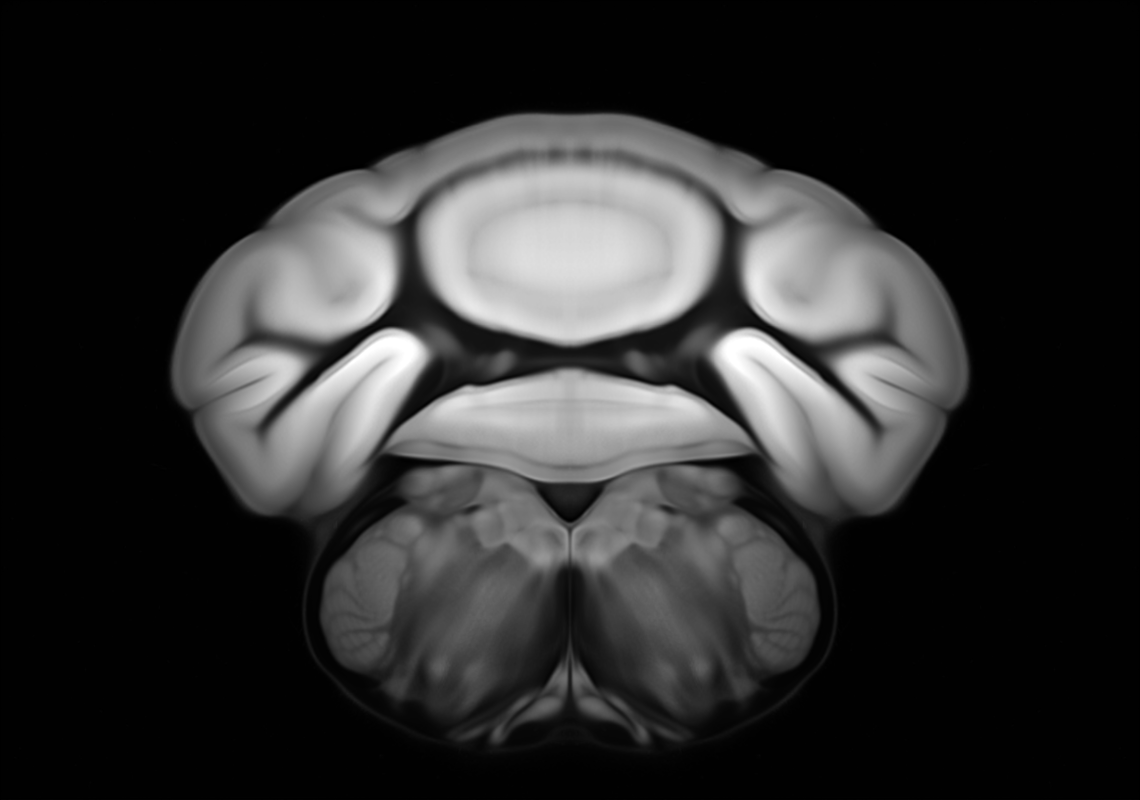

Supplement: Supplementary file 7 — Supplementary Data 5 [file 41467_2019_13057_MOESM7_ESM.zip › Suppl_File2_CCFbackground/AllenCCF_Z113.tif]

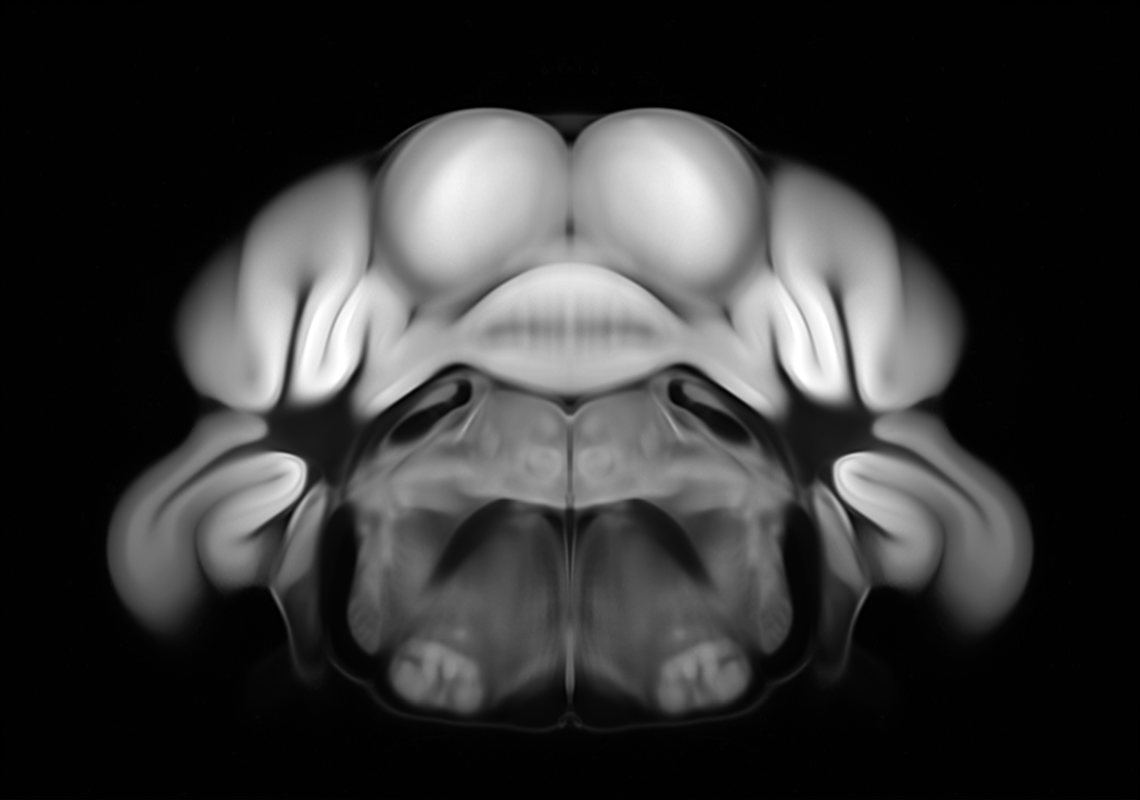

Supplement: Supplementary file 7 — Supplementary Data 5 [file 41467_2019_13057_MOESM7_ESM.zip › Suppl_File2_CCFbackground/AllenCCF_Z098.tif]

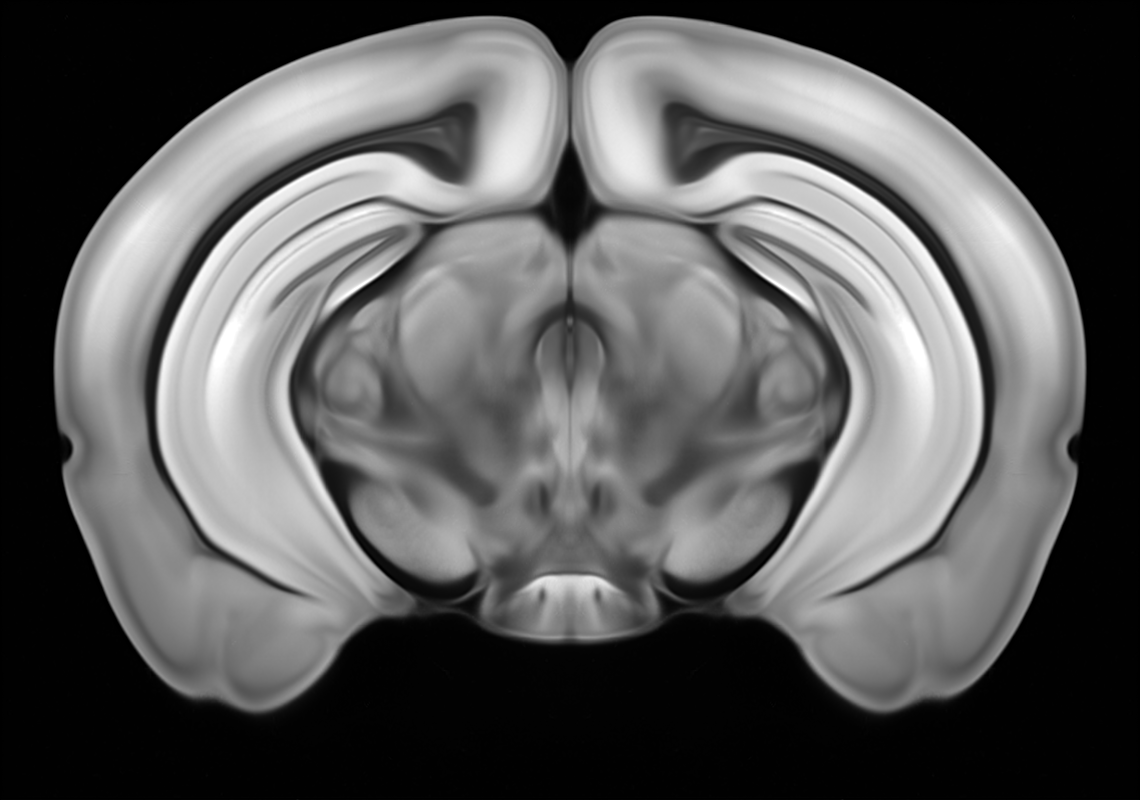

Supplement: Supplementary file 7 — Supplementary Data 5 [file 41467_2019_13057_MOESM7_ESM.zip › Suppl_File2_CCFbackground/AllenCCF_Z073.tif]

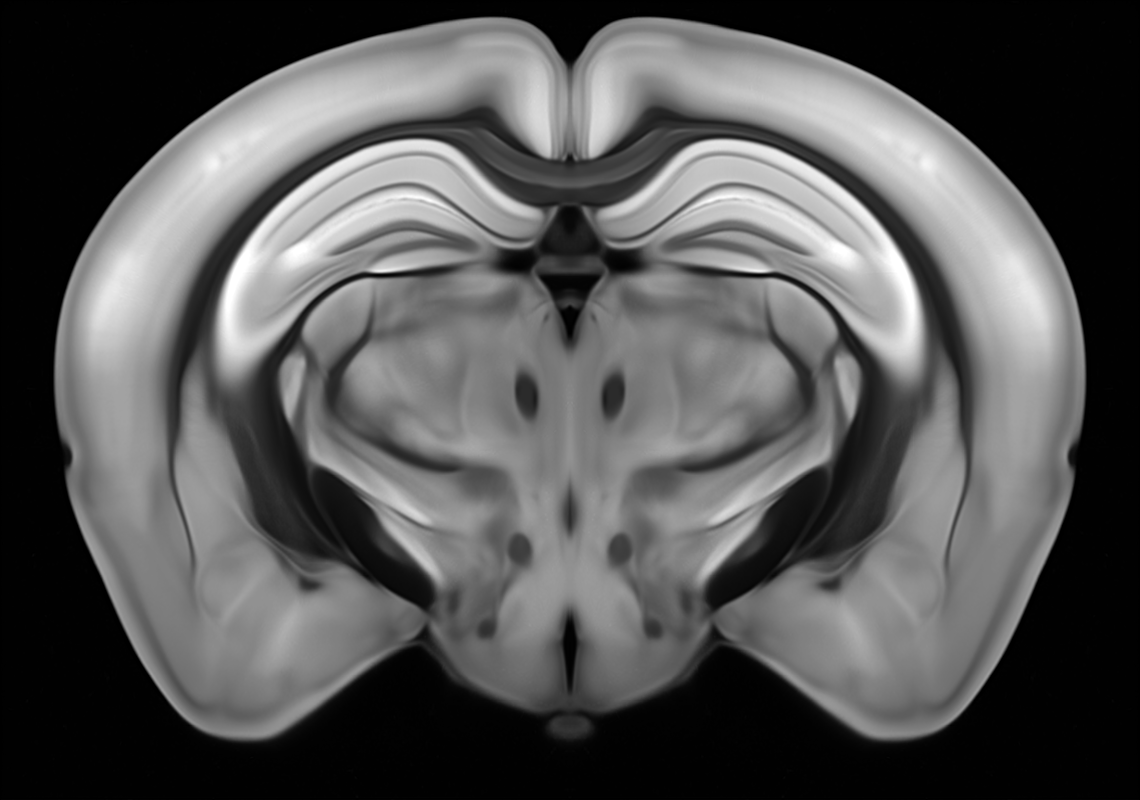

Supplement: Supplementary file 7 — Supplementary Data 5 [file 41467_2019_13057_MOESM7_ESM.zip › Suppl_File2_CCFbackground/AllenCCF_Z067.tif]

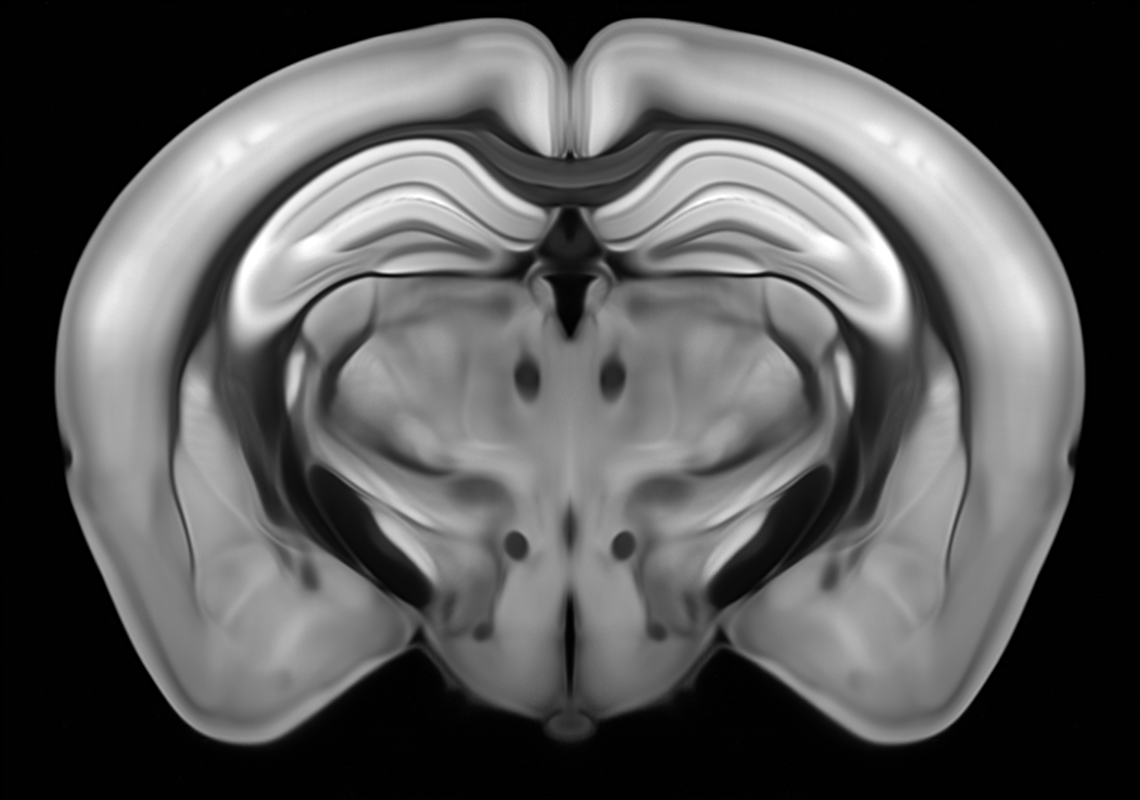

Supplement: Supplementary file 7 — Supplementary Data 5 [file 41467_2019_13057_MOESM7_ESM.zip › Suppl_File2_CCFbackground/AllenCCF_Z066.tif]

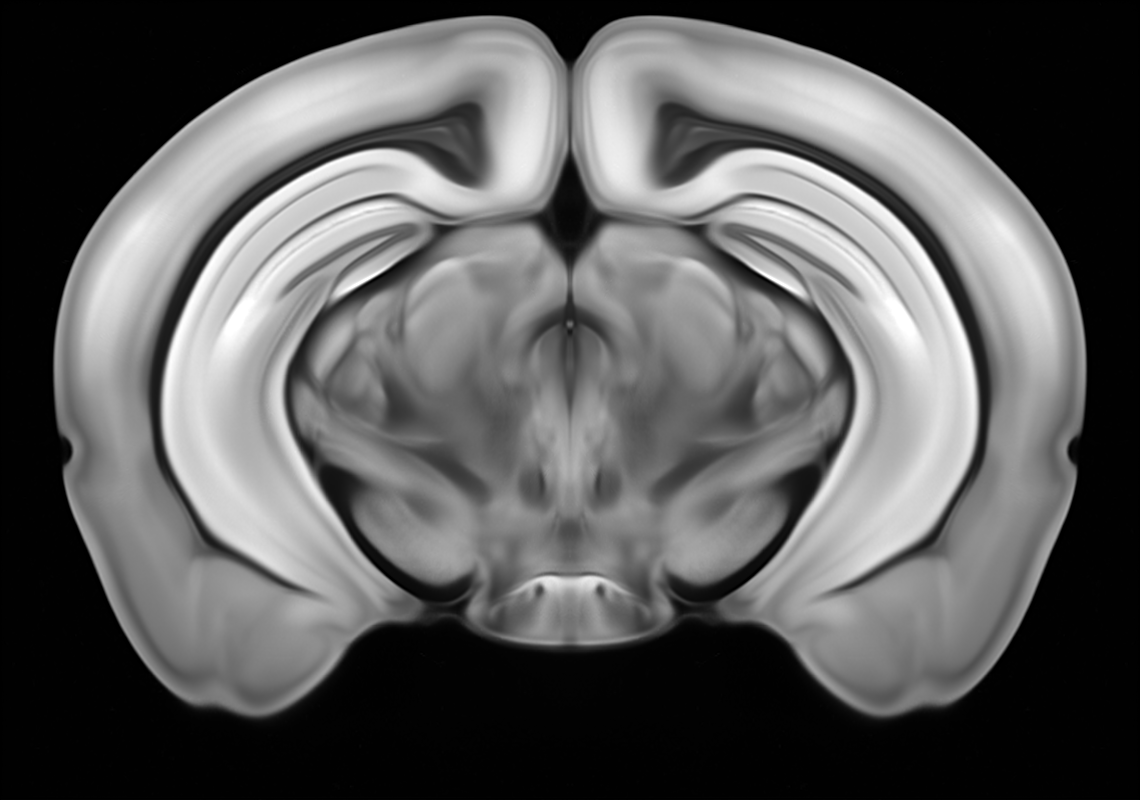

Supplement: Supplementary file 7 — Supplementary Data 5 [file 41467_2019_13057_MOESM7_ESM.zip › Suppl_File2_CCFbackground/AllenCCF_Z072.tif]

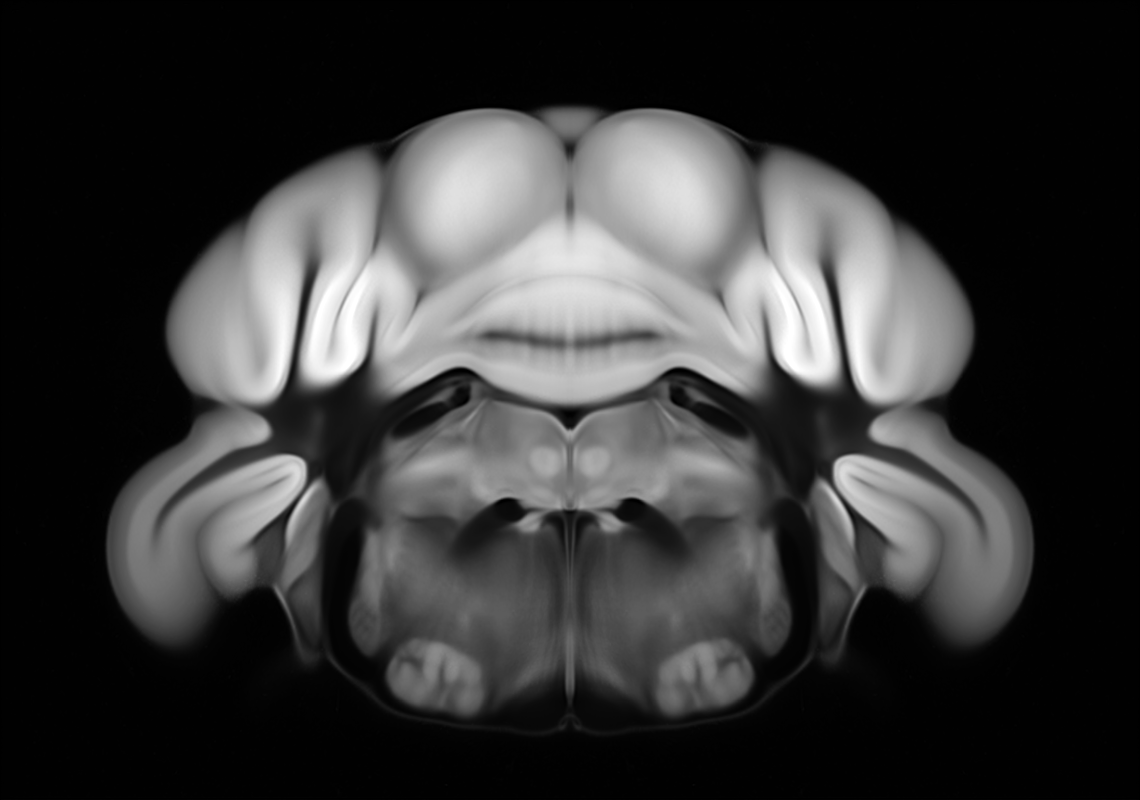

Supplement: Supplementary file 7 — Supplementary Data 5 [file 41467_2019_13057_MOESM7_ESM.zip › Suppl_File2_CCFbackground/AllenCCF_Z099.tif]

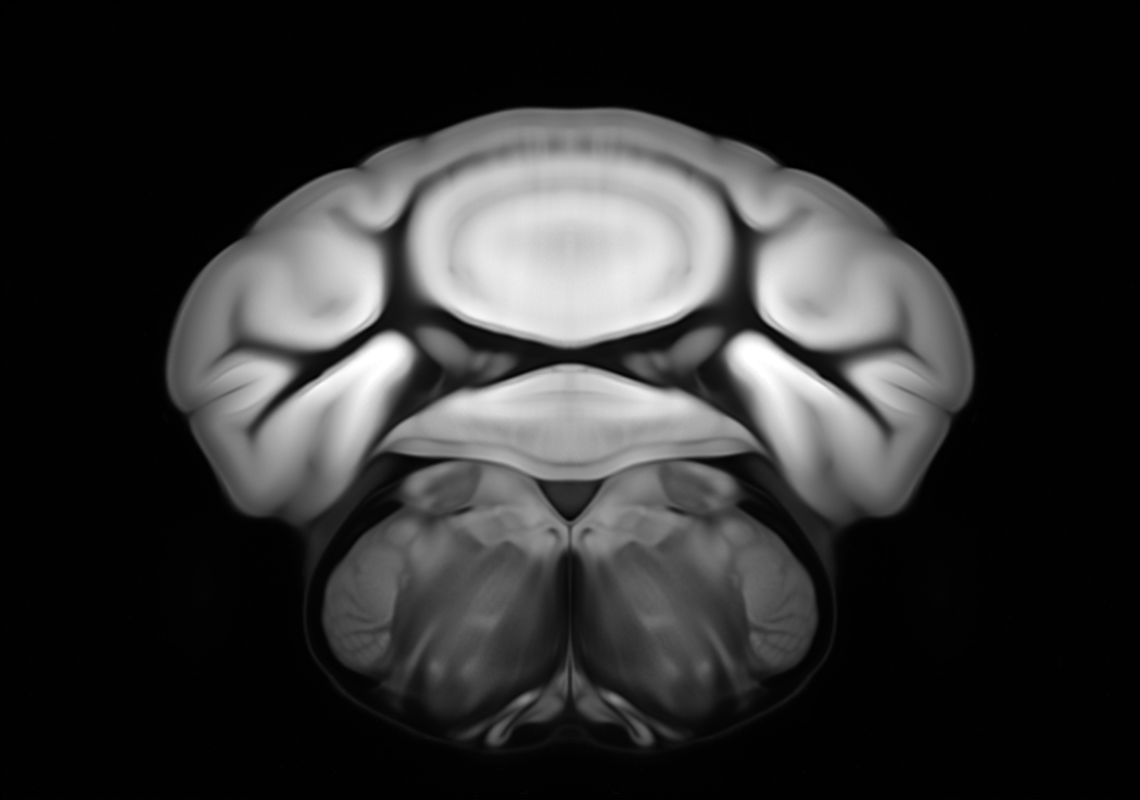

Supplement: Supplementary file 7 — Supplementary Data 5 [file 41467_2019_13057_MOESM7_ESM.zip › Suppl_File2_CCFbackground/AllenCCF_Z112.tif]

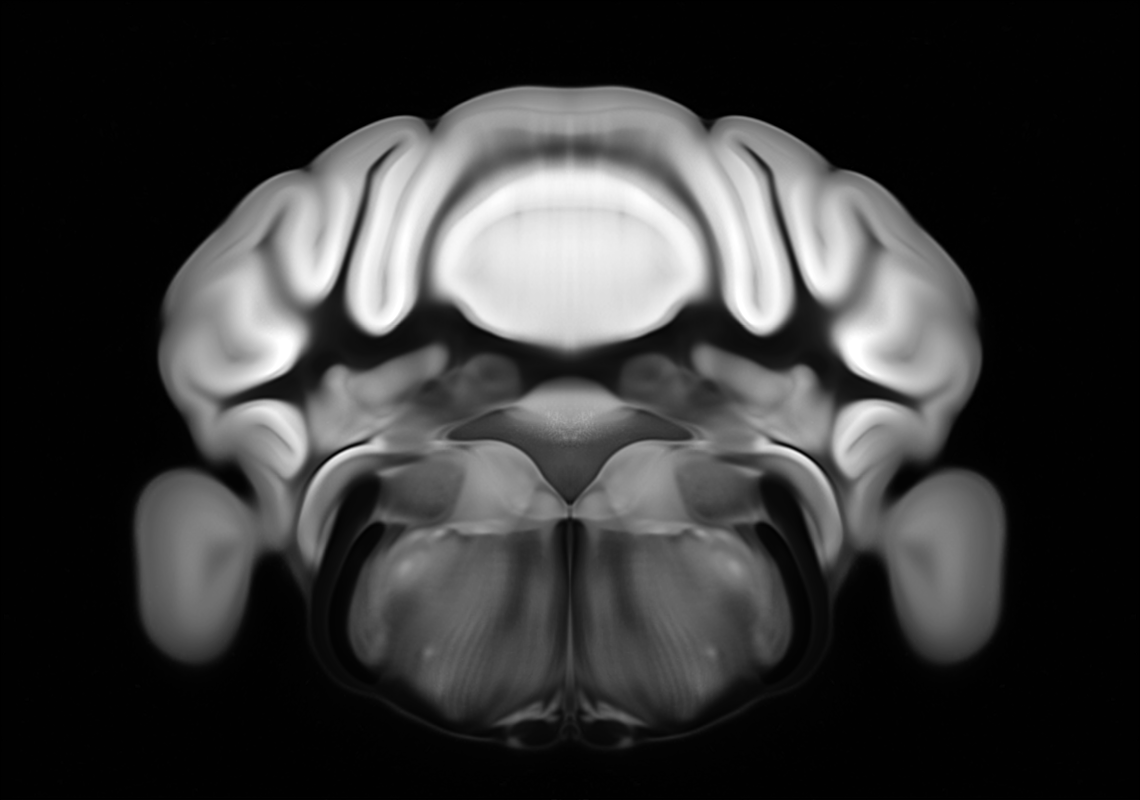

Supplement: Supplementary file 7 — Supplementary Data 5 [file 41467_2019_13057_MOESM7_ESM.zip › Suppl_File2_CCFbackground/AllenCCF_Z106.tif]

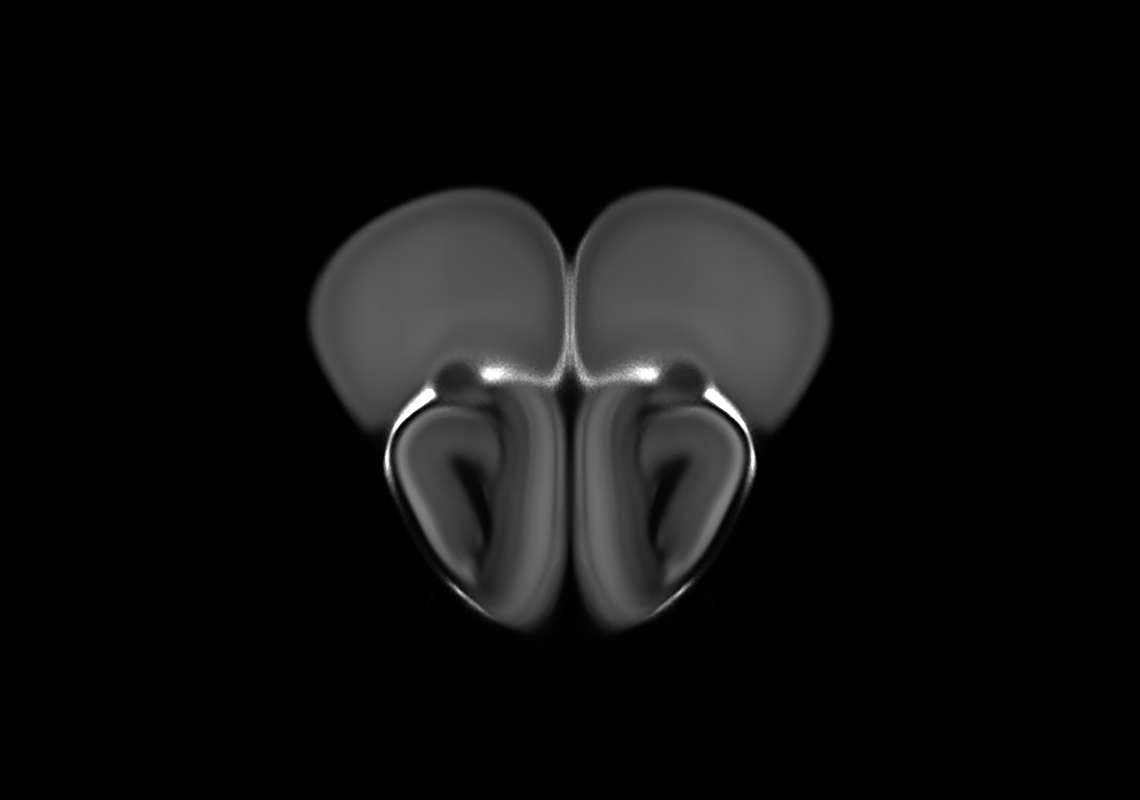

Supplement: Supplementary file 7 — Supplementary Data 5 [file 41467_2019_13057_MOESM7_ESM.zip › Suppl_File2_CCFbackground/AllenCCF_Z015.tif]

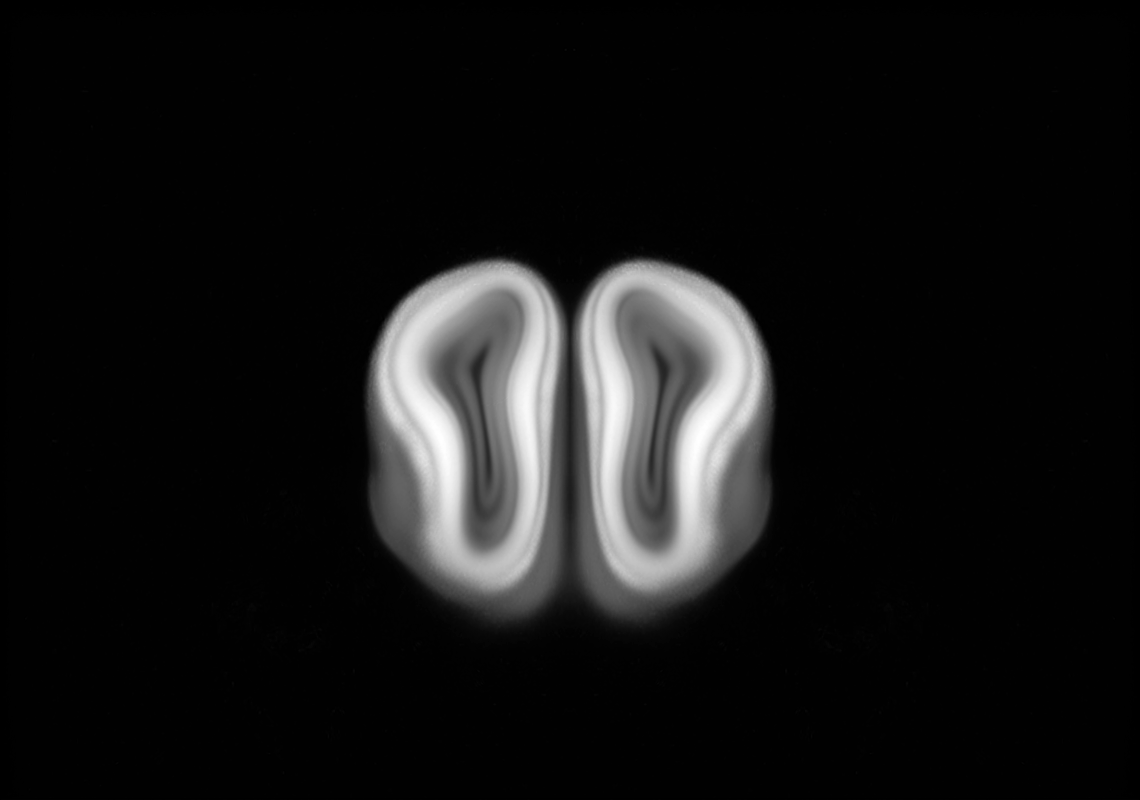

Supplement: Supplementary file 7 — Supplementary Data 5 [file 41467_2019_13057_MOESM7_ESM.zip › Suppl_File2_CCFbackground/AllenCCF_Z001.tif]

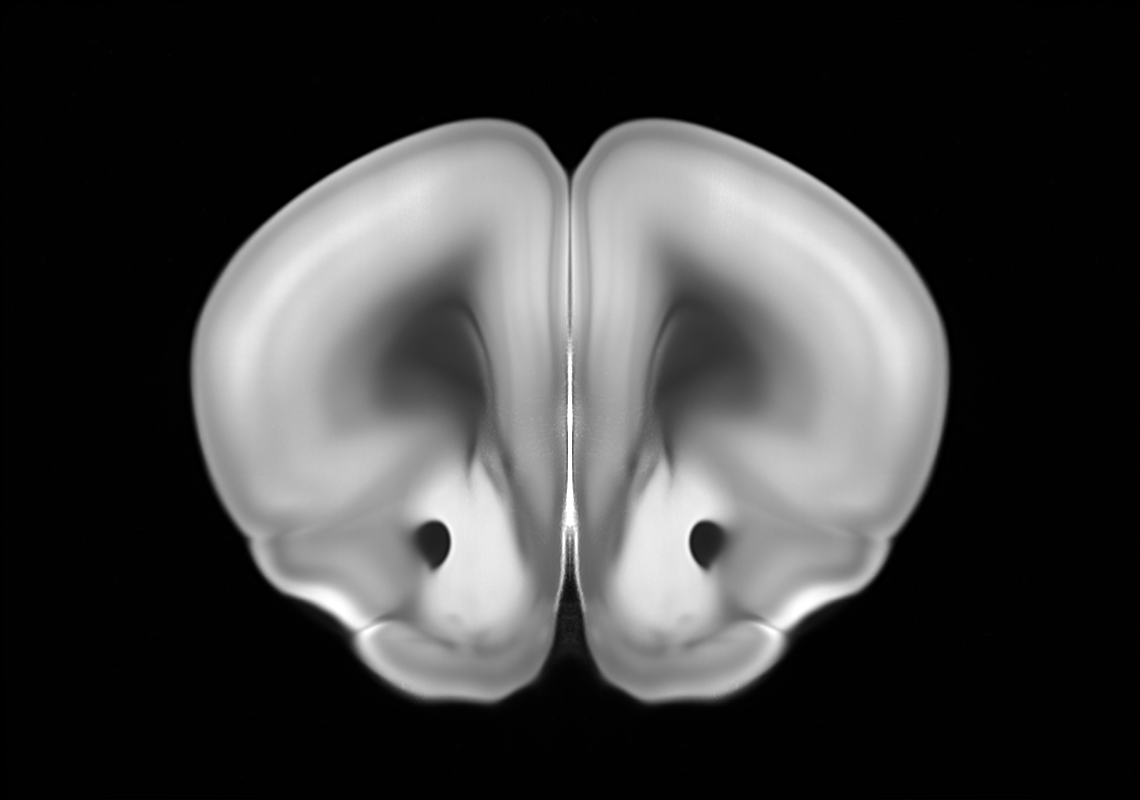

Supplement: Supplementary file 7 — Supplementary Data 5 [file 41467_2019_13057_MOESM7_ESM.zip › Suppl_File2_CCFbackground/AllenCCF_Z029.tif]

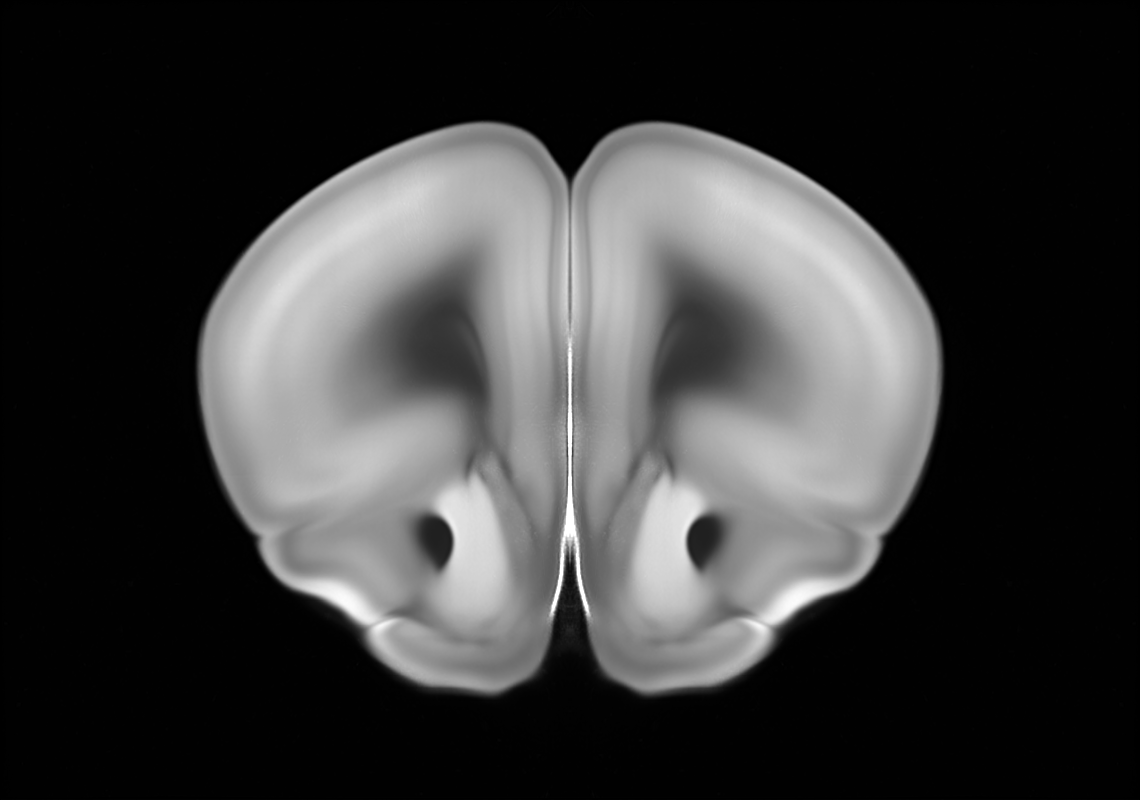

Supplement: Supplementary file 7 — Supplementary Data 5 [file 41467_2019_13057_MOESM7_ESM.zip › Suppl_File2_CCFbackground/AllenCCF_Z028.tif]

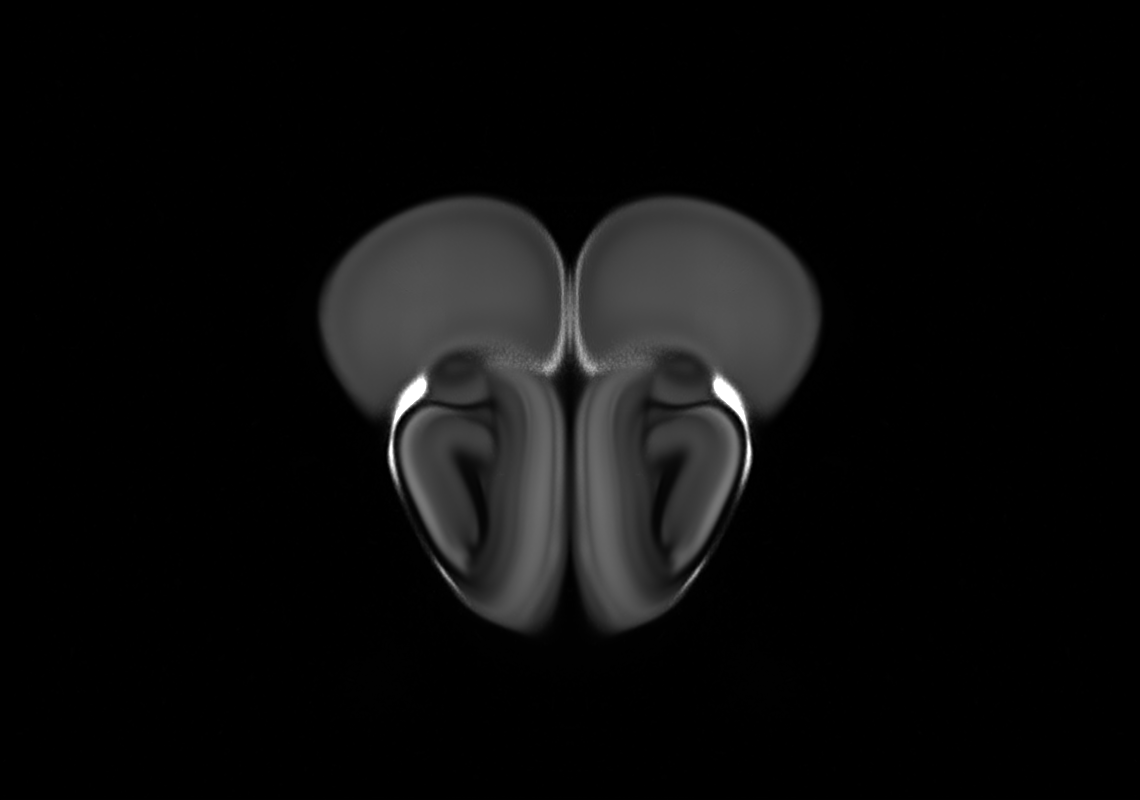

Supplement: Supplementary file 7 — Supplementary Data 5 [file 41467_2019_13057_MOESM7_ESM.zip › Suppl_File2_CCFbackground/AllenCCF_Z014.tif]

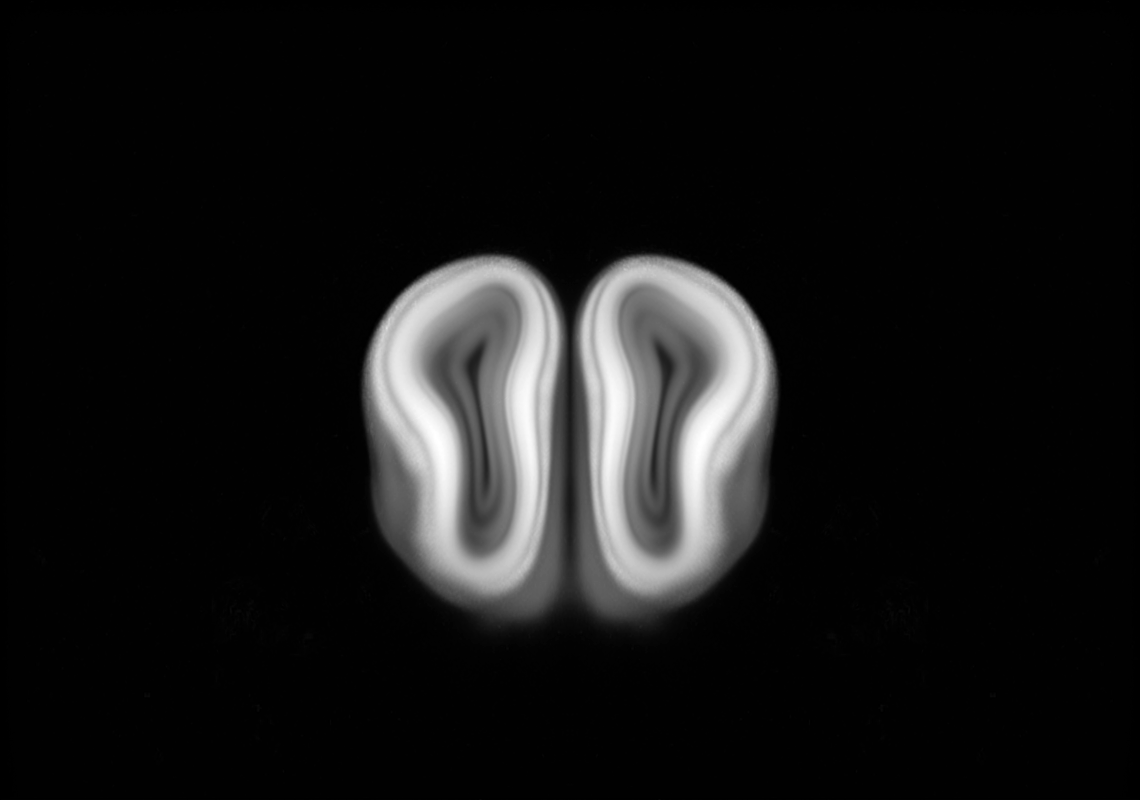

Supplement: Supplementary file 7 — Supplementary Data 5 [file 41467_2019_13057_MOESM7_ESM.zip › Suppl_File2_CCFbackground/AllenCCF_Z002.tif]

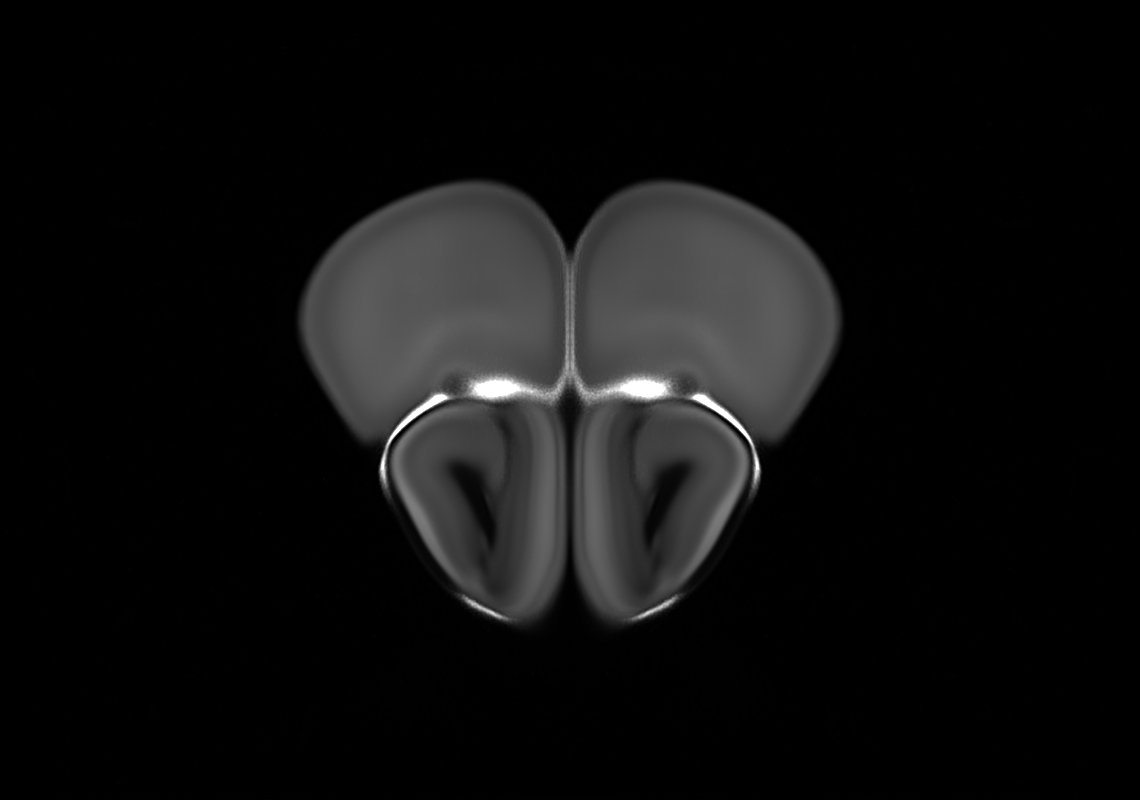

Supplement: Supplementary file 7 — Supplementary Data 5 [file 41467_2019_13057_MOESM7_ESM.zip › Suppl_File2_CCFbackground/AllenCCF_Z016.tif]

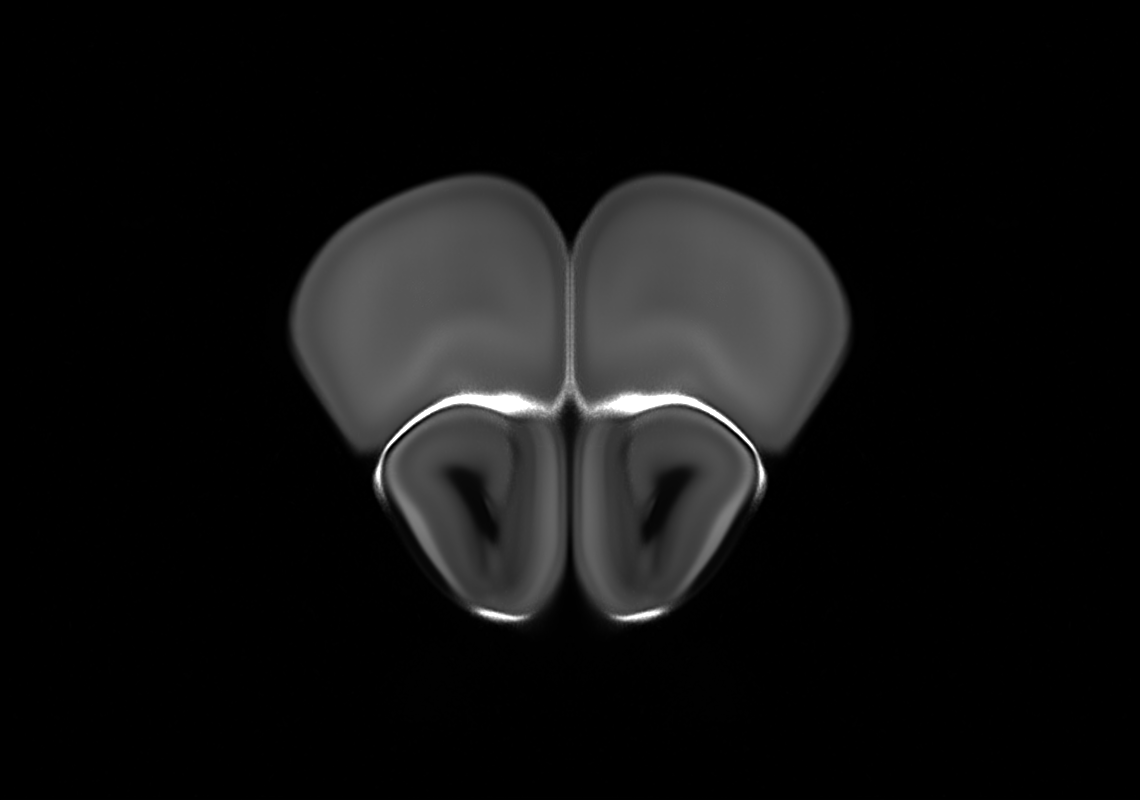

Supplement: Supplementary file 7 — Supplementary Data 5 [file 41467_2019_13057_MOESM7_ESM.zip › Suppl_File2_CCFbackground/AllenCCF_Z017.tif]

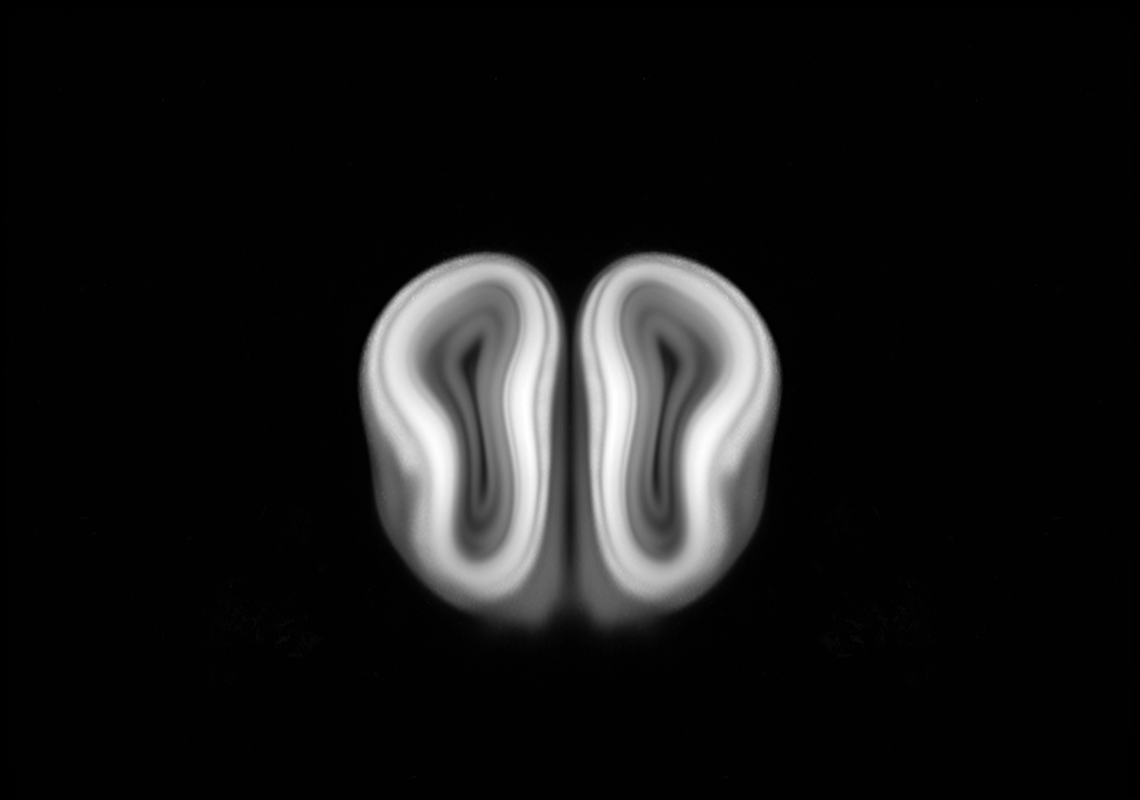

Supplement: Supplementary file 7 — Supplementary Data 5 [file 41467_2019_13057_MOESM7_ESM.zip › Suppl_File2_CCFbackground/AllenCCF_Z003.tif]

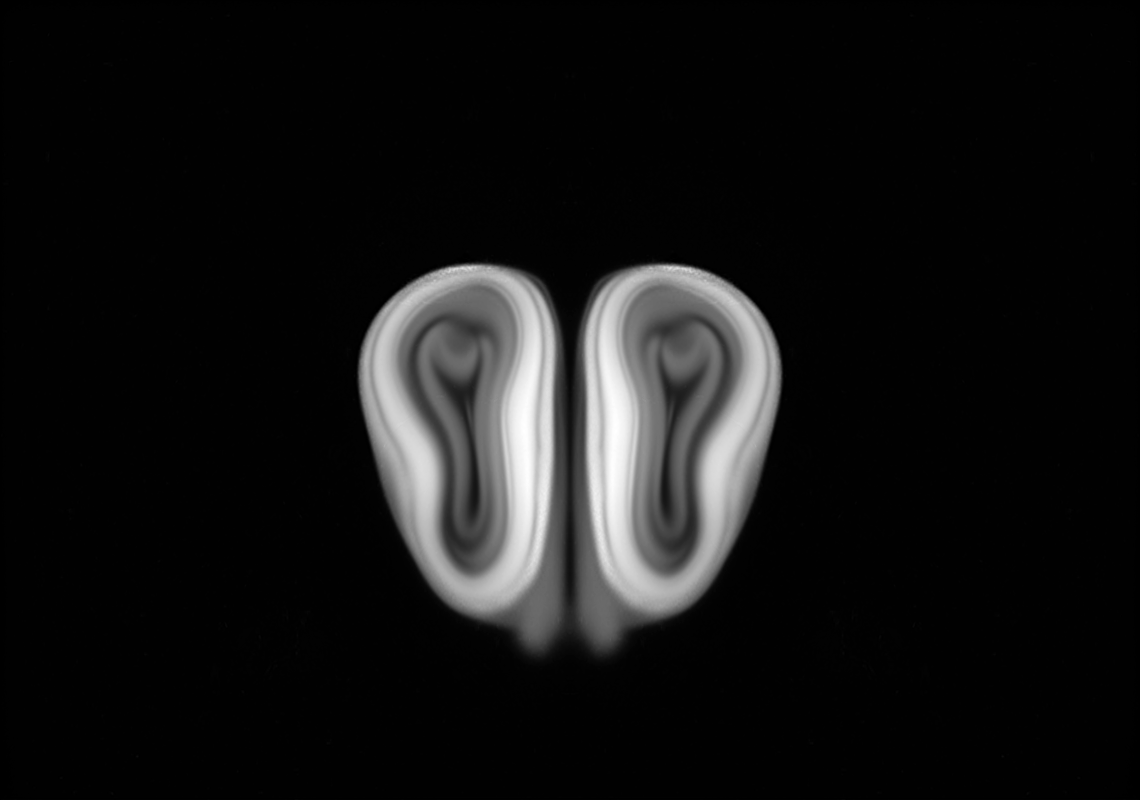

Supplement: Supplementary file 7 — Supplementary Data 5 [file 41467_2019_13057_MOESM7_ESM.zip › Suppl_File2_CCFbackground/AllenCCF_Z007.tif]

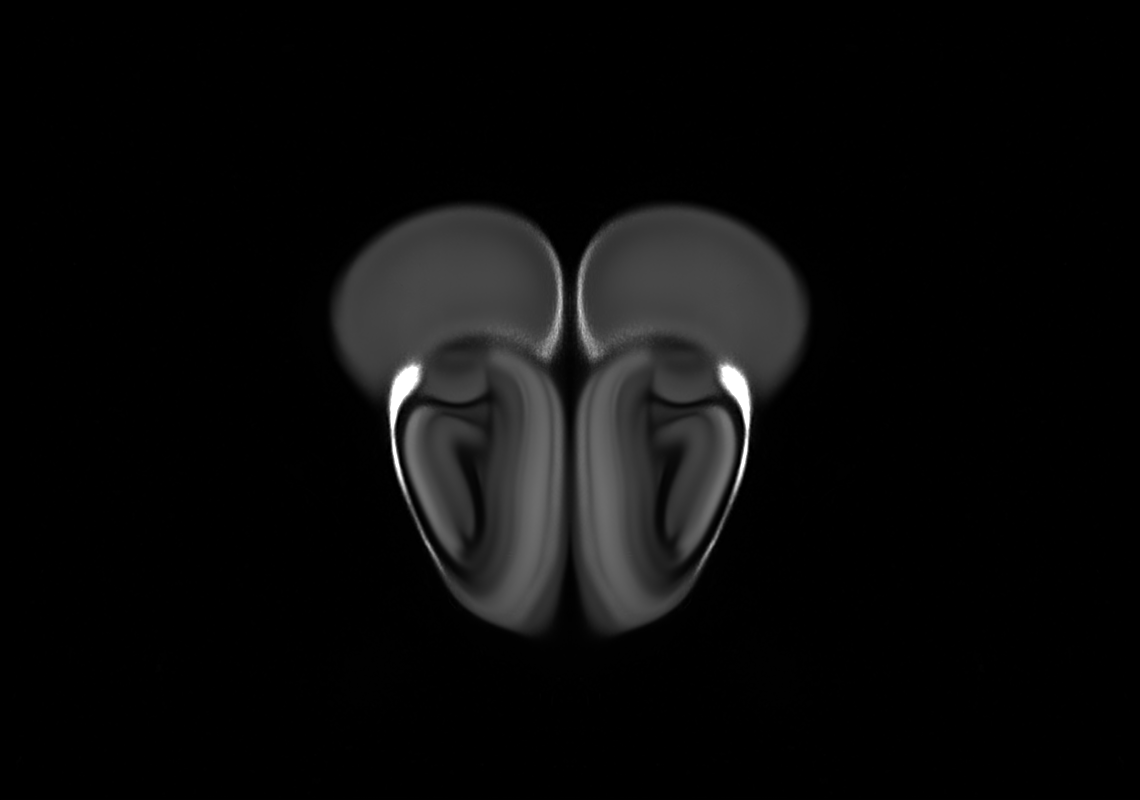

Supplement: Supplementary file 7 — Supplementary Data 5 [file 41467_2019_13057_MOESM7_ESM.zip › Suppl_File2_CCFbackground/AllenCCF_Z013.tif]

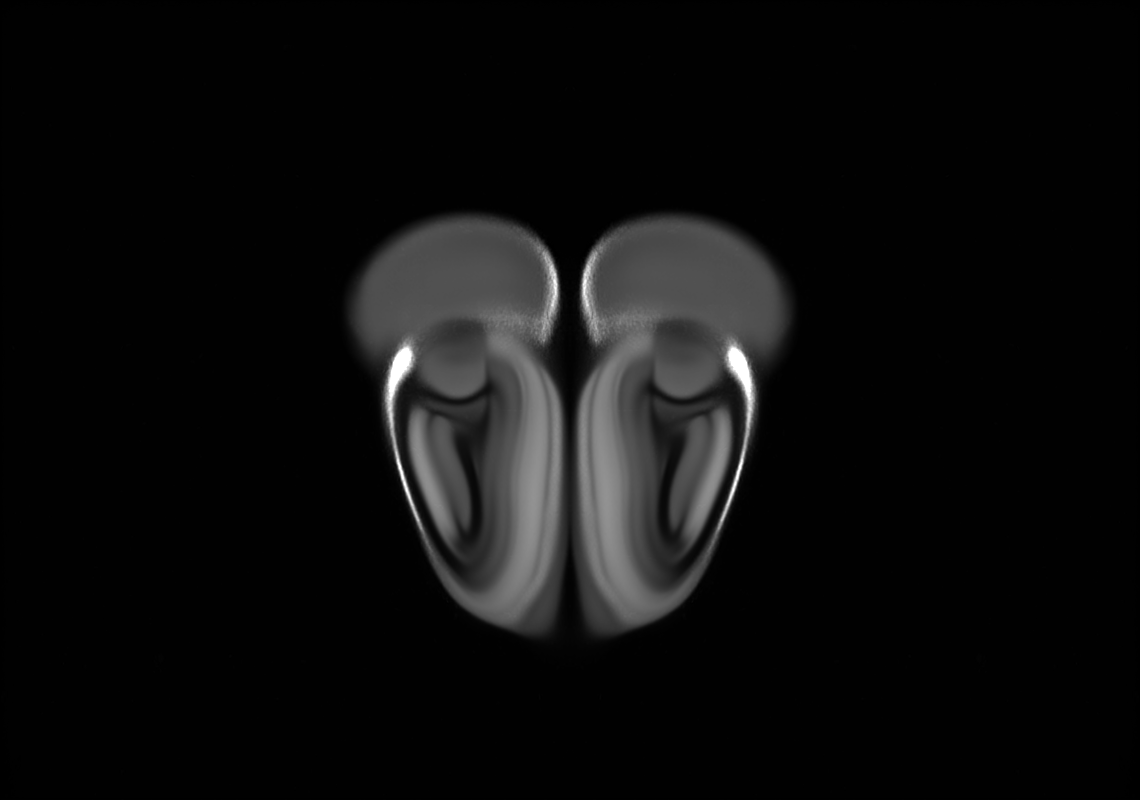

Supplement: Supplementary file 7 — Supplementary Data 5 [file 41467_2019_13057_MOESM7_ESM.zip › Suppl_File2_CCFbackground/AllenCCF_Z012.tif]

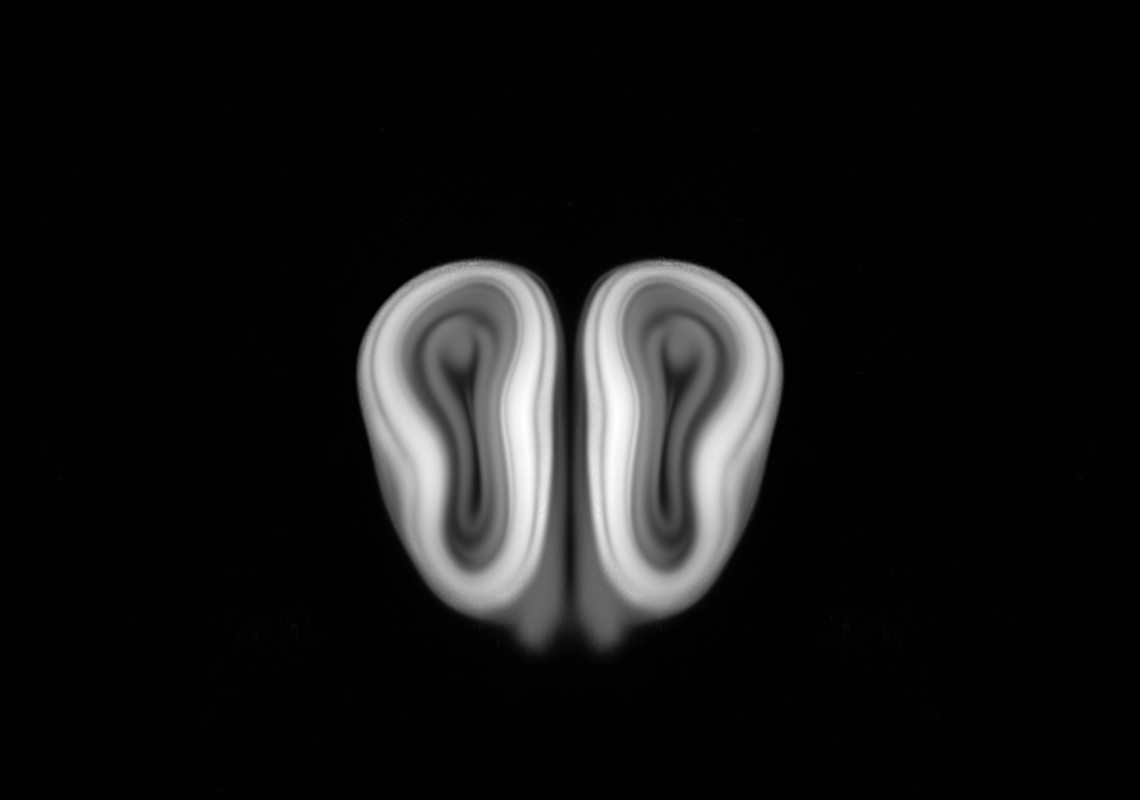

Supplement: Supplementary file 7 — Supplementary Data 5 [file 41467_2019_13057_MOESM7_ESM.zip › Suppl_File2_CCFbackground/AllenCCF_Z006.tif]

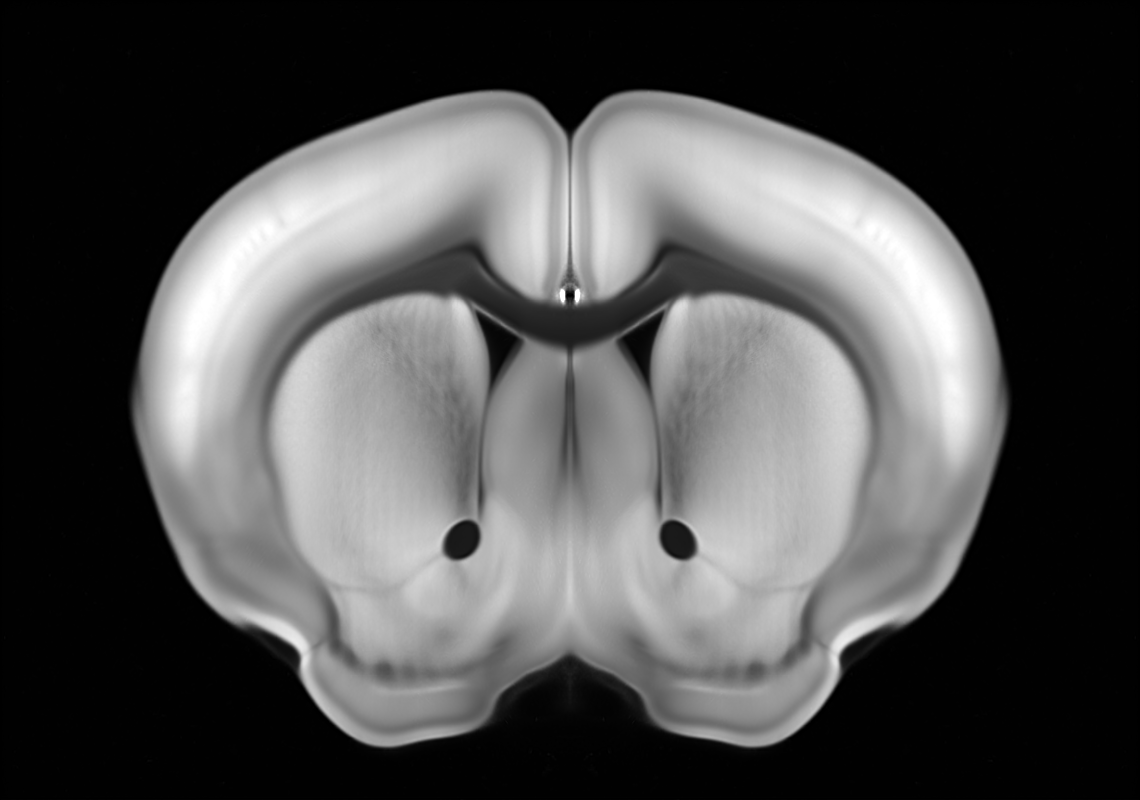

Supplement: Supplementary file 7 — Supplementary Data 5 [file 41467_2019_13057_MOESM7_ESM.zip › Suppl_File2_CCFbackground/AllenCCF_Z038.tif]

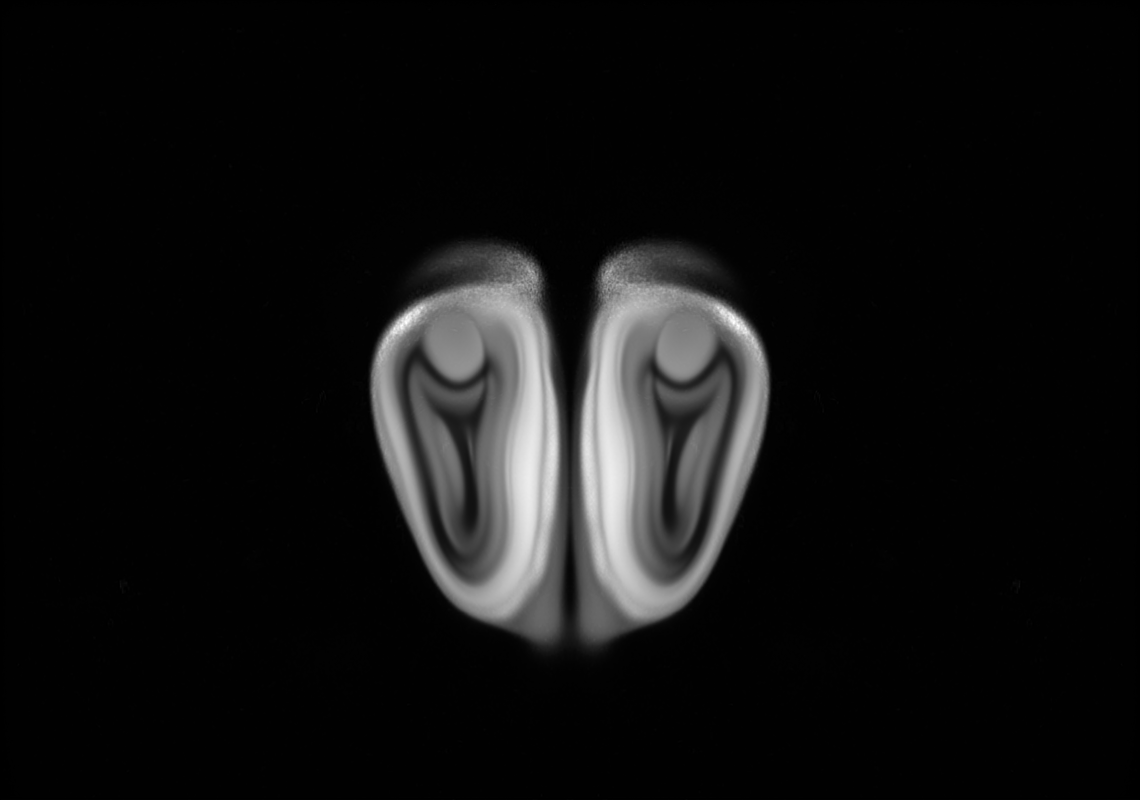

Supplement: Supplementary file 7 — Supplementary Data 5 [file 41467_2019_13057_MOESM7_ESM.zip › Suppl_File2_CCFbackground/AllenCCF_Z010.tif]

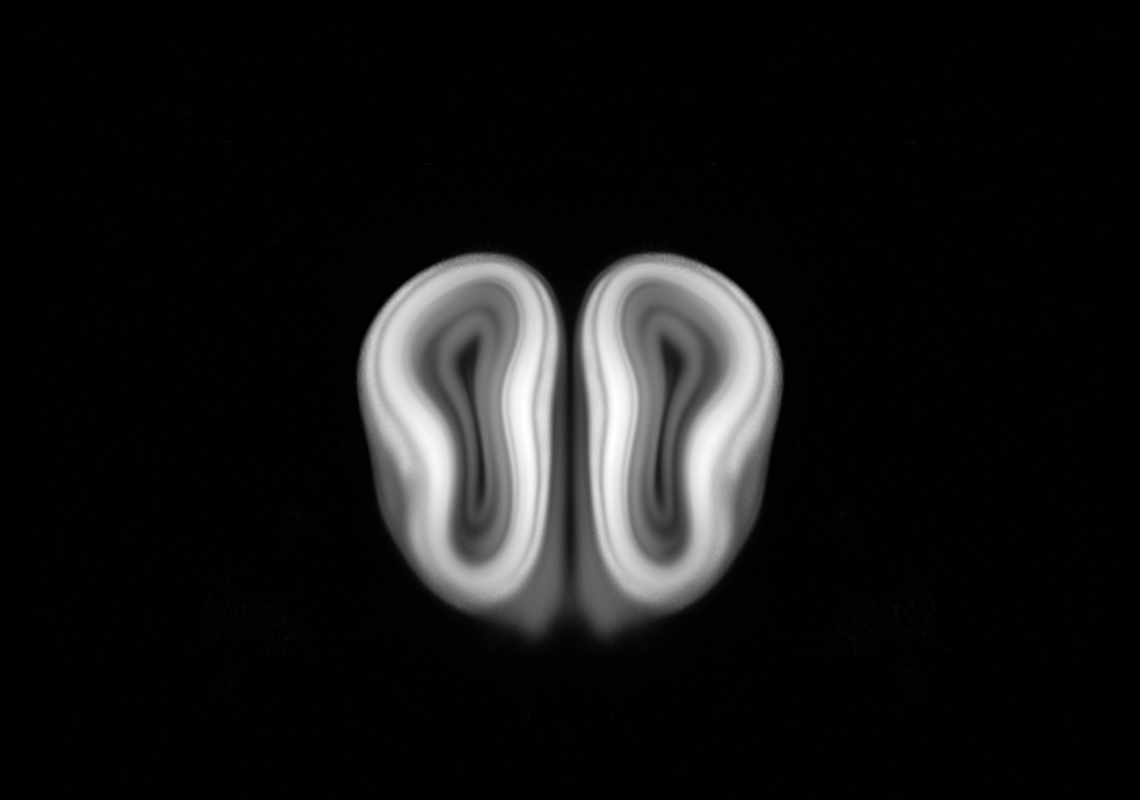

Supplement: Supplementary file 7 — Supplementary Data 5 [file 41467_2019_13057_MOESM7_ESM.zip › Suppl_File2_CCFbackground/AllenCCF_Z004.tif]

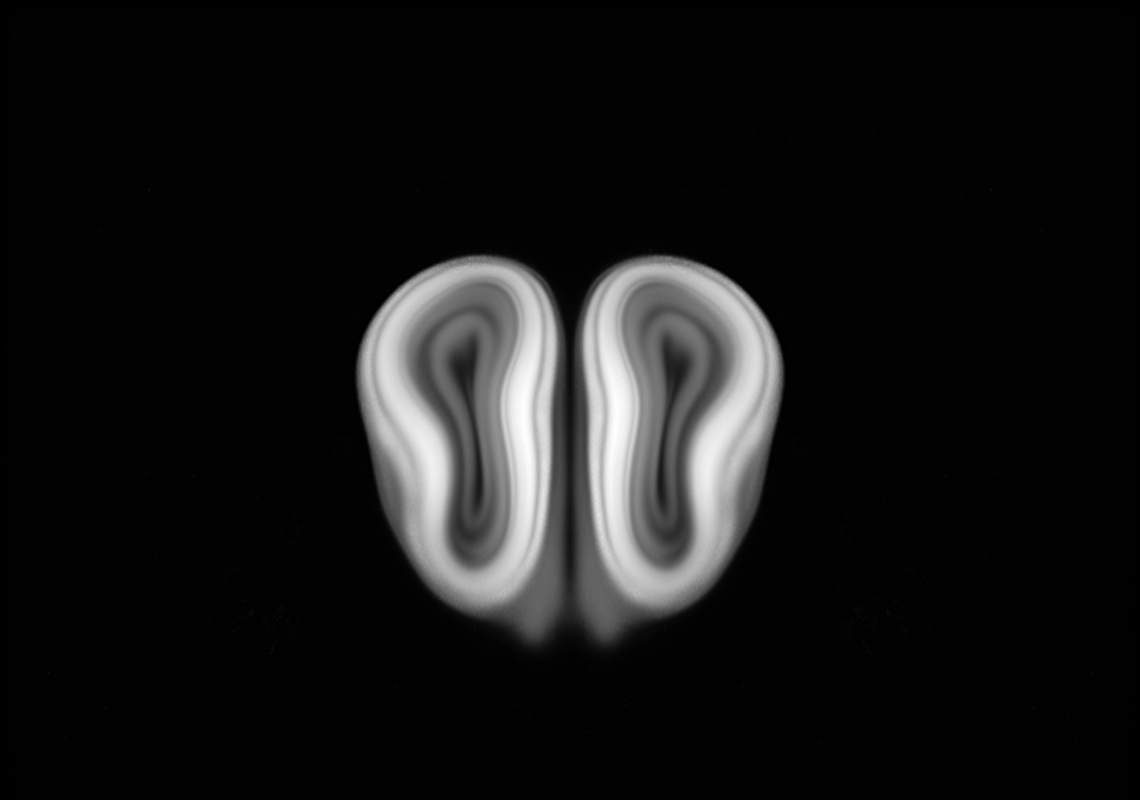

Supplement: Supplementary file 7 — Supplementary Data 5 [file 41467_2019_13057_MOESM7_ESM.zip › Suppl_File2_CCFbackground/AllenCCF_Z005.tif]

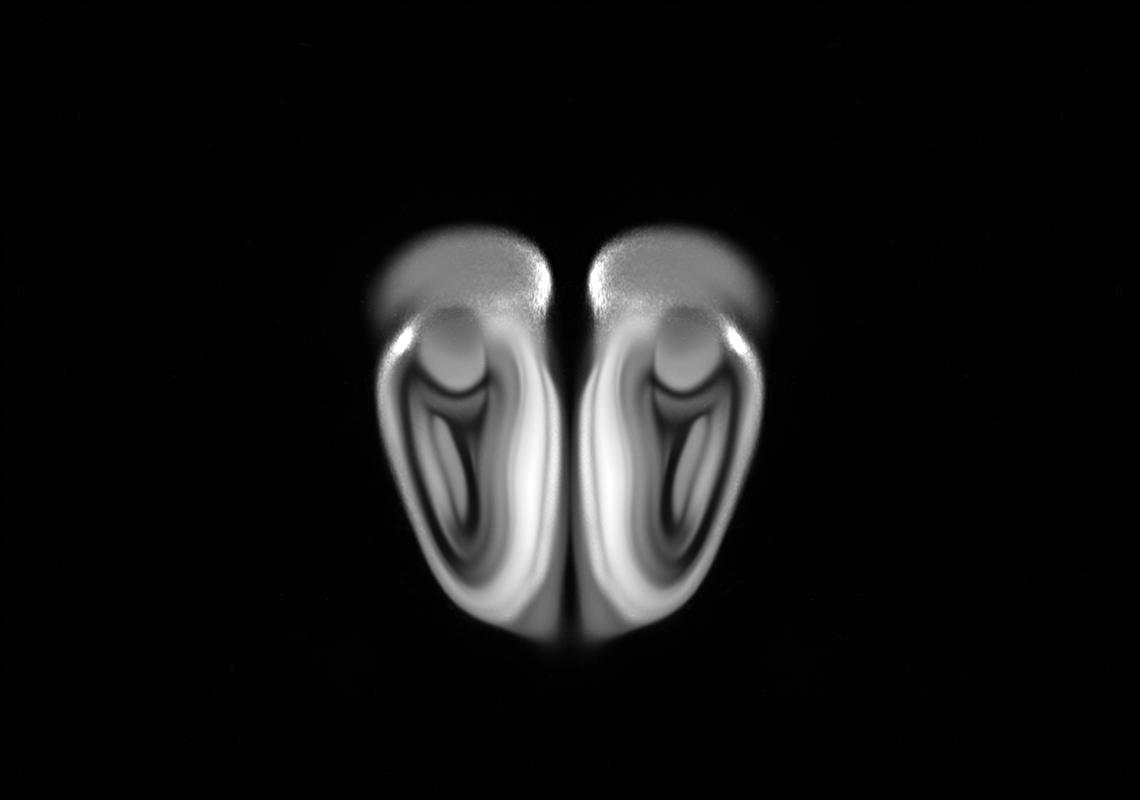

Supplement: Supplementary file 7 — Supplementary Data 5 [file 41467_2019_13057_MOESM7_ESM.zip › Suppl_File2_CCFbackground/AllenCCF_Z011.tif]

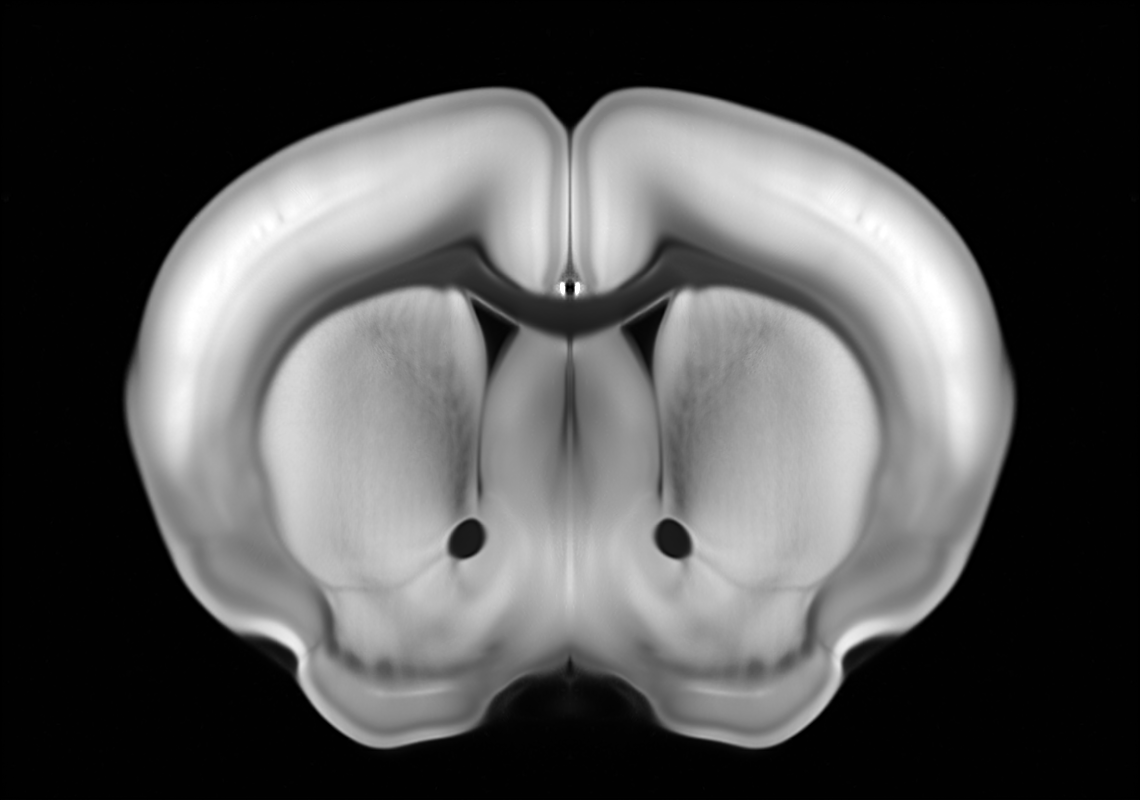

Supplement: Supplementary file 7 — Supplementary Data 5 [file 41467_2019_13057_MOESM7_ESM.zip › Suppl_File2_CCFbackground/AllenCCF_Z039.tif]
