# Supplementary material for: Enhanced and unified anatomical labeling for a common mouse brain atlas
Source: Nat Commun. 2019 Nov 7;10:5067. doi: 10.1038/s41467-019-13057-w (PMC6838086; doi:10.1038/s41467-019-13057-w)
Supplement: Supplementary file 11 — Reporting Summary [file 41467_2019_13057_MOESM11_ESM.pdf]

nature research

Corresponding author(s): Yongsoo Kim

Last updated by author(s): Sep 9, 2019

Reporting Summary

Nature Research wishes to improve the reproducibility of the work that we publish. This form provides structure for consistency and transparency in reporting. For further information on Nature Research policies, see Authors & Referees and the Editorial Policy Checklist.

Statistics

For all statistical analyses, confirm that the following items are present in the figure legend, table legend, main text, or Methods section.

n/a	Confirmed	
☒	☐	The exact sample size (*n*) for each experimental group/condition, given as a discrete number and unit of measurement
☒	☐	A statement on whether measurements were taken from distinct samples or whether the same sample was measured repeatedly
☒	☐	The statistical test(s) used AND whether they are one- or two-sided<br>*Only common tests should be described solely by name; describe more complex techniques in the Methods section.*
☒	☐	A description of all covariates tested
☒	☐	A description of any assumptions or corrections, such as tests of normality and adjustment for multiple comparisons
☒	☐	A full description of the statistical parameters including central tendency (e.g. means) or other basic estimates (e.g. regression coefficient) AND variation (e.g. standard deviation) or associated estimates of uncertainty (e.g. confidence intervals)
☒	☐	For null hypothesis testing, the test statistic (e.g. *F*, *t*, *r*) with confidence intervals, effect sizes, degrees of freedom and *P* value noted<br>*Give P values as exact values whenever suitable.*
☒	☐	For Bayesian analysis, information on the choice of priors and Markov chain Monte Carlo settings
☒	☐	For hierarchical and complex designs, identification of the appropriate level for tests and full reporting of outcomes
☒	☐	Estimates of effect sizes (e.g. Cohen's *d*, Pearson's *r*), indicating how they were calculated

Our web collection on statistics for biologists contains articles on many of the points above.

Software and code

Policy information about availability of computer code

Data collection	Custom built stitching algorithm to reconstruct images from serial two-photon tomography and Elastix for image registration were publicly distributed in the Kim et al., 2017 Cell, DOI: 10.1016/j.cell.2017.09.020). Elastix registration parameter files can be found in Supplementary Data 6.<br>Our python based code to perform Dice Similarity Coefficient calculation can be found in the Dryad data (https://doi.org/10.5061/dryad.t1g1jwsxw) under "6_Atlas-comaparison_Dice".<br>All codes can be used without any restriction.
Data analysis	Adobe Illustrator for vector drawing, FIJI (ImageJ) for anatomical label digitization

For manuscripts utilizing custom algorithms or software that are central to the research but not yet described in published literature, software must be made available to editors/reviewers. We strongly encourage code deposition in a community repository (e.g. GitHub). See the Nature Research guidelines for submitting code & software for further information.

Data

Policy information about availability of data

All manuscripts must include a data availability statement. This statement should provide the following information, where applicable:

- Accession codes, unique identifiers, or web links for publicly available datasets
- A list of figures that have associated raw data
- A description of any restrictions on data availability

Data that support the findings of this study and new data from the current study are available in Dryad data (https://doi.org/10.5061/dryad.t1g1jwsxw).
Additional data are available from the authors on reasonable request.
Following data are obtained from publically available sources.
- MRI labels (https://imaging.org.au/AMBMC/AMBMC): Hippocampus, cerebellum, cortex, Basal Ganglia, Diencephalon labels
- Allen Connectivity dataset (http://help.brain-map.org/display/mouseconnectivity/API): Injection dataset from isocortical areas from C57bl/6 mice

- Mouse Connectome Project (http://www.mouseconnectome.org/CorticalMap/page/map/5): Cortico-striatal projection map
- BICCN cell type data (http://www.brainimagelibrary.org/download.html): Chat_Ai75_M_382462, Emx1_Ai75_M_343525, Gad2_Ai75_M_398912, Ctgf-T2A_Ai75_M_395411, Ntsr1_Ai75_M_369820, Rbp4_Ai75_M_392433, Cux2_Ai75_M_384010.

Field-specific reporting

Please select the one below that is the best fit for your research. If you are not sure, read the appropriate sections before making your selection.

☒ Life sciences ☐ Behavioural & social sciences ☐ Ecological, evolutionary & environmental sciences

For a reference copy of the document with all sections, see nature.com/documents/nr-reporting-summary-flat.pdf

Life sciences study design

All studies must disclose on these points even when the disclosure is negative.

Sample size	For cell type specific transgenic marker brains, we use N = 1 per kind. Cell type specific transgenic mice showed very sterotypic distribution of labeled cells in anatomical regions. Thus, a single sample is sufficient to highlight labeled areas for our anatomical work.
Data exclusions	None
Replication	When we measure variability of cell counts within selected marker brains (parvalbumin, somatostatin, and VIP-Cre mice), the standard deviation of densities in anatomical areas was less than 10% of mean, which suggested highly stereotypic distribution of labeled cells in different brain regions.
Randomization	Our work does not include experiments requiring group comparision for statistical analysis. Thus, no randomization was used.
Blinding	Our anatomy work requires us to know natures of signals in order to be useful for anatomical delineations. Thus, no blinding was used.

Reporting for specific materials, systems and methods

We require information from authors about some types of materials, experimental systems and methods used in many studies. Here, indicate whether each material, system or method listed is relevant to your study. If you are not sure if a list item applies to your research, read the appropriate section before selecting a response.

Materials & experimental systems

n/a	Involved in the study	
☒	☐	Antibodies
☒	☐	Eukaryotic cell lines
☒	☐	Palaeontology
☐	☒	Animals and other organisms
☒	☐	Human research participants
☒	☐	Clinical data

Methods

n/a	Involved in the study	
☒	☐	ChIP-seq
☒	☐	Flow cytometry
☒	☐	MRI-based neuroimaging

Animals and other organisms

Policy information about studies involving animals; ARRIVE guidelines recommended for reporting animal research

Laboratory animals	For cell type specific labeling, we used oxytocin (OT)-Cre, and OT receptor (OTR)-Cre, and Avptm-Cre mice all crossed with Cre dependent reporter mice (Ai14). All animals were from 2 - 3 months old. Both males and females were used for the study.
Wild animals	No wild animals were used in the study.
Field-collected samples	No field-collected samples were used in the study.
Ethics oversight	All animal work has been approved by the Institutional Animal Care and Use Committee of Penn State University College of Medicine.

Note that full information on the approval of the study protocol must also be provided in the manuscript.
